# Contribution of autosomal rare and *de novo* variants to sex differences in autism

**DOI:** 10.1016/j.ajhg.2025.01.016

**Published:** 2025-02-14

**Authors:** Mahmoud Koko, F. Kyle Satterstrom, Autism Sequencing Consortium, Branko Aleksic, Mykyta Artomov, Mafalda Barbosa, Elisa Benetti, Catalina Betancur, Monica Biscaldi-Schafer, Anders D. Børglum, Harrison Brand, Alfredo Brusco, Joseph D. Buxbaum, Gabriele Campos, Simona Cardaropoli, Diana Carli, Angel Carracedo, Marcus C.Y. Chan, Andreas G. Chiocchetti, Brian H.Y. Chung, Brett Collins, Ryan L. Collins, Edwin H. Cook, Hilary Coon, Claudia I.S. Costa, Michael L. Cuccaro, David J. Cutler, Mark J. Daly, Silvia De Rubeis, Bernie Devlin, Ryan N. Doan, Enrico Domenici, Shan Dong, Chiara Fallerini, Montserrat Fernández-Prieto, Giovanni Battista Ferrero, Christine M. Freitag, Jack M. Fu, J. Jay Gargus, Sherif Gerges, Elisa Giorgio, Ana Cristina Girardi, Stephen Guter, Emily Hansen-Kiss, Gail E. Herman, Irva Hertz-Picciotto, David M. Hougaard, Christina M. Hultman, Suma Jacob, Miia Kaartinen, Lambertus Klei, Alexander Kolevzon, Itaru Kushima, So Lun Lee, Terho Lehtimäki, Lindsay Liang, Carla Lintas, Alicia Ljungdahl, Caterina Lo Rizzo, Yunin Ludena, Patricia Maciel, Behrang Mahjani, Nell Maltman, Marianna Manara, Dara S. Manoach, Gal Meiri, Idan Menashe, Judith Miller, Nancy Minshew, Matthew Mosconi, Rachel Nguyen, Norio Ozaki, Aarno Palotie, Mara Parellada, Maria Rita Passos-Bueno, Lisa Pavinato, Minshi Peng, Margaret Pericak-Vance, Antonio M. Persico, Isaac N. Pessah, Kaija Puura, Abraham Reichenberg, Alessandra Renieri, Kathryn Roeder, Stephan J. Sanders, Sven Sandin, F. Kyle Satterstrom, Stephen W. Scherer, Sabine Schlitt, Rebecca J. Schmidt, Lauren Schmitt, Katja Schneider-Momm, Paige M. Siper, Laura Sloofman, Moyra Smith, Christine R. Stevens, Pål Suren, James S. Sutcliffe, John A. Sweeney, Michael E. Talkowski, Flora Tassone, Karoline Teufel, Elisabetta Trabetti, Slavica Trajkova, Maria del Pilar Trelles, Brie Wamsley, Jaqueline Y.T. Wang, Lauren A. Weiss, Mullin H.C. Yu, Ryan Yuen, APEX consortium, Deep Adhya, Carrie Allison, Bonnie Ayeung, Rosie Bamford, Simon Baron-Cohen, Richard Bethlehem, Tal Biron-Shental, Graham Burton, Wendy Cowell, Jonathan Davies, Dori Floris, Alice Franklin, Lidia Gabis, Daniel Geschwind, David M. Greenberg, Yuanjun Gu, Alexandra Havdahl, Alexander Heazell, Rosemary Holt, Matthew Hurles, Yumnah Khan, Meng-Chuan Lai, Madeline Lancaster, Michael Lombardo, Hilary Martin, Jose Gonzalez Martinez, Jonathan Mill, Mahmoud Koko, Kathy Niakan, Adam Pavlinek, Lucia Dutan Polit, Marcin Radecki, David Rowitch, Laura Sichlinger, Deepak Srivastava, Alexandros Tsompanidis, Florina Uzefovsky, Varun Warrier, Elizabeth Weir, Xinhe Zhang, Varun Warrier, Hilary Martin

**Affiliations:** 1 Human Genetics, Wellcome Sanger Institute, Hinxton, Cambridgeshire CB10 1SA, UK; 2 Program in Medical and Population Genetics, Broad Institute of MIT and Harvard, Cambridge, MA, USA; 3 Stanley Center for Psychiatric Research, Broad Institute of MIT and Harvard, Cambridge, MA, USA; 4 Analytic and Translational Genetics Unit, Department of Medicine, Massachusetts General Hospital, Boston, MA, USA; 5 Department of Psychiatry, Autism Research Centre, University of Cambridge, Cambridge, Cambridgeshire CB2 8AH, UK

**Keywords:** exome sequencing, rare variant association, Autism Sequencing Consortium, ASC, Simons Foundation Powering Autism Research for Knowledge, SPARK

## Abstract

Autism is four times more prevalent in males than females. To study whether this reflects a difference in genetic predisposition attributed to autosomal rare variants, we evaluated sex differences in effect size of damaging protein-truncating and missense variants on autism predisposition in 47,061 autistic individuals using a liability model with differing thresholds. Given the sex differences in the rates of cognitive impairment among autistic individuals, we also compared effect sizes of rare variants between individuals with and without cognitive impairment or motor delay. Although these variants mediated different likelihoods of autism with versus without cognitive or motor difficulties, their effect sizes on the liability scale did not differ significantly by sex exome wide or in genes sex-differentially expressed in the cortex. *De novo* mutations were enriched in genes with male-biased expression in the adult cortex, but these genes did not show a significant sex difference on the liability scale, nor did the liability conferred by these genes differ significantly from other genes with similar loss-of-function intolerance and sex-averaged cortical expression. Exome-wide female bias in *de novo* protein-truncating mutation rates on the observed scale was driven by high-confidence and syndromic autism-predisposition genes. In summary, autosomal rare and damaging coding variants confer similar liability for autism in females and males.

## Teaser

This work evaluates the impact of rare coding variants on autism predisposition in 47,061 autistic individuals. It shows that the effect sizes of damaging autosomal variants on the liability scale do not differ significantly by sex, whether exome-wide or in genes sex-differentially expressed in the adult or fetal cortex.

## Introduction

Large-scale rare variant association studies in autism have shown that rare protein-coding variants contribute significantly to autism liability. Genes associated with autism—particularly those with strong statistical or molecular evidence—are enriched for loss-of-function (LoF)-intolerant genes expressed in the brain.^1^^,^^2^^,^^3^ It remains unclear whether rare variants contribute to the sex bias in autism; it is approximately four times more prevalent in males. This sex bias is less pronounced when autism is accompanied by cognitive impairment or motor developmental delay.^4^^,^^5^

The genetic underpinnings of autism include both rare and common variants.^6^^,^^7^^,^^8^ The collective effect of the different genetic factors predisposing to autism on the trait prevalence is typically studied using the liability threshold model.^4^ This model is often used to explain how additive genetic factors relate to a dichotomous diagnosis—by postulating that the combined effect of these predisposition factors in the population is normally distributed and that individuals diagnosed with autism will have exceeded a certain diagnostic threshold.^9^ Under this model, a sex difference in population prevalence may arise either because there is a higher threshold in females relative to males so that females require a higher genetic predisposition to be diagnosed^10^ or due to a single threshold with sex-biased effect sizes (gene-by-sex interaction) causing the same set of variants to push males, but not females, past the threshold.^11^ Unlike comparisons on the observed scale (e.g., fold enrichment in variant rates in females versus males), examining effect sizes on the liability scale allows for a direct comparison between groups with different proportions of individuals with the trait.

Previous work on 12,270 autistic individuals showed that females have a higher burden of rare damaging variants.^12^ However, it was not clarified if those observed differences translate into differences in liability. A separate analysis of overlapping cohorts (11,986 autistic individuals) by the Autism Sequencing Consortium (ASC) suggested that the observed significant sex differences in *de novo* mutation (DNM) enrichment did not translate into differences in the average effect size attributed to these damaging variants on the liability scale.^2^ Importantly, autistic individuals show different rates of DNMs and over-transmission of damaging rare alleles depending on the presence of co-occurring cognitive impairment or dysmorphism.^13^^,^^14^ More recent ASC work on 20,627 autistic individuals did not show evidence for gene-by-sex interaction among those harboring rare variants in autosomal genes significantly associated with autism and suggested that sex and phenotypic severity additively associate with rare variant burden in these autism-predisposition genes.^1^ Given that a larger proportion of autistic females than males have co-occurring cognitive impairment, potentially because of an underdiagnosis of autistic females with otherwise typical cognitive development,^15^^,^^16^ it is unclear if the observed sex difference in rare variant rates is simply a reflection of differences in the proportion of individuals with cognitive impairment between sexes.^17^^,^^18^

Larger, more recently released cohorts like the Simons Foundation Powering Autism Research for Knowledge (SPARK), which includes more than 40,000 autistic individuals, offer a chance to examine rare variant liability and its relation to autism and co-occurring conditions in depth. Here, we meta-analyzed the ASC and SPARK datasets^1^^,^^14^ of exome-sequenced samples to explore whether there is a sex difference in rare variant liability, both exome wide and in focused gene sets of high-confidence autism-associated genes and genes with sex-biased expression in the fetal and adult cortex (Figure S1). We performed sex-stratified analyses of rare missense and protein-truncating variants (PTVs) in exonic coding regions in 47,061 autistic individuals and 25,593 siblings or control individuals not diagnosed with autism from cohorts curated by SPARK and the ASC (Table S1). We then explored the relationship between the sex differences in liability and cognitive or motor difficulties co-occurring with autism.

## Subjects and methods

### A note on terminology

We use neutral terminology, including “autistic individuals,” throughout the manuscript, in line with the preferences of a large number of autistic people. However, we use standard statistical terminology (e.g., liability, liability threshold model, risk ratio, and gene burden) to be consistent with other literature.

### Ethics and approvals

We confirm that the datasets used for this study were obtained from research projects complying with relevant ethical regulations. The procedures followed were in accordance with the ethical standards of the responsible committee(s) on human experimentation (institutional and national), and proper informed consent was obtained. The ASC studies^1^^,^^2^ were approved by Mass General Brigham Human Research Committee Institutional Review Board (IRB) protocol nos. 2012P001018 and 2013P000323. Access to SPARK phenotypic and genetic data^14^ was approved by the Simons Foundation Autism Research Initiative (SFARI). SPARK participants were recruited under Western IRB protocol no. 20151664.

### SPARK cohort

We used the second version of the integrated whole-exome sequencing data release^14^ ("iWES2"; Figure S2) which spanned five sequencing waves (WES1–5) encompassing 106,744 individuals (44,304 of them were diagnosed with autism, and the rest were non-autistic parents, siblings, and a few extended family members). These included 25,386 trios (18,172 autistic individuals and 7,214 not diagnosed with autism), 23,346 samples with one sequenced parent (17,644 autistic individuals and 5,702 not diagnosed with autism), and 58,012 samples without parental sequences (8,488 autistic individuals and 49,524 not diagnosed with autism—with the latter group formed mostly of parents of other individuals in the trio-/pair-sequenced groups, i.e., few multi-generational families). (See Tables S3 and S4 for sample size in the different genetic ancestry groups.)

First, we performed exome quality control (QC) on all samples in iWES2, as detailed in supplemental methods section 1. Briefly, we annotated the variant calls with coding consequences on Matched Annotation from NCBI and Ensembl (MANE) transcripts (Ensembl release 108; genome build GRCh38) and filtered for variants having synonymous or more damaging consequences, prioritizing the most severe consequence when two genes were affected. We removed variants failing a random forest quality filter (Figure S3); genotypes with low depth (DP < 10), low genotype quality (GQ < 10), or low variant allele fraction (VAF < 0.25); and outlier samples on these metrics: total and singleton variant count, transition-transversion ratio, insertion-deletion ratio, and heterozygous-homozygous ratio.

We then excluded all samples that were potentially part of the ASC cohort (by removing all individuals in SPARK who indicated their previous participation in ASC studies), parents and siblings reported to have a developmental disorder/motor delay or cognitive impairment, and autistic parents. We then defined a set of maximally unrelated probands and maximally unrelated siblings by incrementally removing individuals with the highest number of related people (within each of these two subsets) while preferentially retaining females. Following QC, we evaluated the genotypes of 20,236 trio-sequenced individuals (13,473 with autism and 6,763 not diagnosed with autism) to identify rare DNMs and inherited variants (SPARK and gnomAD minor-allele frequency < 0.1%; see supplemental methods section 3.2 for details).

We also did supplementary analyses in which we examined ultra-rare inherited variants (in one SPARK family and not in gnomAD) in an additional 18,816 child-parent pairs with one sequenced parent (13,435 with autism and 5,381 not diagnosed with autism) and ultra-rare variants (allele frequency < 0.005%) of undetermined origin in 8,905 individuals without sequenced parents (6,533 with autism and 2,372 not diagnosed with autism). Further details on rare and ultra-rare variant filtering are available in supplemental methods section 4. (See Table S5 for the sample size after QC.)

### ASC cohort

The QC of this dataset is described elsewhere in the context of a large rare variant association analysis.^1^ This previous analysis primarily examined both sexes jointly using data from the Simons Simplex Collection and smaller ASC family-based cohorts (Figure S4), the SPARK Pilot and first exome sequencing wave (WES1), and Swedish and Danish case-control cohorts. From the ASC, we received sex-stratified gene-level rare variant counts^1^^,^^2^ (gnomAD minor-allele frequency < 0.1%) grouped by their mode of inheritance into DNMs (in probands or siblings) or inherited variants (transmitted or untransmitted in the probands).

DNM counts came from 10,488 individuals in the ASC family-based cohort only (8,028 autistic individuals and 2,460 not diagnosed with autism) and did not include those ascertained in SPARK Pilot and WES1 families and so were independent of the SPARK iWES2 dataset presented in the previous section. Some of the DNMs in the ASC cohort were collated from older studies and did not have accompanying information on inherited alleles. Therefore, inherited variants were evaluated in 9,929 of the 10,488 individuals for whom we had DNMs (7,570 autistic children and 2,359 siblings). We also obtained ultra-rare variant counts (allele frequency = ∼0.005%) from 14,188 individuals from the ASC case-control cohorts (5,591 autistic individuals and 8,597 not diagnosed with autism). Table S2 shows the number of probands, siblings, and parents across the different ASC sites. See supplemental methods sections 3.1 (trios dataset) and 4.2 (case-control dataset) for more details.

### *De novo* and inherited variants

We analyzed DNMs, rare transmitted variants, and rare untransmitted variants annotated as damaging PTVs, damaging missense variants, or synonymous variants. The analysis was limited to 17,296 protein-coding genes annotated in both the ASC and SPARK after QC. PTVs were considered damaging if they occurred in 1,742 highly LoF-intolerant genes in the most-constrained decile for the LoF observed over expected upper bound fraction (LOEUF) score, and missense variants were considered damaging if they had a missense badness, PolyPhen, and constraint (MPC) score ≥ 2 (in all genes). For additional sensitivity analyses, we relaxed the filtering threshold to include PTVs in the 2^nd^ or 3^rd^ LOEUF deciles (1,765 and 1,781 genes, respectively) and missense variants with MPC scores ≥1.

We carried out sex-stratified comparisons (autistic individuals against sex-matched siblings) as well as direct comparisons between sexes (autistic females versus autistic males) as described in the next section. The primary analysis of *de novo* and rare inherited variants (allele frequency < 0.1%) was performed in the trio-sequenced individuals in both SPARK and ASC (21,501 autistic individuals and 9,223 siblings not diagnosed with autism). (See Table S6 for a list of trio-sequenced samples and Table S7 for a list of these DNMs.) The remaining data (individuals with sequence data from one or neither parent) were used for analyses of ultra-rare variants.

### Exome-wide enrichment

The following statistical analyses are described in detail in supplemental methods section 5 and summarized briefly here. We used the ratio between the rate of DNMs in the probands (DNMs per sample) and the rate of DNMs in the siblings as a measure of enrichment.^1^^,^^2^ For inherited variants, we calculated the ratio between parental alleles transmitted to the probands and the remaining untransmitted alleles. A ratio of 1 in the context of DNM analysis means that the probands and siblings have equal rates of rare DNMs; in the context of transmission analysis, it means that there is transmission equilibrium (half of the rare parental alleles are transmitted to the probands). For simplicity, we may refer to both ratios as the “rate ratio.”

To test for the significance of observed deviations from a DNM rate ratio 1, we used a two-sided binomial exact test to compare the DNM counts in the probands and the siblings. This tested whether the proportion of DNMs seen in the probands (from all DNMs in the probands and siblings) is significantly different from the proportion expected given their sample size (expected rate = *n*_probands_/(*n*_probands_ + *n*_siblings_)). For inherited variants, we used a two-sided binomial exact test to compare the counts of transmitted and untransmitted alleles in the probands, examining whether the fraction of parental alleles transmitted to the probands was significantly different from 0.5. We obtained the confidence intervals (CIs) for the rate ratio from these binomial tests. Comparisons of variant rates between autistic individuals and control subjects in the case-control dataset were evaluated the same way as DNMs.

In direct tests of autistic females and males, we used the same method described above (a binomial test) to compare the fraction of total DNM counts (i.e., total in autistic males and females) that were observed in autistic females with the fraction expected given the fraction of all autistic individuals that were female. For transmission analysis, we calculated the ratio between the total number of parental alleles in autistic females and the total number in both autistic males and females and used this as the expected ratio for a binomial test comparing the transmitted variant counts in autistic females and the total transmitted variants (in both autistic males and females).

We performed these tests separately for each sex in SPARK and ASC and meta-analyzed the rate ratios for the sex-stratified comparisons using the inverse variance-weighted average of the rate ratios. In these exome-wide comparisons, the *p* values from the binomial test were conservatively corrected for 54 tests (using Bonferroni correction) from these groups: three sex-stratified comparisons (males, females, and sex difference), three cohorts (ASC, SPARK, and meta-analysis of both), three variant classes (synonymous, missense, and protein truncating), and two inheritance models (*de novo* and transmitted). We also used the Benjamini-Hochberg false discovery rate (FDR) adjustment, as the Bonferroni correction is conservative given the non-independence of the meta-analyzed estimates. We used asterisks in the figures to indicate whether the *p* values were <0.05 after Bonferroni correction (^∗∗∗^), after FDR adjustment (^∗∗^), or only before correction (^∗^).

### Variant liability

Assuming that autism liability is additive and normally distributed in the general population, the difference in the average liability in individuals who harbor particular variants and the average in the general population is a measure of the average effect size of these variants, i.e., an estimate of how far this group of variants pushes individuals harboring them (on average) on the liability scale. This variant liability can be estimated from variant rates in the study cohorts, as detailed previously.^2^ The procedure we used to calculate the estimates is depicted in Figure S5 and detailed in supplemental methods section 6.1.

We used an autism population prevalence estimate of 2.5% in males (1 in 40), with a male-to-female prevalence ratio^19^ of 4:1. We took the *p* values obtained from a binomial test comparing the variant counts in the probands and siblings (outlined above) and estimated the standard errors of the average liability estimates and, subsequently, the 95% CIs. These calculations were performed separately for each sex and each cohort and then meta-analyzed between cohorts. To directly compare autistic females and males, we calculated the difference between the variant liability estimates obtained separately in female and male probands (*Z* score difference). We corrected the *p* values for multiple testing in a similar manner to the exome-wide enrichment (54 tests).

Moreover, we explored whether removing 354 high-confidence and syndromic autosomal genes curated by the SFARI^20^—hereafter, SFARI genes—would uncover any sex-biased exome-wide variant liability. Under a model where females have a higher liability threshold than males (a "different-threshold" model), the sex ratio of damaging mutations in a gene or group of genes (i.e., higher rates in females) would be inversely correlated with the penetrance of those mutations,^4^ with highly penetrant mutations being more prevalent in females. Because SFARI genes constitute a group of genes with higher autism penetrance relative to the remaining genes, they would be expected to show a higher sex bias on the observed scale (but similar effects on liability). Removing them would thus enrich the analysis with genes mediating lower risk for autism, helping to highlight any underlying sex bias in effect sizes on the liability scale if it exists. PTVs in SFARI gene set were considered damaging if they occurred in 218 SFARI genes that are highly LoF intolerant (in the most-intolerant LOEUF decile), whereas missense variants were considered damaging if they had an MPC score ≥ 2 (in all 354 genes).

### Autism with co-occurring cognitive difficulties

To explore how genetic architecture differed by phenotype, we split the autistic individuals in the ASC and SPARK cohorts into those with autism with co-occurring cognitive impairment and those with autism who otherwise had typical cognitive development or did not have reported information to allow classification (see supplemental methods section 2). We then compared each of these with siblings not diagnosed with autism as control subjects.

In the ASC cohort, we leveraged predefined categories of coexisting cognitive difficulties. Here, we separately tested rare variant enrichment in autistic individuals with co-occurring cognitive impairment (defined by the ASC as a full-scale intelligence quotient (IQ) score < 70, a Human Phenotype Ontology term indicating intellectual disability/cognitive impairment, or an International Classification of Diseases code indicating this diagnosis). The remaining autistic individuals with unknown cognitive impairment status and those with borderline and average IQs were grouped together. For the DNM analysis, there were 1,519 autistic males and 387 females with cognitive impairment (∼23% of 6,615 autistic males and ∼27% of 1,413 autistic females; relative risk of cognitive impairment in females versus males = 1.19). For the over-transmission analyses, which did not include data from previously published work focused on DNMs, there were 1,357 autistic males and 335 females with cognitive impairment (∼22% of 6,249 autistic males and ∼25% of 1,321 autistic females; relative risk = 1.17). (See Figure S4 and Table S11 for the percentage of autistic individuals with cognitive impairment across the contributing ASC sites.)

We classified SPARK individuals in a roughly similar manner to the ASC, i.e., one group for those with cognitive impairment (reported diagnosis of cognitive impairment or an IQ < 70) and another group for those without cognitive impairment or with an unknown cognitive impairment status. The proportions of those with cognitive impairment in SPARK trios were ∼23% among 10,482 males (*n* = 2,424) and ∼26% among 2,991 females (*n* = 775), with a relative risk of 1.12.

When estimating liability in the group with autism and cognitive impairment, we scaled the sex-specific population prevalence using the observed percentages of autistic individuals with cognitive impairment, i.e., using prevalence estimates of ∼0.58% in males (23% × 2.5%) and ∼0.16% in females (26% × 0.625%) for liability calculations; we subtracted these estimates from the total autism prevalence for each sex to estimate the liability in the second group of autistic individuals without cognitive impairment or with unknown cognitive impairment status. Male-specific *Z* scores were subtracted from female-specific estimates to assess the sex difference.

This phenotypic grouping did not consider motor delay, another important co-occurring condition.^2^ To address these deficiencies, we performed a separate analysis in SPARK, in which we had more detailed phenotype data. Specifically, we defined two groups of autistic individuals with cognitive or motor impairment or without these conditions, excluding those with unknown status (see supplemental methods section 2.2).

Here, SPARK individuals reported to have an IQ < 80, cognitive impairment (reported professional diagnosis of an intellectual disability, cognitive impairment, global developmental delay, or borderline intellectual functioning), or motor delay (reported professional diagnosis of delay in walking or developmental coordination disorder) constituted the “autism with motor or cognitive impairment” group (4,209 trio sequenced, 4,714 with one sequenced parent, and 1,778 without sequenced parents). We chose the IQ cutoff of 80 since it was previously suggested that defining cognitive impairment in SPARK based on this cutoff minimizes the grouping of average and borderline IQ individuals together and that a diagnosis of an intellectual disability does not necessarily require an IQ < 70.^21^ Individuals reported not to have any of these co-occurring conditions formed the “autism without motor or cognitive impairment” group (7,420 trio sequenced, 6,938 with one sequenced parent, and 2,141 without sequenced parents), whereas those with missing data on these phenotypes (1,844 trio sequenced, 1,756 with one sequenced parent, and 2,615 without sequenced parents) were considered unclassified.

Among 11,630 autistic individuals in SPARK who could be classified, ∼36% fell in the autism with motor or cognitive impairment group (among trios: 35% in males and 40% in females). The liability was estimated using prevalence estimates of ∼0.88% in males (35% × 2.5%) and 0.25% in females (40% × 0.625%) in the autism with motor or cognitive impairment group; for the autism without motor or cognitive impairment group, we used prevalence estimates of ∼1.63% in males (2.5%–0.88%) and ∼0.38% in females (0.625%–0.25%). Male-specific *Z* scores were subtracted from female-specific estimates to assess the sex difference.

### Gene set burden

We evaluated the rare variant burden in SFARI genes and genes with sex-biased expression derived from meta-analyses of two bulk RNA sequencing (RNA-seq) datasets of human fetal cortical tissues^22^ or two bulk RNA-seq datasets of adult human cortical tissues.^23^ We examined these gene sets directly and also gauged the extent of the observed enrichment against the burden expected for a similarly sized gene set selected from the remaining protein-coding genes. For each tested gene set, we selected a random gene set matched for LoF constraint, brain expression, and coding sequence length distribution (described further in supplemental methods section 7.3) and counted DNMs, transmitted variants, and untransmitted variants. We repeated this procedure 10,000 times with replacement and took the average ratio (rate ratio between DNM counts in probands and siblings or transmitted-to-untransmitted ratio in the probands) and then used this ratio as the expected ratio in a binomial test as described above. Specifically, we tested the difference between the rate of DNMs between probands and siblings against the permutation-averaged expected ratio for this gene set (instead of the sample size ratio used in the exome-wide analyses), and we similarly tested rare variant over-transmission against the permutation-averaged expected transmitted-to-untransmitted ratio for the given gene set (instead of 0.5 as used in the exome-wide analysis). We also used the average variant rates across these 10,000 permutations instead of the rate in siblings to estimate the variant liability attributed to a gene set in excess of what is expected for matched genes.

### Liability to coexisting developmental difficulties

Co-occurring cognitive and motor difficulties are more prevalent among autistic females, and a direct comparison between autistic individuals with co-occurring motor/cognitive difficulties versus those without coexisting difficulties could help understand whether there is sex-biased liability to having such phenotypes. Here, we used a different-threshold model where these coexisting conditions were the trait, autistic individuals with these conditions were the probands, and autistic individuals without these conditions were the control subjects. We performed these comparisons in SPARK only (i.e., removing those with unknown information) rather than comparing those with cognitive impairment in SPARK and ASC to those without cognitive impairment or with unknown cognitive impairment status, as this grouping would probably underestimate the differences between the two groups (making the interpretation of results difficult). We estimated the sex-stratified liability of having motor or cognitive impairment among autistic individuals assuming prevalences of 0.4 in females and 0.35 in males (the observed prevalences in SPARK trios). To validate the conclusions from this analysis that may be confounded by the use of autistic probands as control subjects (e.g., collider bias), we leveraged published DNMs from 31,565 children ascertained for various neurodevelopmental disorders (NDDs)^14^ and compared the observed counts of damaging protein-truncating and missense DNMs to the expected counts from a mutational model (see supplemental methods section 3.1) in order to estimate the liability attributed to these variants assuming NDDs have population prevalences^24^^,^^25^ of 2% in males and 1.5% in females (the observed sex ratio in this NDD cohort was ∼1.3).

## Results

We examined the burden of autosomal *de novo* and rare inherited variants (minor-allele frequency < 0.1%) exome wide and in specific gene sets in a cohort of 21,501 autistic individuals (13,473 from SPARK and 8,028 from ASC) and 9,223 siblings (6,763 from SPARK and 2,460 from ASC) (see Figure S1 for an outline of all analyses). In these trio-sequenced individuals, the sex-stratified synonymous variant rates (variants per sample) were comparable between the autistic probands and siblings not diagnosed with autism (i.e., rate ratio not significantly different from 1), whereas the rates of *de novo* high-confidence PTVs in highly LoF-intolerant genes (hereafter, damaging PTVs) and missense variants with an MPC score ≥ 2 (damaging missense) were higher in autistic probands. Autistic females showed higher rates of damaging variants than autistic males, particularly those occurring *de novo*, albeit to different degrees in SPARK and ASC (see Table S12 and supplemental results section 1 for details). Over-transmission was most noticeable in damaging PTVs (Figure S6). Additional analyses of ultra-rare variants in 13,435 autistic individuals with sequence data from one parent and 12,125 autistic individuals without parental sequence data also showed an enrichment in damaging PTVs (Figure S7; Table S13).

**Figure 1:**
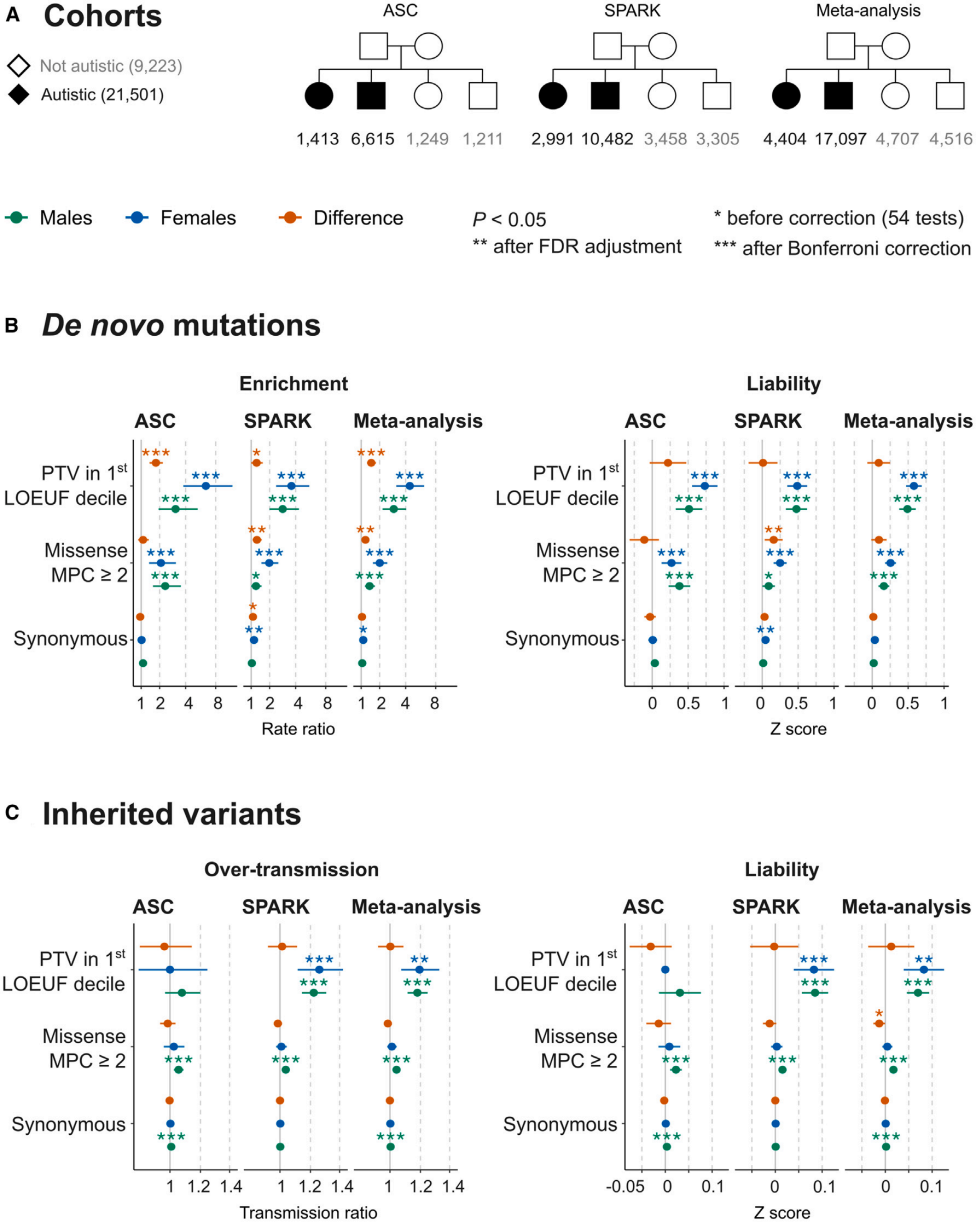
Exome-wide rare variant burden and liability in SPARK and ASC trio-sequenced cohorts (A) The sample size of the trio-sequenced individuals in the Simons Foundation Powering Autism Research for Knowledge (SPARK) study and the Autism Sequencing Consortium (ASC) cohorts. (B) Sex-stratified DNM enrichment and liability. To obtain sex-specific effect sizes on the observed scale, the average DNM rate per trio in autistic males (green dots) or females (blue dots) was divided by the average rate in sex-matched siblings not diagnosed with autism (rate ratio; left). Corresponding effect sizes on the liability scale (*Z* score; right) were measured as explained in Figure S5A and supplemental methods section 6. For sex differences in enrichment (red dots), the observed DNM rate in autistic females was divided by the rate in autistic males (a rate ratio > 1 thus indicates that females show a higher enrichment). The sex difference in variant liability was estimated by subtracting the *Z* scores of the male-only analysis from the female-only *Z* scores (a *Z* score > 0 indicates that females show a higher effect size on the liability scale). (C) Over-transmission and liability of inherited variants. These were assessed using similar comparisons between parental alleles transmitted to autistic individuals and untransmitted alleles (see subjects and methods). Error bars show 95% confidence intervals. See supplemental results sections 2.1.3 and 2.2.3 for further details on synonymous variant imbalances.

Our comparisons of DNMs and inherited variants between sexes (Figure 1; Table S8), which we describe in more detail in supplemental results section 2, recapitulated the known sex-differential patterns of enrichment of damaging PTVs (in the 1^st^ LOEUF decile) and missense variants (MPC ≥ 2) in these cohorts.^1^^,^^2^^,^^12^^,^^14^ Previous work in a subset of the current ASC cohort^2^ showed that the sex bias in DNM rates was strongest when examining PTVs in highly LoF-intolerant genes (defined in that work as a probability of LoF intolerance [pLI] ≥ 0.995) but not significant in less intolerant genes. Similarly, we do not find a significant sex bias on the observed scale when testing other genes in the 2^nd^ and 3^rd^ LOEUF deciles or missense DNMs with lower MPC scores (MPC ≥ 1) (Figure S8; Table S14). However, these comparisons (rate ratios) do not take into account the differences in trait prevalence. Therefore, we next examined whether the sex differences on the observed scale translate into sex differences in liability, which allows comparing effect sizes between groups with different trait prevalences.

### Sex differences in genetic liability conferred by rare variants exome wide

Here, we focus on the meta-analyzed (ASC and SPARK) cohort (Figure 1A) with a total sample size of 4,404 autistic females (versus 4,707 female siblings) and 17,097 autistic males (versus 4,516 male siblings). Despite the relatively higher enrichment (rate ratio) of damaging protein-truncating and missense DNMs in autistic females compared to males (Figure 1B), we did not find statistically significant differences between the male-derived and female-derived liability estimates, neither when testing damaging PTVs in the 1^st^ LOEUF decile and missense variants with MPC scores ≥ 2 (Figure 1) nor when using relaxed filters to include PTVs in the 2^nd^ and 3^rd^ LOEUF deciles and missense variants with MPC scores ≥ 1 (Figure S8).

Specifically, the effect sizes of damaging protein-truncating DNMs (1^st^ LOEUF decile) on the liability scale were not significantly different between autistic females and autistic males (*Z*_sex__-difference_ = 0.90; 95% CI = −0.0684 to 0.25; *p* = 0.27), nor were the effect sizes of damaging missense DNMs (MPC ≥ 2) (*Z*_sex-difference_ = 0.093; 95% CI = −0.014 to 0.20; *p* = 0.087). Similarly, the liability was not significantly different between sexes when comparing DNM rates between autistic probands and all siblings (instead of sex-matched siblings) or sex-discordant siblings (Figure S9; Table S15).

Rare inherited damaging PTVs conveyed significant liabilities in females and males, but these effect sizes were not significantly different between the two sexes (*Z*_sex-difference_ = 0.013; 95% CI = −0.037 to 0.062; *p* = 0.62). Inherited damaging missense variants had higher liability in males compared to females. However, the difference was small and only significant before correcting for 54 multiple tests (*Z*_sex-difference_ = −0.013, 95% CI = −0.00052 to −0.026; *p* = 0.041; FDR-adjusted *p* = 0.096; Bonferroni-corrected *p* = 1). As detailed in supplemental results section 2.2.3, there was a small imbalance in transmitted and untransmitted synonymous alleles in males (*Z*_males_ = 0.0016; 95% CI = 0.0007 to 0.0026), but it is unlikely to affect the main conclusions (i.e., no significant sex differences).

The sex-stratified cohort-level effect sizes in Figure 1 were generally consistent with the meta-analyzed estimates except for inherited damaging PTVs; these conveyed significant liability only in SPARK (Figure 1C). Although the ASC cohort did not show a significant enrichment in inherited damaging PTVs exome wide, it was significantly enriched for inherited damaging PTVs in known autism-predisposition genes (i.e., high-confidence PTVs in 218 highly LoF-intolerant genes among 354 SFARI genes; Figure S11; supplemental results section 2.2). Conversely, removing SFARI genes did not change the overall conclusions regarding the (lack of) sex differences in the average liability of DNM (Figure S10) and rare inherited variants (Figure S11). (See Table S16 for details.)

Further details on cohort-level sex differences in variant rates and liability among trios are presented in supplemental results sections 2.1 and 2.2. We note that synonymous DNMs in autistic females in SPARK showed an association with autism (*Z* = 0.046; 95% CI = 0.01 to 0.082; *p* = 0.011) that did not persist in the meta-analysis (*Z* = 0.036; 95% CI = 0.0055 to 0.067; *p* = 0.052) (see “liability” in Figure 1B). Restricting to ultra-rare alleles in SPARK (Figure S12; Table S17) controlled this spurious signal in synonymous DNMs (see supplemental results section 2.1.4).

An exome-wide analysis in other subsets of the SPARK and ASC cohorts reiterated the findings from the trio analysis. In brief, the average effect size of ultra-rare inherited variants in autistic individuals with sequence data from one parent in SPARK did not differ significantly between sexes after correction for multiple testing (Figure S13; Table S18; supplemental results section 2.3.1). Similarly, there was no significant sex difference in the average liability conferred by ultra-rare variants in the ASC case-control cohorts or in the autistic individuals without parental sequence data in SPARK (Figure S14; Table S19; supplemental results section 2.3.2).

To test whether there are differences between mothers and fathers in the burden, transmission, and liability of ultra-rare parental alleles (seen in one parent and not in gnomAD), we leveraged data from trio-sequenced child-parent pairs in ASC (*n* = 7,570 × 2) and SPARK (*n* = 13,473 × 2), along with other duo-sequenced pairs in SPARK (*n* = 13,435), and evaluated parent-of-origin effects in 55,521 autistic-child nonautistic-parent pairs. Although the mothers of autistic individuals had a significantly higher burden of damaging ultra-rare protein-truncating (rate ratio = 1.15, 95% CI = 1.08 to 1.22, *p* = 2.67 × 10^−6^) and damaging missense ultra-rare (rate ratio = 1.06, 95% CI = 1.03 to 1.09, *p* = 3.0 × 10^−5^) variants compared to the fathers (Figure S15; Table S20), the transmission ratios and the effect sizes of inherited alleles on the liability scale did not differ significantly by the sex of the parent (*p* > 0.05) (Figure S16; Table S21). (See supplemental results section 2.3.3 for details.)

To recapitulate, the average liability conferred by damaging *de novo* protein-truncating and missense mutations as well as inherited PTVs did not show a significant sex difference in a meta-analysis of trio-sequenced individuals from ASC and SPARK. Inherited damaging missense variants conferred higher liability in males, but this difference was very small in magnitude and only significant before accounting for multiple testing. It was also not seen when examining the transmission of ultra-rare variants in a separate set of autistic individuals from SPARK with sequence data from one parent, when examining all trio-/duo-sequenced child-parent pairs together, or in the remaining case-control analyses (ASC case-control cohorts and autistic individuals without parental sequence data in SPARK). Next, we examined whether rare variant liability differs when accounting for co-occurring cognitive difficulties.

### Exome-wide burden in autistic individuals with or without cognitive difficulties

**Figure 2:**
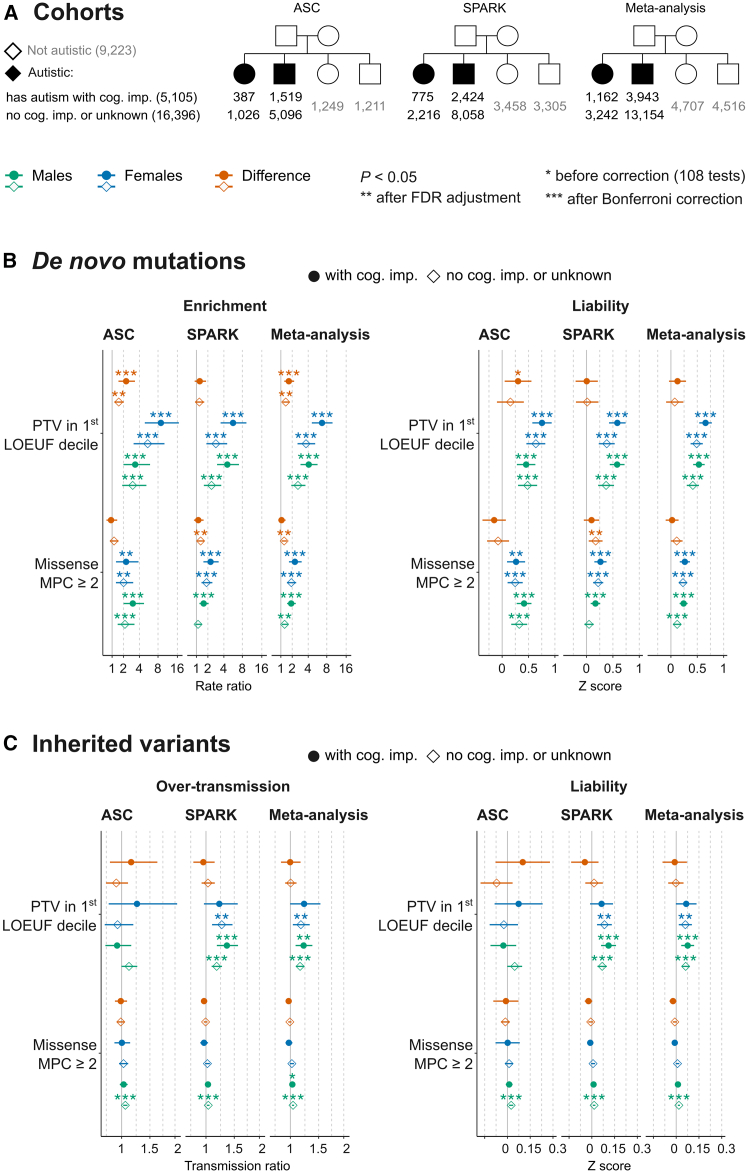
Sex differences in the association between exome-wide burden of damaging *de novo* and rare variants and co-occurring intellectual disability in ASC and SPARK Trio-sequenced individuals from the Autism Sequencing Consortium (ASC) and Simons Foundation Powering Autism Research for Knowledge (SPARK) study cohorts were divided into those who have co-occurring cognitive impairment (cog. imp.; solid dots) and those who do not have cognitive impairment or have missing information (no cog. imp. or unknown; blank diamonds). The sample size of these subgroups is given in (A). In each group, we examined the risk ratio and average liability attributed to damaging DNMs (versus the same group of siblings) (B) and rare inherited variants (transmitted versus untransmitted) (C). Variant burden and liability estimates were meta-analyzed between ASC and SPARK. Sex differences in DNM rate ratios were estimated by direct comparisons of autistic females and males (ratio > 1 means that females have a higher DNM rate), and sex differences in the effect sizes on the liability scale were estimated by subtracting the *Z* scores (score > 0 means that females have a higher effect size).

Both SPARK and ASC cohorts included a mixture of autistic individuals with varying degrees of cognitive difficulties.^1^^,^^14^ There is a higher likelihood of co-occurring cognitive impairment among autistic females compared to autistic males (relative risks of 1.17 in the ASC cohort and 1.12 in SPARK); autistic individuals with cognitive impairment, in turn, have a higher likelihood of harboring high-impact DNMs than those without cognitive impairment.^14^ We hypothesized that the sex differences in damaging DNM rate ratios seen when examining a mix of individuals with and without cognitive difficulties may reflect a difference in the relative frequency of cognitive impairment between the sexes. We thus performed exome-wide comparisons in ASC and SPARK trios stratified by co-occurring cognitive impairment. Specifically, we compared a group of autistic individuals with coexisting cognitive impairment and another group of individuals without cognitive impairment or with an unknown status (since these could not be distinguished using the available ASC data) to the same set of siblings and then meta-analyzed the outcomes between ASC and SPARK (Figure 2A).

In this meta-analysis (Figure 2; Table S9), damaging protein-truncating DNMs showed sex differences on the observed scale among those with cognitive impairment (rate ratio_sex-difference_ = 1.68, 95% CI = 1.28 to 2.20, *p* = 1.8 × 10^−4^, Bonferroni-corrected *p* = 0.02) and, to a lesser extent, those without cognitive difficulties (rate ratio_sex-difference_ = 1.4, 95% CI = 1.14 to 1.71, *p* = 0.0012, FDR-adjusted *p* = 0.0037, Bonferroni-corrected *p* = 0.12). There was no significant sex difference in damaging missense DNM rates between those with cognitive impairment (rate ratio_sex-difference_ = 1.06; 95% CI = 0.80. to 1.39; *p* = 0.69). However, damaging missense DNMs showed significantly higher rates in females than males without cognitive impairment (rate ratio_sex-difference_ = 1.27, 95% CI = 1.06 to 1.51, *p* = 0.0080, FDR-adjusted *p* = 0.021, Bonferroni-corrected *p* = 0.86). On the liability scale, the meta-analyzed effect sizes of damaging protein-truncating and missense DNMs did not differ significantly between the two sexes after stratifying by cognitive impairment (*p* > 0.05) (Figure 2B). Rare inherited variants did not show significant sex differences in over-transmission and liability (Figure 2C). The cohort-level analyses revealed more nuanced patterns, which we discuss in detail in supplemental results section 3.1.

To follow up on the finding that there was no significant exome-wide sex difference in DNM burden when removing SFARI genes in our analysis of all autistic individuals (Figure S10B), we performed a similar analysis (i.e., removing SFARI genes) in those with and without cognitive impairment. For this, we meta-analyzed ASC and SPARK and included additional relaxed variant filters to examine variants with smaller effect sizes. We did not find any significant sex differences in the burden of damaging *de novo* and rare inherited variants, neither on the observed nor the liability scale, reiterating that the observed exome-wide differences in DNM rates are driven primarily by SFARI genes (Figure S17; Table S22).

On the other hand, SFARI genes—when examined separately—had significantly higher damaging DNM rates in females versus males with cognitive impairment (also in those without cognitive difficulties but to a lesser extent), and the sex bias in DNM rates was significantly higher than what is expected from matched genes (Table S23). Still, on the liability scale, the meta-analyzed effect sizes of these DNMs did not differ significantly between the two sexes (Figure S18), nor did the liability conveyed by rare inherited variants in this gene set (Figure S19). Further details are given in supplemental results section 3.2. Moreover, we did not find any significant sex differences in the liability conveyed by *de novo* and rare inherited variants in sex-biased differentially expressed genes in the human fetal cortex (117 female-biased and 305 male-biased genes) or adult cortex (2,427 female-biased and 2,852 male-biased genes) after correction for multiple testing (Figures S20–S27; Tables S24–S27). The average effect sizes of these sex-differentially expressed genes (on both scales) were not significantly higher than what are measured in similarly sized gene sets matched for coding length, LoF constraint, and sex-averaged expression levels (see supplemental results section 3.3).

**Figure 3:**
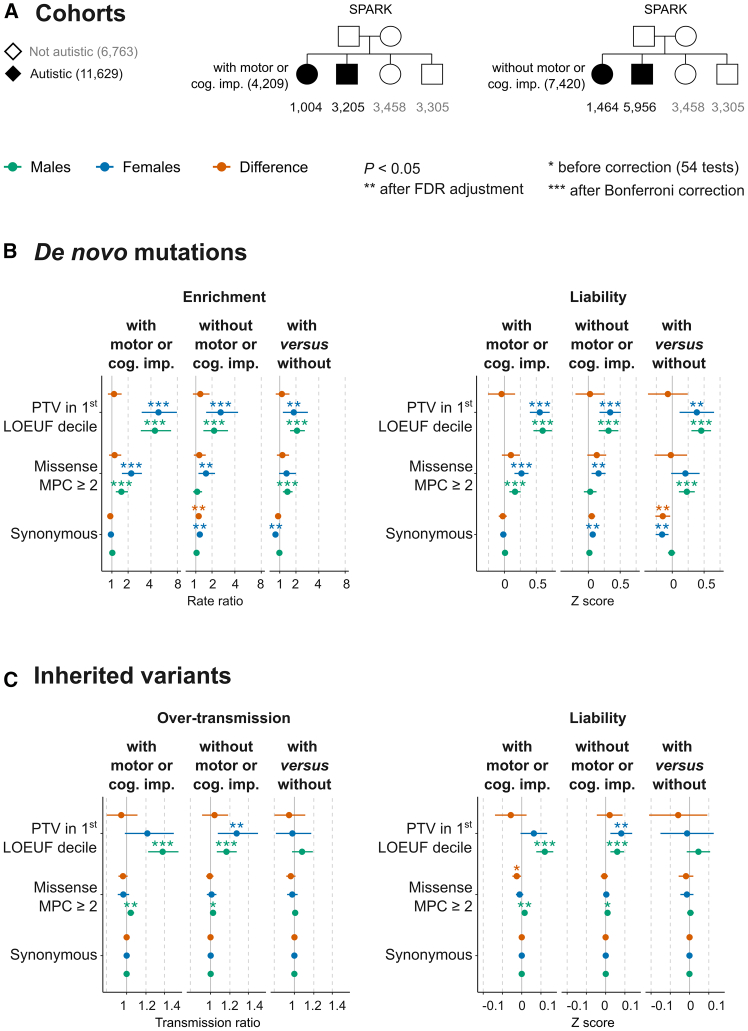
Rare variant burden in autistic individuals with and without cognitive impairment or motor delay in SPARK trio-sequenced cohort (A) The sample size in two sub-cohorts of trio-sequenced individuals from the Simons Foundation Powering Autism Research for Knowledge (SPARK) study divided based on the presence of co-occurring motor developmental delays or cognitive impairment (“motor or cog. imp.”). (B) Sex-stratified observed DNM rates (left) and average liability (right) (see subjects and methods). In addition to the comparisons versus siblings, autistic probands with motor or cognitive difficulties were compared directly to sex-matched autistic individuals without these co-occurring conditions (“with versus without”). (C) Over-transmission of rare inherited variants (left) and their average liability (right) (see subjects and methods). See supplemental results section 4.1.3 for details on the imbalance of synonymous variants. Limiting the analysis in (B) to ultra-rare DNMs in ancestry-matched autistic females and siblings showed well-balanced synonymous DNM burden (*p* = 0.42; Figure S28).

We repeated the exome-wide comparisons in SPARK sub-cohorts stratified by co-occurring cognitive difficulties and motor delay (both versus the same set of siblings not diagnosed with autism in SPARK) while removing those with unknown motor/cognitive impairment status (Figure 3A). These comparisons are detailed in supplemental results section 4.1. In brief, among those with motor/cognitive difficulties (Figure 3; Table S10), we found similar results (i.e., comparable effect sizes on the liability scale) to those seen in probands with cognitive impairment (Figure 2). The results in SFARI genes (Figure S29; Table S29) were also congruent with what we saw when stratifying by cognitive impairment (Figure S18; Table S22) (for details, see supplemental results section 4.2).

Compared to sex-matched siblings, synonymous DNMs were more prevalent in autistic females without motor or cognitive impairment (*Z* = 0.069; 95% CI = 0.028 to 0.11; *p* = 9.9 × 10^−4^) but not in autistic females with these conditions (*Z* = −0.018; 95% CI = −0.068 to 0.033; *p* = 0.50) (see “liability” in Figure 3B). This spurious association was not seen when evaluating ultra-rare DNMs in samples well-matched on genetic ancestry (*Z* = 0.029; 95% CI = −0.041 to 0.099; *p* = 0.42) (see Figure S28, Table S28, and supplemental results section 4.1.3).

In SPARK, there was no significant sex difference in damaging DNM rates (observed scale) after stratification by coexisting cognitive impairment despite the more stringent grouping (removing those with unknown status and considering motor difficulties) (SPARK in Figures 2B and 3B). As this is different from what is seen in the ASC cohort (ASC in Figure 2B), we ran a permutation analysis in SPARK (see supplemental results section 4.2.3 for details), which suggested that the difference in rates of coexisting difficulties between males and females does not drive the sex differences in DNM rates on the observed scale (Figure S30; Table S30). The liability conveyed by damaging rare inherited variants when considering both cognitive and motor difficulties (Figure 3C) was comparable to when we stratified the cohort by cognitive impairment only (Figure 2C). Similarly, there were no significant sex differences in the effect sizes of these variants after accounting for multiple testing. We did not observe significant sex differences in the rate ratios of ultra-rare damaging variants in the remaining autistic individuals in SPARK with one sequenced parent (Figure S31; Table S31) or without sequenced parents (Figure S32; Table S32) after stratifying by cognitive or motor impairment (see supplemental results section 4.3).

In summary, we have seen in a meta-analysis of SPARK and ASC family-based cohorts that the exome-wide effect sizes of damaging *de novo* and rare inherited variants on the liability scale did not differ significantly between autistic males and females—either before or after stratifying by coexisting cognitive difficulties. Sex differences in the genetic burden on the observed scale were most prominent for damaging protein-truncating DNMs and among those with coexisting cognitive impairment. These differences were driven by high-confidence and syndromic-autism-predisposition genes, which had significantly higher effect sizes than similarly sized genes matched for LoF constraint and brain expression.

### Rare variants are insufficient to reach the liability threshold for autism

A key question is whether the genetic predisposition conveyed by damaging *de novo* and rare variants is sufficient by itself to cause autism, which would require crossing a liability threshold of ∼2 units in males and higher in females. For this, we consider here the effect sizes of protein-truncating DNMs in the 1^st^ LOEUF decile (the most impactful class) (Figure 2B). In those with autism and cognitive impairment, the exome-wide average effect sizes were 0.66 in females (95% CI = 0.54 to 0.78) and 0.53 in males (95% CI = 0.42 to 0.64), and the effect sizes in SFARI genes were 1.34 units in females (95% CI = 1.1 to 1.57) and 1.26 in males (95% CI = 1.03 to 1.50). Smaller effect sizes were observed in those without cognitive impairment or with an unknown status (∼0.5 exome wide and ∼1 in autism-predisposition genes). We obtained similar estimates when we examined autistic individuals with or without motor/cognitive difficulties in SPARK (Figures 3B and S29). Thus, in none of these groups of individuals are these rare variants sufficient to cause autism by themselves.

To gain a better understanding of the sex differences in genetic predisposition to cognitive and motor difficulties co-occurring with autism, we compared probands with motor/cognitive impairment to those without these coexisting difficulties in SPARK. In contrast to the former comparisons against non-autistic siblings, which tested the liability conferred to autism *per se,* this analysis measures the extent of the liability to coexisting difficulties in those already diagnosed with autism and whether it differs by sex. Here, we used a different-threshold model where these coexisting difficulties have prevalences of 0.35 among autistic males and 0.40 among females, i.e., females have a lower threshold because there are more females than males with motor/cognitive impairment.

Under this model, the observed 2-fold enrichment of protein-truncating DNMs (in the 1^st^ LOEUF decile) in autistic individuals with versus without motor or cognitive impairment translates to 0.39 units on the liability scale in females (95% CI = 0.11 to 0.66) and 0.45 units in males (95% CI = 0.30 to 0.61) (Figure 3B)—a sufficient amount to reach the threshold to have motor or cognitive impairment in autistic individuals (∼0.25 units in females and ∼0.39 in males). In SFARI genes, the effect sizes were 0.67 in females (95% CI = 0.30 to 1.03) and 0.57 in males (95% CI = 0.35 to 0.80) (Figure S29). The sex difference was not significant, either exome wide (*p* = 0.66) or in SFARI genes (*p* = 0.67). Seeking further validation, we estimated the liability conferred by damaging DNMs in SFARI genes in 31,565 trios diagnosed with NDDs not specifically ascertained for autism^14^ (see supplemental results section 4.4). The effect sizes were indeed sufficient to reach the liability threshold for NDDs and not significantly different between the sexes in this independent cohort (Figure S33; Table S33).

To sum up, damaging protein-truncating DNMs in SFARI autism-predisposition genes confer similar liabilities in both sexes that are enough to reach the threshold for having neurodevelopmental conditions in general or coexisting motor and cognitive difficulties in autistic individuals in particular. However, they do not confer enough liability to reach the threshold for autism *per se* in either sex, regardless of whether these individuals have coexisting motor or cognitive difficulties or not.

## Discussion

We examined the sex differences in rare autosomal coding variant rates both exome wide and in specific gene sets in 47,061 autistic individuals from two large autism cohorts,^1^^,^^14^ showing that the average liability attributed to damaging rare variants exome wide and in genes with sex-biased expression in the cortex is not statistically significantly different between males and females.

The sex differences in DNM rates on the observed scale did not translate into differences in variant liability between sexes (Figure 1B) and were driven by known autism-predisposition genes (Figure S10), which also increase the chance of other developmental disorders affecting motor and cognitive skills (Figure S33). We did not find evidence that the sex differences in damaging DNM rate ratios seen when examining a mix of individuals with and without motor and cognitive difficulties reflect a difference in the relative frequency of these endo-phenotypes between the sexes. Autistic individuals in the ASC cohort still showed enrichment of DNMs in females versus males even when restricting to those with cognitive impairment (Figure 2B). Although we did not see a significant sex difference in the rates of damaging protein-truncating DNMs in SPARK when autistic individuals were stratified by coexisting cognitive impairment (Figure 2B) or motor/cognitive difficulties (Figure 3B), a permutation analysis suggested that co-occurring difficulties—alone—cannot account for the sex differences on the observed scale (Figure S30). Thus, it seems likely that the difference in damaging DNM rates between the sexes is, instead, a reflection of the different thresholds under the liability threshold model, which we discuss further below.

SFARI high-confidence and syndromic-autism-predisposition genes drove the exome-wide female bias in DNM burden on the observed scale (Figure S10), showing substantially higher DNM rates compared to matched genes with comparable constraint and brain expression (Figure S18). This is consistent with our recent observation (in a smaller subset of SPARK and Simons Simplex Collection) that the female excess in damaging DNMs is explained by a small set of developmental genes and is not accounted for fully by co-occurring difficulties.^26^ The effect sizes of damaging variants in these genes on the liability scale were also higher than those estimated for matched genes but did not differ significantly by sex (Figure S18). On the other hand, the liability attributed to damaging *de novo* and rare variants in a small set of sex-differentially expressed genes in the fetal cortex^22^ or a larger set of genes with sex-biased expressed in the adult cortex^23^ did not differ significantly from what is expected from matched genes (Figures S20–S27). Among these differentially expressed genes, the strongest enrichment and female bias on the observed scale (rate ratios) was seen in protein-truncating DNMs in genes showing male-biased expression in the adult cortex, but this was attributable to their overlap with LoF-intolerant genes and genes with high expression in brain (Figure S20). This is in line with previous findings that long highly brain-expressed genes overlap significantly with autism-predisposition genes and explain their over-representation among *Fragile X messenger ribonucleoprotein 1*-binding targets^27^ (*FMR1* [MIM: 309550]). The enrichment of sex-differentially expressed genes in damaging DNMs is likely a reflection of their enrichment in neuronal genes, which has been previously noted.^23^ The findings of these analyses of differentially expressed genes should be interpreted cautiously, as there are several caveats, which we discuss briefly in supplemental results section 3.3.4.

Protein-disrupting alterations confer the highest predisposition for autism among rare short coding variants.^1^^,^^12^^,^^14^ Since genes implicated through *de novo* association are generally developmental disorder genes (e.g., SFARI genes), it is unclear whether they increase the predisposition for autism *per se*. Under a different-threshold liability threshold model, in which autism predisposition is assumed to be additive and normally distributed (Figure S5), an autism prevalence of 2.5% among males in the general population puts the threshold for autism diagnosis at 1.96 standardized units (2.5 units in females assuming ∼4:1 ratio). (See Figure S5 and supplemental methods section 6.1.) We estimate that the liability conveyed by protein-truncating DNMs (in highly LoF-constrained genes) alone is insufficient to reach the threshold for autism diagnosis in the absence of other factors such as, for example, a high polygenic score for autism. This was true for the liabilities estimated in those with autism and motor/cognitive difficulties or without these coexisting conditions in SPARK (Figure 3), when meta-analyzing SPARK and ASC stratified by cognitive impairment (Figure 2), and when considering protein-truncating DNMs in SFARI genes in all these groups (Figures S18 and S29).

On the other hand, the analysis we carried out in SPARK to estimate the liability to co-occurring motor and cognitive difficulties suggests that damaging protein-truncating DNMs have an average effect size that conveys enough risk to cause these co-occurring difficulties when they are seen in approximately one-third of the SPARK cohort of autistic children—a proportion similar to that of the whole population of autistic individuals.^4^ Protein-truncating DNMs in SFARI genes conferred enough predisposition to cognitive and motor difficulties among autistic individuals in SPARK so that an autistic individual harboring such a variant will have exceeded the liability threshold for these co-occurring difficulties (Figure S29), reflecting how cognitive or motor difficulties are key phenotypic presentations associated with harboring damaging variants in these genes. This was corroborated by observations in an independent cohort of trios ascertained for various NDDs rather than autism specifically—where these genes conferred genetic predisposition on par with genes curated for their association with developmental disorders (DDG2P genes^28^) (Figure S33). Thus, our results suggest that damaging DNMs are insufficient by themselves to cause autism, but they can cause cognitive or motor impairment (NDDs). This fits with the observation that most autism-predisposition genes are known to cause motor disorders or intellectual disability with high penetrance (most are DDG2P genes), but the penetrance of autism in most of these genes is often low to moderate.^4^^,^^29^ Hence, autism is likely an incompletely penetrant, complex phenotype within the phenotypic spectrum of these developmental genes.

The difference in the proportion of autistic females versus autistic males with cognitive impairment could be explained by the presence of genetic risk factors with similar effect sizes in the two sexes that are highly penetrant for cognitive impairment but are more frequent in autistic females than autistic males due to the different thresholds for autism. In particular, as noted above, protein-truncating DNMs in highly LoF-intolerant genes or SFARI genes have—on average—moderate penetrance for autism but high penetrance for cognitive impairment. The liability threshold for diagnosing cognitive impairment is relatively similar between the sexes, as suggested by the low sex bias in neurodevelopmental conditions featuring profound cognitive difficulties.^24^^,^^25^^,^^30^^,^^31^; For instance, there are 1.6 males for every female in the Deciphering Developmental Disorders study.^32^ Females reaching the (high) threshold on the liability scale for autism diagnosis are more likely than both non-autistic females and autistic males to harbor damaging protein-truncating DNMs (evidenced by the higher risk ratio for carrying these DNMs on the observed scale; Figure 1B), and in turn, more autistic females than autistic males are likely to have reached the threshold for cognitive impairment as well (given the high penetrance of these DNMs for cognitive impairment in both sexes). It is plausible that the sex difference in the rates of autism with coexisting cognitive impairment may partly reflect biased diagnostic sensitivities, e.g., because females typically show autistic traits that are less likely (than autistic traits seen typically in males) to prompt clinical evaluation if they have otherwise typical cognitive development.^33^ However, epidemiological data suggest that the higher proportion of autistic females with cognitive impairment (relative to autistic males) is more likely a true property of the autistic spectrum,^34^ and this was observed to various degrees in several autism cohorts with different ascertainment strategies, e.g., in SPARK and among most of the ASC sites (Figure S4).

Our analysis relied on a standard liability threshold model assuming equal variance of the liability distribution in males and females.^4^^,^^10^ This model is easily interpretable and allows direct comparisons between the two sexes on the same scale. Under this different-threshold model, females require a higher load of additive risk elements to reach their higher threshold; the equal effect sizes on the liability scale, and the sex-differential rates of highly penetrant variants, are consistent with the assumptions of the different-threshold model. However, the liability conveyed by rare variants alone is not sufficient to reach the threshold for autism diagnosis in the absence of other factors, even when lower thresholds in females (sex ratios of 3:1 and 2:1) are considered (Figure S34; Table S34). The genetic predisposition from rare variants could be complemented by other factors, e.g., common variants. It has been postulated that common variants may drive most of the genetic predisposition for autism,^35^ although this varies substantially between cohorts and methodologies. Common variants, as measured by polygenic indices, may play a larger role among phenotypic groups that show relatively more male bias; we have previously shown that the association between an autism diagnosis and an autism polygenic score capturing a proportion of the predisposition from common variants is more pronounced in autistic individuals with few motor and cognitive developmental difficulties than in those with several developmental disabilities.^26^ Moreover, we previously saw that the sex difference in polygenic over-transmission (i.e., higher deviation from mid-parental polygenic scores in females) was evident only when examining autistic individuals without cognitive impairment,^26^^,^^36^ the group with the more pronounced difference in liability thresholds. Notably, we find significantly higher rates of damaging ultra-rare variants in the mothers compared to the fathers of autistic individuals (Figure S15), similar to what was shown for common inherited alleles.^36^ However, we do not see significant differences in the effect sizes of these alleles when transmitted to their children (Figure S16).

Although our results are consistent with the different-threshold liability threshold model, alternative models with less restrictive assumptions, e.g., higher variance in males,^4^^,^^37^ may better capture the true underlying distribution of autism predisposition in the population. While sex differences in autism prevalence may result from combined differences in the mean and variance of the liability (Figure S5B), the observations made in a population-based analysis in ∼1 million Swedish individuals best fitted a model with higher variance in males—and also suggested that autism heritability may be lower in females than males.^38^ Curiously, the effect sizes of rare inherited damaging variants on the liability scale were significantly higher in males when we tested a model that assumes that the variance in males is ∼2–3 times higher than in females (Figure S35; Table S35), although DNMs did not show any significant difference in their effect sizes. We take from this that the effect sizes of damaging DNMs are indeed similar between males and females, whereas better modeling of liability may uncover more nuanced differences in the liability attributed to inherited variants.

With all models, the contribution of any sex differences in the effect size of rare and common variants—or lack thereof—to the sex-biased prevalence of autism should be interpreted in light of the small phenotypic variance they explain.^39^ The high familial and twin heritability of autism^38^^,^^40^^,^^41^ (∼70%–90%) suggests a major genetic contribution. Rare protein-coding variants, variants in conserved non-coding regions, copy-number alterations, and tandem repeats explain ∼10% of the variance.^1^^,^^12^^,^^42^^,^^43^^,^^44^ Additive SNP heritability from common variants as estimated from a recent autism genome-wide association study (GWAS)^45^ is also ∼10%. Finding where the missing heritability lies (e.g., with more powerful association studies across the spectrum of allele frequencies and coding/non-coding variation types) is therefore essential to develop reliable proxies for genetic liability and model sex differences properly. Furthermore, it is also essential to understand non-additive effects and the role of non-genetic predisposition like prenatal exposures (sex hormones), postnatal (social) environment, sex differences in autism presentations, and biases in assessment tools, referral, and diagnosis leading to under-/mis-diagnosis in females.^33^^,^^39^^,^^46^^,^^47^^,^^48^ Further insights may be gleaned from studying quantitative autistic traits in population samples.^49^^,^^50^

A strength of this study is that we included samples from diverse populations. In theory, since our core analyses were focused on within-family analyses of DNMs and inherited variants, the inclusion of individuals of different ancestries should not create spurious stratification effects. However, in practice, this resulted in a subtle association of synonymous DNMs with autism diagnosis in SPARK (Figure 1A), discussed in supplemental results sections 2.1.3 and 4.1.3. The imbalance in synonymous DNMs was most prominent between autistic females without motor delay or cognitive impairment and sex-matched siblings not diagnosed with autism (Figure 3B). It is unlikely that this subtle imbalance biased the outcomes for damaging protein-truncating DNMs. Another limitation of our study is that we only examined rare variants on the autosomes, ignoring the sex chromosomes. We note that recent large-scale gene association studies from the ASC did not include sex chromosomes,^1^^,^^2^ so the contribution of sex-linked genes may be underestimated. Nonetheless, it seems unlikely that large-effect rare variants on the sex chromosomes are a major driver of the sex difference in autism,^17^ at least among those with co-occurring motor and cognitive impairments, since our previous work in the Deciphering Developmental Disorders cohort found that rare Mendelian-acting coding variants in the X chromosome contributed similarly in males and females and did not explain the observed 1.6:1 male bias.^32^

To summarize, deleterious *de novo* and rare inherited autosomal coding variants confer similar liability for autism in females and males under a different-threshold model. These variants, particularly *de novo* protein-truncating mutations, increase the liability for co-occurring motor or cognitive impairment significantly more than autism with otherwise typical motor and cognitive development. Autosomal DNMs with large effect sizes are therefore unlikely to explain the observed sex differences in autism prevalence. Future studies with larger sample sizes, considering the contribution of both autosomal and sex-linked alleles across the frequency spectrum of rare and common variants, may capture additional predisposing variants with small effect sizes that contribute to the sex differences in autism.

## Data and code availability

This study did not generate new exome or phenotype datasets. The supplemental information contains Figures S1–S35, supplemental results, and supplemental methods. The code used for the main analyses is included in the supplemental methods. Tables S1–S35 (including the data plotted in the figures) are available in a separate supplemental file. SPARK phenotypes and exome data are available for approved users through SFARI Base (https://www.sfari.org/resource/sfari-base/). The ASC data used in this study are available for approved users at NHGRI AnVIL (https://anvilproject.org/data) with the accession ID: phs000298.

## Consortia

The members of the Autism Sequencing Consortium are Branko Aleksic, Mykyta Artomov, Mafalda Barbosa, Elisa Benetti, Catalina Betancur, Monica Biscaldi-Schafer, Anders D. Børglum, Harrison Brand, Alfredo Brusco, Joseph D. Buxbaum, Gabriele Campos, Simona Cardaropoli, Diana Carli, Angel Carracedo, Marcus C.Y. Chan, Andreas G. Chiocchetti, Brian H.Y. Chung, Brett Collins, Ryan L. Collins, Edwin H. Cook, Hilary Coon, Claudia I.S. Costa, Michael L. Cuccaro, David J. Cutler, Mark J. Daly, Silvia De Rubeis, Bernie Devlin, Ryan N. Doan, Enrico Domenici, Shan Dong, Chiara Fallerini, Montserrat Fernández-Prieto, Giovanni Battista Ferrero, Christine M. Freitag, Jack M. Fu, J. Jay Gargus, Sherif Gerges, Elisa Giorgio, Ana Cristina Girardi, Stephen Guter, Emily Hansen-Kiss, Gail E. Herman, Irva Hertz-Picciotto, David M. Hougaard, Christina M. Hultman, Suma Jacob, Miia Kaartinen, Lambertus Klei, Alexander Kolevzon, Itaru Kushima, So Lun Lee, Terho Lehtimäki, Lindsay Liang, Carla Lintas, Alicia Ljungdahl, Caterina Lo Rizzo, Yunin Ludena, Patricia Maciel, Behrang Mahjani, Nell Maltman, Marianna Manara, Dara S. Manoach, Gal Meiri, Idan Menashe, Judith Miller, Nancy Minshew, Matthew Mosconi, Rachel Nguyen, Norio Ozaki, Aarno Palotie, Mara Parellada, Maria Rita Passos-Bueno, Lisa Pavinato, Minshi Peng, Margaret Pericak-Vance, Antonio M. Persico, Isaac N. Pessah, Kaija Puura, Abraham Reichenberg, Alessandra Renieri, Kathryn Roeder, Stephan J. Sanders, Sven Sandin, F. Kyle Satterstrom, Stephen W. Scherer, Sabine Schlitt, Rebecca J. Schmidt, Lauren Schmitt, Katja Schneider-Momm, Paige M. Siper, Laura Sloofman, Moyra Smith, Christine R. Stevens, Pål Suren, James S. Sutcliffe, John A. Sweeney, Michael E. Talkowski, Flora Tassone, Karoline Teufel, Elisabetta Trabetti, Slavica Trajkova, Maria del Pilar Trelles, Brie Wamsley, Jaqueline Y.T. Wang, Lauren A. Weiss, Mullin H.C. Yu, and Ryan Yuen.

The members of the APEX Consortium are Deep Adhya, Carrie Allison, Bonnie Ayeung, Rosie Bamford, Simon Baron-Cohen, Richard Bethlehem, Tal Biron-Shental, Graham Burton, Wendy Cowell, Jonathan Davies, Dori Floris, Alice Franklin, Lidia Gabis, Daniel Geschwind, David M. Greenberg, Yuanjun Gu, Alexandra Havdahl, Alexander Heazell, Rosemary Holt, Matthew Hurles, Yumnah Khan, Meng-Chuan Lai, Madeline Lancaster, Michael Lombardo, Hilary Martin, Jose Gonzalez Martinez, Jonathan Mill, Mahmoud Koko Musa, Kathy Niakan, Adam Pavlinek, Lucia Dutan Polit, Marcin Radecki, David Rowitch, Laura Sichlinger, Deepak Srivastava, Alexandros Tsompanidis, Florina Uzefovsky, Varun Warrier, Elizabeth Weir, Xinhe Zhang.

## Acknowledgments

We thank the participants and investigators who contributed to the datasets of the Simons Foundation Powering Autism Research for Knowledge (SPARK) project, the Autism Sequencing Consortium (ASC), the Simons Simplex Collection (SSC), and the Lundbeck Foundation Initiative for Integrative Psychiatric Research (iPSYCH) project. This work was supported by the 10.13039/100014370Simons Foundation Autism Research Initiative (SFARI) through grant RNAG/669 G10 9280 to H.M., V.W., and other principal investigators of the Autism Prenatal Sex Differences Consortium (APEX). The ASC received support from 10.13039/100014370SFARI (574598, 736613, and 647371 to S.J.S.; 575097 to B.D. and K.R.; 573206 to M.E.T. and M.J.D.; 571009 to J.D.; and 606362 and 608540 to M.E.T., M.J.D., J.D.B., B.D., K.R., and S.J.S., all from the consortia author list), 10.13039/100000051NHGRI (HG008895 to M.J.D., S.G., and M.E.T. from the consortia author list), 10.13039/100000025NIMH (MH115957 and MH123155 to M.E.T.; MH111658 and MH057881 to B.D.; MH097849, MH111661, and MH100233 to J.D.B.; MH109900 and MH123184 to K.R.; MH111660 and MH129722 to M.J.D.; and MH111662 and MH100027 to S.J.S., all from the consortia author list), NICHD (HD081256 and HD096326 to M.E.T. from the consortia author list), 10.13039/100009619AMED (JP21WM0425007 to N.O. from the consortia author list), and the Beatrice and Samuel Seaver Foundation. This research was funded in whole, or in part, by the Wellcome Trust (grant number 220540/Z/20/A). For the purpose of Open Access, the authors have applied a CC-BY public copyright licence to any Author Accepted Manuscript version from this submission.

## Author contributions

Study design, H.M., V.W., M.K., and the APEX Consortium; samples and data generation, the Autism Sequencing Consortium; quality control and data preparation, M.K. and F.K.S.; analysis, M.K.; writing – draft, M.K. and H.M.; writing – editing and critical revisions, H.M., M.K., F.K.S., and V.W.; study direction and supervision, H.M. and V.W.

## Declaration of interests

The authors declare no competing interests.

## Web resources

OMIM, http://www.omim.org
NHGRI AnVIL, https://anvilproject.org/data
SFARI Base, https://www.sfari.org/resource/sfari-base/

## Supplemental information

Document S1. Figures S1–S35, supplemental results, and supplemental methods

Document S2. Tables S1–S35

Document S3. Article plus supplemental information
