# Supplementary material for: Contribution of autosomal rare and de novo variants to sex differences in autism
Source: Am J Hum Genet. 2025 Feb 14;112(3):599–614. doi: 10.1016/j.ajhg.2025.01.016 (PMC11947420; doi:10.1016/j.ajhg.2025.01.016)
Supplement: Document S1. Figures S1–S35, supplemental results, and supplemental methods [file mmc1.pdf]

The American Journal of Human Genetics, Volume *112*

## Supplemental information

# Contribution of autosomal rare and *de novo* variants to sex differences in autism

**Mahmoud Koko, F. Kyle Satterstrom, Autism Sequencing Consortium, APEX consortium, Varun Warrier, and Hilary Martin**

# Consortia members and affiliations

## Autism Sequencing Consortium (ASC)

- Branko Alexie: Department of Psychiatry, Graduate School of Medicine, Nagoya University, Nagoya, Japan
- Mykyta Artomov: Center for Genomic Medicine, Massachusetts General Hospital, Boston, MA, USA; Program in Medical and Population Genetics, Broad Institute of MIT and Harvard, Cambridge, MA, USA; Stanley Center for Psychiatric Research, Broad Institute of MIT and Harvard, Cambridge, MA, USA; Harvard Medical School, Boston, MA, USA
- Mafalda Barbosa: The Mindich Child Health and Development Institute, Icahn School of Medicine at Mount Sinai, New York, NY, USA; Department of Genetics and Genomic Sciences, Icahn School of Medicine at Mount Sinai, New York, NY, USA
- Elisa Benetti: Med Biotech Hub and Competence Center, Department of Medical Biotechnologies, University of Siena, Siena, Italy; Medical Genetics, , University of Siena, Siena, Italy
- Catalina Betancur: Sorbonne Université, INSERM, CNRS, Neuroscience Paris Seine, Institut de Biologie Paris Seine, Paris, France
- Monica Biscaldi-Schafer: Department of Child and Adolescent Psychiatry, Psychosomatics and Psychotherapy, Goethe University Frankfurt, Frankfurt, Germany
- Anders D. Børglum: The Lundbeck Foundation Initiative for Integrative Psychiatric Research, iPSYCH, Aarhus, Denmark; Department of Biomedicine—Human Genetics, Aarhus University, Aarhus, Denmark; Center for Genomics and Personalized Medicine, Aarhus, Denmark; Bioinformatics Research Centre, Aarhus University, Aarhus, Denmark
- Harrison Brand: Center for Genomic Medicine, Massachusetts General Hospital, Boston, MA, USA; Program in Medical and Population Genetics, Broad Institute of MIT and Harvard, Cambridge, MA, USA; Department of Neurology, Massachusetts General Hospital and Harvard Medical School, Boston, MA, USA; Pediatric Surgical Research Laboratories, Department of Surgery, Massachusetts General Hospital, Boston, MA, USA
- Alfredo Brusco: Department of Medical Sciences, University of Torino, Turin, Italy; Medical Genetics Unit, 'Città della Salute e della Scienza' University Hospital, Turin, Italy
- Joseph D. Buxbaum: Seaver Autism Center for Research and Treatment, Icahn School of Medicine at Mount Sinai, New York, NY, USA; Department of Psychiatry, Icahn School of Medicine at Mount Sinai, New York, NY, USA; The Mindich Child Health and Development Institute, Icahn School of Medicine at Mount Sinai, New York, NY, USA; Department of

Genetics and Genomic Sciences, Icahn School of Medicine at Mount Sinai, New York, NY, USA; Friedman Brain Institute, Icahn School of Medicine at Mount Sinai, New York, NY, USA; Department of Neuroscience, Icahn School of Medicine at Mount Sinai, New York, NY, USA

- Gabriele Campos: Centro de Pesquisas sobre o Genoma Humano e Células tronco, Instituto de Biociências, Universidade de São Paulo, São Paulo, Brazil
- Simona Cardaropoli: Department of Public Health and Pediatrics, University of Torino, Turin, Italy
- Diana Carli: Department of Public Health and Pediatrics, University of Torino, Turin, Italy
- Angel Carracedo: Grupo de Medicina Xenómica, Centro de Investigación en Red de Enfermedades Raras (CIBERER), CIMUS, Universidade de Santiago de Compostela, Santiago de Compostela, Spain; Fundación Pública Galega de Medicina Xenómica, Servicio Galego de Saúde (SERGAS), Santiago de Compostela, Spain
- Marcus C. Y. Chan: Department of Pediatrics and Adolescent Medicine, Duchess of Kent Children’s Hospital, The University of Hong Kong, Hong Kong Special Administrative Region, China
- Andreas G. Chiocchetti: Department of Psychiatry, Graduate School of Medicine, Nagoya University, Nagoya, Japan
- Brian H. Y. Chung: Department of Pediatrics and Adolescent Medicine, Duchess of Kent Children’s Hospital, The University of Hong Kong, Hong Kong Special Administrative Region, China
- Brett Collins: Seaver Autism Center for Research and Treatment, Icahn School of Medicine at Mount Sinai, New York, NY, USA; Department of Psychiatry, Icahn School of Medicine at Mount Sinai, New York, NY, USA; The Mindich Child Health and Development Institute, Icahn School of Medicine at Mount Sinai, New York, NY, USA
- Ryan L. Collins: Center for Genomic Medicine, Massachusetts General Hospital, Boston, MA, USA; Program in Medical and Population Genetics, Broad Institute of MIT and Harvard, Cambridge, MA, USA; Department of Neurology, Massachusetts General Hospital and Harvard Medical School, Boston, MA, USA; Program in Bioinformatics and Integrative Genomics, Harvard Medical School, Boston, MA, USA
- Edwin H. Cook: Institute for Juvenile Research, Department of Psychiatry, University of Illinois at Chicago, Chicago, IL, USA
- Hilary Coon: Department of Internal Medicine, University of Utah, Salt Lake City, UT, USA; Department of Psychiatry, Huntsman Mental Health Institute, University of Utah, Salt Lake City, UT, USA
- Claudia I. S. Costa: Centro de Pesquisas sobre o Genoma Humano e Células tronco, Instituto de Biociências, Universidade de São Paulo, São Paulo, Brazil

- Michael L. Cuccaro: The John P Hussman Institute for Human Genomics, The University of Miami Miller School of Medicine, Miami, FL, USA
- David J. Cutler: Department of Human Genetics, Emory University School of Medicine, Atlanta, GA, USA
- Mark J. Daly: Center for Genomic Medicine, Massachusetts General Hospital, Boston, MA, USA; Program in Medical and Population Genetics, Broad Institute of MIT and Harvard, Cambridge, MA, USA; Stanley Center for Psychiatric Research, Broad Institute of MIT and Harvard, Cambridge, MA, USA; Analytic and Translational Genetics Unit, Department of Medicine, Massachusetts General Hospital, Boston, MA, USA; Harvard Medical School, Boston, MA, USA; Institute for Molecular Medicine Finland (FIMM), University of Helsinki, Helsinki, Finland
- Silvia De Rubeis: Seaver Autism Center for Research and Treatment, Icahn School of Medicine at Mount Sinai, New York, NY, USA; Department of Psychiatry, Icahn School of Medicine at Mount Sinai, New York, NY, USA; The Mindich Child Health and Development Institute, Icahn School of Medicine at Mount Sinai, New York, NY, USA; Friedman Brain Institute, Icahn School of Medicine at Mount Sinai, New York, NY, USA
- Bernie Devlin: Department of Psychiatry, University of Pittsburgh School of Medicine, Pittsburgh, PA, USA
- Ryan N. Doan: Division of Genetics and Genomics, Boston Children’s Hospital, Boston, MA, USA
- Enrico Domenici: Department of Cellular, Computational and Integrative Biology, , University of Trento, Trento, Italy
- Shan Dong: Department of Psychiatry, UCSF Weill Institute for Neurosciences, University of California San Francisco, San Francisco, CA, USA
- Chiara Fallerini: Med Biotech Hub and Competence Center, Department of Medical Biotechnologies, University of Siena, Siena, Italy; Medical Genetics, , University of Siena, Siena, Italy
- Montserrat Fernández-Prieto: Grupo de Medicina Xenómica, Centro de Investigación en Red de Enfermedades Raras (CIBERER), CIMUS, Universidade de Santiago de Compostela, Santiago de Compostela, Spain; Neurogenetics group, Instituto de Investigación Sanitaria de Santiago (IDIS-SERGAS), Santiago de Compostela, Spain
- Giovanni Battista Ferrero: Department of Public Health and Pediatrics, University of Torino, Turin, Italy
- Christine M. Freitag: Department of Psychiatry, Graduate School of Medicine, Nagoya University, Nagoya, Japan
- Jack M. Fu: Center for Genomic Medicine, Massachusetts General Hospital, Boston, MA, USA; Program in Medical and Population Genetics, Broad Institute of MIT and Harvard,

Cambridge, MA, USA; Department of Neurology, Massachusetts General Hospital and Harvard Medical School, Boston, MA, USA

- J. Jay Gargus: Center for Autism Research and Translation, University of California Irvine, Irvine, CA, USA
- Sherif Gerges: Center for Genomic Medicine, Massachusetts General Hospital, Boston, MA, USA; Program in Medical and Population Genetics, Broad Institute of MIT and Harvard, Cambridge, MA, USA; Stanley Center for Psychiatric Research, Broad Institute of MIT and Harvard, Cambridge, MA, USA; Harvard Medical School, Boston, MA, USA
- Elisa Giorgio: Department of Medical Sciences, University of Torino, Turin, Italy
- Ana Cristina Girardi: Centro de Pesquisas sobre o Genoma Humano e Células tronco, Instituto de Biociências, Universidade de São Paulo, São Paulo, Brazil
- Stephen Guter: Institute for Juvenile Research, Department of Psychiatry, University of Illinois at Chicago, Chicago, IL, USA
- Emily Hansen-Kiss: Department of Diagnostic and Biomedical Sciences, University of Texas Health Science Center at Houston, School of Dentistry, Houston, TX, USA
- Gail E. Herman: The Research Institute at Nationwide Children's Hospital, Columbus, OH, USA
- Irva Hertz-Picciotto: MIND (Medical Investigation of Neurodevelopmental Disorders) Institute, University of California Davis, Davis, CA, USA
- David M. Hougaard: Department of Child and Adolescent Psychiatry, Psychosomatics and Psychotherapy, Goethe University Frankfurt, Frankfurt, Germany; Center for Neonatal Screening, Department for Congenital Disorders, Statens Serum Institut, Copenhagen, Denmark
- Christina M. Hultman: Department of Medical Epidemiology and Biostatistics, Karolinska Institutet, Stockholm, Sweden
- Suma Jacob: Institute for Juvenile Research, Department of Psychiatry, University of Illinois at Chicago, Chicago, IL, USA
- Miia Kaartinen: Department of Child Psychiatry, Tampere University and Tampere University Hospital, Tampere, Finland
- Lambertus Klei: Department of Psychiatry, University of Pittsburgh School of Medicine, Pittsburgh, PA, USA
- Alexander Kolevzon: Seaver Autism Center for Research and Treatment, Icahn School of Medicine at Mount Sinai, New York, NY, USA; Department of Psychiatry, Icahn School of Medicine at Mount Sinai, New York, NY, USA; Department of Pediatrics, Icahn School of Medicine at Mount Sinai, New York, NY, USA

- Itaru Kushima: The Lundbeck Foundation Initiative for Integrative Psychiatric Research, iPSYCH, Aarhus, Denmark; Medical Genomics Center, Nagoya University Hospital, Nagoya, Japan
- So Lun Lee: Department of Pediatrics and Adolescent Medicine, Duchess of Kent Children's Hospital, The University of Hong Kong, Hong Kong Special Administrative Region, China
- Terho Lehtimäki: Department of Clinical Chemistry, Fimlab Laboratories and Finnish Cardiovascular Research Center-Tampere, Faculty of Medicine and Health Technology, Tampere University, Tampere, Finland
- Lindsay Liang: Department of Psychiatry, UCSF Weill Institute for Neurosciences, University of California San Francisco, San Francisco, CA, USA
- Carla Lintas: Service for Neurodevelopmental Disorders, University Campus Bio-medico of Rome, Rome, Italy
- Alicia Ljungdahl: Department of Psychiatry, UCSF Weill Institute for Neurosciences, University of California San Francisco, San Francisco, CA, USA
- Caterina Lo Rizzo: Medical Genetics, , University of Siena, Siena, Italy; Genetica Medica, Azienda Ospedaliera Universitaria Senese, Siena, Italy
- Yunin Ludena: MIND (Medical Investigation of Neurodevelopmental Disorders) Institute, University of California Davis, Davis, CA, USA
- Patricia Maciel: Life and Health Sciences Research Institute, School of Medicine, University of Minho, Braga, Portugal
- Behrang Mahjani: Seaver Autism Center for Research and Treatment, Icahn School of Medicine at Mount Sinai, New York, NY, USA; Department of Psychiatry, Icahn School of Medicine at Mount Sinai, New York, NY, USA; Department of Medical Epidemiology and Biostatistics, Karolinska Institutet, Stockholm, Sweden
- Nell Maltman: Institute for Juvenile Research, Department of Psychiatry, University of Illinois at Chicago, Chicago, IL, USA
- Marianna Manara: Medical Genetics, , University of Siena, Siena, Italy; Genetica Medica, Azienda Ospedaliera Universitaria Senese, Siena, Italy
- Dara S. Manoach: Department of Psychiatry, Massachusetts General Hospital and Harvard Medical School, Boston, MA, USA
- Gal Meiri: The Azrieli National Center for Autism and Neurodevelopment Research, Ben-Gurion University of the Negev, Beer-Sheva, Israel; Pre-School Psychiatry Unit, Soroka University Medical Center, Beer Sheva, Israel
- Idan Menashe: Department of Public Health, Ben-Gurion University of the Negev, Beer-Sheva, Israel; National Autism Research Center of Israel, Ben-Gurion University of the Negev, Beer-Sheva, Israel

- Judith Miller: Children's Hospital of Philadelphia, Philadelphia, PA, USA; Department of Psychiatry, University of Utah, Salt Lake City, UT, USA
- Nancy Minshew: Department of Psychiatry, University of Pittsburgh School of Medicine, Pittsburgh, PA, USA
- Matthew Mosconi: Life Span Institute and Kansas Center for Autism Research and Training, University of Kansas, Lawrence, KS, USA
- Rachel Nguyen: Center for Autism Research and Translation, University of California Irvine, Irvine, CA, USA
- Norio Ozaki: The Lundbeck Foundation Initiative for Integrative Psychiatric Research, iPSYCH, Aarhus, Denmark; Institute for Glyco-core Research (iGCORE), Nagoya University, Nagoya, Japan
- Aarno Palotie: Program in Medical and Population Genetics, Broad Institute of MIT and Harvard, Cambridge, MA, USA; Analytic and Translational Genetics Unit, Department of Medicine, Massachusetts General Hospital, Boston, MA, USA; Institute for Molecular Medicine Finland (FIMM), University of Helsinki, Helsinki, Finland; Psychiatric & Neurodevelopmental Genetics Unit, Department of Psychiatry, Massachusetts General Hospital, Boston, MA, USA
- Mara Parellada: Department of Child and Adolescent Psychiatry, Hospital General Universitario Gregorio Marañón, IiSGM, CIBERSAM, School of Medicine Complutense University, Madrid, Spain
- Maria Rita Passos-Bueno: Centro de Pesquisas sobre o Genoma Humano e Células tronco, Instituto de Biociências, Universidade de São Paulo, São Paulo, Brazil
- Lisa Pavinato: Department of Medical Sciences, University of Torino, Turin, Italy
- Minshi Peng: Department of Statistics and Data Science, Carnegie Mellon University, Pittsburgh, PA, USA;
- Margaret Pericak-Vance: The John P Hussman Institute for Human Genomics, The University of Miami Miller School of Medicine, Miami, FL, USA
- Antonio M. Persico: Interdepartmental Program 'Autism 0-90', 'Gaetano Martino' University Hospital, University of Messina, Messina, Italy
- Isaac N. Pessah: MIND (Medical Investigation of Neurodevelopmental Disorders) Institute, University of California Davis, Davis, CA, USA; Department of Molecular Biosciences, University of California Davis, School of Veterinary Medicine, Davis, CA, USA
- Kaija Puura: Department of Child Psychiatry, Tampere University and Tampere University Hospital, Tampere, Finland
- Abraham Reichenberg: Seaver Autism Center for Research and Treatment, Icahn School of Medicine at Mount Sinai, New York, NY, USA; Department of Psychiatry, Icahn School

of Medicine at Mount Sinai, New York, NY, USA; The Mindich Child Health and Development Institute, Icahn School of Medicine at Mount Sinai, New York, NY, USA; Department of Environmental Medicine and Public Health, Icahn School of Medicine at Mount Sinai, New York, NY, USA

- Alessandra Renieri: Med Biotech Hub and Competence Center, Department of Medical Biotechnologies, University of Siena, Siena, Italy; Medical Genetics, , University of Siena, Siena, Italy; Genetica Medica, Azienda Ospedaliera Universitaria Senese, Siena, Italy
- Kathryn Roeder: Department of Statistics and Data Science, Carnegie Mellon University, Pittsburgh, PA, USA; Computational Biology Department, Carnegie Mellon University, Pittsburgh, PA, USA
- Stephan J. Sanders: Department of Psychiatry, UCSF Weill Institute for Neurosciences, University of California San Francisco, San Francisco, CA, USA
- Sven Sandin: Seaver Autism Center for Research and Treatment, Icahn School of Medicine at Mount Sinai, New York, NY, USA; Department of Psychiatry, Icahn School of Medicine at Mount Sinai, New York, NY, USA; Department of Medical Epidemiology and Biostatistics, Karolinska Institutet, Stockholm, Sweden
- F. Kyle Satterstrom: Program in Medical and Population Genetics, Broad Institute of MIT and Harvard, Cambridge, MA, USA; Stanley Center for Psychiatric Research, Broad Institute of MIT and Harvard, Cambridge, MA, USA; Analytic and Translational Genetics Unit, Department of Medicine, Massachusetts General Hospital, Boston, MA, USA
- Stephen W. Scherer: Program in Genetics and Genome Biology, The Centre for Applied Genomics, The Hospital for Sick Children, Toronto, Ontario, Canada; Department of Molecular Genetics and McLaughlin Centre, University of Toronto, Toronto, Ontario, Canada
- Sabine Schlitt: Department of Psychiatry, Graduate School of Medicine, Nagoya University, Nagoya, Japan
- Rebecca J. Schmidt: MIND (Medical Investigation of Neurodevelopmental Disorders) Institute, University of California Davis, Davis, CA, USA
- Lauren Schmitt: Institute for Juvenile Research, Department of Psychiatry, University of Illinois at Chicago, Chicago, IL, USA
- Katja Schneider-Momm: Department of Psychiatry, Graduate School of Medicine, Nagoya University, Nagoya, Japan
- Paige M. Siper: Seaver Autism Center for Research and Treatment, Icahn School of Medicine at Mount Sinai, New York, NY, USA; Department of Psychiatry, Icahn School of Medicine at Mount Sinai, New York, NY, USA; The Mindich Child Health and Development Institute, Icahn School of Medicine at Mount Sinai, New York, NY, USA

- Laura Sloofman: Seaver Autism Center for Research and Treatment, Icahn School of Medicine at Mount Sinai, New York, NY, USA; Department of Psychiatry, Icahn School of Medicine at Mount Sinai, New York, NY, USA; The Mindich Child Health and Development Institute, Icahn School of Medicine at Mount Sinai, New York, NY, USA
- Moyra Smith: Center for Autism Research and Translation, University of California Irvine, Irvine, CA, USA
- Christine R. Stevens: Program in Medical and Population Genetics, Broad Institute of MIT and Harvard, Cambridge, MA, USA; Stanley Center for Psychiatric Research, Broad Institute of MIT and Harvard, Cambridge, MA, USA; Analytic and Translational Genetics Unit, Department of Medicine, Massachusetts General Hospital, Boston, MA, USA
- Pål Suren: Norwegian Institute of Public Health, Oslo, Norway
- James S. Sutcliffe: Department of Molecular Physiology & Biophysics and Psychiatry, Vanderbilt University School of Medicine, Nashville, TN, USA; Vanderbilt Genetics Institute, Vanderbilt University School of Medicine, Nashville, TN, USA
- John A. Sweeney: Department of Psychiatry, University of Cincinnati, Cincinnati, OH, USA
- Michael E. Talkowski: Center for Genomic Medicine, Massachusetts General Hospital, Boston, MA, USA; Program in Medical and Population Genetics, Broad Institute of MIT and Harvard, Cambridge, MA, USA; Department of Neurology, Massachusetts General Hospital and Harvard Medical School, Boston, MA, USA; Stanley Center for Psychiatric Research, Broad Institute of MIT and Harvard, Cambridge, MA, USA; Program in Bioinformatics and Integrative Genomics, Harvard Medical School, Boston, MA, USA
- Flora Tassone: MIND (Medical Investigation of Neurodevelopmental Disorders) Institute, University of California Davis, Davis, CA, USA; Department of Biochemistry and Molecular Medicine, University of California Davis, School of Medicine, Sacramento, CA, USA
- Karoline Teufel: Department of Psychiatry, Graduate School of Medicine, Nagoya University, Nagoya, Japan
- Elisabetta Trabetti: Department of Neurosciences, Biomedicine and Movement Sciences, Section of Biology and Genetics, University of Verona, Verona, Italy
- Slavica Trajkova: Department of Medical Sciences, University of Torino, Turin, Italy
- Maria del Pilar Trelles: Seaver Autism Center for Research and Treatment, Icahn School of Medicine at Mount Sinai, New York, NY, USA; Department of Psychiatry, Icahn School of Medicine at Mount Sinai, New York, NY, USA
- Brie Wamsley: Program in Neurogenetics, Department of Neurology, David Geffen School of Medicine, University of California Los Angeles, Los Angeles, CA, USA
- Jaqueline Y. T. Wang: Centro de Pesquisas sobre o Genoma Humano e Células tronco, Instituto de Biociências, Universidade de São Paulo, São Paulo, Brazil

- Lauren A. Weiss: Department of Psychiatry, UCSF Weill Institute for Neurosciences, University of California San Francisco, San Francisco, CA, USA
- Mullin H. C. Yu : Department of Pediatrics and Adolescent Medicine, Duchess of Kent Children's Hospital, The University of Hong Kong, Hong Kong Special Administrative Region, China
- Ryan Yuen: Program in Genetics and Genome Biology, The Centre for Applied Genomics, The Hospital for Sick Children, Toronto, Ontario, Canada

# Autism Prenatal and Sex Differences (APEX)

- Dwaipayan Adhya: Department of Psychiatry, Autism Research Centre, University of Cambridge, Cambridge, Cambridgeshire, CB2 8AH, UK; Department of Basic and Clinical Neuroscience, Maurice Wohl Clinical Neuroscience Institute, Institute of Psychiatry, Psychology and Neuroscience, King's College London, London, UK
- Carrie Allison: Department of Psychiatry, Autism Research Centre, University of Cambridge, Cambridge, Cambridgeshire, CB2 8AH, UK
- Bonnie Ayeung: Department of Psychiatry, Autism Research Centre, University of Cambridge, Cambridge, Cambridgeshire, CB2 8AH, UK
- Rosie Bamford: University of Exeter Medical School, Exeter, Devon, EX2 5DW, UK
- Simon Baron-Cohen: Department of Psychiatry, Autism Research Centre, University of Cambridge, Cambridge, Cambridgeshire, CB2 8AH, UK
- Richard Bethlehem: Department of Psychiatry, Autism Research Centre, University of Cambridge, Cambridge, Cambridgeshire, CB2 8AH, UK; Department of Psychiatry, Brain Mapping Unit, University of Cambridge, Cambridge, Cambridgeshire, CB2 8AH, UK
- Tal Biron-Shental: Department of Obstetrics and Gynecology, Meir Medical Center, Kfar Saba, Israel; Sackler Faculty of Medicine, Tel Aviv University, Tel Aviv, Israel
- Graham Burton: Centre for Trophoblast Research, Department of Physiology, Development and Neuroscience, University of Cambridge, Cambridge, CB2 3EG, UK
- Wendy Cowell: University of Exeter Medical School, Exeter, Devon, EX2 5DW, UK
- Jonathan Davies: University of Exeter Medical School, Exeter, Devon, EX2 5DW, UK
- Dori Floris: Department of Cognitive Neuroscience, Donders Institute for Brain Cognition and Behaviour, Radboud University Medical Centre, 6525EN Nijmegen, The Netherlands; Methods of Plasticity Research, Department of Psychology, University of Zurich, Zurich, Switzerland
- Alice Franklin: University of Exeter Medical School, Exeter, Devon, EX2 5DW, UK

- Lidia Gabis: Tel Aviv University, Wolfson Hospital and Maccabi healthcare, Tel Aviv, Israel
- Daniel Geschwind: Program in Neurobehavioral Genetics, Center for Autism Research and Treatment, Semel Institute, David Geffen School of Medicine, University of California, Los Angeles, Los Angeles, CA 90095, USA; Department of Neurology, David Geffen School of Medicine, University of California, Los Angeles, 695 Charles E. Young Drive South, Los Angeles, CA 90095, USA; Department of Psychiatry, Semel Institute, David Geffen School of Medicine, University of California, Los Angeles, 695 Charles E. Young Drive South, Los Angeles, CA 90095, USA; Department of Human Genetics, David Geffen School of Medicine, University of California, Los Angeles, CA 90095, USA
- David M. Greenberg: Interdisciplinary Department of Social Sciences, Bar-Ilan University, Ramat Gan, Israel; Department of Music, Bar-Ilan University, Ramat Gan, Israel; Autism Research Centre, Department of Psychiatry, University of Cambridge, Cambridge, UK
- Yuanjun Gu: Department of Psychiatry, Autism Research Centre, University of Cambridge, Cambridge, Cambridgeshire, CB2 8AH, UK
- Alexandra Havdahl: Center for Genetic Epidemiology and Mental Health, Norwegian Institute of Public Health, Oslo, Norway; Nic Waals Institute, Lovisenberg Diakonale Hospital, Oslo, Norway; PROMENTA Research Center, Department of Psychology, University of Oslo, Oslo, Norway; MRC Integrative Epidemiology Unit, University of Bristol, Bristol, UK
- Alexander Heazell: Maternal and Fetal Health Research Centre, School of Medical Sciences, University of Manchester, Manchester, UK; Saint Mary's Hospital, Manchester University NHS Foundation Trust, Manchester, UK
- Rosemary Holt: Department of Psychiatry, Autism Research Centre, University of Cambridge, Cambridge, Cambridgeshire, CB2 8AH, UK
- Matthew Hurles: Wellcome Sanger Institute, Hinxton, Cambridgeshire, CB10 1SA, UK
- Yumnah Khan: Department of Psychiatry, Autism Research Centre, University of Cambridge, Cambridge, Cambridgeshire, CB2 8AH, UK
- Meng-Chuan Lai: Department of Psychiatry, Autism Research Centre, University of Cambridge, Cambridge, Cambridgeshire, CB2 8AH, UK; The Margaret and Wallace McCain Centre for Child, Youth & Family Mental Health and Azrieli Adult Neurodevelopmental Centre, Campbell Family Mental Health Research Institute, Centre for Addiction and Mental Health, Toronto, Canada; Department of Psychiatry and Autism Research Unit, The Hospital for Sick Children, Toronto, Canada; Department of Psychiatry, Temerty Faculty of Medicine, University of Toronto, Toronto, Canada; Department of Psychiatry, National Taiwan University Hospital and College of Medicine, Taipei, Taiwan

- Madeline Lancaster: MRC Laboratory for Molecular Biology, University of Cambridge, Cambridge, UK
- Michael Lombardo: Laboratory for Autism and Neurodevelopmental Disorders, Center for Neuroscience and Cognitive Systems, Istituto Italiano di Tecnologia, Rovereto, Italy
- Hilary Martin: Wellcome Sanger Institute, Hinxton, Cambridgeshire, CB10 1SA, UK
- Jose Gonzalez Martinez: MRC Laboratory for Molecular Biology, University of Cambridge, Cambridge, UK
- Jonathan Mill: University of Exeter Medical School, Exeter, Devon, EX2 5DW, UK
- Mahmoud Koko Musa: Wellcome Sanger Institute, Hinxton, Cambridgeshire, CB10 1SA, UK
- Kathy Niakan: Cambridge Reproduction, University of Cambridge, Cambridge, UK; The Centre for Trophoblast Research, Department of Physiology, Development and Neuroscience, University of Cambridge, Cambridge, UK; Human Embryo and Stem Cell Laboratory, The Francis Crick Institute, London, UK; Wellcome Trust-Medical Research Council Stem Cell Institute, University of Cambridge, Jeffrey Cheah Biomedical Centre, Cambridge, UK; Epigenetics Programme, Babraham Institute, Cambridge, UK
- Adam Pavlinek: Department of Basic and Clinical Neuroscience, Institute of Psychiatry, Psychology and Neuroscience, King's College London, London, UK; MRC Centre for Neurodevelopmental Disorders, King's College London, London, UK
- Lucia Dutan Polit: Department of Basic and Clinical Neuroscience, Institute of Psychiatry Psychology and Neuroscience, King's College London, London, UK; MRC Centre for Neurodevelopmental Disorders, King's College London, London, UK
- Marcin Radecki: Department of Psychiatry, Autism Research Centre, University of Cambridge, Cambridge, Cambridgeshire, CB2 8AH, UK; Social and Affective Neuroscience Group, IMT School for Advanced Studies Lucca, Lucca 55100, Italy
- David Rowitch: Wellcome-MRC Cambridge Stem Cell Institute, University of Cambridge, Cambridge CB2 0AW, UK; Department of Paediatrics, University of Cambridge, Cambridge, UK
- Laura Sichlinger: Department of Basic and Clinical Neuroscience, The Maurice Wohl Clinical Neuroscience Institute, Institute of Psychiatry, Psychology & Neuroscience, King's College London, London, UK; MRC Centre for Neurodevelopmental Disorders, Institute of Psychiatry, Psychology & Neuroscience, King's College London, London, UK
- Deepak Srivastava: Department of Basic and Clinical Neuroscience, Institute of Psychiatry Psychology and Neuroscience, King's College London, London, UK; MRC Centre for Neurodevelopmental Disorders, King's College London, London, UK
- Alexandros Tsompanidis: Department of Psychiatry, Autism Research Centre, University of Cambridge, Cambridge, Cambridgeshire, CB2 8AH, UK

- Florina Uzefovsky: Ben-Gurion University of the Negev, Beer-Sheva, 84105, Israel
- Varun Warrier: Department of Psychiatry, Autism Research Centre, University of Cambridge, Cambridge, Cambridgeshire, CB2 8AH, UK
- Elizabeth Weir: Department of Psychiatry, Autism Research Centre, University of Cambridge, Cambridge, Cambridgeshire, CB2 8AH, UK
- Xinhe Zhang: Department of Psychiatry, Autism Research Centre, University of Cambridge, Cambridge, Cambridgeshire, CB2 8AH, UK

# Supplemental Figures

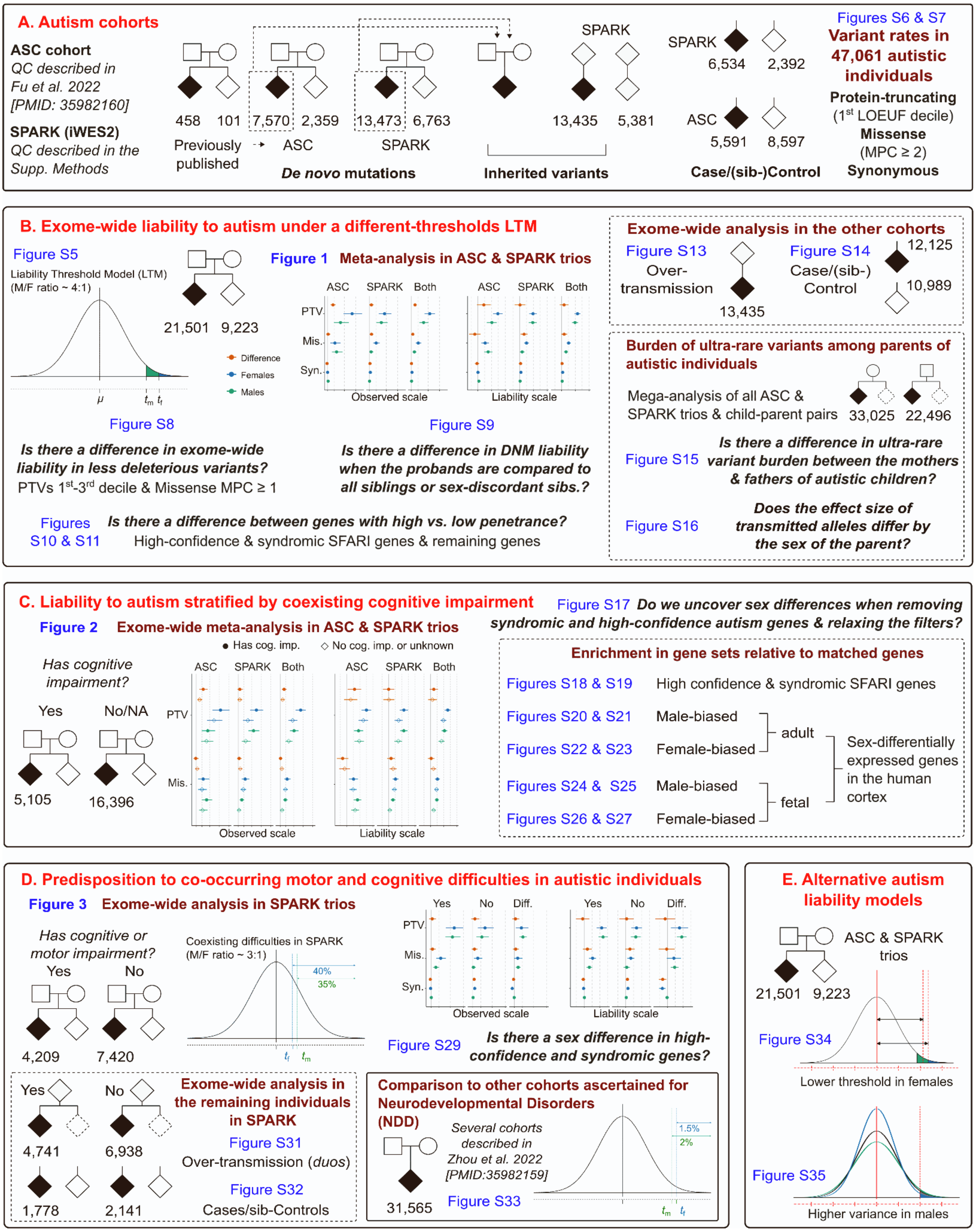


Figure S1: Flow chart summarizing the analyses in this study.

The main analyses were performed (separately) for *de novo* and rare inherited variants ascertained in the family-based cohort. Additional exome-wide analyses in the remaining individuals with one/no sequenced parent(s) are shown where applicable. For each analysis or research question, the flowchart indicates the relevant main and supplemental figures.

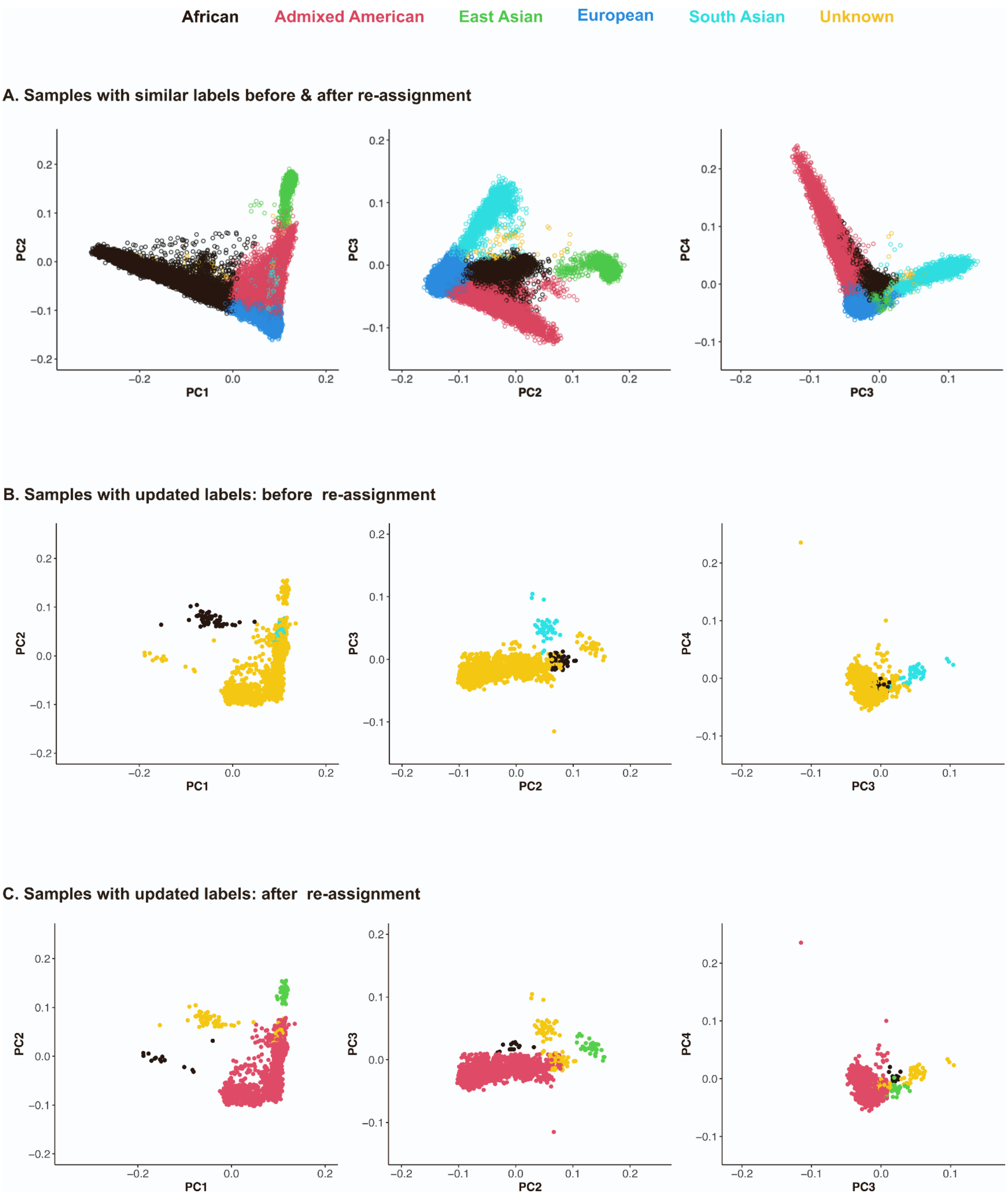


Figure S2: Population labels of SPARK samples.

Principal component analysis (PCA) was used to check the validity of the pre-assigned population labels from SPARK against the top principal components projected on the 1000 Genomes space (reference samples not shown). Most samples clustered as expected (**A**). Samples with unknown labels and those not clustering with their respective pre-assigned groups (**B**) were re-classified (**C**). See section 1.3. 'Ancestry Inference' of the Supplemental Methods and Tables S3 & S4 for details.

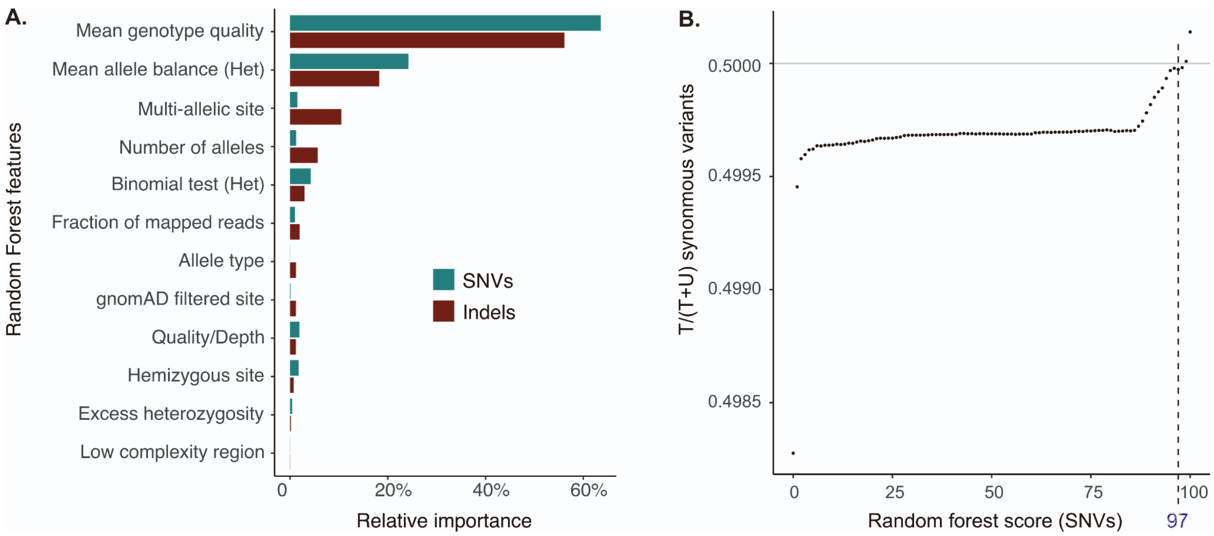


Figure S3: Assigning variant quality scores using Random Forest.

A Random Forest was trained using 12 features (**A**) to assign a variant-level quality score between 0 (lowest quality) and 100 (highest quality). The average genotype quality per variant (all genotypes) and mean allele balance per variant (heterozygous genotypes) had the highest relative importance (y-axis). The fraction of the transmitted (T) alleles from the total transmitted & untransmitted (T+U) parental synonymous variants achieved after filtering with increasing stringency (higher Random Forest scores) is shown in **B**. Single Nucleotide Variants (SNVs) with scores > 97 (dotted line) and Insertions-Deletions (Indels) with scores > 92 were retained. See section 1.6. (Variant QC) for further details.

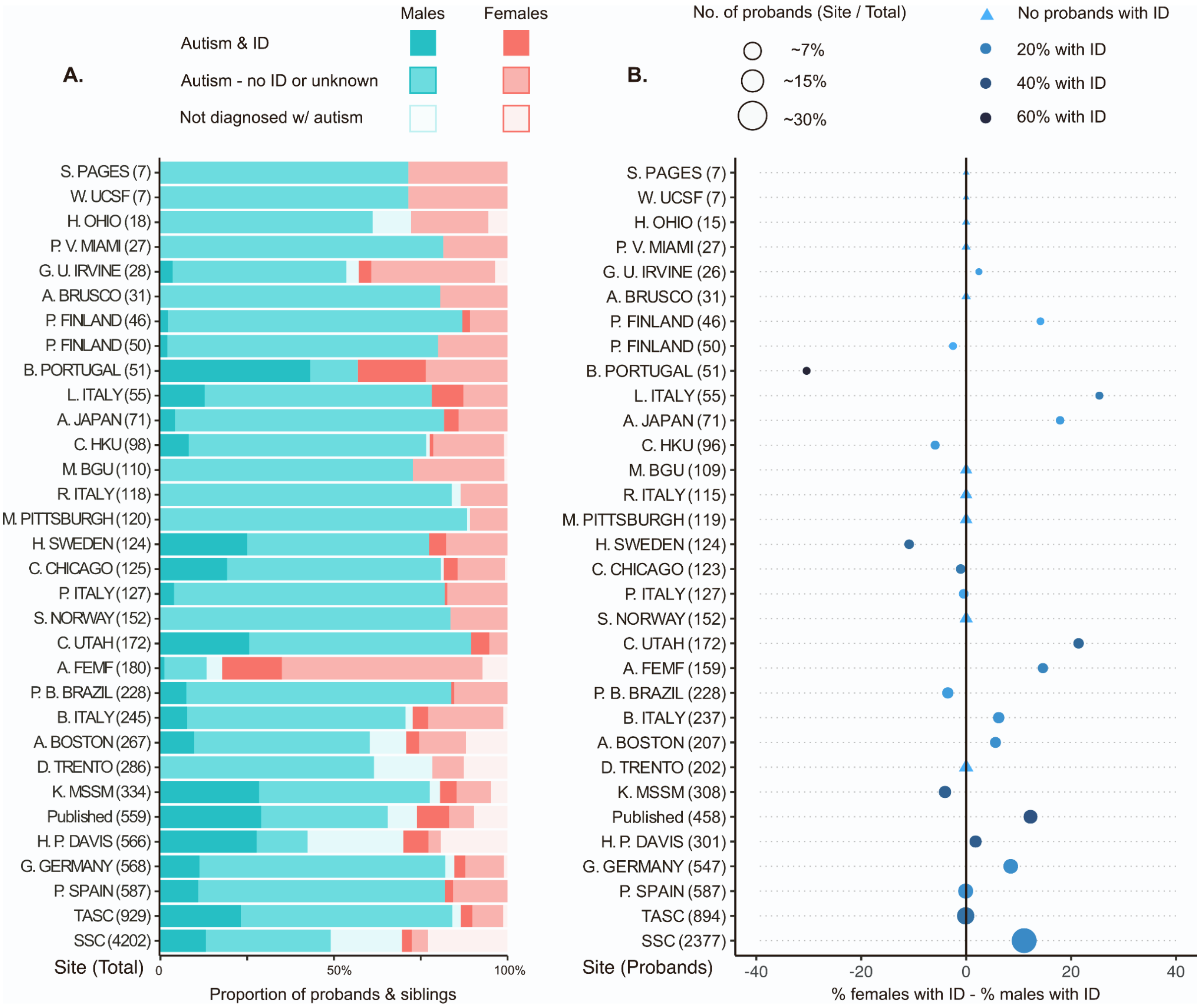


Figure S4: The proportion of autistic individuals with intellectual disability across the ASC cohorts.

**A**, The sample size of each sub-cohort in the ASC trios dataset and the proportion of autistic probands with intellectual disability (ID), without intellectual disability (or unknown), and siblings not diagnosed with autism. The cohorts are sorted by their size (total of probands and siblings) along the y axis. See Table S2 for these counts. **B**, The difference in the proportion of females with ID and males with ID within each sub-cohort. The 'dot' size indicates the percentage of individuals with ID in the cohort (both males and females), whereas its position along the x-axis indicates sex bias; if placed in the middle, then the % of autistic individuals with ID is equal amongst males and females, to the right means more females with ID, to the left more males with ID.

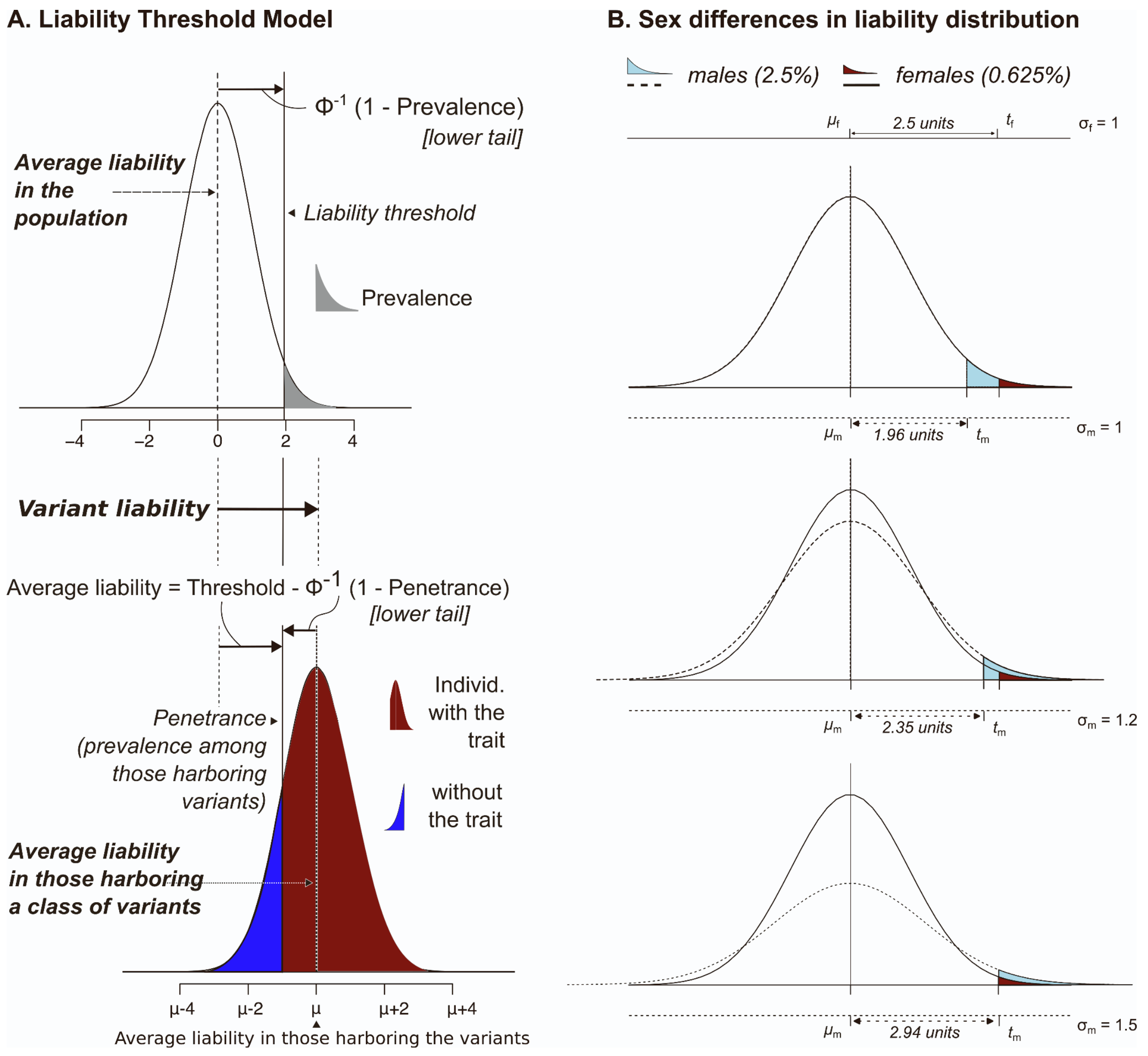


Figure S5: Estimating rare variant liability from population prevalence and variant rates.

**A**, The Liability Threshold Model postulates that risk factors act additively and underlie a normally distributed liability distribution in the general population (top), where the threshold determines the trait prevalence. When examining a group of rare variants (bottom), the average liability amongst all individuals harboring these variants will reflect the average effect size of the variant class. If the prevalence of the trait among individuals harboring a class of rare variants is known ('penetrance'), the two distributions can be juxtaposed (or overlaid), leveraging the threshold as a reference point, to get an estimate of the distance between their means. See the 'Variant liability' section for a detailed description. **B**, Sex differences in population prevalence between two groups (e.g. males and females) can arise from differences in the threshold or the variance. The three models show how different properties of the liability distribution in females (solid line) and males (dotted line) can underlie the same male-to-female ratio. Assuming the liability distribution in females has a variance of one and a threshold that is +2.5 units from the mean (prevalence = 0.625%), a prevalence of 2.5% in males (4:1 ratio) can result from males having the same variance but a lower threshold than females (top), higher variance but the same threshold (bottom) or a combination of the two (middle).

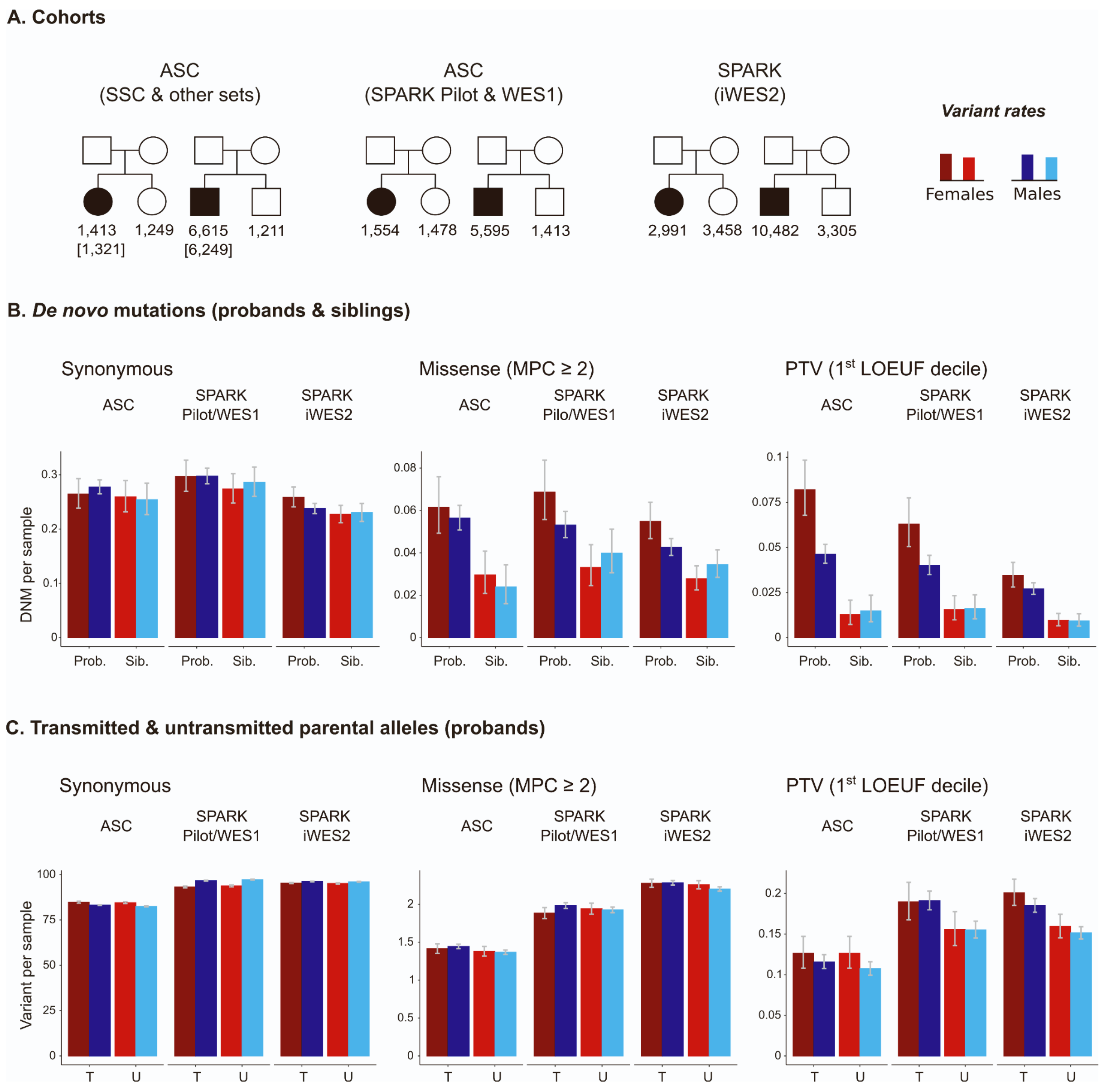


Figure S6: Rare *de novo* and inherited variant rates in the ASC & SPARK trio-sequenced cohorts.

**A**, The pedigrees show the sample size used for counting inherited variants & calculating *de novo* rates in the Autism Sequencing Consortium (ASC) cohort and the Simons Foundation Powering Autism Research for Knowledge (SPARK) cohort (sample size for inherited variants given between brackets as some individuals in the ASC cohort did not have information on inherited alleles). **B**, *De novo* mutation rates in the probands (prob.) and siblings (sib.). **C**, Average counts of transmitted (T) and untransmitted (U) parental alleles at rare variants (MAF < 0.1%). SPARK Pilot/WES1 is shown for comparison *versus* SPARK iWES2, as it was processed using the same pipeline used for the remaining ASC samples. The error bars indicate the 95% confidence intervals of the variant rates. See section 1 of the Supplemental Results for details.

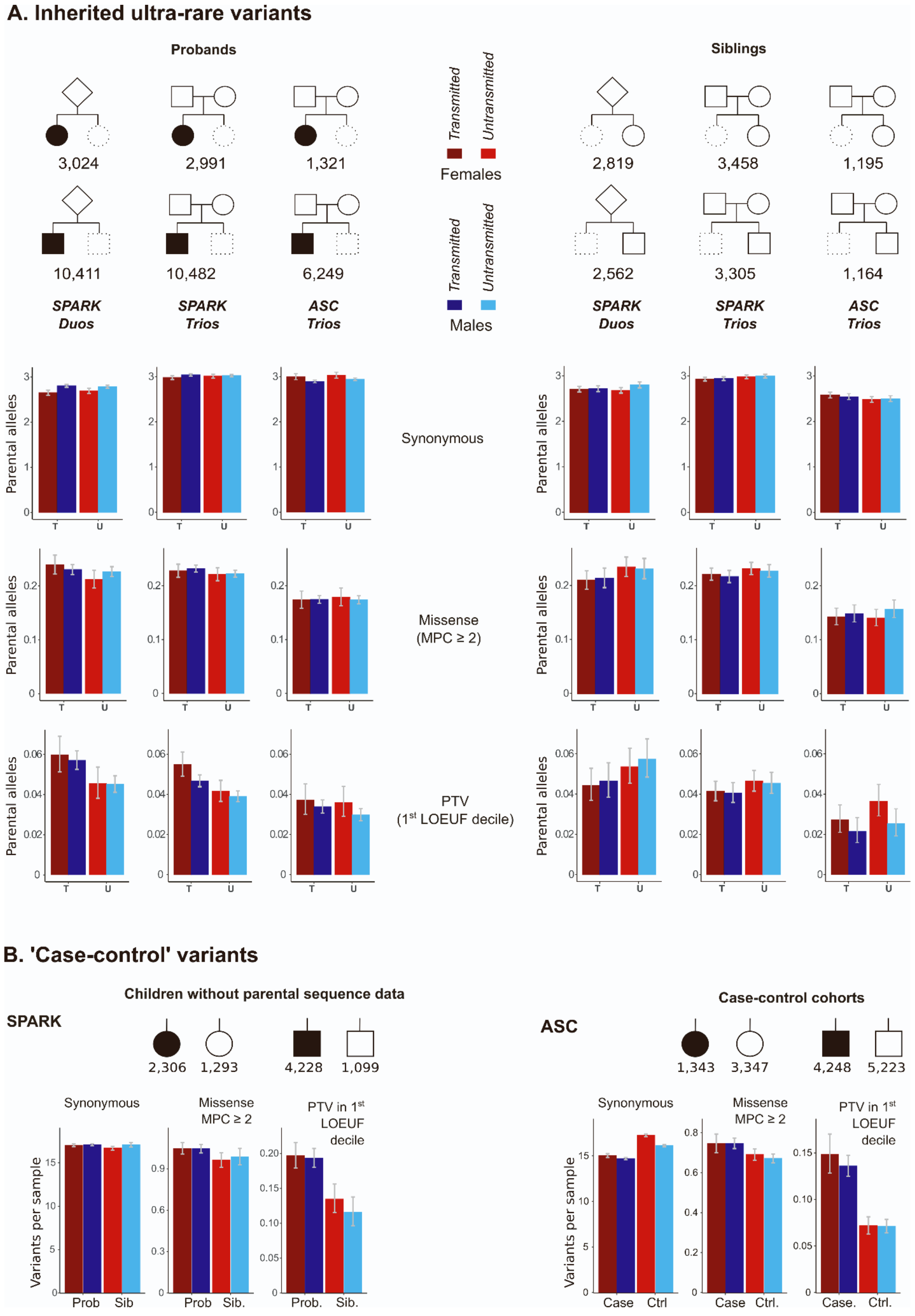


Figure S7: Ultra-rare inherited and case-control variant rates in SPARK and ASC cohorts.

**A**, The transmission rates of ultra-rare parental alleles (seen in one parent and not in gnomAD) in probands and siblings with exome data from one or both parents in the Simons Foundation Powering Autism Research for Knowledge (SPARK) cohort (duos & trios) and the Autism Sequencing Consortium (ASC) cohort (trios). **B**, Ultra-rare variant rates (allele frequency < 0.005%) in the remaining children in SPARK, who did not have parental sequence data, and the ASC case-control cohorts. The pedigrees show the sample size used for counting parental alleles (**A**) or case-control variants (**B**). The bar plots show the sex-stratified ultra-rare variant rates; The error bars indicate the 95% confidence intervals of the variant rates. See section 1 of the Supplemental Results for details.

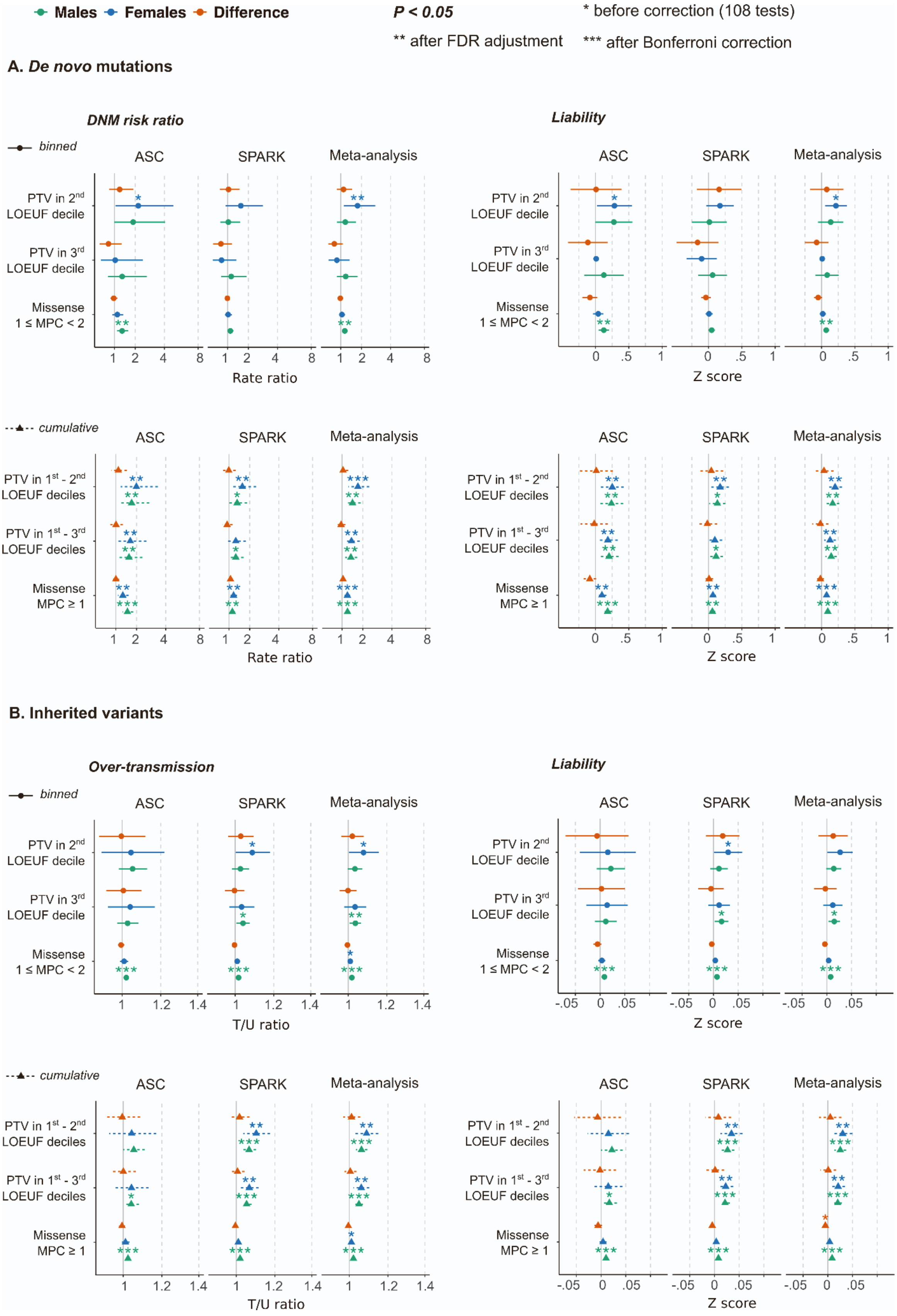


Figure S8: Sensitivity analysis for damaging *de novo* and rare inherited variants in LoF-intolerant genes.

**A,** Sex-stratified *de novo* mutation rate ratios (left) and liability (right) in trio-sequenced individuals in the Simons Foundation Powering Autism Research for Knowledge study (SPARK) and the Autism

Sequencing Consortium (ASC) cohorts. **B**, Over-transmission and liability of inherited variants in these cohorts. Whereas the primary analysis in Figure 1 focused on protein-truncating variants in highly-LoF-constrained genes, these analyses examined protein-truncating variants in less constrained genes and damaging missense variants with lower MPC scores. Error bars show 95% confidence intervals.

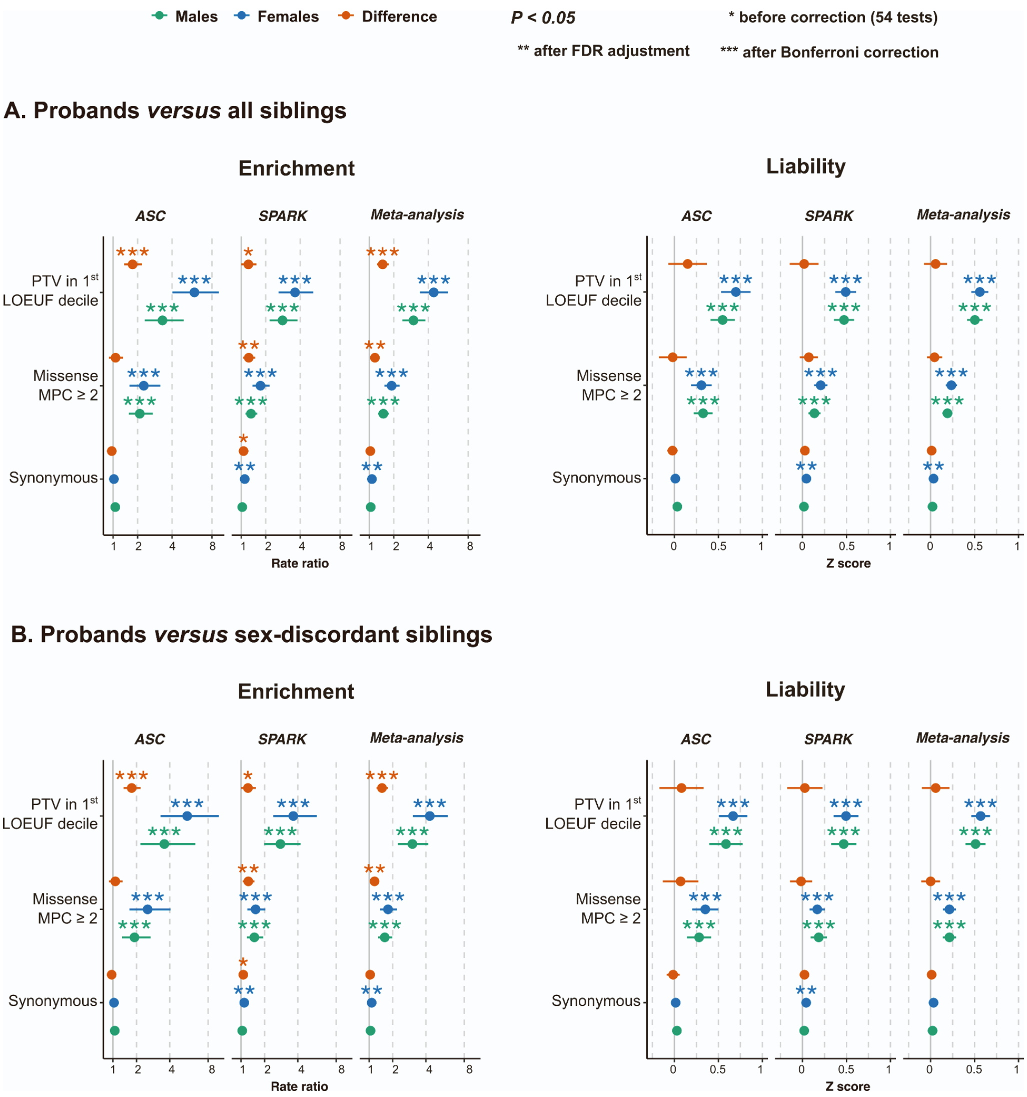


Figure S9: DNM enrichment in autistic probands *versus* all siblings and sex-discordant siblings.

Sex-stratified *de novo* mutation enrichment (left) and liability (right) were measured by comparing the DNM rates in female or male probands to the DNM rates seen in all siblings (males and females) in **A** or sex-discordant siblings (female pronands vs. male siblings, male probands vs. female siblings) in **B**, which is comparable to the analysis presented in Figure 1B (*versus* sex-matched siblings). Error bars show 95% confidence intervals. For sex differences, a rate ratio > 1 indicates that females show a higher enrichment; a Z score > 0 indicates that females show a higher effect size on the liability scale. The sex differences on the liability scale reflect the Z score difference between females and males (*versus* all siblings in **A** and *versus* sex-discordant siblings in **B**), similar to what is shown in Figure 1B (*versus* sex-alike siblings). Note that the sex differences in DNM rate ratios are obtained through direct comparisons of DNM rates of female and male probands and are therefore independent of the set of siblings used (i.e. identical values in A, B and Figure 1B). However, the differences in liability are Z-score differences, and thus represent different tests in A, B and Figure 1B.

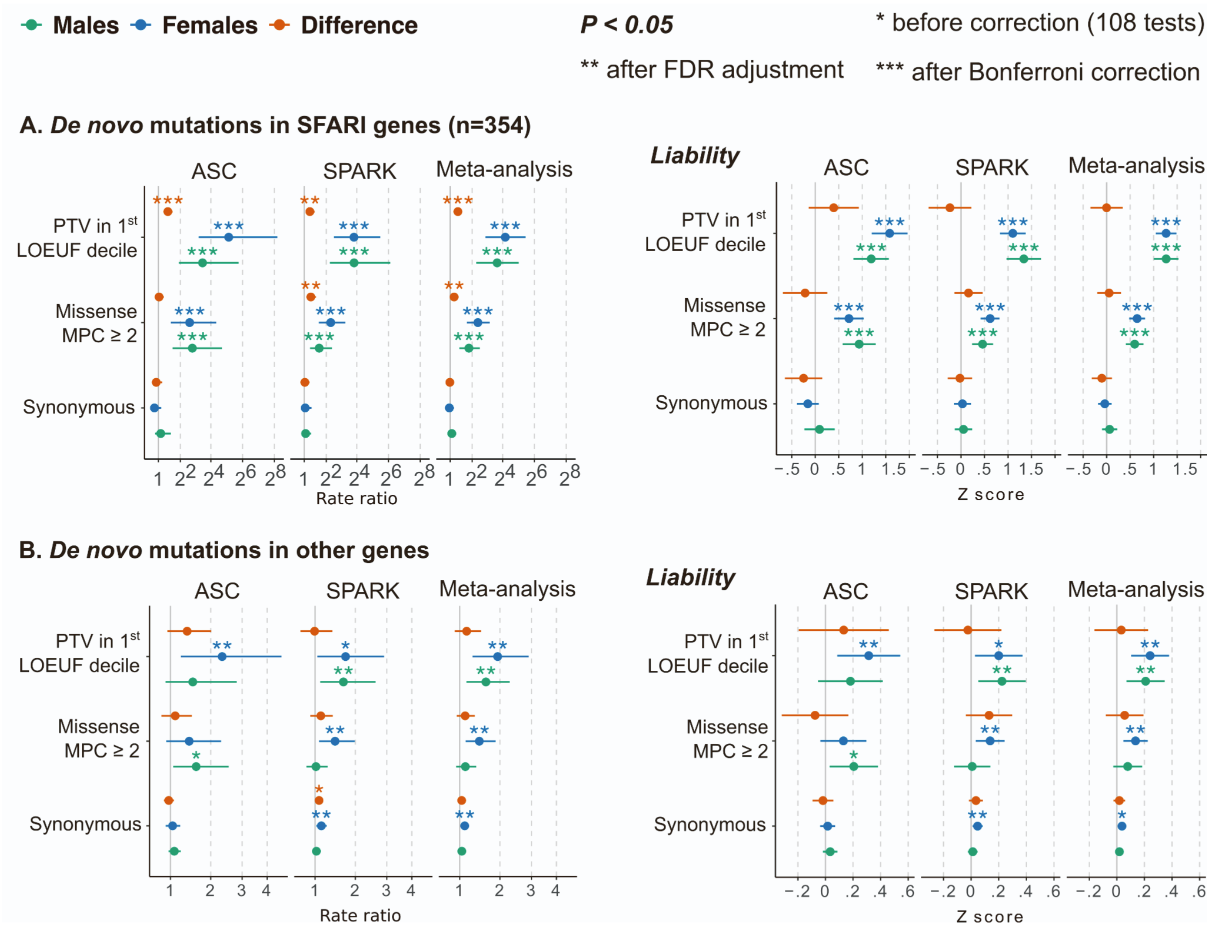


Figure S10: Enrichment of *de novo* mutations in 354 high-confidence and syndromic SFARI autism predisposition genes *versus* all other genes.

Sex-stratified *de novo* mutation rate ratios (left) and liability (right) (see Methods) in SFARI genes (**A**) and all other genes (**B**). For sex differences, a rate ratio > 1 indicates that females show a higher enrichment; a Z score > 0 indicates that females show a higher effect size on the liability scale. Error bars show 95% confidence intervals. Similar to Figure 1B; See Figure S11 for inherited variants.

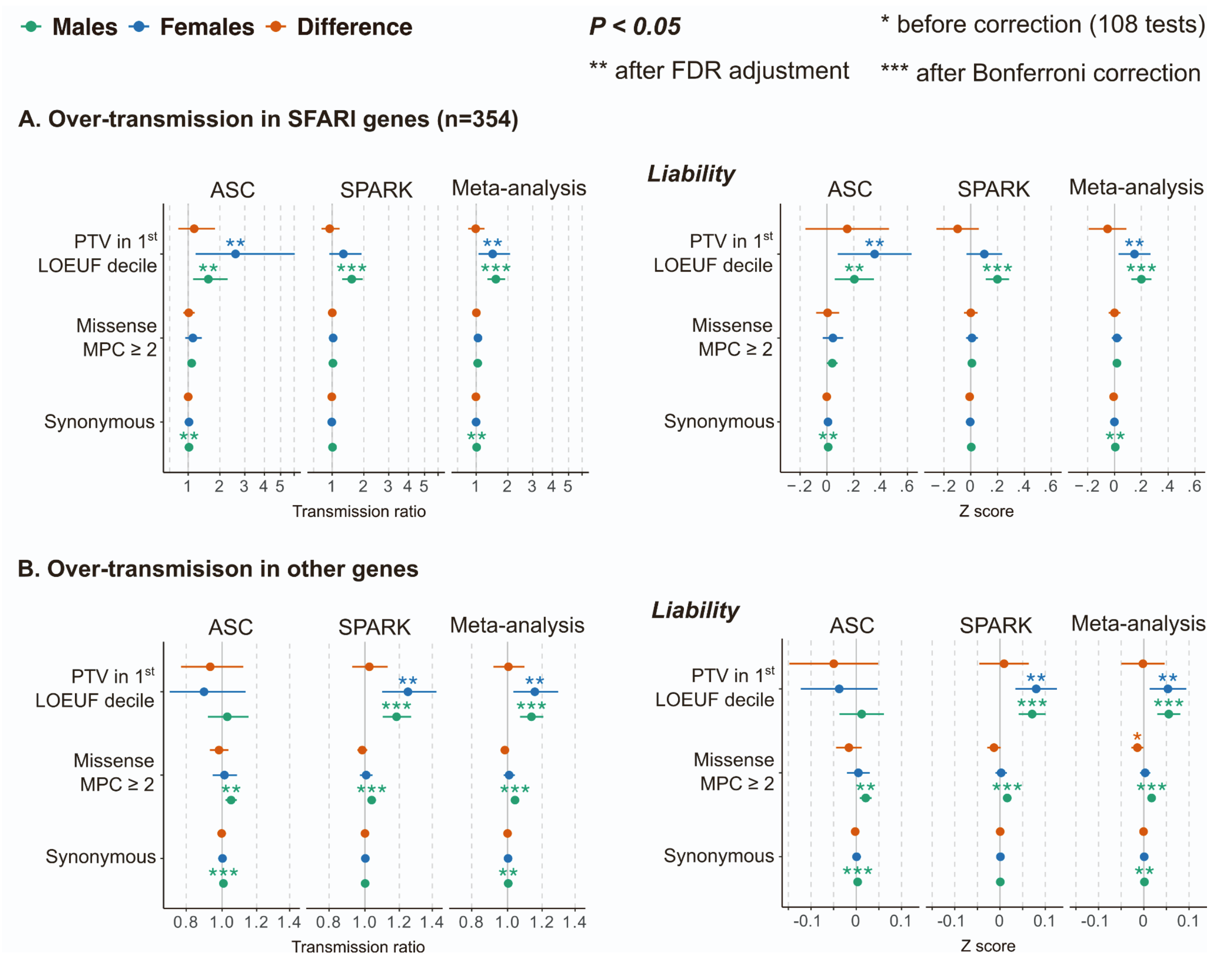


Figure S11: Over-transmission of rare inherited variants in 354 high-confidence and syndromic SFARI autism predisposition genes *versus* all other genes.

Over-transmission (left) and liability (right) of rare inherited variants in SFARI high-confidence genes (**A**) *versus* all other genes (**B**) (see Methods). Error bars show 95% confidence intervals. Note that the scale on the x-axis is different in A and B. Similar to Figure 1C. See Figure S10 for *de novo* mutations.

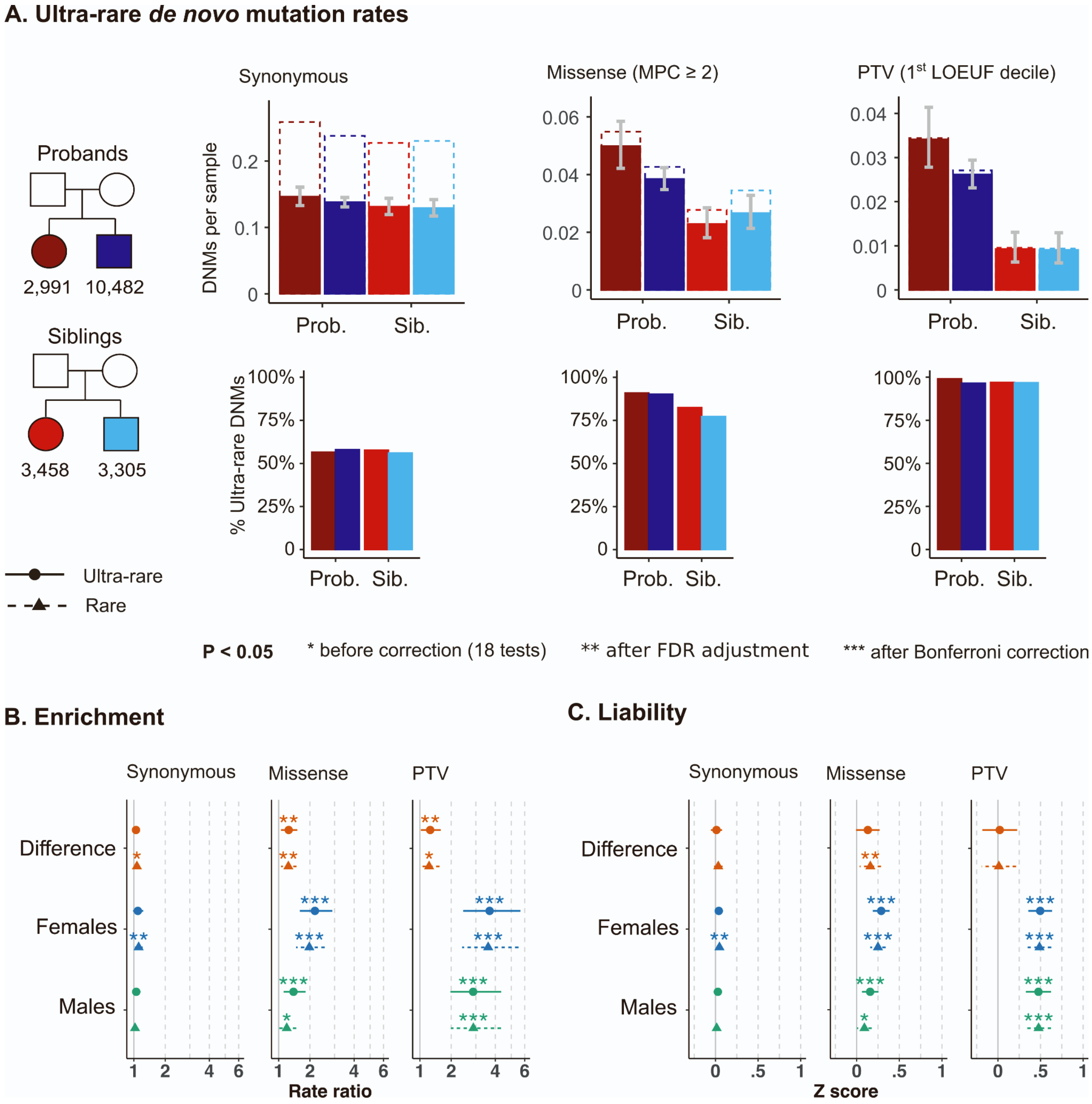


Figure S12: Enrichment of ultra-rare *de novo* mutations in SPARK trio-sequenced individuals.

**A**, Rates of ultra-rare *de novo* mutations (DNM) (filled bars) compared to all rare DNMs (dotted lines). Most damaging protein-truncating and missense DNMs were ultra-rare compared to about half of the synonymous DNMs. **B,** Ultra-rare DNM rate ratios between probands and siblings, compared to rare DNMs. **C**, The liability of ultra-rare and rare DNMs. There was no significant difference in the average liability of ultra-rare synonymous DNM, in contrast to the difference seen in rare DNMs. Error bars show 95% confidence intervals.

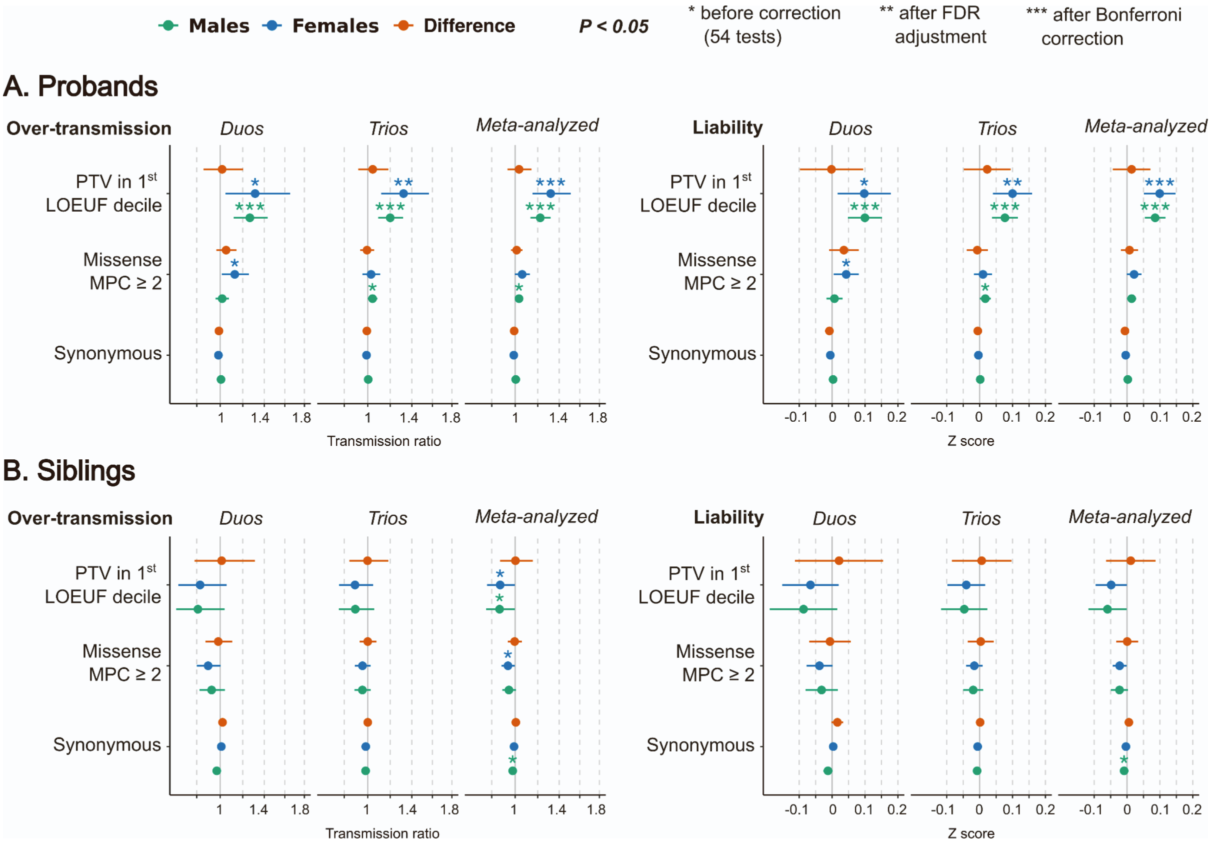


Figure S13: Over-transmission of ultra-rare variants in SPARK.

Over-transmission of ultra-rare variants (i.e. seen in one parent in the dataset, absent from gnomAD) was studied in child-parent pairs with one (duos) or two sequenced parents (trios), then meta-analyzed. This analysis was performed in probands (**A**) and siblings (**B**). Error bars show 95% confidence intervals. Related to over-transmission analysis of rare (MAF < 0.1%) variants in trios (Figure 1C). See Figure S7A for the sample size of these cohorts.

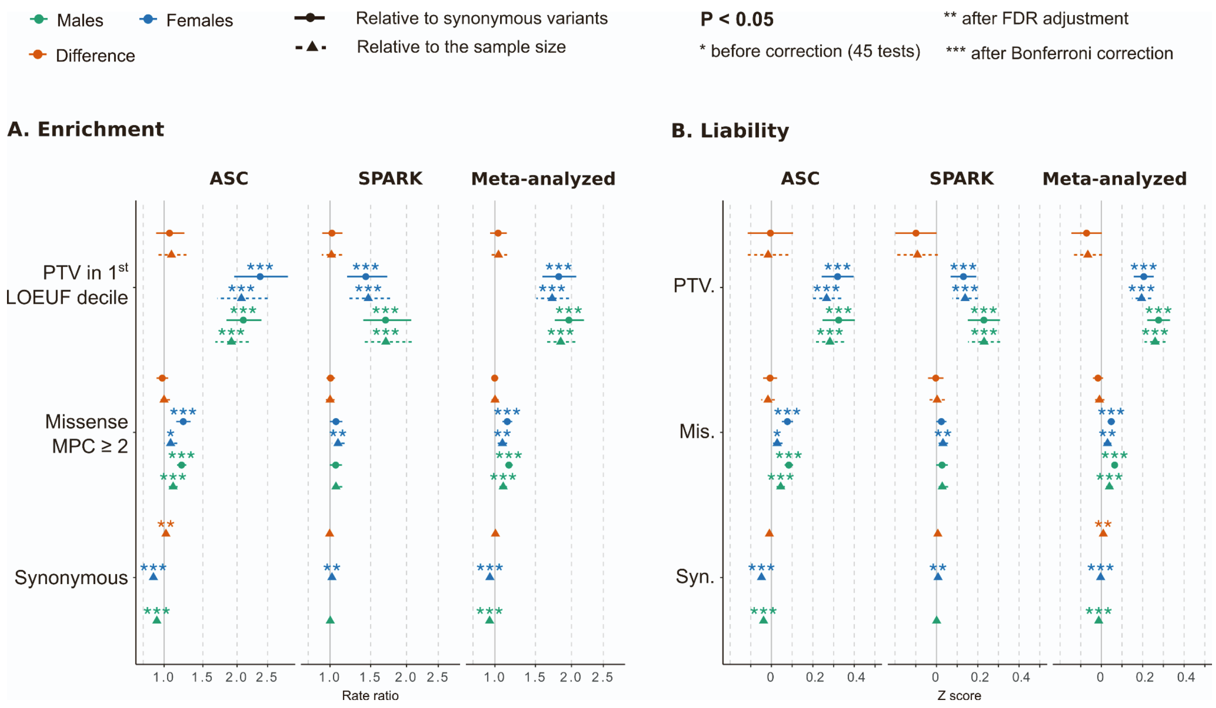


Figure S14: Enrichment of ultra-rare variants the case-control cohorts.

Ultra-rare variant rates in autism cases (ASC) and autistic probands without parental sequence data (SPARK) were compared to autism controls (ASC) or siblings not diagnosed with autism (SPARK). The error bars show the 95% confidence intervals of the effect size on the observed scale (**A**) and liability scale (**B**). The enrichment and liability were assessed relative to the sample size of the case/control cohorts (i.e. assuming that the expected rare variant burden per sample is similar across cohorts). To account for the differences in ultra-rare variant counts arising from the differences in ancestry, damaging missense and protein truncating variants were also compared to the expected rate ratio from synonymous variants (i.e. normalizing the average variant rates using synonymous variant counts). See Figure S7B for the sample sizes and ultra-rare variant rates.

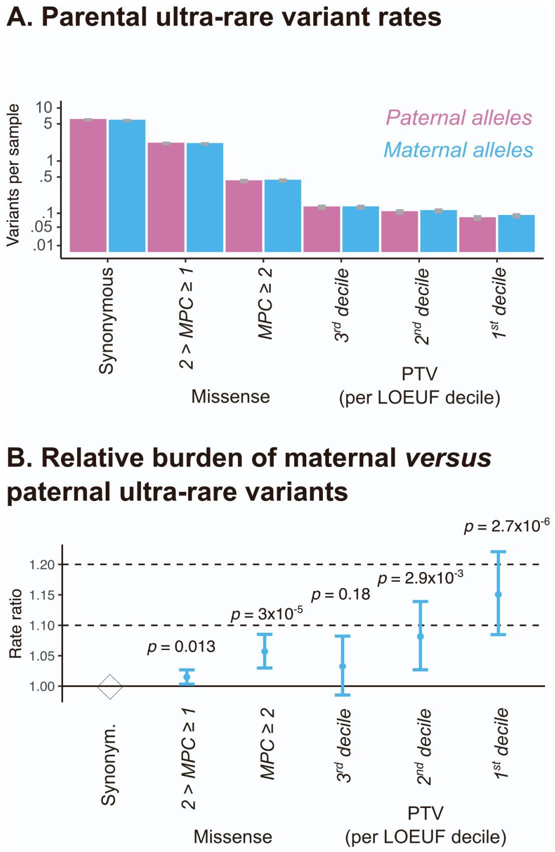


Figure S15: Parental differences in the burden of ultra-rare variants.

The transmission of ultra-rare variants (i.e. variants seen in one parent in the dataset, absent from gnomAD) was evaluated across all child-parent pairs from SPARK and ASC cohorts encompassing 34,478 (unique) autistic children (21,043 trios & 13,435 duos). Each parent-child pair was considered separately, resulting in 55,521 pairs. **A**, Ultra-rare variant rates in the mothers and fathers, pooled across all cohorts. Note the log scale on the y-axis. Error bars show 95% confidence intervals. **B**, A comparison of relative variant rates between mothers and fathers. The rate ratios, their 95% CI (error bars) and *p* values were obtained from binomial tests comparing variant counts in the mothers and fathers, and using the synonymous variant counts in mothers and fathers to derive the expected rate (hence, the rate ratio of synonymous variants is 1).

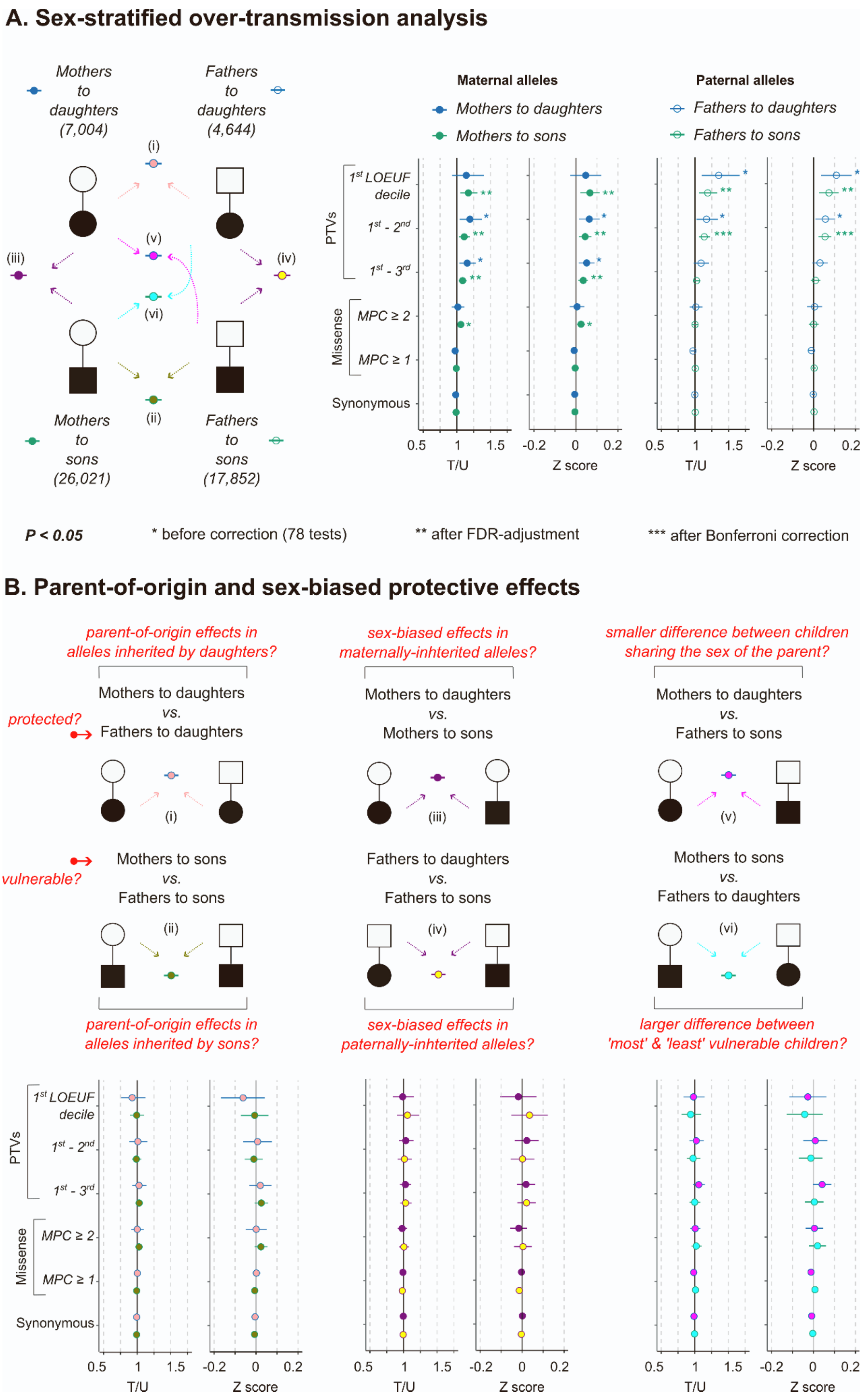


Figure S16: Parent-of-origin and sex-biased protective effects in inherited ultra-rare variants.

The transmission ratios (T/U) and effect sizes on the liability scale (Z score) of maternally-inherited and paternally-inherited alleles were examined separately in sons and daughters (**A**). This mega-analysis was performed across all child-parent pairs from trio- and duo-sequenced children in

ASC and SPARK (the number of child-parent pairs is given in schematic on the left-hand side). In this test, T/U ratio > 1 or Z score > 0 means there is evidence for over-transmission from a comparison of transmitted to untransmitted alleles. The effect sizes (on the observed and liability scales) were used to test for parent-of-origin effects and sex-biased vulnerability to damaging maternal or paternal alleles (**B)**. Six pairwise tests were performed (numbered i-v and color-coded similarly in **A** and **B**). The schematics on the top of each forest plot in **B** indicate the tested alleles and summarizes the question it attempts to answer; the effect size is given for the allele on the left-hand side in the schematic relative to the allele on the right-hand side; e.g., in test 'vi', a T/U ratio > 1 would mean that maternal alleles show more over-transmission to sons (hypothesized to be 'vulnerable') relative to paternal alleles transmitted to daughters (hypothesized to be 'protected'). In all panels, p values were obtained from binomial tests; Error bars show 95% confidence intervals.

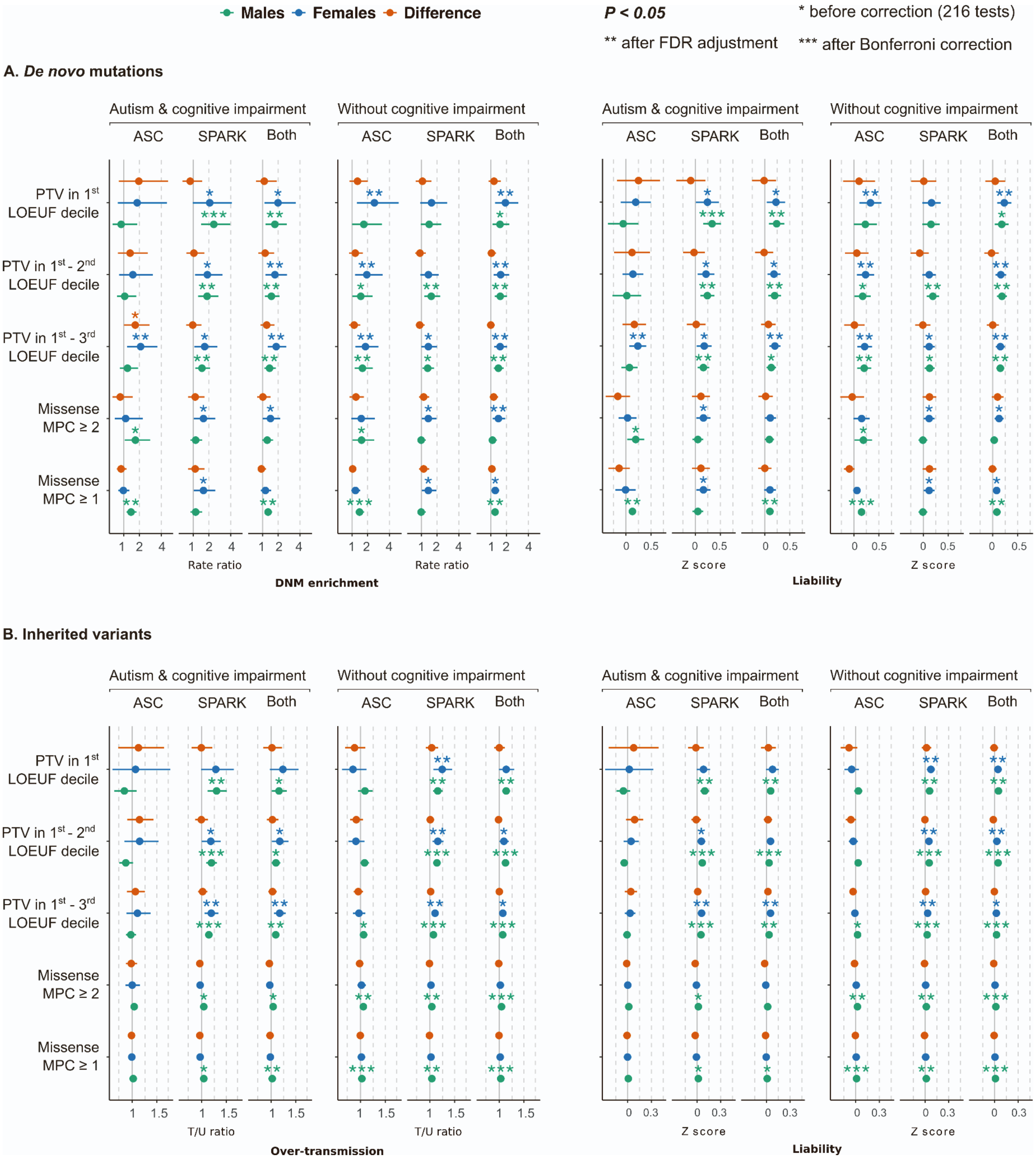


Figure S17: Exome-wide enrichment of *de novo* and rare variants after excluding high-confidence and syndromic autism risk genes amongst individuals with and without cognitive impairment.

**A**, Sex-stratified *de novo* mutation rate ratios (left) and liability (right) calculated after excluding SFARI genes. **B**, Over-transmission of rare damaging variants (left) and the liability conveyed by inherited alleles (right) in these phenotypic groups. The enrichment and liability was calculated separately in those with or without cognitive impairment (including those with unknown status). See Figure 3 for the sample sizes. For sex differences, a rate ratio > 1 indicates that females show a higher enrichment; a Z score > 0 indicates that females show a higher effect size on the liability scale. Error bars show 95% confidence intervals.

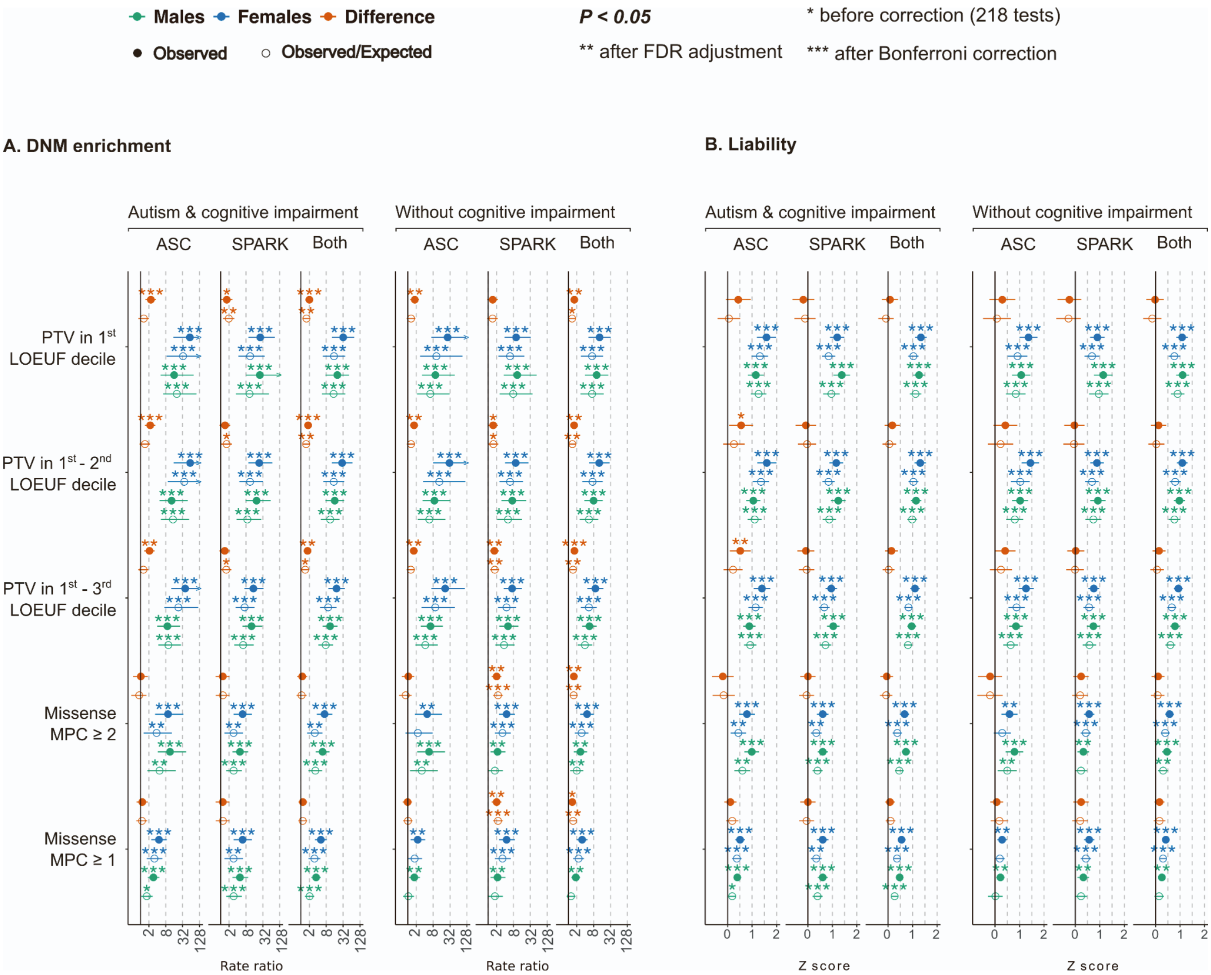


Figure S18: Meta-analysis of the burden of *de novo* mutations in SFARI genes in autistic individuals with and without cognitive impairment in ASC and SPARK.

Sex-stratified *de novo* mutation rate ratios (**A**) and liability (**B**) in SFARI high-confidence and syndromic autism risk genes calculated separately in those with or without cognitive impairment (including those with unknown status). Additional comparisons were performed to compare the observed burden and effect sizes to what is expected for a matched gene set ('Observed/Expected'). For sex differences, a rate ratio > 1 indicates that females show a higher enrichment; a Z score > 0 indicates that females show a higher effect size on the liability scale. Error bars show 95% confidence intervals.

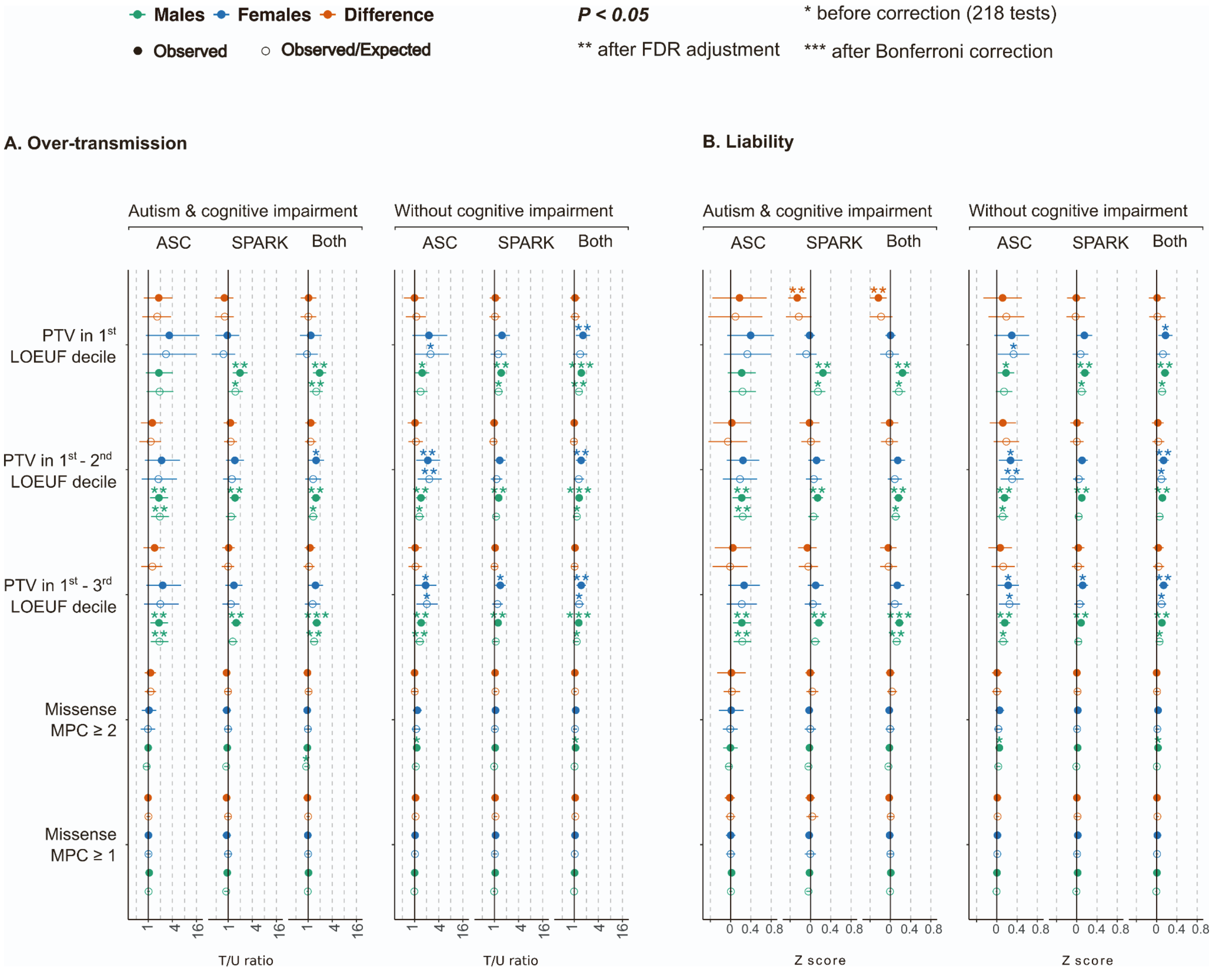


Figure S19: Meta-analysis of the burden of rare inherited variants in SFARI genes in autistic individuals with cognitive impairment in ASC and SPARK.

Over-transmission of rare damaging variants (**A**) in SFARI high-confidence and syndromic autism risk genes and the liability conveyed by inherited alleles (**B**) calculated separately in those with or without cognitive impairment (including those with unknown status). Additional comparisons were performed to compare the observed transmission rates and effect sizes to what is expected for a matched gene set (‘Observed/Expected’). For sex differences, a transmission ratio > 1 indicates that females show a higher over-transmission; a Z score > 0 indicates that females show a higher effect size on the liability scale. Error bars show 95% confidence intervals.

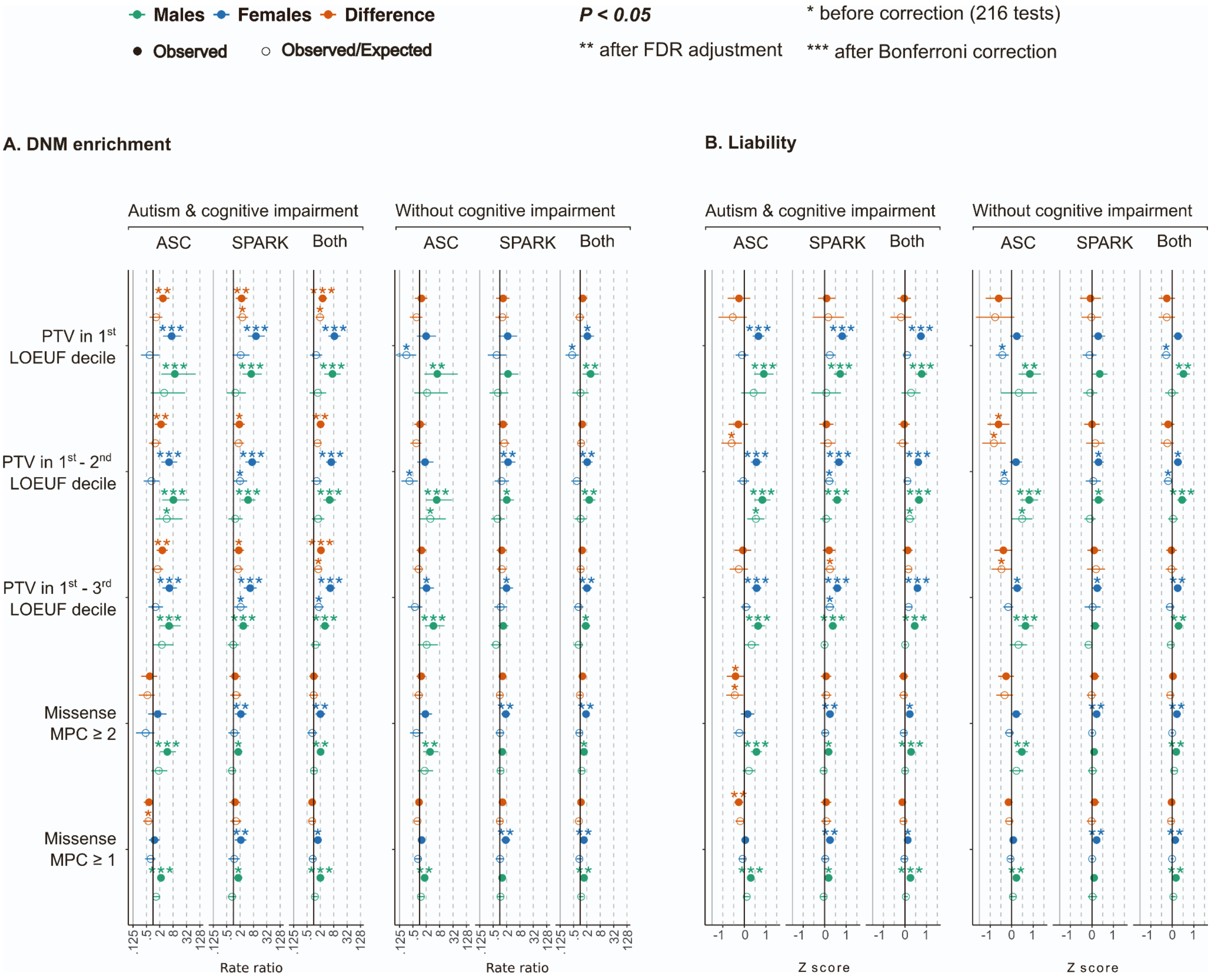


Figure S20: Burden and liability of *de novo* mutations in autosomal protein-coding genes showing male-biased expression in the human adult cortex.

**A**, Enrichment of damaging *de novo* mutations in a set of 2,852 sex-differentially expressed protein-coding genes with higher expression in males compared to females in two RNA-seq datasets (483 genes in the 1st LOEUF decile, 443 in the 2nd decile, 416 in the 3rd decile; see section 7.1 of the Supplemental Methods). The enrichment was calculated separately in those with or without cognitive impairment (including those with unknown status). Additional comparisons were performed to compare the observed *de novo* rates and effect sizes to what is expected for a matched gene set ('Observed/Expected'). **B**, The liability conveyed by *de novo* mutations in this gene set. Error bars show 95% confidence intervals. For sex differences, a rate ratio > 1 indicates that females show a higher burden; a Z score > 0 indicates that females show a higher effect size on the liability scale.

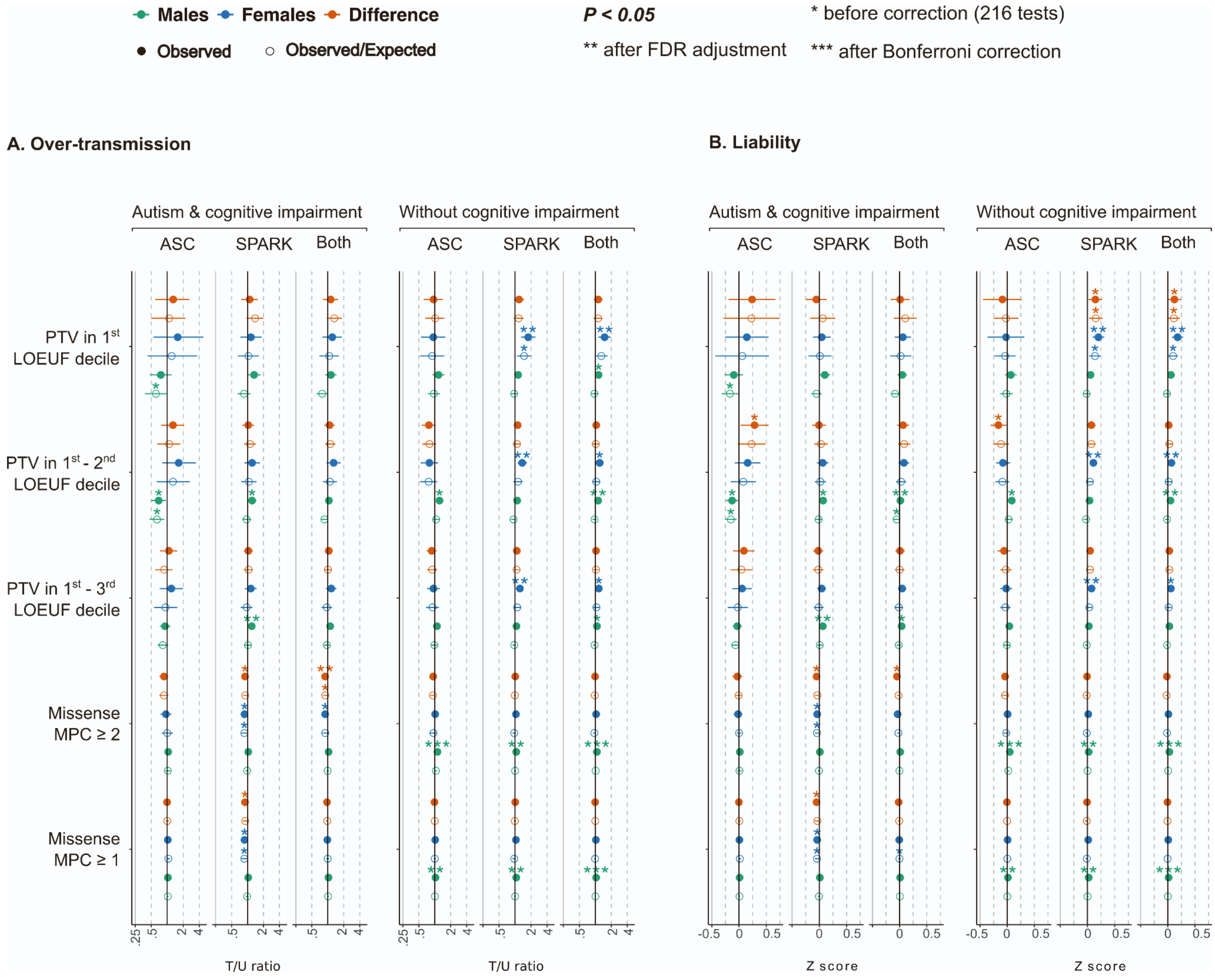


Figure S21: Over-transmission and liability of rare inherited variants in autosomal protein-coding genes showing male-biased expression in the human adult cortex.

**A**, Over-transmission of rare damaging variants in a set of 2,852 sex-differentially expressed protein-coding genes with higher expression in males compared to females in two RNA-seq datasets (483 genes in the 1st LOEUF decile, 443 in the 2nd decile, 416 in the 3rd decile; see section 7.1 of the Supplemental Methods). The enrichment was calculated separately in those with or without cognitive impairment (including those with unknown status). Additional comparisons were performed to compare the observed transmission rates and effect sizes to what is expected for a matched gene set ('Observed/Expected'). **B**, The liability conveyed by inherited alleles in this gene set. Error bars show 95% confidence intervals. For sex differences, a transmission ratio > 1 indicates that females show a higher over-transmission; a Z score > 0 indicates that females show a higher effect size on the liability scale.

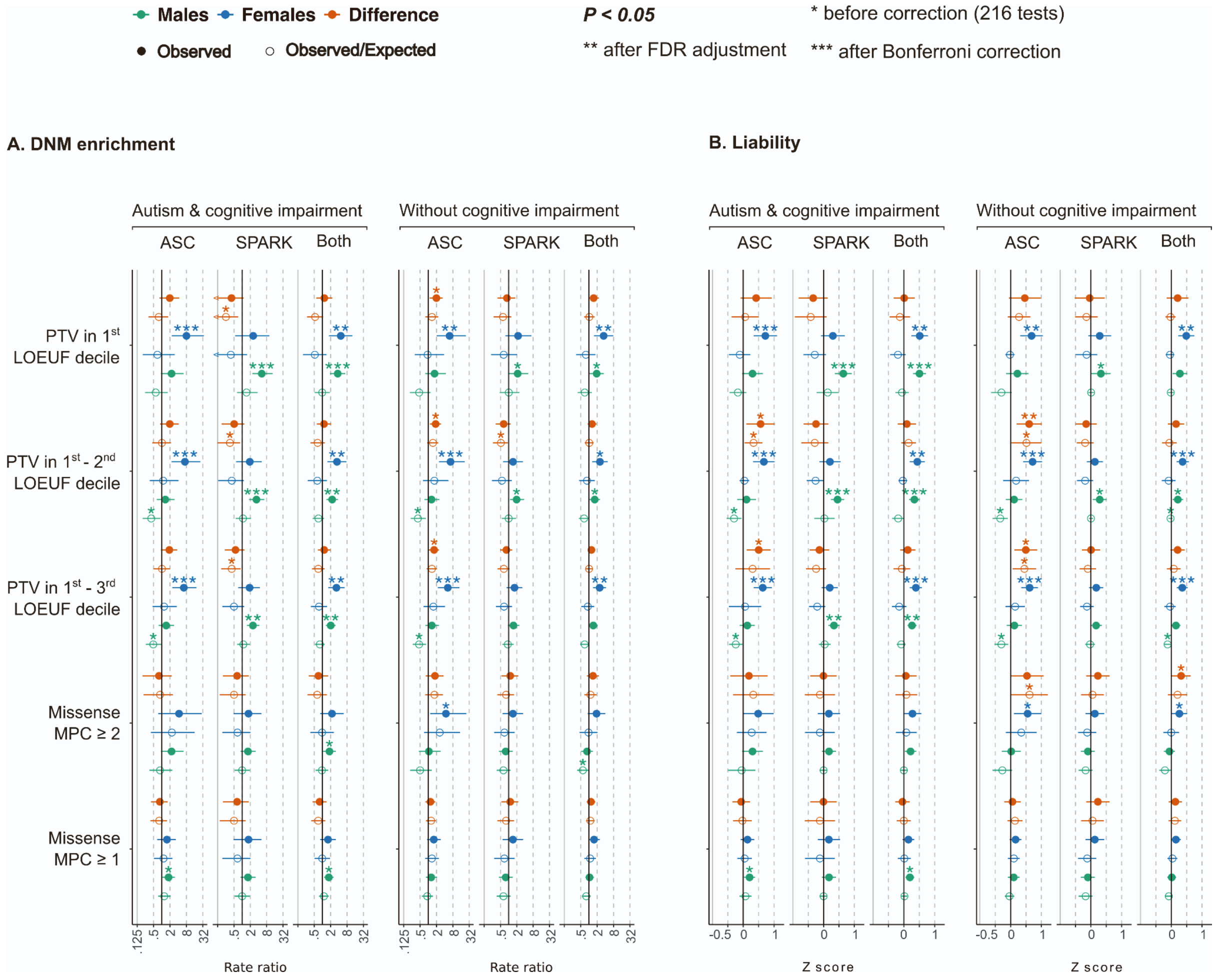


Figure S22: Burden and liability of *de novo* mutations in autosomal protein-coding genes showing female-biased expression in the human adult cortex.

**A**, Enrichment of damaging *de novo* mutations in a set of 2,427 sex-differentially expressed protein-coding genes with higher expression in females compared to males in two RNA-seq datasets (381 genes in the 1st LOEUF decile, 344 in the 2nd decile, 319 in the 3rd decile; see section 7.1 of the Supplemental Methods). The enrichment was calculated separately in those with or without cognitive impairment (including those with unknown status). Additional comparisons were performed to compare the observed *de novo* rates and effect sizes to what is expected for a matched gene set ('Observed/Expected'). **B**, The liability conveyed by *de novo* mutations in this gene set. Error bars show 95% confidence intervals. For sex differences, a rate ratio > 1 indicates that females show a higher burden; a Z score > 0 indicates that females show a higher effect size on the liability scale.

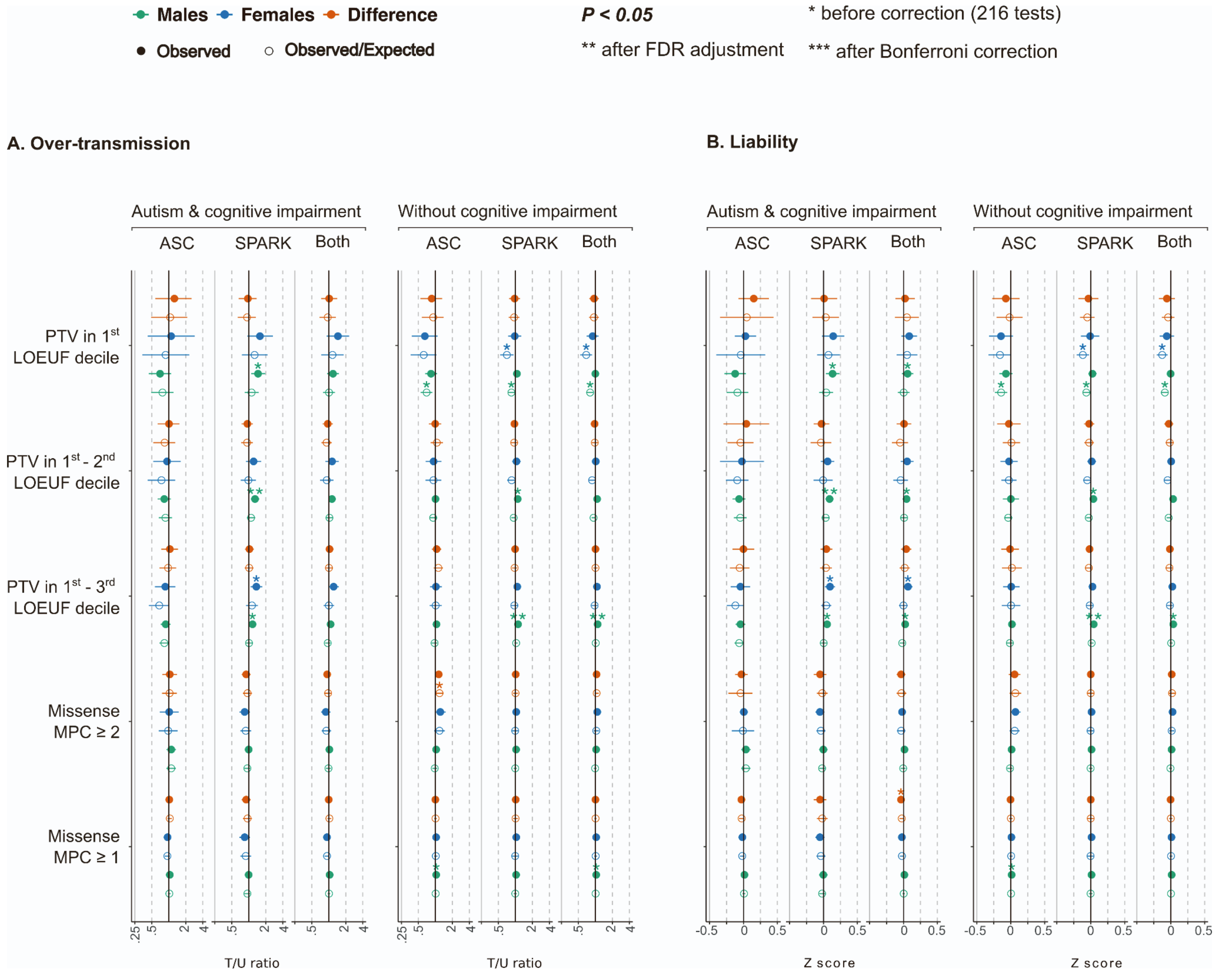


Figure S23: Over-transmission and liability of rare inherited variants in autosomal protein-coding genes showing female-biased expression in the human adult cortex.

**A**, Over-transmission of rare damaging variants in a set of 2,427 sex-differentially expressed protein-coding genes with higher expression in females compared to males in two RNA-seq datasets (381 genes in the 1st LOEUF decile, 344 in the 2nd decile, 319 in the 3rd decile; see section 7.1 of the Supplemental Methods). The enrichment was calculated separately in those with or without cognitive impairment (including those with unknown status). Additional comparisons were performed to compare the observed transmission rates and effect sizes to what is expected for a matched gene set ('Observed/Expected'). **B**, The liability conveyed by inherited alleles in this gene set. Error bars show 95% confidence intervals. For sex differences, a transmission ratio > 1 indicates that females show a higher over-transmission; a Z score > 0 indicates that females show a higher effect size on the liability scale.

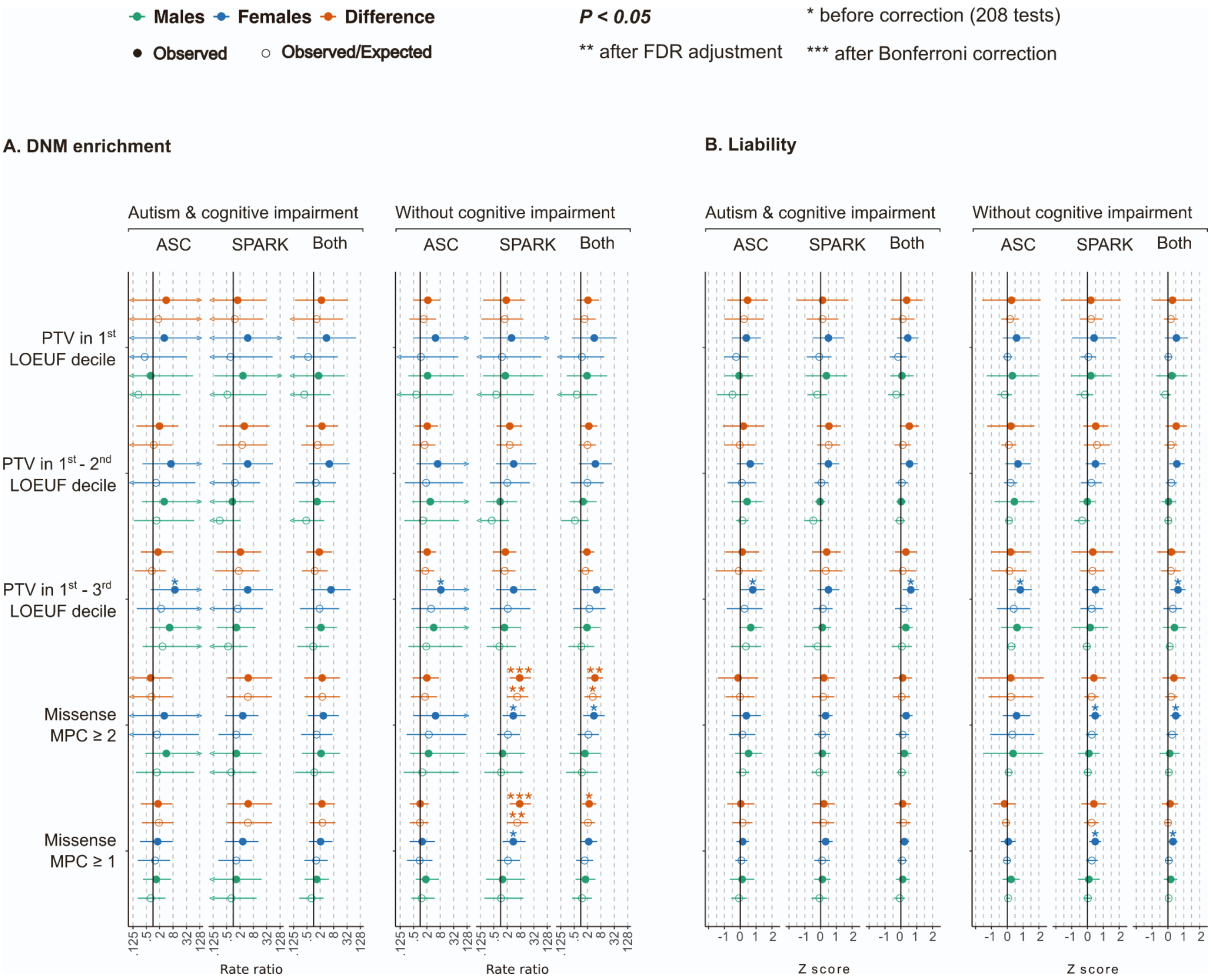


Figure S24: Burden and liability of *de novo* mutations in autosomal protein-coding genes showing male-biased expression in the human fetal cortex.

**A**, Enrichment of damaging *de novo* mutations in a set of 305 sex-differentially expressed protein-coding genes with higher expression in males compared to females in two RNA-seq datasets (39 genes in the 1st LOEUF decile, 49 in the 2nd decile, 42 in the 3rd decile; see section 7.1 of the Supplemental Methods). The enrichment was calculated separately in those with or without cognitive impairment (including those with unknown status). Additional comparisons were performed to compare the observed *de novo* rates and effect sizes to what is expected for a matched gene set ('Observed/Expected'). **B**, The liability conveyed by *de novo* mutations in this gene set. Error bars show 95% confidence intervals. For sex differences, a rate ratio > 1 indicates that females show a higher burden; a Z score > 0 indicates that females show a higher effect size on the liability scale.

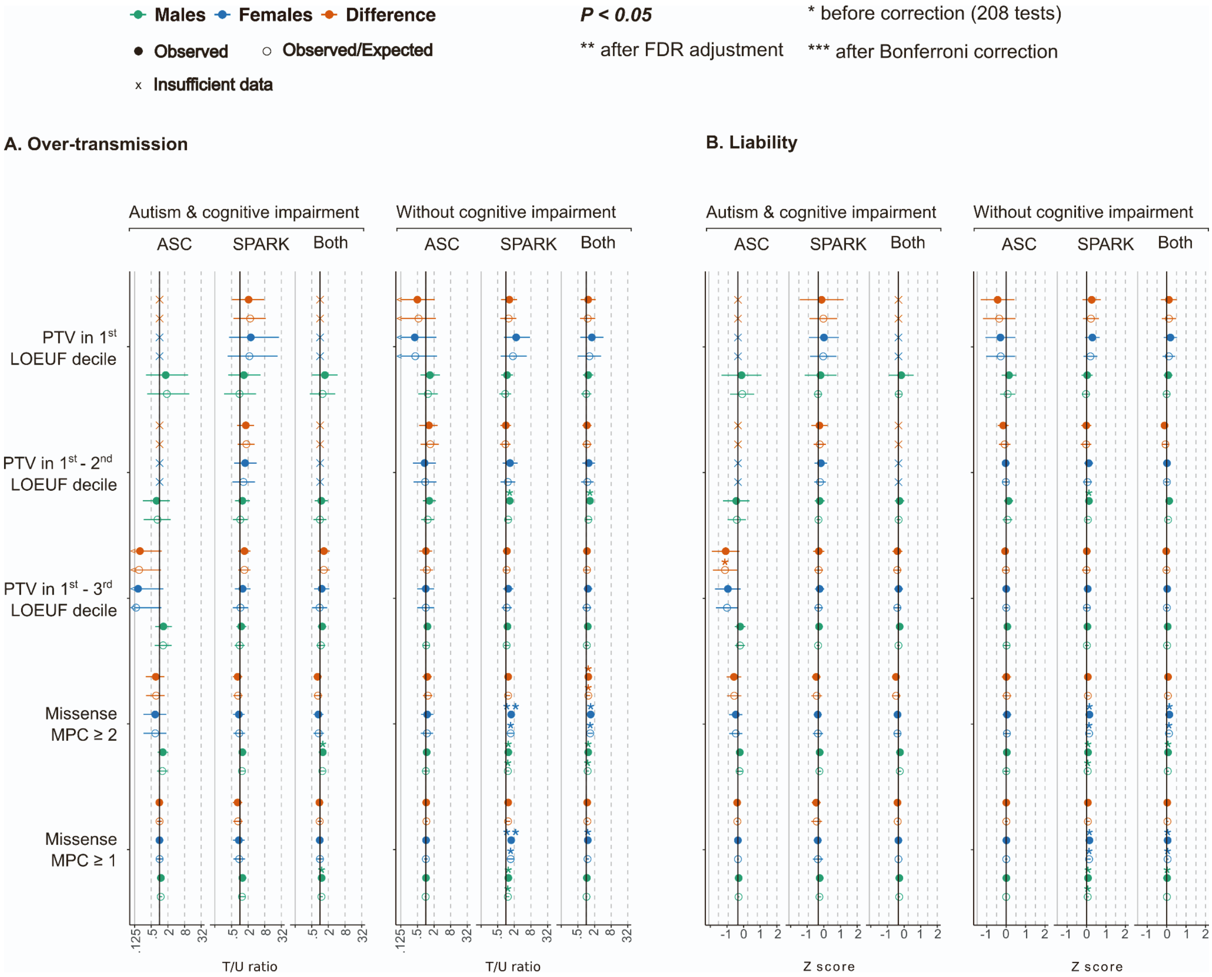


Figure S25: Over-transmission and liability of rare inherited variants in autosomal protein-coding genes showing male-biased expression in the human fetal cortex.

**A**, Over-transmission of rare damaging variants in a set of 305 sex-differentially expressed protein-coding genes with higher expression in males compared to females in two RNA-seq datasets (39 genes in the $1^{st}$ LOEUF decile, 49 in the $2^{nd}$ decile, 42 in the $3^{rd}$ decile; see section 7.1 of the Supplemental Methods). The enrichment was calculated separately in those with or without cognitive impairment (including those with unknown status). Additional comparisons were performed to compare the observed transmission rates and effect sizes to what is expected for a matched gene set ('Observed/Expected'). **B**, The liability conveyed by inherited alleles in this gene set. Error bars show 95% confidence intervals. For sex differences, a transmission ratio > 1 indicates that females show a higher over-transmission; a Z score > 0 indicates that females show a higher effect size on the liability scale.

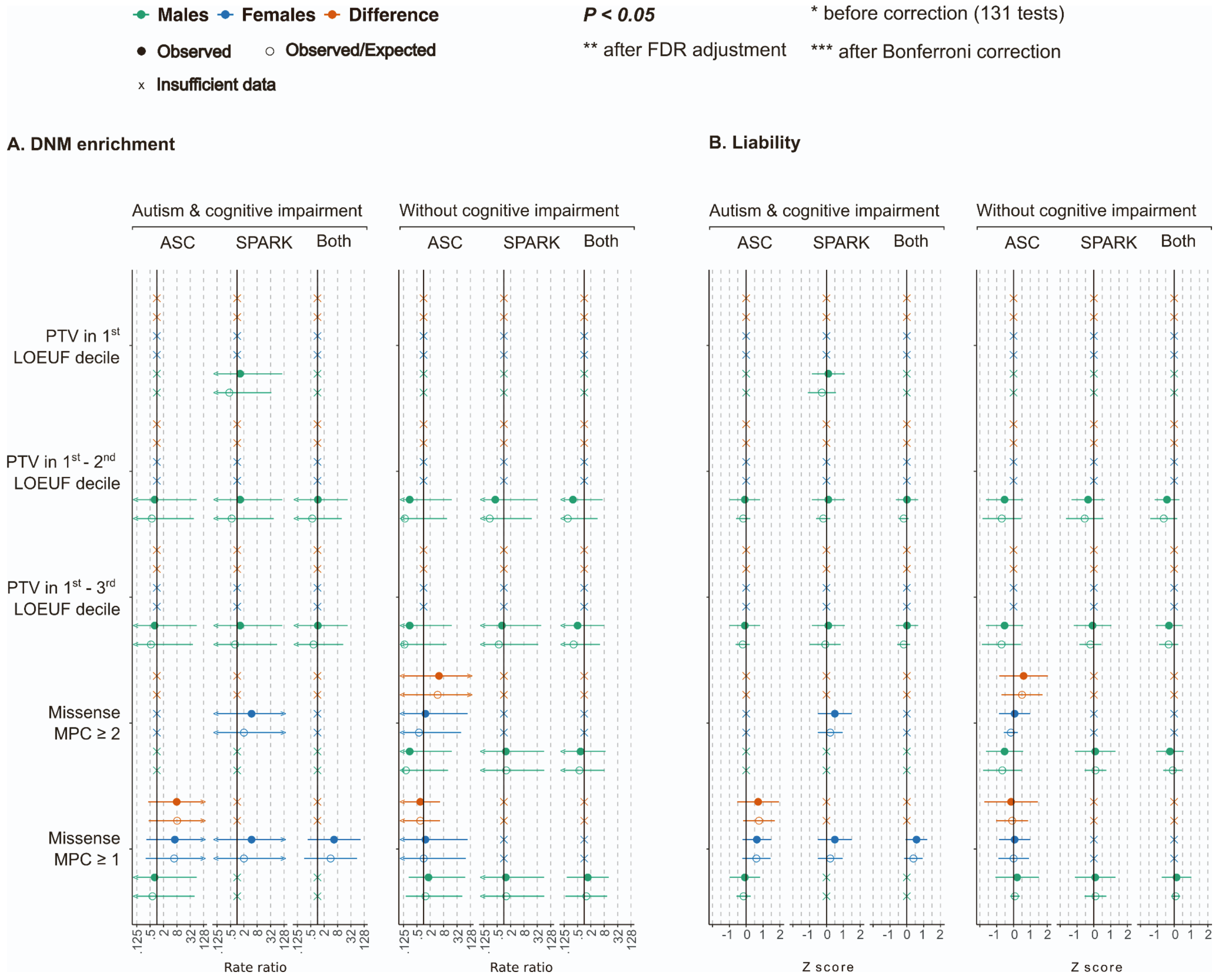


Figure S26: Burden and liability of *de novo* mutations in autosomal protein-coding genes showing female-biased expression in the human fetal cortex.

**A**, Enrichment of damaging *de novo* mutations in a set of 117 sex-differentially expressed protein-coding genes with higher expression in females compared to males in two RNA-seq datasets (8 genes in the 1st LOEUF decile, 15 in the 2nd decile, 11 in the 3rd decile; see section 7.1 of the Supplemental Methods). The enrichment was calculated separately in those with or without cognitive impairment (including those with unknown status). Additional comparisons were performed to compare the observed *de novo* rates and effect sizes to what is expected for a matched gene set (‘Observed/Expected’). **B**, The liability conveyed by *de novo* mutations in this gene set. Error bars show 95% confidence intervals. For sex differences, a rate ratio > 1 indicates that females show a higher burden; a Z score > 0 indicates that females show a higher effect size on the liability scale. These analyses were underpowered and most did not have sufficient data (no variants in probands, siblings or both; marked with ‘x’).

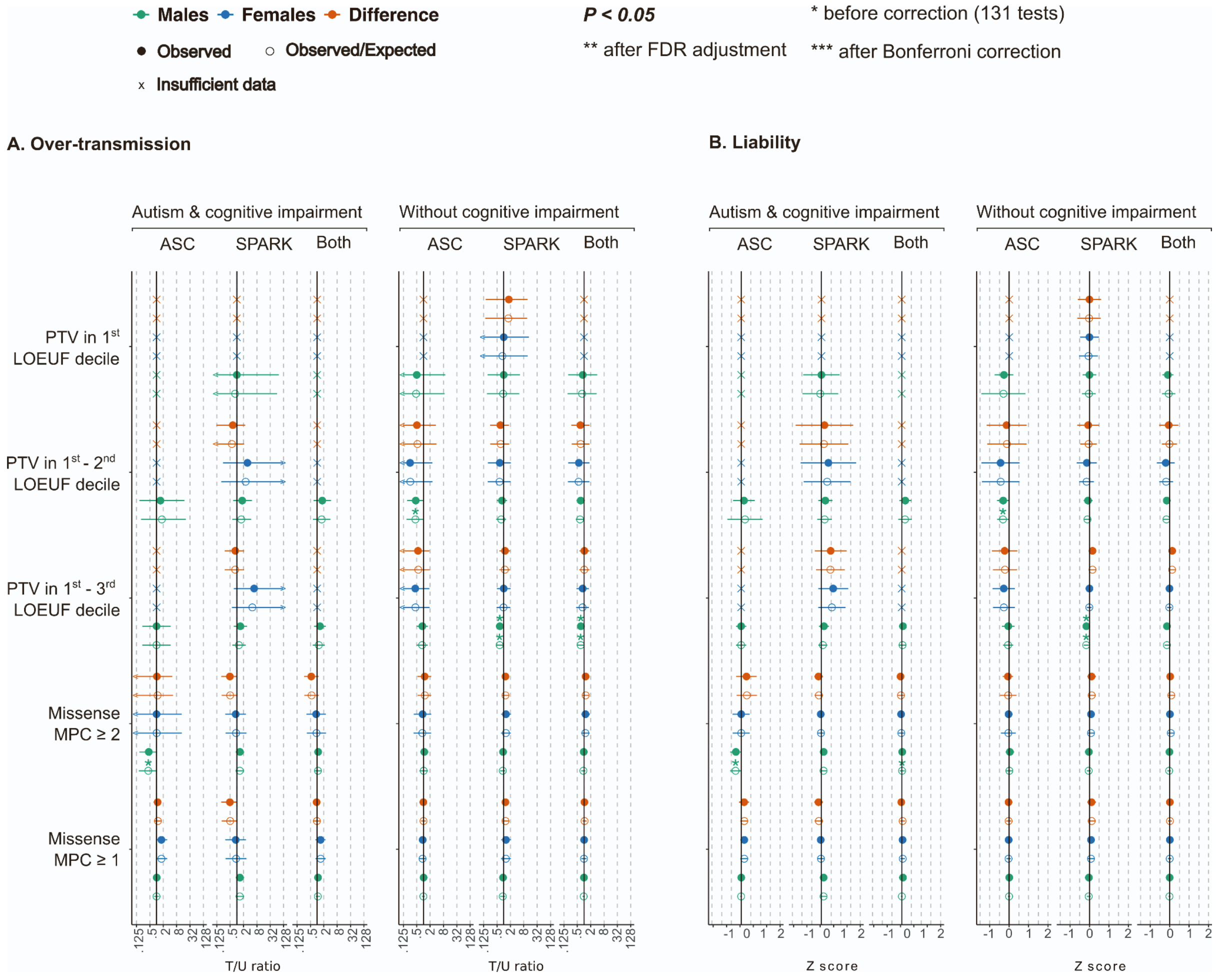


Figure S27: Over-transmission and liability of rare inherited variants in autosomal protein-coding genes showing female-biased expression in the human fetal cortex.

**A**, Over-transmission of rare damaging variants in a set of 117 sex-differentially expressed protein-coding genes with higher expression in females compared to males in two RNA-seq datasets (8 genes in the 1st LOEUF decile, 15 in the 2nd decile, 11 in the 3rd decile; see section 7.1 of the Supplemental Methods). The enrichment was calculated separately in those with or without cognitive impairment (including those with unknown status). Additional comparisons were performed to compare the observed transmission rates and effect sizes to what is expected for a matched gene set ('Observed/Expected'). **B**, The liability conveyed by inherited alleles in this gene set. Error bars show 95% confidence intervals. For sex differences, a transmission ratio > 1 indicates that females show a higher over-transmission; a Z score > 0 indicates that females show a higher effect size on the liability scale.

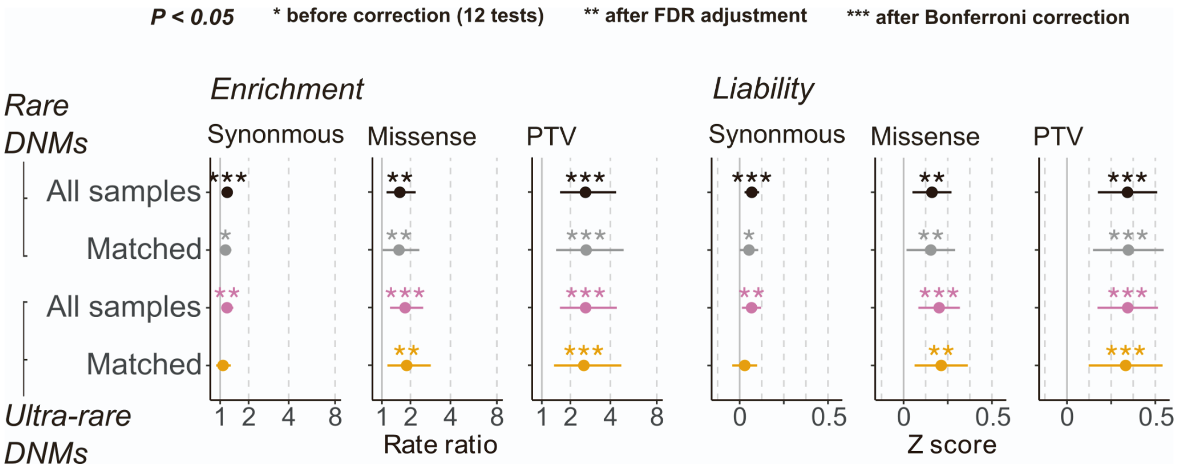


Figure S28: Enrichment of *de novo* mutations in ancestry-matched subset of autistic females without cognitive or motor impairment and siblings.

Enrichment and liability in 'All samples' was tested in 1,464 autistic females without motor or cognitive impairment and sex-matched siblings from different genetic ancestry groups (see Table S4). 'Matched' indicates comparisons between 868 female probands of European ancestry and 2,235 ancestry-matched siblings (see Supplemental Methods section 3.4).

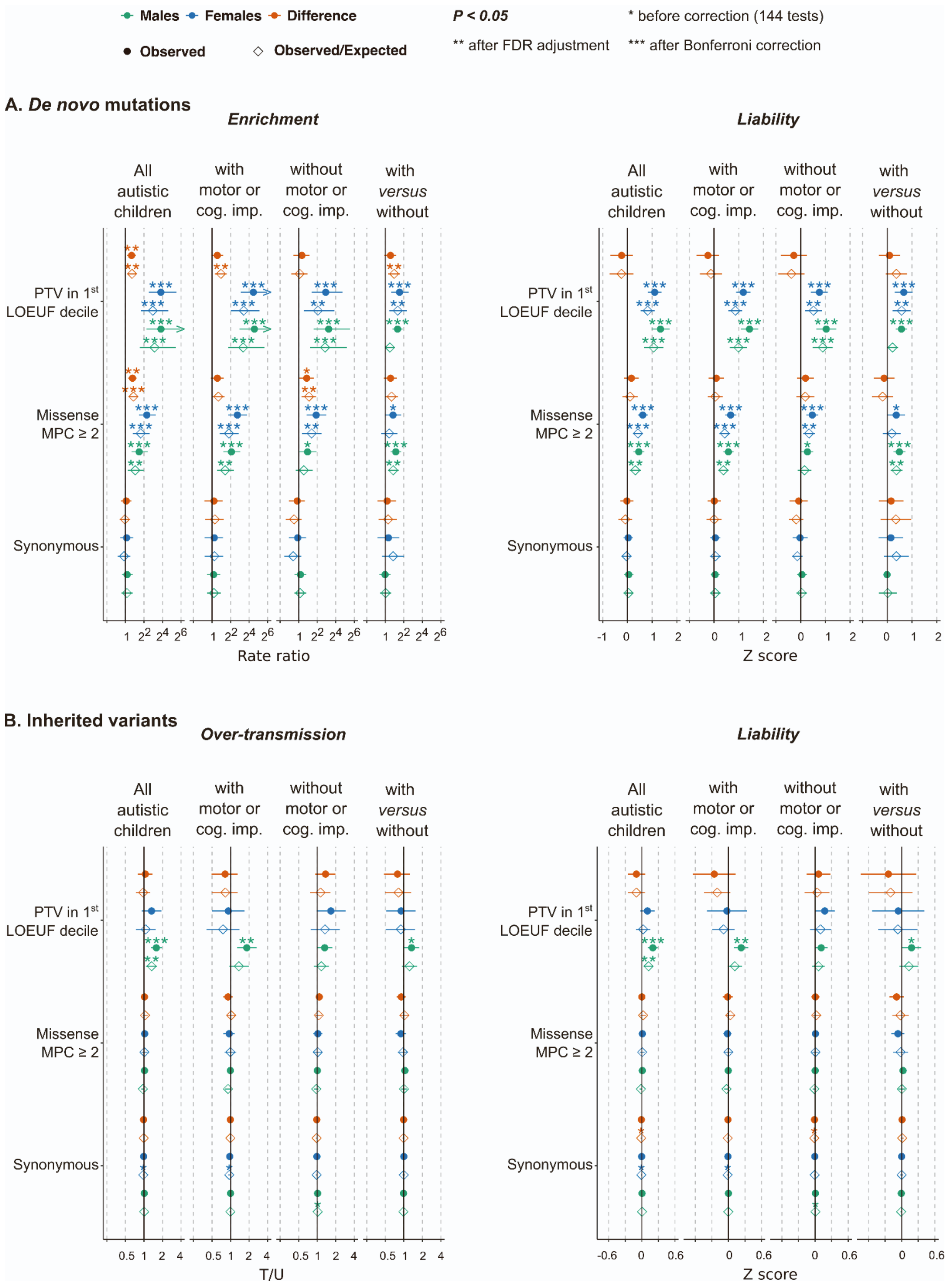


Figure S29: Enrichment of *de novo* mutations and rare inherited in 354 SFARI genes in autistic individuals with and without motor and cognitive impairment in SPARK trios.

Enrichment analysis of *de novo* mutations (DNM) (**A**) and rare variant over-transmission analysis (**B**) in SFARI high-confidence and syndromic genes in all SPARK trios, two SPARK sub-cohorts of autistic individuals ascertained to have autism with or without co-occurring developmental delay or cognitive impairment (*versus* siblings), and a direct comparison between these two groups. The rate ratio (DNMs) and transmitted/untransmitted alleles ratio (inherited variants) are shown on the left-hand side (i.e. observed scale); Average variant liability attributed to these variants is shown on the right-hand side. The enrichment and liability was also compared to a set of matched genes selected to have similar coding length, LoF-constraint, and brain expression.

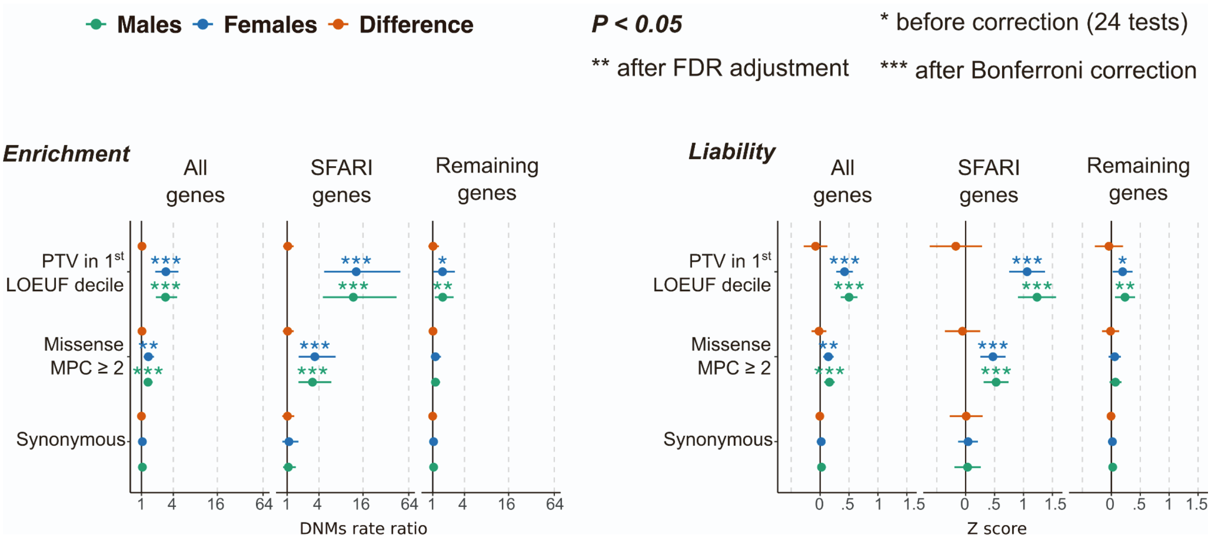


Figure S30: Permutation analysis in SPARK.

Each trio-sequenced sample was randomly assigned to the males or females cohort. This was performed within each phenotypic group in SPARK (autism with co-existing motor or cognitive difficulties, without co-occurring difficulties, unknown status, siblings) to keep the proportion of autistic individuals with or without motor or cognitive impairment constant. The enrichment and liability shown here were then calculated across all autistic individuals.

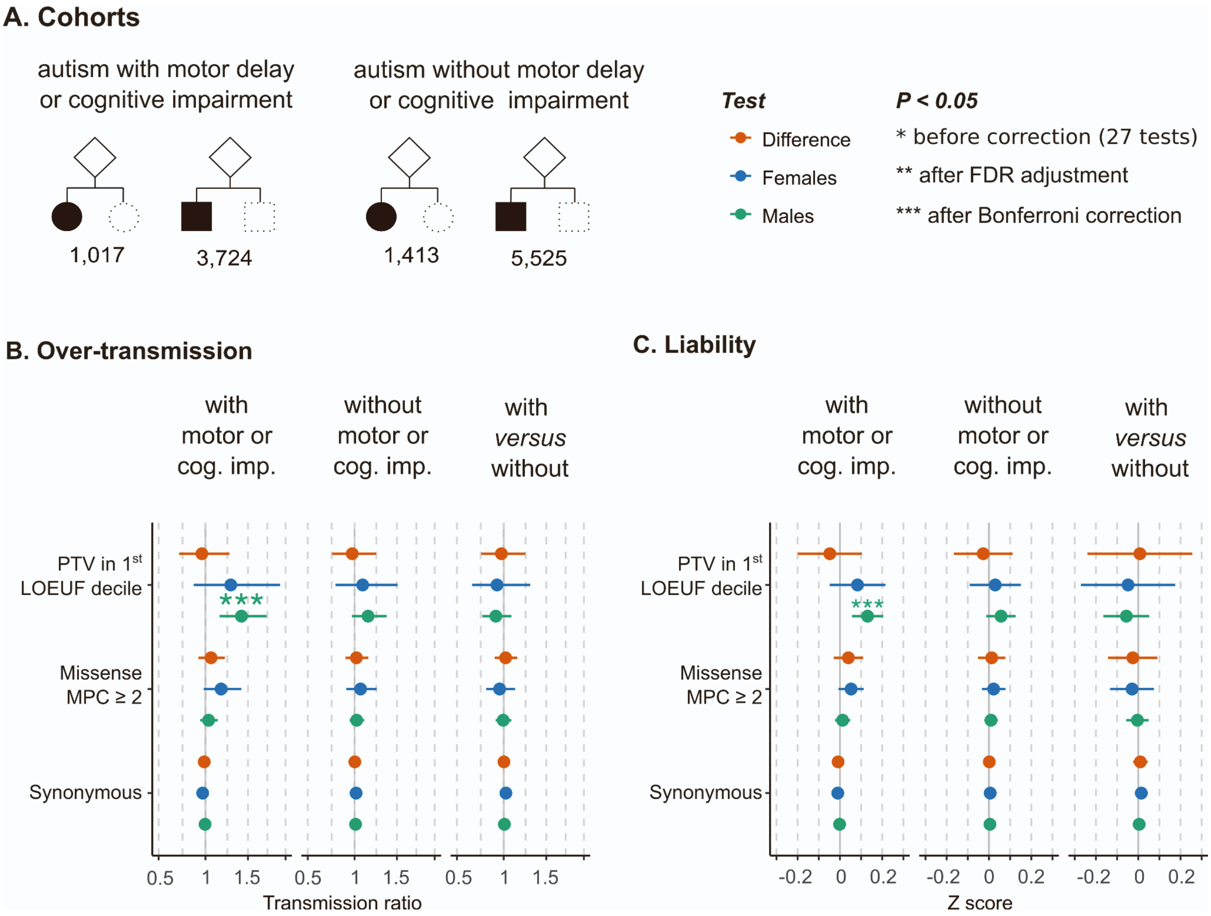


Figure S31: Over-transmission of ultra-rare inherited variants in SPARK individuals with one sequenced parent, stratified by motor and cognitive impairment.

**A**, The sample size of the different cohorts included in this analysis (note that the transmission analysis is done in probands only). **B**, Over-transmission analysis showing the ratio between rare parental alleles transmitted to autistic individuals with or without motor or cognitive impairment and untransmitted alleles. Transmission ratios were also compared between these two groups (see the Methods). **C**, The liability attributable to inherited rare variants. Related to the analysis presented in Figure S13A (‘SPARK Duos’).

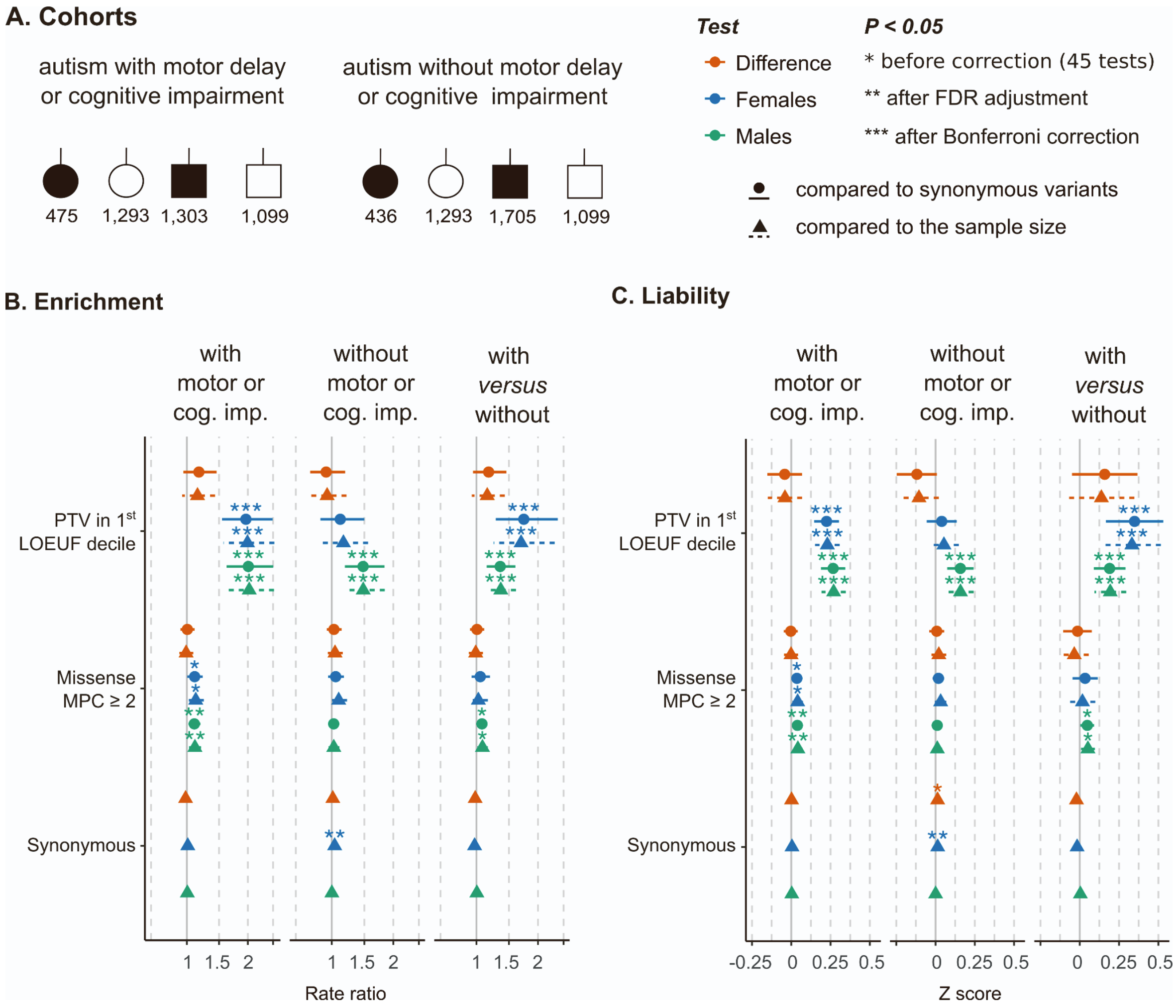


Figure S32: Enrichment of ultra-rare variants in SPARK individuals without sequenced parents, stratified by motor and cognitive impairment.

The enrichment of ultra-rare variants in a cohort of autistic probands without parental sequence data in SPARK (**A**) was examined on the observed scale (**B**) and liability scale (**C**). Variant rates were compared between autistic stratified by co-occurring cognitive or motor impairment and siblings not diagnosed with autism as well as between the two autism sub-cohorts ('with *versus* without') . The error bars show the 95% confidence intervals of the effect sizes. The enrichment and liability were assessed relative to the sample size of the case/control cohorts (i.e. assuming that the expected rare variant burden per sample is similar across cohorts). To account for the differences in ultra-rare variant counts arising from the differences in ancestry, damaging missense and protein truncating variants were also compared to the expected rate ratio from synonymous variants (i.e. normalizing the average variant rates using synonymous variant counts). This analysis is related to the analysis presented in Figure S14.

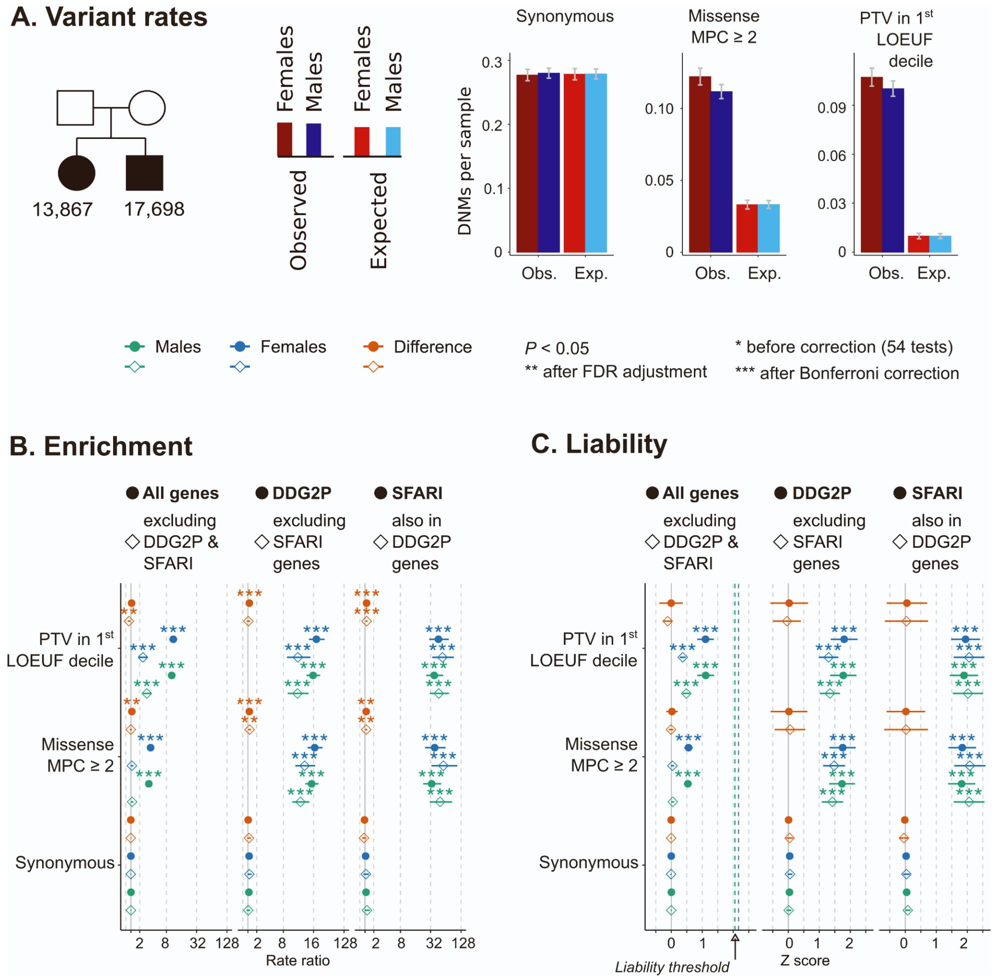


Figure S33: Enrichment and liability of damaging *de novo* mutations in 31,565 children diagnosed with neurodevelopmental disorders.

The observed *de novo* mutation rates in these trios were compared to the expected rates from a mutational model (**A**). The enrichment Sex-stratified *de novo* mutation rate ratios (**B**) and liability (**C**) were calculated for 17,296 autosomal protein-coding genes (1,742 in the 1st LOEUF decile), 845 mono-allelic DDG2P genes associated with neurodevelopmental conditions (384 in the 1st LOEUF decile), and 354 SFARI high-confidence and syndromic autism-predisposition genes (218 in the 1st LOEUF decile). The analysis was also performed in 15,032 autosomal protein-coding gene excluding DDG2P & SFARI genes (1,315 in the 1st LOEUF decile), 605 DDG2P genes excluding SFARI genes (209 in the 1st LOEUF decile), and 240 shared genes between SFARI & DDG2P genes (173 in the 1st LOEUF decile). For sex differences, a rate ratio > 1 indicates that females show a higher enrichment than males; a Z score > 0 indicates that females show a higher effect size on the liability scale. Error bars show 95% confidence intervals.

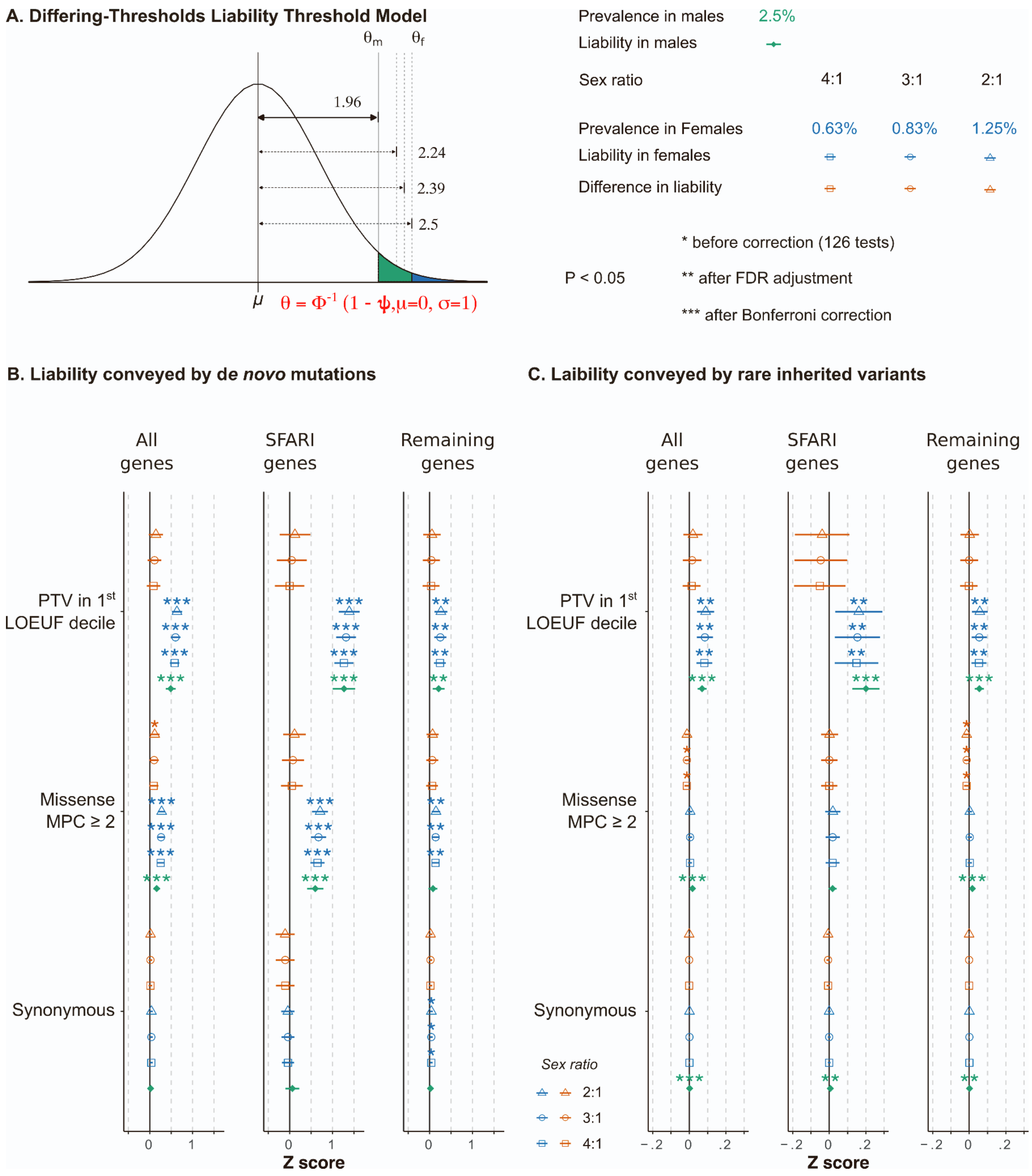


Figure S34: Testing different autism prevalences in females.

The 'standard' Liability Threshold Model (LTM) posits that the genetic liability to autism in the population is normally distributed with equal means (μ) in both sexes and a standard deviation (σ) of 1 (**A**). Since the threshold (θ) determines the prevalence, it can be (back-)calculated from the observed population prevalence using the inverse cumulative density function of the normal distribution ($\Phi^{-1}$). In the Differing-Thresholds LTM, the sex difference in autism prevalence is attributed to females having a higher threshold ($\theta_f$) than males ($\theta_m$). Autism prevalence in males is estimated to be ~2.5%, whereas prevalence estimates in females are less certain (due to e.g., relative rarity or under-diagnosis in females). In the primary analysis (Figure 1), we assumed a prevalence of 0.625% in females (sex ratio of 4:1); Here, we tested higher prevalence estimates (ratios of 3:1 and 2:1). The liability attributed to damaging variants was comparable across a range of autism prevalences in females, both for *de novo* mutations (**B**) and rare inherited variants (**C**). This figure shows a meta-analysis between ASC and SPARK.

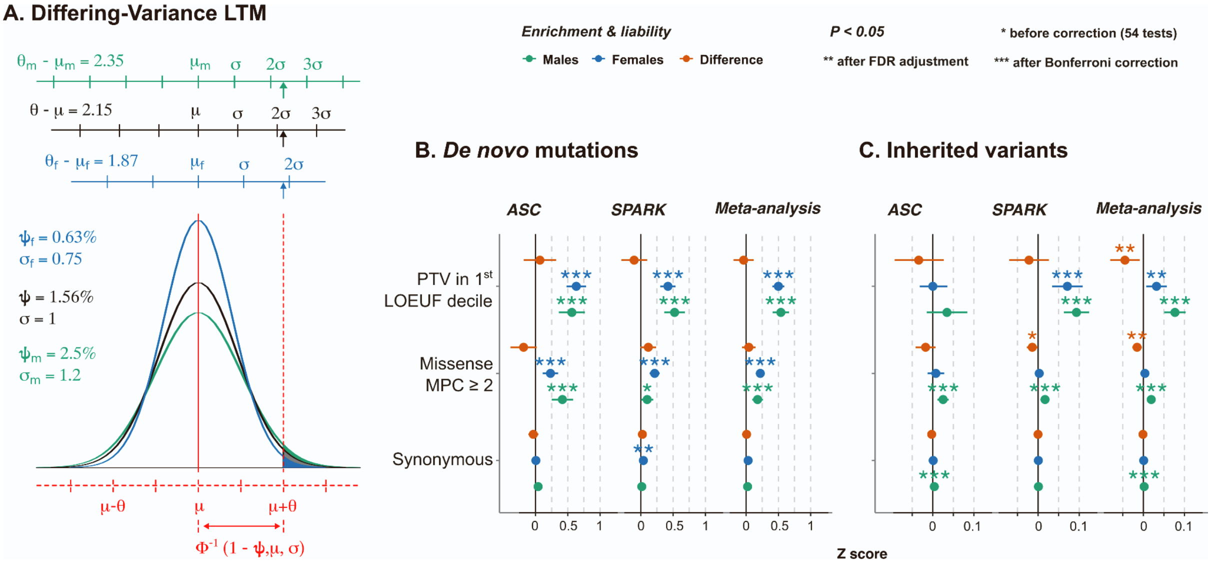


Figure S35: Sex differences in the effect size of de novo and rare inherited variants under different-variances Liability Threshold Model.

Sex differences in autism prevalence can be attributed to the two sexes having differing variances instead of differing thresholds as explained in Figure S4. **A**, An alternative Liability Threshold Model (LTM) which assumes that the liability in the population is normally distributed with a standard deviation (σ) of 1 and equal means (μ=0) in both sexes but higher variance in males ($\sigma_m$ = 1.2) than females ($\sigma_f$ = 0.75). Note that the parameters were chosen as a demonstration (the true variance of autism liability in the population is unknown). **B**, The liability conveyed by *de novo* and rare inherited variants under this differing variance model. The error bars indicate the 95% confidence intervals. The effect sizes are shown on the population scale (σ = 1, μ = 0). See section 6.2 of the Supplemental Methods for more details on calculating the effect sizes.

# Supplemental Results

## 1. DNM and inherited variant rates

We processed exome sequencing data from autism probands, siblings and parents from the second integrated release of whole exome sequencing data (WES) from the Simons Powering Autism Research for Knowledge (SPARK iWES2) study and combined these data with data from the SSC and other smaller cohorts previously curated by the ASC. An overview of all analyses is presented in Figure S1. Here we describe the observed variant rates in the study population.

The curation of *de novo* and rare inherited variants is described in section 3 of the Supplemental Methods. The *de novo* mutation rates observed in the probands and siblings in the currently analyzed SPARK release (iWES2) were comparable to, albeit slightly lower than, the variant rates seen in the ASC cohort. We identified ~0.22 - 0.25 synonymous *de novo* mutations per child (across probands and siblings) in our analysis of SPARK iWES2 trios - slightly lower than the previous ASC analysis of SPARK Pilot/WES1 samples (first sequencing wave) which identified ~0.27 synonymous mutations per child (Figure S6). Small differences are expected given the different *de novo* calling pipelines (see section 3 of the Supplemental Methods) and these rates are within the range seen in other studies. The rate of synonymous DNMs from two different previous analyses of SPARK first sequencing wave was between ~0.20 - 0.23 per child in one study[1] and ~27 - 0.32 per child in another.[2] An integrated analysis across several studies of neurodevelopmental disorders identified between 0.2 - 0.3 synonymous mutations per trio.[3] Though damaging missense variants were defined similarly (MPC ≥ 2), we note that the ASC used a different (earlier) version of MPC scores (ExAC-based v1) than the version we used for SPARK iWES2 (gnomAD-based v2). However, the fraction of missense DNMs with MPC score ≥ 2 among siblings not diagnosed with autism was similar between ASC (4.8%) and SPARK (4.98%).

The rates of rare inherited variants (MAF < 0.1%) in SPARK iWES2 were higher than those seen in the ASC cohort and this difference was most prominent in damaging missense variants. In addition to technical differences, average rare inherited variant counts are also sensitive to population differences, partly because different ancestral groups differ in their demographic histories and hence allele frequency spectra, and also because of how the variants are filtered based on in-sample frequencies. While the ASC cohort is predominantly European (composed of the Simons Simplex Collection and other smaller cohorts; see Figure S4), SPARK has more diversity, being only 73% European ancestry in iWES2 (see

Figure S2 and Table S3). To have a more informative comparison of potential artifactual differences in processing less confounded by the differences in ancestry, we explored the variant rates in the previously analyzed SPARK cohort (Pilot and first sequencing wave WES1; 78% European ancestry), which was processed using the same pipeline as the ASC. The rates (per child) of rare inherited synonymous variants in SPARK Pilot/WES1 were also higher than in the other jointly-processed ASC cohorts but were comparable to the currently used SPARK iWES2 dataset processed differently (Figure S6). This suggests that these differences in rare variant rates are a reflection of the diverse genetic ancestry of SPARK samples more so than mere technical differences.

Ultra-rare variants (transmitted/untransmitted; case/(sib-)control) were defined as detailed in section 4 of the Supplemental Methods. To evaluate over-transmission in SPARK probands who have exome data from one parent (duos), we examined the rates of transmitted and untransmitted ultra-rare inherited variants - i.e. those seen in one parent in the dataset & absent from gnomAD. We also defined ultra-rare variants in SPARK and ASC trios in a similar manner to allow joint analysis of parent of origin effects in all child-parent pairs. Ultra-rare synonymous variant rates were comparable between those duo-sequenced children in SPARK and trio-sequenced children in SPARK and ASC with an average of ~5-6 transmitted ultra-rare synonymous alleles per child (Figure S7A). Ultra-rare 'case-control' synonymous variant rates (i.e., in those without parental sequence data; gnomAD MAF ~ 0.005%) were also comparable between the ASC case-control cohorts and SPARK probands/siblings without parental sequence data (Figure S7B).

# 2. Sex differences in autism likelihood conferred by exome-wide rare variants

Here we describe in more detail the cohort-level results shown in Figure 1 and related analyses in the remaining cohorts.

## 2.1. *De novo* mutations

### 2.1.1. Damaging protein-truncating DNMs

Damaging protein-truncating DNM rates in autistic males were three times higher than sex-matched siblings, and were comparable in SPARK and ASC; We found a 2.9 fold-enrichment in SPARK (95% CI = 2 - 4.3; $p = 2.1 \times 10^{-10}$; Bonferroni-corrected $p = 1.1 \times 10^{-8}$) and a 3.1 fold-enrichment in ASC (95% CI = 1.9 - 5.3 ; $p = 5.1 \times 10^{-8}$; Bonferroni-corrected p =

$2.8x10^{-6}$). The enrichment in females *versus* sex-matched siblings was slightly stronger than what is seen in males in SPARK (risk ratio = 3.6; 95% CI = 2.4 - 5.5; p = $4.8x10^{-12}$; Bonferroni-corrected p = $2.6x10^{-10}$) and much more pronounced in females in the ASC cohort (risk ratio = 6.4, 95% CI =3.8 - 11.6 ; p = $1.7x10^{-17}$; Bonferroni-corrected p = $9.4x10^{-16}$). The most notable sex difference was seen in *de novo* damaging PTV rates in the ASC cohort, where DNMs rates in autistic females were 1.8 times higher than autistic males (95% CI = 1.4 - 2.2; p = $5.1x10^{-7}$; Bonferroni-corrected p = $2.7x10^{-5}$). The difference in SPARK was lower (risk ratio = 1.3; 95% CI = 1 - 1.6) and significant only before correction for multiple testing (p = 0.04). SFARI genes showed significant sex differences on the observed scale. An enrichment of protein-truncating *de novo* mutations, in both sexes, was still observable when removing these known autism predisposition genes, however without significant sex differences (Figure S10A).

On the liability scale, *de novo* damaging PTVs increased the liability by 0.5 standard deviation units in SPARK - similarly for both sexes (95% CI: 0.3 - 0.6 ; Bonferroni-corrected p < $3x10^{-9}$). In the ASC cohort, they also increased the liability by 0.5 units in males (95% CI = 0.3 - 0.7; Bonferroni-corrected p = $3x10^{-7}$), and caused a larger shift in females (Z = 0.75; 95% CI = 0.6 - 0.9 ; Bonferroni-corrected p = $4.7x10^{-16}$), albeit not significantly different from males (p = 0.09). SFARI genes showed the same general patterns of protein-truncating DNM liability, with larger effect sizes. Similar patterns were observed when removing these known autism predisposition genes, with considerably lower effect sizes (Figure S10A). We note here that the discovery of SFARI genes was based, in part, on these datasets.

### 2.1.2. Damaging missense DNMs

Damaging missense mutations showed comparable enrichment in females *versus* siblings in SPARK (risk ratio = 2; 95% CI = 1.5 - 2.6 ; p = $7x10^{-8}$; Bonferroni-corrected p = $3.8x10^{-6}$) and ASC (risk ratio = 2.1; 95% CI = 1.4 - 3.1; p = 0.00013; Bonferroni-corrected p = 0.0075). The enrichment in males was slightly higher in the ASC (risk ratio = 2.4; 95% CI = 1.6 - 3.6 ; p = $8x10^{-7}$; Bonferroni-corrected p = $4.3x10^{-5}$) but considerably lower in SPARK (risk ratio = 1.2; 95% CI = 1 - 1.5 ; p = 0.04; not significant after FDR or Bonferroni correction). In terms of direct comparisons, damaging missense DNMs were slightly more frequent in autistic females *versus* autistic males in SPARK (risk ratio = 1.3 , 1.1 - 1.5; p = 0.0073; FDR-adjusted p = 0.017; FDR-adjusted p = ; Bonferroni-corrected p = 0.39) but did not show a significant sex difference in ASC (p = 0.45).

Females in both ASC and SPARK had comparable variant liability attributed to damaging missense DNMs (ASC = 0.27; 95% CI = 0.13 - 0.4; SPARK = 0.25; 95% CI = 0.16 - 0.24; Bonferroni-corrected p < 0.00044). In males, these missense DNMs showed different

estimates between ASC (Z = 0.38; 95% CI = 0.23 - 0.53) and SPARK (0.09 units; 95% CI = 0.003 - 0.18), and this difference between cohorts was significant ($Z_{Difference}$ = 0.29; 95% CI: 0.12 - 0.46; p = 0.0011). In terms of sex differences, damaging missense DNMs had similar average liability in ASC but significantly lower liability in males in SPARK ($Z_{Difference}$ = -0.16; 95% CI: -0.28 - 0.034; p = 0.013; FDR-adjusted p = 0.031; Bonferroni-corrected p = 0.70). However, these sex-stratified estimates were more congruent between males and females when examining ultra-rare DNMs (see 2.1.3 below), suggesting that the effect sizes are indeed similar. This is in line with the findings from meta-analysis, where the estimates were not significantly different between males and females (Figure 1B). As seen with protein-truncating PTVs, damaging missense DNMs in SFARI genes and the remaining autosomal genes showed similar patterns compared to that obtained with exome-wide analysis, with larger and smaller effect sizes, respectively (Figure S10).

### 2.1.3. Synonymous DNMs

We note that rare synonymous DNMs showed a small but significant liability in autistic females in SPARK (Z = 0.046, 95% CI = 0.01 - 0.082; p = 0.011; FDR-adjusted p = 0.029). This likely reflects the higher genetic diversity in autistic females (72% individuals of European genetic ancestry) compared to sex-matched siblings (75% individuals of European genetic ancestry). This imbalance, however, did not persist after meta-analyzing the two cohorts (Z = 0.036; 95% CI = 0.0055 - 0.067; p = 0.052). A stringently-defined set of ultra-rare DNMs in SPARK (allele frequency < 0.005%) showed well-balanced synonymous mutation counts between female probands and sex-matched siblings while having the same protein-truncating DNM liability (see 2.1.4 below). Therefore, it is unlikely that the meta-analyzed estimates of damaging DNM liability presented in Figure 1 are biased.

### 2.1.4. Ultra-rare *de novo* mutations in SPARK

Almost all protein-truncating DNMs and most damaging missense DNMs were ultra-rare, whereas only half of the synonymous variants were in this group (Figure S12). Consequently, the liability of ultra-rare and rare protein-truncating DNMs were similar; the liability of ultra-rare damaging missense DNMs in males (Z = 0.16; 95% CI = 0.06 - 0.25; p = 0.0015) was higher than that seen with rare missense DNMs (Z = 0.09; 95% CI = 0.003 - 0.18; p = 0.04). There was no significant sex difference in ultra-rare DNM liability (p = 0.065) in contrast to what is seen with rare DNMs (p = 0.012). Ultra-rare synonyms DNMs were not enriched in the probands *versus* siblings, nor did they show a significant sex difference.

All in all, this analysis showed a similar (PTVs) or stronger (missense) enrichment in ultra-rare damaging DNMs along with better-balanced ultra-rare synonymous DNM burden,

which suggests that the imbalance in synonymous DNMs seen in the rare variant analysis is not accompanied by an inflation of the estimates for damaging mutations particularly protein-truncating mutations, which showed equal (or rather slightly higher) risk ratios in ultra-rare DNM compared to rare DNMs.

## 2.2. Rare inherited variants

### 2.2.1. Damaging protein-truncating variants

Damaging PTVs showed evidence of over-transmission from non-autistic parents to autistic individuals in SPARK only - both to female (transmission ratio = 1.3 ; 95% CI = 1.1 - 1.4; p = $1.8 \times 10^{-4}$; Bonferroni-corrected p = $9.5 \times 10^{-3}$) and males (T/U = 1.22; 95% CI = 1.14 - 1.3; p = $3 \times 10^{-9}$; Bonferroni-corrected p = $1.6 \times 10^{-7}$). Similar to the observed over-transmission patterns, inherited damaging PTVs conveyed approximately the same liability in males (Z = 0.085 units; 95% CI = 0.057 - 0.11) and females (Z = 0.082; 95% CI = 0.04 - 0.13).

There was still a significant over-transmission and inherited liability in SPARK when excluding SFARI genes, suggesting that a substantial portion of the genes driving this association are not in the SFARI gene set. In particular, females showed significant over-transmission in the remaining genes rather than SFARI genes, whereas males showed significant over-transmission in both. In the ASC, only SFARI genes showed significant PTV over-transmisisonl, both in males and in females. These findings are shown in Figure S11.

### 2.2.2. Damaging missense variants

Damaging missense variants showed comparable over-transmission to autistic males but not to females in both cohorts; the transmission ratios were significantly higher than 1 in autistic males in ASC (T/U = 1.06; 95% CI = 1.02 - 1.09; p = 0.00033; Bonferroni-corrected p = 0.018) and in SPARK (T/U = 1.04; 95% CI = 1.02 - 1.06; p = $7.4 \times 10^{-5}$; Bonferroni-corrected p = 0.004) but not in females in both (T/U in ASC = 1.02; 95% CI = 0.96 - 1.09; p = 0.47; T/U in SPARK = 1.01; 95% CI = 0.98 - 1.04; p = 0.61). Despite this difference in sex-stratified estimates, there was no statistically significant over-transmission in autistic females when compared directly to autistic males (T/U in ASC = 0.99; 95% CI = 0.94 - 1.04; p = 0.58; T/U in SPARK = 0.99; 95% CI = 0.96 - 1.01; p = 0.32).

The cohort-level liability attributed to inherited damaging missense variants mirrored the over-transmission patterns, where it was significant in males in ASC (Z = 0.023; 95% CI = 0.010 - 0.035) and SPARK (Z = ; 95% CI = 0.015 - 0.0077) but not females, without a significant sex difference (p > 0.093). As noted in the main text, this sex difference was nominally significant in the meta-analyzed cohort (Figure 1). The liability attributed to

inherited damaging missense variants in SFARI genes did not differ significantly from the population mean. Consequently, the liability in the remaining genes mirrored that seen exome-wide (Figure S11B).

### 2.2.3. Synonymous variants

Inherited parental allele counts in SPARK were not significantly different from untransmitted alleles (Figure 1C). There was an imbalance in transmitted and untransmitted synonymous variants in autistic males in the ASC cohort (Transmitted alleles = 519,579; Untransmited = 515,406; Rate ratio = 1.0081) corresponding to a small effect size on the liability scale (Z = 0.0034; 95% CI = 0.0018 - 0.005; p = $4.12 \times 10^{-5}$; Bonferroni-corrected p = 0.0022). This difference is possibly due to the greater sensitivity to call rare variants that are transmitted (i.e. occur at least twice in the dataset) than those that are not transmitted. It was persistent after meta-analysis across cohorts but was extremely small (Z = 0.0016; 95% CI = 0.00070 - 0.0026; p = 0.0001; Bonferroni-corrected p = 0.0056) and thus unlikely to have major implications for the key conclusions.

## 2.3. Ultra-rare variants

### 2.3.1. Inherited variants in the remaining child-parent pairs in SPARK

Ultra-rare inherited PTVs ascertained in a cohort of autistic probands with sequence data from a single parent showed similar transmission ratios to females (T/U = 1.3; 95% CI = 1.05 - 0.65 ; p = 0.018; FDR-adjusted p = 0.14; Bonferroni-corrected p = 0.99) and males (T/U = 1.26; 95% CI = 1.12 - 1.43; p = $1.6 \times 10^{-4}$; FDR-adjusted p = $2.1 \times 10^{-3}$; Bonferroni-corrected p = $8.4 \times 10^{-3}$). Accordingly, these variants conveyed similar liabilities in females (Z = 0.068; 95% CI = 0.017 - 0.18) and males (Z = 0.10; 95% CI = 0.048 - 0.15). The effect size of ultra-rare PTVs were similar in trio-sequenced probands, albeit slightly less prominent in males (Figure S13A).

Ultra-rare damaging missense variants showed over-transmission to females (T/U = 1.13; 95% CI = 1.013 - 1.26; p = 0.028; FDR-adjusted p = 0.19; Bonferroni-corrected p = 1) but not males (T/U = 1.017; 95% CI = 0.096 - 1.077; p = 0.57). This was different from ultra-rare variants in trio-sequenced probands in SPARK, in whom these variants were over-transmitted to males (T/U = 1.042; 95% CI = 1.0010 - 1.085; p = 0.045; FDR-adjusted p = 0.20; Bonferroni-corrected p = 1) but not females (T/U = 1.030; 95% CI = 0.95 - 1.11; p = 0.46) (the over-transmission of ultra-rare variants in trios mirrored what was shown above for rare variants in the same individuals in Figure 1C).

Consequently, the liability was significantly higher than zero in females in the one cohort (Z = 0.043; 95% CI = 0.0046 - 0.081) and in males in the other (Z = 0.017; 95% CI = 0.00042 - 0.034), but not significantly higher than zero in a meta-analysis of the two (In males: Z = 0.014; 95% CI = 0.000096 - 0.028; p = 0.12; In females: Z = 0.021 ; 95% CI = -0.00095 - 0.044; p = 0.07). There was no significant sex difference in any of these ultra-rare variant comparisons. Siblings showed nominal under-transmission of PTVs that was not significantly different from zero on the liability scale (Figure S13B).

### 2.3.2. Ultra-rare variants in cases and controls

Case-control cohorts (including individuals recruited in family-based studies but currently without sequencing data from their parents) form a substantial portion of available autism cohorts. These constitute valuable independent datasets to study sex differences in the enrichment of ultra-rare variants. To leverage these data, we compared the enrichment of ultra-rare variants in 12,125 autistic individuals *versus* 10,962 controls or siblings not diagnosed with autism (Figure S7B). This analysis showed significant enrichment and liability attributed to damaging PTVs, and to a lesser extent damaging missense variants, without significant sex differences (Figure S14). Since the mode of inheritance of alleles in these cohorts is unknown, this analysis captures the combined effect size of *de novo* mutations and inherited variants. On the liability scale, the effect sizes attributed to damaging PTVs in females (Z=0.21; 95% CI = 0.16 - 0.25; p $=3.81\times10^{-21}$; Bonferroni-corrected p = $1.71\times10^{-19}$) and males (Z=0.28; 95% CI = 0.22 - 0.33; p = $8.03\times10^{-36}$; Bonferroni-corrected p = $3.61\times10^{-34}$) were not significantly different ($Z_{Difference}$ = -0.072; 95% CI = -0.14 - 0.0018 ; p =0.056).

### 2.3.3. Ultra-rare variants in mothers and fathers, parent-of-origin effects, and male-biased vulnerability

Previous work has shown that mothers of autistic children are more likely to carry rare damaging variants than fathers.[4] It was also hypothesized that the protective effect in females will render male children especially vulnerable to rare damaging alleles transmitted from mothers and conversely, females will be most protected against alleles transmitted from fathers.[5] Earlier work has shown relatively higher over-transmission from mothers (than fathers) to autistic male children,[4,6,7] but the findings from a more recent analysis were not consistent with the hypothesis of increased ‘male vulnerability’ to maternally inherited alleles.[1] These studies were performed in smaller cohorts largely overlapping with the current cohort. We therefore examined the burden of ultra-rare variants in mothers versus fathers of autistic children in a larger sample size, then went on to evaluate

over-transmission to male and female children (to test whether there are parent-of-origin or sex-biased protective effects).

First, we performed a ‘case-control’ analysis to compare the burden of ultra-rare parental alleles (seen in one parent and not in gnomAD) between the mothers and fathers of autistic individuals. Given the similar rates of ultra-rare parental alleles in ASC & SPARK (~6 synonymous ultra-rare parental alleles per child; Figure S7), we pooled all child-parent pairs in a mega-analysis (n = 55,521 child-parent pairs). Across these child-parent pairs, we found that the average rate of ultra-rare damaging protein-truncating variants in the mothers of autistic children (n = 22,496) were significantly higher than the rates in the fathers (n = 33,025) (Figure S15). This was most noticeable in PTVs in the 1st LOEUF decile (rate ratio = 1.15; 95% CI = 1.08 - 1.22; p = $2.67 \times 10^{-6}$; Bonferroni-corrected p =$1.34 \times 10^{-5}$) and less so in PTVs in the 2nd decile (rate ratio = 1.08; 95% CI = 1.03 - 1.14; p = $2.9 \times 10^{-3}$; Bonferroni-corrected p = 0.015). Ultra-rare damaging missense variants with MPC scores ≥ 2 were also enriched in mothers compared to fathers (rate ratio = 1.06; 95% CI = 1.03 - 1.09; p = $3.0 \times 10^{-5}$; Bonferroni-corrected p = $1.5 \times 10^{-4}$), more so than missense variants with MPC scores between 1 & 2 (rate ratio = 1.015; 95% CI = 1.0031 - 1.027; p = 0.013; Bonferroni-corrected p = 0.065).

Next, we calculated the transmission ratios (T/U) of ultra-rare alleles transmitted from mothers to sons (n = 26,021 pairs), mothers to daughters (n= 7,004), fathers to sons (n = 17,852), and fathers to daughters (n = 4,644) as well as the liability conveyed by these variants (Z scores). (Instead of autistic males and females, we use the terms sons and daughters to refer to autistic children in this section to avoid confusion with the sex of the parent). We performed six pairwise comparisons (listed below; see section 5.3 of the Supplemental Methods for details) to test whether there are parent-of-origin effects and whether sons are more vulnerable to maternally inherited alleles than paternally inherited alleles (conversely, whether daughters are more protected from paternally inherited alleles than maternally inherited alleles).

Specifically, we measured the significance of the following comparisons (the tests numbered ‘i’ through ’vi’ are labeled similarly in Figure S16):

i. Maternal alleles to daughters *versus* paternal alleles to daughters.

ii. Maternal alleles to sons *versus* paternal alleles to sons.

- *When looking at daughters and sons separately, do we see a difference between paternally and maternally inherited alleles*? We expect to see a significant difference if there are parent-of-origin effects e.g., maternal alleles having a larger effect size (than paternal alleles) in sons or paternal alleles having a significantly lower effect size (than maternal alleles) in daughters. In line with all other analyses, we did not test transmission to all children (sons and daughters) combined. Whether there was a

significant difference in such a test or not; we would have done post-hoc sex-stratified tests.

iii. Maternal alleles to daughters *versus* maternal alleles to sons.

iv. Paternal alleles to daughters *versus* paternal alleles to sons.

- *When looking at mothers and fathers separately, do we see a difference between daughters and sons*? If sons are especially vulnerable to maternal alleles, test 'iii' will be significant. If daughters are less vulnerable to paternal alleles, we expect to see a significant difference (daughters showing a lower effect size) in test 'iv'.

v. Maternal alleles to daughters *versus* paternal alleles to sons.

vi. Maternal alleles to sons *versus* paternal alleles to daughters.

- *Is there a significant difference when comparing the most and least vulnerable children?* Test 'vi' will be significant if there is a sex difference in genetic predisposition in the child (i.e. sons are more vulnerable to maternally inherited alleles and daughters are more protected from paternally inherited alleles). If test 'vi' is significant, the result on test 'v' can help indicate whether this is indeed due to a sex difference in the children rather than a parent-of-origin effect; specifically, if both test 'vi' and test 'v' are significant, this suggests a parent-of-origin effect, whereas if test 'vi' is significant and test 'v' is not significant, this suggests that there is a sex difference in the child.

We did not find significant sex differences in parental transmission or variant liability that may suggest parent-of-origin effects or sex-biased liability. The transmission ratios and Z scores did not differ significantly across these comparisons ($p > 0.05$) when we tested different thresholds for damaging PTVs ($1^{st}$, $1^{st}$ - $2^{nd}$ and $1^{st}$ - $3^{rd}$ LOEUF deciles) or missense variants (MPC ≥ 2, MPC ≥ 1). These results are presented in Figure S16. As the effect sizes of parental alleles were not significantly different, we went on and performed sex-stratified mega-analysis similar to the analysis presented in Figure 1C (n=21,043), leveraging the larger sample size (n=34,478 unique children). Again, we did not find significant sex differences between autistic females and males in these variant classes.

# 3. Examining the predisposition to autism with and without cognitive difficulties jointly in ASC and SPARK trios

We present here the findings from the analysis shown in Figure 2 and related analyses in gene sets.

## 3.1. Exome-wide enrichment and liability

The findings from the analysis of de novo mutations are presented in the main text. Here, we provide details on the effect size of inherited rare parental alleles.

### 3.1.1. *De novo* mutations

The sex differences on the observed scale in the meta-analysis were driven by DNMs from the ASC cohort, which showed female bias both amongst those with cognitive impairment (rate $\text{ratio}_{\text{Sex-Difference/ASC}}$ = 2.27; 95% CI = 1.53 - 3.34; $p = 3.3\text{x}10^{-5}$; Bonferroni-corrected $p$ = 0.0036) and without cognitive impairment (rate $\text{ratio}_{\text{Sex-Difference/ASC}}$ = 1.55; 95% CI = 1.18 - 2.03; $p$ = 0.0015; FDR-adjusted $p$ = 0.0047; Bonferroni-corrected $p$ = 0.17). The sex difference in SPARK was not significant whether with cognitive impairment (rate $\text{ratio}_{\text{Sex-Difference/SPARK}}$ = 1.26; 95% CI = 0.86 - 1.82; $p$ = 0.21) or without cognitive impairment (rate $\text{ratio}_{\text{Sex-Difference/SPARK}}$ = 1.23; 95% CI = 0.91 - 1.66; $p$ = 0.16); However, the observed sex differences before stratifying by cognitive impairment was not substantial and only significant before correction for multiple testing (rate $\text{ratio}_{\text{Sex-Difference/SPARK}}$ = 1.27 ; 95% CI = 1.004 - 1.60; $p$ = 0.043; Figure 1B). The sex difference in damaging missense DNMs was significant in SPARK (rate $\text{ratio}_{\text{Sex-Difference/SPARK}}$ = 1.34; 95% CI = 1.07 - 1.51; $p$ = 0.0080; FDR-adjusted $p$ = 0.021; Bonferroni-corrected $p$ = 0.86) but not in the ASC cohort (rate $\text{ratio}_{\text{Sex-Difference/ASC}}$ = 1.16; 95% CI = 0.86 - 1.53; p = 0.30). There was no significant sex difference in damaging missense DNM rates between those with cognitive impairment in the individual cohorts (rate $\text{ratio}_{\text{Sex-Difference/ASC}}$ = 0.92; 95% CI = 0.58 - 1.42; $p$ = 0.75; rate $\text{ratio}_{\text{Sex-Difference/SPARK}}$ = 1.14; 95% CI = 0.81 - 1.60; $p$ = 0.44).

The average effect size of protein truncating DNMs in those with autism and cognitive impairment was 0.66 in females (95% CI = 0.54 - 0.78; $p = 1.3\text{x}10^{-26}$; Bonferroni-corrected $p = 1.5\text{x}10^{-24}$) and 0.53 in males (95% CI = 0.42 - 0.64; $p = 5.2\text{x}10^{-21}$; Bonferroni-corrected $p = 5.6\text{x}10^{-19}$), without a significant difference ($p$ = 0.13). In those without cognitive difficulties, it was 0.49 in females (95% CI = 0.37 - 0.60; $p = 7.8\text{x}10^{-17}$; Bonferroni-corrected $p = 8.4\text{x}10^{-15}$)

and 0.42 in males (95% CI = 0.30 - 0.53; p = $7.4x10^{-12}$; Bonferroni-corrected = $8.0x10^{-10}$), and the difference was similarly not significant ($p$ = 0.24). Damaging missense DNMs increased the average liability in autistic individuals without cognitive impairment by 0.23 units in females (95% CI = 0.15 - 0.31; $p$ = $1.1x10^{-7}$; Bonferroni-corrected $p$ = $1.2x10^{-7}$) and 0.11 in males (95% CI = 0.04 - 0.20; $p$ = 9.8 $x10^{-5}$; Bonferroni-corrected $p$ = 0.011). In those with cognitive impairment, damaging missense DNMs had an average liability of 0.26 in females (95% CI = 0.17 - 0.36; $p$ = $3.8x10^{-7}$; Bonferroni-corrected $p$ = $4.1x10^{-5}$) and 0.24 in males (95% CI = 0.17 - 0.32; $p$ = $0.42x10^{-11}$; Bonferroni-corrected $p$ = $4.5x10^{-9}$). These effect sizes were not significantly different between the two sexes whether with cognitive impairment ($p$ = 0.71) or without it ($p$ = 0.052).

We have shown that the rates and effect sizes of protein-truncating variants in the $2^{nd}$-$3^{rd}$ LOEUF decile and missense variants with 2 > MPC ≥ 1 (Figure S8) did not differ by sex, nor the effect sizes of highly deleterious variants (PTVs in $1^{st}$ LOEUF decile, missense variants with MPC score ≥ 2) outside a limited group of SFARI high-confidence and syndromic genes (Figure S10). We performed a similar analysis across ASC & SPARK stratified by coexisting cognitive impairment, in which we removed SFARI genes and examined stringent and relaxed filtering thresholds (PTVs in $1^{st}$/$1^{st}$-$2^{nd}$/$1^{st}$-$3^{rd}$ LOEUF deciles; missense variants with MPC ≥ 1 or MPC ≥ 2). In this meta-analysis, the rate ratios of damaging *de novo* mutations and their attributed liability did not differ significantly between males and females (Figure S17).

The cohort-level analyses revealed more nuanced patterns. First, the effect size of damaging protein-truncating DNMs was significantly higher in females *versus* males with cognitive impairment in ASC ($Z_{Sex-Difference/ASC}$ = 0.30, 95% CI = 0.05 - 0.55, $p$ = 0.020, FDR-adjusted $p$ = 0.054), but not in SPARK ($Z_{Sex-Difference/SPARK}$ = 0.0037; 95% CI = -0.21 - 0.21; $p$ = 0.97). The liability estimated in ASC trios was 0.76 in females (95% CI = 0.57 - 0.94; $p$ = $6.3x10^{-16}$; Bonferroni-corrected $p$ = $6.9x10^{-14}$) and 0.45 in males (95% CI = 0.28 - 0.63; $p$ = $3.7x10^{-7}$; Bonferroni-corrected $p$ = $4.0x10^{-5}$), whereas in SPARK it was 0.58 both in females (95% CI = 0.43 - 0.74; $p$ = $3.3x10^{-13}$; Bonferroni-corrected $p$ = $3.6x10^{-11}$) and males (95% CI = 0.44 - 0.72; $p$ = $2.8x10^{-16}$; Bonferroni-corrected $p$ = $3.0x10^{-14}$). This could reflect an ascertainment bias from including relatively more females with severe phenotypes and profound difficulties (who are more likely to high-impact variants e.g., protein-truncating DNMs) in the ASC cohort resulting in over-estimated effect sizes in females. (To calculate the liability, we estimate the allele frequency of damaging variants in the autistic females in the population from the observed frequency in female probands).

Second, the effect size of damaging missense DNMs was significantly higher in females *versus* males without cognitive impairment in SPARK ($Z_{Sex-Difference/SPARK}$ = 0.17, 95% CI = 0.04 - 0.30, $p$ = 0.010, FDR-adjusted $p$ = 0.027, Bonferroni-corrected $p$ = 1), but not in

ASC ($Z_{Sex\text{-}Difference/SPARK}$ = -0.078; 95% CI = -0.29 - 0.13; *p* = 0.47). In SPARK, the effect size was 0.22 in females (95% CI = 0.12 - 0.32; *p* = $7.4 \times 10^{-6}$; Bonferroni-corrected *p* = $8.0 \times 10^{-4}$) and 0.05 in males (95% CI = -0.04 - 0.14; *p* = 0.29). In ASC, it was 0.25 in females (95% CI = 0.10 - 0.39; *p* = $0.0007.5 \times 10^{-4}$; FDR-adjusted *p* = 0.0027; Bonferroni-corrected *p* = 0.081) and 0.32 in males (95% CI = 0.17 - 0.47; FDR-adjusted *p* =$2.7 \times 10^{-5}$; p = $1.3 \times 10^{-4}$; Bonferroni-corrected *p* = 0.0029). This pattern is similar to what was seen when analyzing the full SPARK cohort before stratification on cognitive impairment (Figure 1B); there, we noticed that more stringent quality/frequency filtering of DNMs attenuates the sex difference in the estimated effect sizes of damaging missense DNMs (Figure S12).

### 3.1.2. Inherited variants

In the meta-analyzed cohort of autistic individuals with cognitive impairment (Figure 2C), over-transmission of damaging protein-truncating variants was not significantly different (*p* = 0.96) between males (rate ratio = 1.23; 95% CI = 1.09 - 1.38; *p* = $8.2 \times 10^{-4}$; FDR-adjusted *p* = 0.0029; Bonferroni-corrected *p* = 0.089) and females (rate ratio = 1.24; 95% CI = 1.0 - 1.53; *p* = 0.054). The sex difference was also not significant (*p* = 0.93) in the remaining individuals, where the transmission ratio was 1.17 in males (95% CI = 1.09 - 1.24; *p* = $3.3 \times 10^{-6}$; Bonferroni-corrected *p* = $3.5 \times 10^{-4}$) and 1.18 in females (95% CI = 1.04 - 1.34; *p* = 0.0091; FDR-adjusted *p* = 0.024; Bonferroni-corrected *p* = 0.99). In line with the analysis in the full cohort before stratification on cognitive or motor difficulties (Figure 1C), the increased transmission of damaging protein-truncating variants was driven by inherited variants seen in SPARK (Figure 2C). Damaging missense variants displayed a more homogeneous pattern where both ASC & SPARK showed significant, albeit small, over-transmission in males without cognitive impairment but not in females.

On the liability scale, damaging PTVs had a meta-analyzed effect size of 0.066 in females (95% CI = -0.002 - 0.13; *p* = 0.15) and 0.076 in males (95% CI = 0.03 - 0.12, *p* = $7.7 \times 10^{-5}$; Bonferroni-corrected *p* = 0.0083) when measured in those with cognitive impairment. The effect size was also comparable in those without cognitive impairment, both in females (Z = 0.06; 95% CI = 0.017 - 0.10; p = 0.0045; FDR-adjusted *p* = 0.013; Bonferroni-corrected *p* = 0.48) and males (Z = 0.062; 95% CI = 0.034 - 0.089; *p* = $1.3 \times 10^{-5}$; Bonferroni-corrected *p* = 0.0014). As noted above, this was driven by variants identified in SPARK. The effect size of damaging missense variants on the liability scale in males was 0.018 (95% CI = 0.011 - 0.025; *p* = $2.4 \times 10^{-6}$; Bonferroni-corrected *p* = $2.6 \times 10^{-4}$). In females, the effect size was lower (Z = 0.0086; 95% CI = -0.0034 - 0.021; *p* = 0.34) but the difference was not significant (*p* = 0.20). In those with cognitive impairment, the liability was not

significantly higher than zero in both sexes. As with transmission ratios, ASC & SPARK showed highly congruent patterns.

## 3.2. High-confidence and syndromic autism predisposition genes (SFARI genes)

We examined the enrichment and liability conveyed by SFARI high-confidence and syndromic genes across both ASC & SPARK, testing additional relaxed variant filters; We also performed similar comparisons against randomly selected genes matched for coding length, LoF constraint and adult expression.

The meta-analyzed rates of protein-truncating DNMs in SFARI genes (1st LOEUF decile) were significantly higher in autistic females compared to autistic males (Figure S18A), both when examining those with cognitive impairment (rate ratio = 2.0; 95% CI = 1.45 - 2.76, p =$2.3x10^{-5}$; Bonferroni-corrected p = $5.1x10^{-3}$) (rate ratio compared to matched genes = 1.57; 95% CI = 1.14 - 2.17; p = $5.7x10^{-3}$; FDR-adjusted p = 0.021) as well as the remaining individuals (rate ratio = 1.36; 95% CI = 1.03 - 1.79; p = 0.03; FDR-adjusted p = 0.083) (rate ratio compared to matched genes = 1.34; 95% CI = 1.03 - 1.79; p = 0.031; FDR-adjusted p = 0.083). Damaging missense variants (MPC ≥ 2) were significantly enriched in females *versus* males without cognitive impairment (rate ratio = 1.56; 95% CI = 1.14 - 2.15; p = $5.5x10^{-3}$; FDR-adjusted p = 0.014; Bonferroni-corrected p = 1) (rate ratio compared to matched genes = 1.54; 95% CI = 1.13 - 2.12; p = 6.9x10-3; FDR-adjusted p = 0.024; Bonferroni-corrected p = 1) but not in those with cognitive impairment (rate ratio = 1.11; 95% CI = 0.74 - 1.69, p = 0.61) (rate ratio compared to matched genes = 1.05; 95% CI = 0.70 - 1.60; p = 0.80). These results remained consistent when less restrictive filters were applied (Figure S18A). The effect sizes of damaging protein-truncating and missense DNMs on the liability scale were not significantly different between the sexes (p > 0.05) and were higher than what is expected from matched genes (Figure S18B). Amongst these groups, protein-truncating DNMs in the 1st LOEUF decile increased the liability by 1-1.33 units (0.77-1.11 units relative to matched genes); damaging missense DNMs increased the liability by 0.47-0.73 units (0.3-0.46). When looking at individual cohorts, protein-truncating DNMs in the ASC cohort had a significantly higher effect size in females (see 3.1.1 for a discussion of possible explanations).

Rare inherited variants did not show significant differences in over-transmission of damaging protein-truncating missense variants to autistic males and females (Figure S19A). Notably, the effect size of rare inherited PTVs in the 1st LOEUF decile in those diagnosed autism with cognitive impairment was significantly lower in females (Z = 0.0037; 95% CI =

-0.097 - 1.10; p = 0.31) than males (Z = 0.18; 95% CI = 0.034 - 0.32; p = 0.033; FDR-adjusted p = 0.077) when examining highly LoF-intolerant SFARI genes in the 1st decile ($Z_{Difference}$ = 0.24; 95% CI = 0.072 - 0.40; p = $4.9 \times 10^{-3}$; FDR-adjusted p = 0.015; Bonferroni-corrected p = 1), and this was driven by inherited variants in SPARK (Figure S19B). However, the difference was not significant when relaxing the filters to include PTVs in the 1st and 2nd ($Z_{Difference}$ = 0.024; 95%CI = -0.094 - 0.14; p = 0.69); Here, the effect size was 0.16 in males (95% CI = 0.069 - 0.25) and 0.15 in females (95% CI = 0.00063 - 0.29). This favors that the liability of damaging PTVs is not significantly different between males and females.

## 3.3. Sex-differentially expressed genes in the cortex

### 3.3.1. Male-biased genes in the adult cortex

**De novo mutations:**

Genes with male-biased differential expression in the adult cortex (n = 2,852) showed stronger enrichment of damaging protein-truncating DNMs in autistic females compared to autistic males with cognitive impairment (rate ratio ~ 2.5) but there was no significant sex difference on the liability scale. The effect size of damaging protein-truncating DNMs (in 483 genes in the 1st LOEUF decile) was 0.78 in males (95% CI = 0.53 - 1.03; Bonferroni-corrected p = $9.3 \times 10^{-7}$) and 0.74 in females (95% CI = 0.55 - 0.93; Bonferroni-corrected p = $2.0 \times 10^{-11}$) (difference: p = 0.82). Comparable outcomes (with smaller effect sizes) were observed when including protein-truncating DNMs in the 2nd and the 3rd LOEUF decile in the analysis (443 and 416 genes respectively). Damaging missense DNMs in autistic individuals with cognitive impairment, as well as damaging protein-truncating and missense DNMs in autistic individuals without cognitive impairment or with unknown status, were enriched in both sexes but there was no significant sex difference in rate ratios (observed scale) or Z scores (liability scale) (Figure S20).

Notably, the enrichment of damaging protein-truncating DNMs on the observed scale in this gene set (~2.5 times higher in females than males) was more prominent than amongst SFARI autism-predisposition genes (2 times higher in females than males), although the enrichment in each sex in SFARI genes was stronger, with rate ratio of ~32 in females and ~20 in males (~9 in females and ~8 in males in this gene set). (The difference between the estimates of males and females in each gene set is lower than indicated the female bias because the sex-stratified estimates are from comparisons between autistic children and siblings not diagnosed with autism whereas the sex difference is from direct comparisons between autistic females and males).

Moreover, both the enrichment and liability in male-biased differentially expressed genes were not significantly higher (after multiple testing correction) than what is expected from matched genes with similar coding length, LoF-constraint and brain expression. The effect sizes in SFARI genes, on the other hand, were higher than matched genes on both scales (Figure S18A). This suggests that the enrichment of damaging DNMs in this gene set in autistic males or females (the sex-stratified rate ratios and Z-scores in Figure S20) is a reflection of the enrichment of male-biased, sex-differentially expressed, cortical genes in LoF-constrained and highly-brain-expressed genes more than them conveying a relatively higher risk for autism (like SFARI genes). This enrichment (of LoF-constrained genes amongst male-biased differentially expressed genes in the adult cortex) requires validation and the mechanisms responsible for it are not yet known.

**Inherited variants:**

Damaging rare inherited protein-truncating and missense variants were not significantly over-transmitted among autistic individuals with cognitive impairment when examined in each sex separately (after correction for multiple testing). The transmission ratio of damaging missense variants (but not damaging PTVs) was significantly lower in females than in males (rate ratio = 0.89; 95% CI = 0.81 - 0.97; FDR-adjusted p = 0.043); yet, there was no significant sex difference on the liability scale (after correction for multiple testing) (Figure S21).

Among autistic individuals without cognitive impairment or with unknown status, there was evidence of significant over-transmission of damaging protein-truncating variants to both females and males (T/U ratios ~ 1 - 1.5; Z-scores ~ 0 - 0.2) and damaging missense variants to males only (T/U ratios ~ 1 - 1.08; Z-scores = 0 - 0.03). There was no significant sex difference on both scales for either variant types after correcting for multiple testing, and the effect sizes were not significantly higher than what is expected for matched genes. These results are presented in Figure S21.

### 3.3.2. Female-biased genes in the adult cortex

Protein-truncating DNM rates amongst a set of genes with female-biased expression in the adult cortex (n = 2,427) were not significantly different between females and males (Figure S22). Amongst autistic individuals with cognitive impairment, the effect size (on the liability scale) of protein-truncating DNMs in the 1$^{st}$ LOEUF decile (381 genes) was 0.51 in males (95% CI = 0.29 - 0.72; Bonferroni-corrected = 0.0012) and females (95% CI = 0.24 - 0.78; FDR-adjusted p = 0.0044; Bonferroni-corrected p = 0.066); these Z scores were not significantly different between the two sexes (p = 0.96). Similar patterns were seen when

including PTVs in the 2nd (344 genes) and the 3rd LOEUF deciles (319 genes) in the analysis. The enrichment in those without cognitive impairment was less pronounced (smaller effect sizes on the liability scale) than what is seen in those with cognitive impairment, particularly amongst males. The enrichment was not significantly higher than what is expected for a similarly sized gene set matched for coding length, LoF-constraint and brain expression. Damaging missense variants (MPC ≥ 2; MPC ≥ 1) were not enriched in males or females in either of these two phenotypic groups after correction for multiple testing (Figure S22). Similarly, rare inherited protein-truncating and missense variants in this gene set did not convey significant increase in liability (Figure S23).

### 3.3.3. Sex-biased genes in the fetal cortex

We did not find a significant enrichment of damaging *de novo* and rare inherited protein-truncating variants - after correction for multiple testing - in 305 male-biased genes (39 genes in the 1st LOEUF decile, 49 in the 2nd decile, 42 in the 3rd decile) (see Figure S24 for DNMs and Figure S25 for rare inherited variants) nor 117 genes with female-biased expression in the fetal cortex (8 genes in the 1st LOEUF decile, 15 in the 2nd decile, 11 in the 3rd decile) (see Figures S26 for DNMs and Figure S27 for rare inherited variants), neither with the primary variant filters (PTVs in the 1st LOEUF decile, missense with MPC score ≥ 2) nor relaxed filtering (1st-3rd LOEUF deciles, MPC ≥ 1). Damaging missense DNMs in male-biased genes were significantly more enriched in females *versus* males amongst those without cognitive impairment or with unknown status (Figure S24), and this was driven by SPARK (however, the enrichment in each sex was not significant after correction for multiple testing). The effect sizes on the liability scale (for all the variant classes and phenotypic groups) did not differ significantly from zero (after correction for multiple testing). Some of these analyses were under-powered due to the small number of genes and several tests could not be performed (especially in female-biased genes; Figures S26-S27).

### 3.3.4. Limitations

It is important to approach the interpretation of all the presented results on sex-differentially-expressed genes in the adult and fetal cortex with caution given the heterogeneity of expression data[8,9] (which we aimed to mitigate by using results from meta-analyzed RNA-seq datasets to define gene sets), the exclusion of genes on chromosome X in our analysis, and the fact that we did not account the extent of sex-differential expression (fold-change) as well as other several sources of bias, e.g., the differences across brain regions and cell types and the time points at which expression is examined. In particular, limiting the analysis to autosomal genes showing sex differential

expression in the cortex has a substantial influence on the results of gene set analysis. For instance, Kissel *et al*.[10] observed that the enrichment of (fetal) female-biased differentially expressed genes in Developmental and Epileptic Encephalopathies (DEE) genes was driven by female-biased genes on chromosome X; Fass *et al.*[11] found a significant overlap between high-confidence autism predisposition genes and female-biased differentially expressed genes in several (adult) brain regions except for the cortex, which showed an enrichment of autism genes in male-bias differentially expressed genes. Interestingly, the enrichment in non-cortical regions was still attributed to the fact that many of the risk loci were found on the X chromosome, which renders them more likely to be upregulated in females. Replication in independent datasets or testing genes that show sex differences in protein expression[12] may add new insights.

# 4. Liability conveyed by *de novo* and rare variants in autistic individuals with *versus* without cognitive and motor difficulties in SPARK

## 4.1. Exome-wide effects of *de novo* and rare inherited variants

Here, we describe in more detail the findings presented in Figure 3 (SPARK trios).

### 4.1.1. Damaging DNMs

Contrary to the observed sex differences seen before stratifying by phenotype (rate $\text{ratio}_{\text{Sex-Difference/SPARK}}$ = 1.27 ; 95% CI = 1.004 - 1.60; p = 0.043; Figure 1B), rates of damaging *de novo* protein-truncating mutations were not significantly different between autistic females and males with motor and cognitive impairment (rate $\text{ratio}_{\text{Sex-Difference}}$ = 1.12; 95% CI = 0.79 - 1.57; p = 0.49) or autistic females and males without these difficulties (rate $\text{ratio}_{\text{Sex-Difference}}$ = 1.25; 95% CI = 0.84 - 1.83; p = 0.23). The effect size attributed to protein-truncating DNMs in autistic individuals with co-occurring motor or cognitive impairment was 0.60 in males (95% CI: 0.46 - 0.74; p = $2.3\times10^{-17}$; Bonferroni corrected p = $1.3\times10^{-15}$) and 0.55 in females (95% CI = 0.40 - 0.71; p = $3.2\times10^{-12}$; Bonferroni corrected p = $1.1\times10^{-10}$), without a significant difference ($Z_{\text{Sex-Difference}}$ = -0.045; 95% CI = -0.26 - 0.17; p = 0.68). *De novo* protein-truncating mutations increased the liability to autism without cognitive impairment or motor delay similarly in males (Z = 0.32; 95% CI: 0.16 - 0.47; p = $5.9\times10^{-5}$; Bonferroni corrected p = 0.0032) and females (Z = 0.34; 95% CI: 0.17 - 0.51; p = $6.2\times10^{-5}$; Bonferroni corrected p = 0.0034) ($Z_{\text{Sex-Difference}}$ = 0.026; 95% CI = -0.20 - 0.25; p =0.82). When comparing autistic individuals with motor delay or cognitive impairment to those without these co-occurring difficulties, these DNMs had similar liabilities (p = 0.66) in males (Z = 0.45; 95% CI = 0.30 - 0.61; p = $7.3\times10^{-9}$; Bonferroni-corrected p = $4\times10^{-7}$) and females (Z = 0.39; 95% CI = 0.11 - 0.66; p = 0.0053; FDR-adjusted p = 0.019).

Damaging *de novo* missense mutations increased the liability both in males (Z = 0.17; 95% CI = 0.077 - 0.25; p = 0.00024 ; Bonferroni-corrected p = 0.0016) and in females with motor or cognitive impairment (Z = 0.27; 95% CI = 0.16 - 0.34; p = $2.2\times10^{-6}$; FDR-adjusted p =0.00012) - without a significant difference (p = 0.16). In those without motor or cognitive impairment, these DNMs significantly increased autism liability in females (Z = 0.016; 95% CI: 0.05 - 0.27 ; p =0.0043; FDR-adjusted p = 0.018) but not in males (Z = 0.029; 95% CI: -0.07 - 0.13 ; p =0.57), though the difference was not significant (p = 0.081).

In direct comparisons between those with and without these difficulties, damaging missense DNMs had an increased liability to co-occurring difficulties in males (Z = 0.23; 95% CI = 0.10 - 0.35; p = 0.00032; Bonferroni-corrected p = 0.017) but the liability was not significantly increased in females (Z = 0.21; 95% CI = -0.012 - 0.43; p = 0.071). The difference was not statistically significant ($Z_{Difference}$ = -0.023; 95% CI = -0.28 - 0.23; p = 0.86).

## 4.1.2. Inherited damaging variants

In autistic individuals with motor or cognitive impairment (Figure 3C), inherited PTV liability was increased in males (Z = 0.12; 95% CI = 0.076 - 0.16; p = $1.1 \times 10^{-7}$; Bonferroni-corrected p = $6 \times 10^{-6}$) but not significantly so in females (Z = 0.063; 95% CI = -0.0060 - 0.13 ; p = 0.073), yet the difference was not significant ($Z_{Difference}$ = -0.057; 95% CI = -0.14 - 0.025; p = 0.17). In the other group (without co-occurring difficulties), inherited PTVs had comparable effect sizes (p = 0.54) in males (Z = 0.06; 95% CI: 0.25 - 0.10; p = 0.0049; Bonferroni-corrected p = 0.046) and in females (Z = 0.08; 95% CI: 0.25 - 0.14; p = 0.00085; FDR-adjusted p = 0.019). Notably, ultra-rare inherited variants ascertained in a separate cohort of autistic individuals with sequence data from one parent showed similar patterns in those with motor or cognitive difficulties but not in those without co-occurring difficulties (see 4.3.1 below).

Among those with motor or cognitive impairment (Figure 3C), inherited damaging missense variants conveyed significant liability in males (Z = 0.015; 95% CI = 0.0030 - 0.027; p = 0.015; FDR-adjusted p = 0.047) but not in females (Z = -0.011; 95% CI = -0.30 - 0.0083; p = 0.27). The difference was significant only before correction for multiple testing (Z = -0.026; 95% CI = -0.048 - 0.0032; p = 0.025). The liability attributed to inherited damaging missense variants in females without cognitive impairment was not significantly increased (Z = 0.00035; 95% CI = -0.13 - 0.02). These variants increased the liability in males (Z = 0.01; 95% CI = 0.0006 - 0.02; p = 0.037) but this did not pass FDR-adjustment (p = 0.11). There was no significant difference in the average liability of inherited missense variants between those with and without co-occurring difficulties (p > 0.43).

## 4.1.3. Imbalance of synonymous DNMs

As presented in the main text (Figure 3B), synonymous DNMs were more prevalent in autistic females without motor or cognitive impairment compared to those with these co-occurring difficulties as well as sex-matched siblings (rate ratio = 1.23; 95% CI = 1.082 - 1.38; p = 0.00099). We examined the burden and liability in ultra-rare DNMs in this sub-cohort (Figure S28) and found better-balanced DNM counts between the probands and siblings, yet not completely controlling the spurious association (rate ratio = 1.22; 95% CI =

1.038 - 1.43; p = 0.015), which is likely to be an artifact of the diverse ancestries included in the sample.

We then examined whether limiting the analysis to a stringently ancestry-matched set of 868 autistic females and 2,235 siblings could resolve this issue (see section 3.4 of the Supplemental Methods). This ancestry matching alone resulted in better-balanced DNM counts between the probands and siblings with some residual imbalance (rate ratio = 1.17; 95% CI = 1 - 1.37; p = 0.047). Next, we combined the two filtering strategies, i.e. by evaluating ultra-rare DNMs in samples well-matched on genetic ancestry. Here, synonymous DNMs were not associated with autism (rate Ratio = 1.09; 95% CI = 0.88 - 1.35; p = 0.42).

Lastly, we turned to protein-truncating DNMs to see whether the estimated enrichment in protein-truncating DNMs differed with more stringent frequency and ancestry matching. The rare DNM rate ratio was 2.65 for rare DNMs in the complete cohort (95% CI = 1.61 - 4.37; p = $6.2 \times 10^{-5}$) *versus* 2.68 in ancestry-matched samples (95% CI = 1.47 - 4.9; p = 0.00067). The ultra-rare DNM rate ratio was 2.66 in the complete cohort (95% CI = 1.6 - 4.42; p = $8.4 \times 10^{-5}$) *versus* 2.57 in ancestry-matched samples (95% CI = 1.4 - 4.7; p = 0.0018). Thus, the estimates of protein-truncating DNM enrichment obtained when examining the full cohort were robust to these differences in genetic ancestry and were slightly more precise.

## 4.2. *De novo* and rare variants in high-confidence and syndromic SFARI genes

### 4.2.1. Observed effect sizes

Among autistic individuals with motor and cognitive impairment (Figure S29A), protein-truncating DNMs were enriched in autistic males (rate ratio = 23.72; 95% CI = 7.78 - 117.82; p = $6.3 \times 10^{-18}$; Bonferroni-corrected p = $9.1 \times 10^{-16}$) and females (rate ratio = 22.04; 95% CI = 8.53 - 72.47; p = $2.4 \times 10^{-16}$; Bonferroni-corrected p = $3.4 \times 10^{-14}$) but not significantly different between the two sexes (risk ratio = 1.48, 95% CI = 0.94 - 2.28; p = 0.079). Amongst those without these co-occurring conditions, protein-truncating DNMs were also significantly enriched in autistic males (rate ratio = 9.43; 95% CI = 3.05 - 47.25; p = $4.4 \times 10^{-7}$; Bonferroni-corrected p = $6.3 \times 10^{-5}$) and females (rate ratio = 7.56; 95% CI = 2.65 - 26.38; p = $1.5 \times 10^{-5}$; Bonferroni-corrected p = 0.0021) without a significant difference between the sexes (risk ratio = 1.28, 95% CI = 0.68 - 2.27; p = 0.36). As expected for these known developmental genes, protein-truncating DNMs were substantially enriched in those with motor or cognitive impairment *versus* those without co-occurring difficulties, in males (rate

ratio = 2.51; 95% CI = 1.73 - 3.68; p = $6.6x10^{-7}$; Bonferroni-corrected p = $9.5x10^{-5}$) and females (rate ratio = 2.92; 95% CI = 1.55 - 5.69; p = $3.4x10^{-4}$; Bonferroni-corrected p = 0.048). The enrichment in missense DNM enrichment in SFARI genes was less prominent and there were no significant sex differences in rate ratios after accounting for multiple testing; these DNMs were also more enriched in those with motor or cognitive difficulties *versus* those without these co-occurring difficulties. The observed sex differences in DNM risk ratios did not translate to a significant sex-bias on the liability scale (Figure S29A). Inherited variants did not show significant differences in over-transmission rates and liability compared to matched genes (Figure S29B).

### 4.2.2. Effect sizes relative to matched genes

Both the rate ratio in autistic individuals *versus* siblings and the rate ratio in autistic females *versus* autistic males (sex difference) were significantly higher than what is expected from matched genes, i.e, significantly higher than the rate ratio calculated from randomly selected genes matched on LoF-constraint, sex-averaged brain expression and coding length profiles. In the full cohort analysis (not split by coexisting motor or cognitive impairment), this was evident in protein-truncating (driven by autistic probands who have motor/cognitive impairment) as well as missense DNMs (driven by those without motor/cognitive impairment). Notably, autistic females and males in the ‘motor and cognitive impairment’ group do not show a significant sex difference in their DNM rates *per se*, i.e. relative to what is expected from the sample size of the cohort, yet the sex difference in DNM rates is significantly higher than what is expected from matched genes. In the same vein, damaging missense DNMs in the ‘autism without motor/cognitive impairment’ had significantly higher female-bias when compared to matched genes but not when tested relative to the sample size. These findings are presented in Figure S29.

On the liability scale, protein-truncating DNMs in SFARI genes increased the liability to autism with cognitive and motor impairment by 0.99 units more than a matched gene set in males (95% CI = 0.64 - 1.34; p = $2.6x10^{-8}$; Bonferroni-corrected p = $3.7x10^{-6}$) and 0.86 units more that matched genes in females (95% CI = 0.59 - 1.14; p = $1.1x10^{-9}$; Bonferroni-corrected p = $1.6x10^{-7}$), without a significant sex difference (p= 0.58). In the ‘autism without cognitive & motor impairment’ group (relative to matched genes), the sex difference between males (Z = 0.89; 95% CI = 0.49 - 1.30; p = $1.8x10^{-5}$; Bonferroni-corrected p = 0.0025) and females (Z = 0.52; 95% CI = 1.7 - 0.86 ; p = 0.0032; FDR-adjusted p= 0.017; Bonferroni-corrected p = 0.46) was also not significant (p = 0.17). Despite this sex bias in the enrichment relative to matched genes, the ‘excess’ autism liability conferred by damaging variants in SFARI genes on top of what is expected from matched genes was not

significantly different between males and females (i.e. for these comparisons against matched genes, the sex difference on the observed scale did not translate into differences on the liability scale).

### 4.2.3. Permutation analysis

The lack of significant sex differences in DNM rates in SPARK is consistent before (Figure 2) and after (Figure 3) considering motor difficulties/removing those with missing information. As mentioned before (section 2.1), the remaining autosomal genes did not show significant sex-differences in DNM rate ratios (Figure S10). Since SFARI genes are known to cause multiple developmental difficulties with high penetrance including cognitive impairment and motor delays, this suggests that the sex difference on the observed scale may be driven by differences in the proportions of autistic females and males with these co-occurring difficulties (40% in females *versus* 35% in males; relative risk = 1.16). Indeed, there is no significant sex bias when the analysis is performed separately in those with/without motor or cognitive impairment as we show here. However, it is possible that the smaller sample size in these stratified analyses reduced the power. Moreover, we do not see similar results in ASC (Figure 2B). We ran a permutation analysis by shuffling the sex labels within the four phenotypic groups (i.e. sampling without replacement within these groups: autism with motor or cognitive impairment, without coexisting difficulties, unknown status, siblings) before examining the full cohort again, so that the proportion of individuals with coexisting conditions is kept constant, then calculated the sex-stratified variant rates. We repeated this procedure 1,000 times and took the average rate of synonymous & damaging DNMs, then examined the rate ratios between the probands and siblings. We did not see any significant sex difference when we performed this analysis for all protein-coding genes (exome-wide), SFARI high-confidence and syndromic autism genes or the remaining genes (Figure S30). This suggested that the difference in the proportion of co-occurring difficulties was not the main determinant of the difference in variant rates - congruent with a recent ASC analysis (using regression to evaluate the odds of harboring deleterious DNMs that are associated with sex and cognitive impairment status) which did not find evidence for a significant interaction between sex and cognitive impairment.[13]

## 4.3. Exome-wide burden of ultra-rare variants in the remaining individuals

### 4.3.1. Over-transmission in children with sequence data from one parent

As shown in Figure S31, inherited PTVs conferred significant liability in males with motor or cognitive co-occurring (Z = 0.13; 95% CI = 0.057 - 0.20; p = 0.00052; Bonferroni-corrected p = 0.14) but not females (Z = 0.083; 95% CI = -0.048 - 0.21; p = 0.21), yet without a significant sex difference (p = 0.54), whereas the liability in the other group was not significantly increased from the population mean in both sexes (p > 0.11). The liability attributed to inherited PTVs did not differ significantly between those with and without co-occurring difficulties (p > 0.31). Ultra-rare damaging missense variants in SPARK individuals with sequence data from one parent did not convey significant liability.

### 4.3.2. Enrichment of ultra-rare variants in the remaining individuals without parental sequence data

There were no significant sex differences in the enrichment and liability damaging of protein-truncating and missense ultra-rare variants among the remaining probands and siblings from SPARK (Figure S32). The effect size of ultra-rare protein-truncating variants (adjusted for synonymous variants) was similar in males (Z = 0.27; 95% CI = 0.19 - 0.34; p = $1.4x10^{-11;}$ Bonferroni-corrected p = $6.4x10^{-10}$) and females with motor and cognitive difficulties (Z = 0.22; 95% CI = 0.15 - 0.30; p = $2.2x10^{-8}$; Bonferroni-corrected p = $1.0x10^{-7}$). Damaging missense variants had an effect sizes of 0.039 in males (95% CI = 0.0096 - 0.068; p = 0.0094; FDR-adjusted p = 0.033; Bonferroni-corrected p = 0.42) and 0.036 in females with cognitive impairment or motor delay (95% CI = 0.0020 - 0.069; p = 0.038; FDR-adjusted p = 0.095).

Among those without motor and cognitive difficulties, PTVs had an effect size of 0.16 in males (95% CI = 0.074 - 0.24; p = 0.00021; Bonferroni-corrected p = 0.0096) compared to 0.040 in females (95% CI = -0.056 - 0.42; p = 0.42) although the difference was not statistically significant (p = 0.068). There was no significant enrichment in damaging missense variants in autistic individuals without these co-occurring conditions. When the two groups were compared (with *versus* without motor or cognitive impairment), the effect size of PTVs was 0.19 in males (95% CI = 0.089 - 0.29; p = 0.00023; Bonferroni-corrected p = 0.010) and 0.35 in females (95% CI = 0.17 - 0.53; p = 0.00017; Bonferroni-corrected p = 0.0079), and it was not significantly different (p = 0.14). Missense variants had a slightly higher liability to autism with (vs. without) motor or cognitive impairment in males (Z = 0.048;

95% CI = 0.0046 - 0.092; p = 0.03) but the liability did not differ between the two groups after correction for multiple testing (FDR-adjusted p = 0.08).

## 4.4. Effect sizes of *de novo* mutations in an NDD cohort

To test whether the genetic predisposition conveyed by damaging DNMs is sufficient to reach the threshold to have developmental impairments (i.e. 'highly' or 'fully penetrant') and not significantly different between the sexes, we estimated their liability in 31,565 trios diagnosed with neurodevelopmental disorders (NDDs) not specifically ascertained for autism (17,698 males and 13,867 females, sex ratio ~ 1.3). These DNMs were curated by Zhou and colleagues[2] from several published cohorts of trio-sequenced children with developmental disorders[14] (17,422 males and 13,636 females), developmental and epileptic encephalopathies[15] (210 males and 146 females) or severe intellectual disability[16,17] (66 male and 85 females).

The observed variant counts were compared to those expected from a mutational model (see section 5.1 of the Supplemental Methods). To estimate liability, we used a prevalence of 2% in males and 1.5% in females (sex ratio ~ 1.3). In addition to SFARI genes, we also tested another set of genes associated with developmental disorders with high-confidence[18] (DDG2P v4.6). Most of the autosomal SFARI genes are also DDG2P genes (281/354, 240 mono-allelic and 41 bi-allelic genes). Specifically, we tested the enrichment and liability in following sets:

- High-confidence autism genes: 354 SFARI genes (218 in the 1st LOEUF decile).
- High-confidence NDD genes: 845 monoallelic DDG2P genes (384 in the 1st decile).
- High-confidence NDD genes with high autism penetrance: 240 genes shared between SFARI & monoallelic DDG2P sets (173 in the 1st decile).
- High-confidence NDD genes with low autism penetrance: 605 DDG2P genes that are not amongst SFARI genes (209 in the 1st decile).
- Low confidence/penetrance NDD genes: 15,032 autosomal protein-coding genes after removing SFARI & mono-allelic DDG2P genes (1,315 in the 1st decile).

On the observed scale, the higher enrichment in damaging DNMs in females *versus* males was driven by known autism and NDD genes (Figure S33B). SFARI genes showed significant female bias in damaging protein-truncating in the 1st LOEUF decile (rate ratio = 1.17; 95% CI = 1.07 - 1.28; Bonferroni-corrected $p$ = 0.017) and missense DNMs with MPC score ≥ 2 (rate ratio = 1.12; 95% CI = 1.02 - 1.24; FDR-adjusted $p$ = 0.032). DDG2P genes showed similar female bias (protein-truncating DNMs: rate ratio = 1.15; 95% CI = 1.06 -

1.24; 0.028) (missense DNMs: rate ratio = 1.15; 95% CI = 1.06 - 1.24; Bonferroni-corrected $p = 0.033$).

In DDG2P genes, the enrichment in protein-truncating DNMs was driven by a subset overlapping SFARI genes (rate ratio = 1.18; 95% CI = 1.08 - 1.28; Bonferroni-corrected $p = 0.012$) rather than the remaining DDG2P genes (rate ratio = 1.04; 95% CI = 0.88 - 1.23; $p = 0.67$). The enrichment in damaging missense DNMs, however, was nominally higher in DDG2P genes not in SFARI genes (rate ratio = 1.17; 95% CI = 1.03 - 1.33; FDR-adjusted $p = 0.027$) than those overlapping SFARI genes (rate ratio = 1.13; 95% CI = 1.02 - 1.25; FDR-adjusted $p = 0.029$). This may indicate that there are additional genes with female bias driven by missense variants that are not (yet) confidently associated with autism (e.g., bias in gene discovery towards pLoF variants) or that have limited/low penetrance for autism. Notably, the burden of protein-truncating DNMs amongst the remaining autosomal genes was significant higher in males versus females when SFARI & DDG2P genes were removed (rate ratio = 1.26; 95% CI = 1.06 - 1.49; FDR-adjusted $p = 0.014$). It remains to be clarified how this relates to the phenotypic spectrum of NDDs.

In all these gene sets, the difference in liability between the two sexes was not significant (Figure S33C). Damaging protein-truncating DNMs in SFARI genes increased NDD risk by 1.89 units in males (95% CI = 1.43 - 2.35; Bonferroni-corrected $p < 1x10^{-20}$) and 1.94 in females (95% CI = 1.48 - 2.41; Bonferroni-corrected $p < 1x10^{-20}$). These effect sizes overlapped the threshold (~2 in males, ~2.2 in females) and were nominally higher than those estimated in DDG2P genes ($Z_{Males}$ = 1.77; 95% CI = 1.35 - 2.20; Bonferroni-corrected $p < 1x10^{-20}$) ($Z_{Females}$ = 1.8; 95% CI = 1.37 - 2.24; Bonferroni-corrected $p < 1x10^{-20}$). This indicates that SFARI genes are nearly fully penetrant for NDDs. Amongst DDG2P genes, the liability was substantially higher amongst genes that are also SFARI genes (~2 units) compared to other DDG2P genes that are not (~1.3 units), again highlighting the high penetrance of SFARI genes for developmental phenotypes.

# 5. Altering the assumptions of the liability model

## 5.1. Lower threshold in females

In the different-thresholds model, the sex difference in autism prevalence is attributed to females having a higher threshold than males, with the assumption that the underlying liability distribution is the same in both sexes (Figure S5B). Estimating the effect sizes under this model requires assumptions about the liability threshold in males and females, which are calculated from estimated population prevalences in males and females using the inverse normal function. In the primary analysis, we used a prevalence estimate of ~2.5% in males and assuming the sex ratio is 4:1 we estimated the prevalence in females to be ~ 0.63%. Accordingly, we set the threshold in our primary analyses to ~1.96 in males and ~2.5 in females. However, the prevalence estimates in females are less certain (due to e.g., relative rarity or under-diagnosis in females) and the sex ratio could be overestimated.[19] Therefore, we tested two alternative models using ratios of 3:1 and 2:1. We did not see evidence of substantial differences in rare variant effect sizes on the liability scale after correction for multiple testing (Figure S34).

## 5.2. Higher variance in males

The true population parameters of the liability distribution are unknown. Sex differences in autism prevalence can alternatively be attributed to the two sexes having differing variances rather than differing thresholds (Figure S5B). We therefore tested an alternative model in which we assumed that the normally distributed liability in the general population (50% females) is the outcome of two underlying liability distributions in males and females with equal means in both sexes but with higher variance in males (standard deviation = 1.2) than females (standard deviation = 0.75) (variance ~2.5 times higher in males). We chose these two parameters so that the population variance is approximately equal to 1. (See section 6.2 of the Supplemental Methods for additional details).

We found that damaging protein-truncating and missense de novo mutations did not show significant differences in their effect sizes between males and females ($p > 0.05$). In contrast, inherited damaging protein-truncating variants had higher effect sizes in males ($Z = 0.077$; 95% CI = 0.051 - 0.10; $p = 1.2 \times 10^{-8}$; Bonferroni-corrected $p = 6.3 \times 10^{-7}$) than females ($Z = 0.032$; 95% CI = 0.0070 - 0.057; $p = 0.0017$; FDR-adjusted $p = 0.0046$; Bonferroni-corrected $p = 0.092$) and the difference was significant after multiple testing correction ($Z_{Difference} = 0.044$; 95% CI = 0.0088 - 0.081; $p = 0.015$; FDR-adjusted $p = 0.035$;

Bonferroni-corrected $p = 0.80$). Inherited damaging missense variants also had significantly higher effect sizes ($Z_{Difference} = 0.015$; 95% CI = 0.0037 - 0.027; $p = 0.0099$; FDR-adjusted $p = 0.025$; Bonferroni-corrected $p = 0.53$) in males ($Z = 0.019$; 95% CI = 0.012 - 0.026; $p = 4.5 \times 10^{-7}$; Bonferroni-corrected $p = 2.4 \times 10^{-5}$) than females ($Z = 0.0037$; 95% CI = -0.0055 - 0.013; $p = 0.65$). These comparisons are shown in Figure S35.

# Supplemental Methods

## 1. SPARK dataset preparation

### 1.1. Exome Sequencing and phenotypic data

We downloaded the Simons Foundation Powering Autism Research for Knowledge (SPARK) integrated whole-exome sequencing data[2] release version 2 through Globus.[20] This release (jointly called VCF v2.2023_01, Deep Variant/GLNexus) encompassed 106,744 samples from five sequencing batches (WES1: 27,177 samples, WES2: 15,671, WES3: 16,476, WES4: 11,340, WES5: 36,080), sequenced using two capture systems (WES1-4: IDT, WES5: TWIST). The dataset included 44,304 autistic individuals (33,335 males and 10,969 females) and 62,440 individuals without an autism diagnosis (24,783 males and 37,657 females), including 27,775 father-child-pairs (19,977 autistic individuals) and 46,343 mother-child pairs (34,011 autistic individuals). These formed 25,386 trios (18,172 autistic individuals), 23,346 samples with one sequenced parent (17,644 autistic individuals), and 58,012 samples without parental WES (8,488 autistic individuals). The phenotypic data for the autistic individuals were downloaded from SFARI's Genotypes and Phenotypes in Families (GPF) database.[21] These phenotypic data were available for 40,346 autistic individuals with WES data.

### 1.2. Variant annotation

The provided dataset included 10,202,547 sites (11,236,834 after splitting multiallelic sites). These sites were annotated using Variant Effect Predictor[22] (VEP) Ensembl release 108. The following annotations were added: consequences on Ensembl genes and transcripts, exon/intron numbers, allele frequencies based on gnomAD exomes (r2.1.1) and gnomAD genomes (r3), MPC scores (v2, gnomAD), Loftee predictions, NMD plugin predictions.[23] The consequences were annotated on 'MANE Plus Clinical' transcripts (Matched Annotation between NCBI and Ensembl v1). For variants with consequences on more than one MANE transcript, one consequence was prioritized based on the severity of the predicted effects or gene constraint. We produced a working dataset in which we split multiallelic sites, set any variants with depth zero to missing, and filtered for variants that were in the exonic regions or the adjacent splice regions of MANE/Clinical transcripts.

## 1.3. Ancestry inference

SPARK provided ancestry labels based on the 1000 Genomes super-populations (Africans [AFR], Admixed Americans [AMR], East Asians [EAS], Europeans [EUR], and South Asians [SAS]), along with subpopulation labels, and probabilities for being assigned to these sub-populations. A group of 2,783 samples were labeled as having an unknown super-population (Table S3). To confirm these labels and to classify the samples in the 'Unknown' group to the nearest population when possible, we projected SPARK iWES2 samples onto a principal components (PCs) space based on 2,536 samples from the 1000 Genomes Project[24] calculated using PLINK.[25] PC analysis of the 1000 Genomes reference data (10 PCs) leveraged 8,718 pruned variants with allele frequency > 1% that are present in at least one sample in SPARK samples, limited to exonic regions of protein coding transcripts described above. SPARK samples were then projected into the 1,000 Genomes PC space. The clustering of these samples was contrasted with the pre-defined ancestry labels. We reclassified the samples where the superpopulation was labeled as 'unknown' to one of the 1,000 Genomes superpopulations if these had a probability exceeding 0.8 of belonging to that group (calculated as the sum of probabilities of belonging to the sub-populations under that group, provided by SPARK), or clustered with that group on each of the first four PCs. Last, we defined a group of admixed individuals that did not cluster closely with their corresponding groups on PC1-4 (Table S4 and Figure S2).

## 1.4. Sex inference

To infer the ploidy of sex chromosomes from sequencing data, we evaluated the depth and genotypes in the hemizygous regions of chrX and chrY, namely, (1) the read depth in chrX normalized by the median chrX depth in samples labeled as males, (2) the read depth in chrY normalized by the median chrY depth in samples labeled as males, (3) the fraction of missing calls on chrY, and (4) the F statistic (the difference between the expected and observed heterozygosity in the hemizygous region of chrX; PLINK). Male sex was inferred for samples with normalized chrY depth between 0.5 and 2, fraction of missing genotypes in chrY < 50%, normalized chrX depth < 2, and F statistic > 0.8. Female sex was inferred for samples with normalized chrX depth between 1 and 3, F statistic > -0.6 and < 0.6, normalized chrY depth < 0.15, and fraction of missing genotypes on chrY > 50%. 122 male and 88 female samples were classified as having ambiguous inferred sex or having a mismatch between reported and inferred sex, and were removed from the burden analyses.

## 1.5. Relatedness inference

The provided meta-data indicated the presence of 27,775 father-child-pairs and 46,343 mother-child pairs (25,386 complete trios). To verify the parental relationships, kinship was estimated using PLINK based on KING's robust algorithm.[26] We required the parent-child pairs to have a kinship estimate between 0.3 and 0.1 and a proportion of SNPs with Identity By State (IBS) sharing < $5x10^{-5}$. Ten parent-child pairs could not be verified based on kinship. A set of maximally unrelated probands (N=39,020) was selected by incrementally removing the samples with the highest number of related autistic individuals (kinship > 0.0884) while prioritizing those with sequenced parents and females in that order. Ties were resolved by retaining the sample with the older identifier. Similar sets of unrelated individuals were defined for the siblings (N=15,056) and parents (N=47,525). We estimated the allele frequencies, Hardy Weinberg Equilibrium *p*-values, and Excess Heterozygosity *p*-values separately in these three sets of unrelated samples (per population) and used the largest *p*-value (i.e. the least significant) as an input for variant quality control (QC).

## 1.6. Variant QC

A Random Forest was trained to identify low quality variant call sites. It was applied separately on SNVs and indels and the training sites were selected from variants heterozygous in one or more samples. Before running the variant QC, we applied basic genotype filtering by setting the genotypes that had any of the following to missing: (1) depth equal to zero, (2) genotype quality (GQ) equal to zero, (3) a conflict between the called genotype and the phred-scaled genotype likelihood, or (4) a *p*-value from a binomial test for allele balance less that $1x10^{-10}$ (for heterozygous calls). Negative training sites were then defined as those that met one of the following criteria, excluding multi-allelic sites: (1) more than 50% of the samples with non-reference (heterozygous or homozygous) genotypes that had GQ < 20, (2) more than 50% of samples with a heterozygous genotype had variant allele fraction (VAF) below 0.2, or (3) the Hardy-Weinberg Equilibrium test *p*-value (calculated for separately for unrelated parents, siblings and probands per population, taking the largest estimate across all populations) was lower than $10^{-9}$. The positive training sites were the high confidence sites from the Broad bundle resource used in the Genome Analysis Toolkit (GATK) variant calling pipeline[27] (SNVs: Omni, Axiom, HapMap; indels: Axiom, Mills). Since these sites are enriched for common variants, we annotated an equal number of randomly selected high confidence transmitted singleton variants (seen in a single child-parent pair, with Allele Quality > 35) as positive training sites. To balance the number of positive (SNVs: 388,268; indels: 17,732) and negative (SNVs: 98,676; indels: 3,961) training

sites, the positive training set was down-sampled to match the size of the negative training set, resulting in a balanced set of 205,274 variants (SNV = 197,352, 2.8% of all SNVs, INDEL=7,922, 2.3% of all indels). The remaining sites were annotated as test sites (SNVs= 6,731,744, indels= 310,362).

We used the `ranger` package[28] in `R 4.1.0` to train random forest models on SNVs and indels separately, with 500 trees and probabilities as an output. The features used to train the random forest, and their relative importance, are depicted in Figure S3A. The training sites did not include any missing values in these features, and missing values among the test sites were set to the median of the respective feature across all sites of similar type (SNV or INDEL). The random forest model was then applied on all training and test sites to obtain probabilistic scores (range 0-1) indicating the probability of being a truly variable site. These scores were scaled to the range 0-100. The performance of the random forest was evaluated using precision and recall against the training sites using the `caret` package[29] in `R`. We then examined the fraction of retained sites from the total dataset and the transmission ratio of synonymous variants with incremental random forest cut-offs. Random Forest score cut-offs of 97 for SNVs (recall = 0.97, precision = 1) and 92 for indels (recall = 0.975, precision = 1.0) were selected, and sites with scores higher than these were retained. The transmitted/untransmitted ratio of autosomal synonymous variants was 0.5 (Figure S3B). Next, the variants were filtered to those with a minimum genotyping rate (per sequencing batch; across five sequencing batches) exceeding 95%. After this QC, 6,345,636 out of 7,273,162 sites were retained (12.8% filtered). These variants spanned 18,959 MANE-Plus transcripts (18,902 protein coding genes; including 57 genes with two transcripts).

## 1.7. Genotype filtering

We applied genotype filters based on genotype quality, allele depth, variant allele fraction, by setting the genotypes to missing if they were not consistent with the observed phred-scaled genotype likelihoods (PLs), had GQ < 10, had DP < 10, were reference genotypes with VAF > 0.25, were homozygous with VAF < 0.75, or were heterozygous with VAF < 0.2 or > 0.8.

## 1.8. Sample QC

We calculated the following sample-level metrics: total number of SNVs, total number of indels, total number of private variants (seen in one family), transition-transversion ratio, insertion-deletion ratio, and heterozygous-homozygous variants

ratio. We regressed these on the population label, four principal components and the number of sequenced samples per family, to account for the population structure and the differences in variant-calling sensitivity in related individuals. We filtered all samples that were more than six standard deviations from the mean of the residuals for any metric, removing 275 samples (AFR: 180, EUR: 55, AMR: 22, EAS: 3, SAS: 6, Unknown/Admixed: 9). Parents with autism and parents and siblings with motor delay or cognitive impairment were filtered (see 2.1). Subsequently, sets of maximally unrelated probands and siblings were selected. Note that this unrelated dataset of probands and siblings was used for the formal analysis of rare variant enrichment, and is prepared independently from the unrelated set presented in section 1.5, which was used to estimate the allele frequency for variant QC. Table S5 shows the sample size per population group after the sample QC.

# 2. Co-existing difficulties

## 2.1. Cognitive impairment in the ASC and SPARK

The ASC studies considered autistic individuals to have co-existing cognitive impairment/intellectual disability if the subject had a full-scale IQ test score < 70, if they were administered but could not complete an IQ test, or if the subject had a human phenotype ontology (HPO) term or International Classification of Diseases (ICD) code indicating intellectual disability or mental retardation.[13,30] The remaining individuals without cognitive impairment and those without information on cognitive impairment status were grouped together. To have a comparable set from SPARK for the purpose of a meta-analysis with ASC, we split autistic individuals in SPARK into those with *versus* another group combining those without cognitive impairment and those with an unknown cognitive impairment status. The phenotypic data for SPARK individuals included whether they had a reported professional diagnosis of cognitive impairment as well as binned IQ test scores. The ‘autism with cognitive impairment group included those with one of the following phenotypic entries: ‘Cognitive_impairment_latest’ = “True”, ‘dev_id’ = 1, or ‘reported_cog_test_score’ = ‘24_below’, ‘25_39’, ‘40_54’, or ‘55_69’.

## 2.2. Motor delay and cognitive impairment in SPARK

We used the following phenotypic data fields to stratify the samples based on the presence of a motor developmental disorder, cognitive impairment or an intellectual disability diagnosis:

- ‘cognitive_impairment_at_enrollment’ and ‘cognitive_impairment_latest’: provided information on cognitive impairment diagnosis (identical values). Probands with a diagnosis were coded as “True” and those without diagnosis were coded as “False”. Missing values were coded as “-”.
- 'dev_id' and 'dev_motor': provided information on cognitive impairment (Intellectual disability, cognitive impairment, global developmental delay, or borderline intellectual functioning, reported professional diagnosis) and motor development (Motor delay, e.g., delay in walking, or developmental coordination disorder; reported professional diagnosis). Diagnoses were coded as “1”. Typical development and missing values were coded as “-”.
- ‘cog_test_score’ and the ‘reported_cog_test_score’: provided binned full-scale IQ measurements (identical values). Missing values were coded as “-”.

We classified the autistic individuals in three phenotypic groups:

1. Autism with motor delay or cognitive impairment:
   ‘Cognitive_impairment_at_enrollment’ = “True, *or*
   ‘Cognitive_impairment_latest’ = “True”, *or*
   ‘dev_motor’ = 1, *or*
   ‘dev_id’ = 1, *or*
   ‘Cog_test_score’ = ‘24_below’,‘25_39’, ‘40_54’, ‘55_69’, or ‘70_79’, *or*
   ‘reported_cog_test_score’ = ‘24_below’,‘25_39’, ‘40_54’, ‘55_69’, or ‘70_79’.
2. Autism without motor or cognitive impairment:
   ‘Cognitive_impairment_at_enrollment’ = “False”, *and*
   ‘Cognitive_impairment_latest’ = “False”, *and*
   IQ bin equivalent to an IQ >= 80 or missing IQ data, *and*
   missing values in the ‘dev_motor’ field, *and*
   missing values in the ‘dev_id’ field.

The remaining probands with missing values in all fields were considered unclassified.

# 3. *De novo* and rare inherited variants in trios

As introduced in the main text, *de novo* mutations (DNMs) were studied in a total of 30,274 trios from the two cohorts by comparing DNM rates in 21,501 autistics (ASC: 8,028; SPARK: 13,473) to those seen in 8,773 siblings (ASC: 2,460; SPARK: 6,763). Over-transmission analysis of rare inherited variants was performed by evaluating the counts

of rare parental alleles transmitted to 21,043 autistic individuals (ASC: 7,570; SPARK: 13,473) *versus* untransmitted alleles. This section outlines the curation of DNMs and inherited alleles in each cohort.

## 3.1. ASC

DNMs in this dataset were obtained from a previous ASC study[13] (see 'Methods: ASC cohort'). These were curated in 9,929 trios (7,570 autistics, 2,359 siblings) as described in detail in the Supplementary Note of Ref.[13] Briefly, the `de_novo()` function from `Hail 0.2` python library[31] was used to identify candidate DNMs, with priors from population allele frequencies. These candidates were then filtered for rare alleles with internal (within ASC dataset) and external (gnomAD) allele frequencies < 0.1%, with additional filtering on genotype depth (allele balance and parent/child depth ratio), variant quality ('ExcessHet' filter and GATK variant quality score log-odds), as well as the number of candidate DNMs per sample. Additional published DNMs from 559 trios (458 probands and 101 siblings) were included, bringing the total sample size to 10,488 trios (8,028 autistics, 2,460 siblings). Transmitted and untransmitted rare parental alleles were counted in 7,570 trios (autistic probands) with available exome data.

## 3.2. SPARK

Following the variant, genotype and sample QC described in section 1 above (see 'SPARK dataset preparation'), we evaluated the genotypes of 20,236 trios (13,473 autistics and 6,763 siblings) for potential DNMs (see counts for probands and siblings in Table S1). We first filtered for candidate DNMs based on the parental genotypes and used the 'trio-dnm2' plugin from bcftools[32] 1.17 to estimate the error probability of *de novo* inheritance based on allele depth (adDNM) and genotype likelihoods (plDNM). Then for each candidate DNM, we transformed the probabilities of it being a *de novo* mutation based on these two methods to a phred-scaled error probability ($-10*\log_{10}$(minimum(plDNM,adDNM)). We then defined putative DNMs as those with phred-scaled error probability ≤ 60 having internal & gnomAD MAF < 0.1%, with an Allele Quality (AQ) score ≥ 30, and that had GQ ≥ 30 in all members of the trio. Finally, we selected one DNM per gene (worst consequence) per individual. This resulted in a dataset of 23,332 DNMs in 13,664 individuals (68% trios with DNMs, average rate of 1.15 DNMs per individual). Between 20% and 22% of the probands and siblings carried rare synonymous DNMs, compared to 23%-25% in the previous analysis of SPARK[13] (WES1). Another previous analysis of SPARK WES1 found ~ 0.20 - 0.23 synonymous DNMs per offspring,[1] suggesting that our DNM callset was of similar quality to

the published ones. To ensure that the sensitivity to identify DNMs was not biased by sample sex, we calculated the odds ratio of carrying a rare *de novo* synonymous (all autosomal genes) or protein-truncating DNM (most-constrained LOEUF decile) among male *versus* female siblings, and found that it did not differ significantly from one.

We also evaluated the transmission of rare variants (MAF < 0.1% in SPARK parents and gnomAD) in the same set of trio-sequenced autistic individuals. Similar to DNMs, we considered variants remaining after basic variant, genotype and sample QC. In instances where a single gene had multiple variants, the variant with the worst consequence was retained. We then filtered for heterozygous variants seen in one parent that had a GQ > 25 in all three samples in the trio to ensure the transmission ratio of synonymous variants was not significantly different from one.

## 3.3. Additional filtering of *de novo* mutations in SPARK

*De novo* mutations, especially damaging ones, are typically ultra-rare, usually seen in a few individuals or not seen at all in the general population. DNM calling from genotypes (see '3.2 SPARK' above) does not leverage allele frequencies as priors, and is best coupled with stringent allele frequency filtering by removing DNMs seen in more than a few individuals in the dataset or in the general population. Given that the ASC DNM dataset was prepared using a different pipeline and filtered for rare alleles (MAF < 0.1%), we adopted a similar cutoff in SPARK to have comparable call sets, thus facilitating fixed-effect meta-analysis of risk ratios. We then performed an additional analysis of ultra-rare DNMs in SPARK to ensure that the conclusions are the same in a call-set of high-confidence DNMs that favors precision over sensitivity. To define ultra-rare DNMs, we further dropped DNMs if the individual had more than one DNM in the same gene, those seen as *de novo* in more than 3 individuals in SPARK, DNMs with MAF ≥ 0.005% in SPARK parents or gnomAD, and DNMs seen in gnomAD and two of the three SPARK cohorts of unrelated individuals used for estimating allele frequency (probands, siblings, parents). This final dataset included 15,072 ultra-rare DNMs, with an average rate of 0.74 ultra-rare DNMs per sample.

## 3.4. Matching samples on principal components

As presented in the main text (Figure 2), *de novo* synonymous mutations showed a spurious association with autism when comparing autistic females without motor or cognitive impairment to sex-matched probands with motor or cognitive impairment or when compared to female siblings. This likely reflects the greater diversity among autistic females without motor or cognitive impairment (69% of samples with European genetic ancestry) relative to

the other two groups (75% of samples with European genetic ancestry in each; see Table S4). We performed an additional sensitivity analysis of rare and ultra-rare *de novo mutation* enrichment in a stringently matched set of samples to ensure that the results obtained for protein-truncating DNMs were not biased. Starting with 1,008 autistic females without cognitive impairment (`P`) and 2,596 female siblings (`S`) of European genetic ancestry (Table S5), we calculated the distance between all pairs of probands and siblings in the first four principal components (see section 1.3). Specifically, we subtracted the first four PCA eigenvectors for each proband `p` in `P` from the eigenvectors of each sibling `s` in `S`, then used the `norm(type="2")` function in `R` to calculate a Euclidean-type spectral norm representing the distance between the two samples in the PC space. We then took the minimum value for each sample in `P` and `S`, which represents the nearest neighboring sample from the opposite cohort. Samples with large values don't have any neighboring samples from the other group and are likely to be poorly-matched on ancestry. We excluded the outliers on this distance metric from the probands and siblings (> 2 median absolute deviations), leaving 868 autistic females and 2,235 siblings. See section 4.1.3 of the Supplementary Results for the outcomes of DNM enrichment analysis in this subset.

# 4. Ultra-rare variants

A substantial part of the ASC & SPARK datasets is formed of children with sequence data from one parent (N=18,816) or without any sequenced parents (N=23,114). Including these in analyses could potentially increase power. Specifically, they can be leveraged to study ultra-rare inherited alleles (when one parental exome is available) or the average effect of a mix of ultra-rare alleles of undetermined origin (case-control analysis), as detailed below. Similarly, ultra-rare variants ascertained in individuals without sequencing data from their parents ('case-control' cohorts) can be used to study the average effects of damaging *de novo* and inherited variants. Ultra-rare alleles in the parents are also well suited for determining whether the effect sizes of inherited variants differ between maternally and paternally inherited alleles (i.e. parent of origin effects) as they are already defined as 'those appearing in one parent' making it simpler to separate maternal from paternal alleles, and in addition offer the largest sample size possible by combining data across all trio- and duo-sequenced individuals.

## 4.1. Ultra-rare inherited variants in child-parent pairs

To be able to leverage the exome data from parents not sequenced as complete trios (Table S3), we studied the transmission of parental alleles in child-parent pairs, treating trios as two separate pairs rather than following the standard approach of comparing the transmission of rare variants in trios only. Studying transmission in parent-child pairs when sequence data is present from only one parent assumes the parents are not consanguineous (therefore unlikely to carry the same ultra-rare variant), and precludes the analysis of variants that are low-frequency but not extremely rare (as their transmission status cannot be determined reliably). Here, we examined ultra-rare parental alleles seen in one parent, in one family, and not seen in gnomAD. These were filtered for high-confidence calls (GQ > 32) to balance the transmission rates of synonymous variants.

To test for parent of origin effects, we ascertained ultra-rare inherited variants in ASC trios in addition to SPARK trios/duos, using a similar definition (seen in one parent in the ASC dataset and not in gnomAD). The rates of synonymous ultra-rare parental alleles were reasonably comparable between ASC & SPARK trio-sequenced probands, with an average of ~6 ultra-rare synonymous variants per parent, so we used all ultra-rare inherited variants to perform a mega-analysis (instead of a meta-analysis) of over-transmission in all 55,521 autistic-child-parent pairs.

## 4.2. Case-control variants in the ASC & SPARK

We evaluated the enrichment & liability of ultra-rare variants in the ASC case-control dataset as a supplementary analysis. This dataset contained samples from the Danish iPSYCH cohort[33] (cases = 4,863; controls = 5,002) and Swedish PAGES samples[30] (cases = 728; controls = 3,595). The processing and a previous sex-averaged analysis of these cohorts (5,591 autistic individuals and 8,597 controls) is described elsewhere.[13] Rare variants in iPSYCH were defined as those with an allele count ≤ 5 in the combined set of gnomAD non-Finnish Europeans (nonpsychiatric subset) and iPSYCH data (allele frequency ≤ 0.0043%). Rare variants in PAGES were defined as those with an allele count ≤ 5 in both ExAC r0.3 (nonpsychiatric subset; external allele frequency ≤ 0.0055%) and the autism cohort (internal allele frequency ≤ 0.014%).

For SPARK, we analyzed ultra-rare variant enrichment in the remaining individuals (6,646 probands and 2,436 siblings) who did not have sequence data from any parent (Table S5) - and as such were not included in all the previous analyses described above (*de novo* and inherited alleles). This design approximates the case-control analysis in ASC by using the siblings as controls (as done when examining the rate ratios of *de novo* mutations).

Whereas the ASC case-control dataset consisted of unrelated individuals, this SPARK sub-cohort included pairs of probands and siblings from the same family (like the trio-based analysis). We included variants seen with an allele frequency < 0.005% (in gnomAD and SPARK unrelated individuals' cohorts) that are seen in up to three individuals in the 'case-(sib)control' cohort of 9,082 individuals remaining after QC (allele frequency < 0.015%). We dropped 156 individuals with ultra-rare synonymous variants counts exceeding four median absolute deviations (> 33 variants), and compared the variant rates in the remaining 8,926 children (6,534 probands *versus* 2,392 siblings).

# 5. Measuring rare variant enrichment

## 5.1. Observed and expected variant rates

We calculated the *de novo* and inherited variant rates for synonymous, damaging missense and protein-truncating variants (most-constrained LOEUF decile) in each autism cohort (ASC, SPARK) by dividing the total number of variants by the sample size. The 95% Confidence Intervals (CI) for the variant rates (Figure S6, Figure S7, Figure S12, Figure S33) were estimated in `R` as follows:

$$95\%\ CI_{lower} = qchisq(0.025, \frac{\#\ Variants}{2 * \#\ Samples})$$

$$95\%\ CI_{upper} = qchisq(0.975, \frac{\#\ Variants + 1}{2 * \#\ Samples})$$

In the autism cohorts (ASC & SPARK), the expected variant rates were obtained from siblings or controls not diagnosed with autism (DNMs and case-control analysis) or rare untransmitted variants in the probands (inherited variants). In the NDD cohort (probands only; curated by Zhou and colleagues[2]), we compared the observed DNM counts to the expected DNM counts from a mutational model from gnomAD[23] (v2.1.1). To obtain exome-wide *de novo* mutation rates, we summed gene-level mutation rates across 17,296 autosomal protein-coding genes considered in our work. The expected rate of protein-truncating DNMs was ~0.05 (~0.0095 in the 1$^{st}$ LOEUF decile); for missense variants, we expect ~0.64 DNMs per child. To estimate the expected rate of damaging missense DNMs (MPC score ≥ 2), we explored the fraction of missense variants with MPC score ≥ 2 from missense variants identified in 9,223 trio-sequenced siblings not diagnosed with autism (included in the analysis presented in Figure 1). We find that ~4.8% of all missense DNMs in the ASC cohort and ~4.98% in SPARK have an MPC score ≥ 2. The corresponding fraction analyzed in another study[2] was 4.50% (largely overlapping samples).

We decided to use an estimate of 5% (rounding these fractions), corresponding to an expected rate of ~0.032 damaging missense DNMs per child.

The observed synonymous mutation rate in the total cohort (~0.28 per child) was significantly higher (p = 0.00076; binomial test) than the mutation rate from the gnomAD mutation model (~0.27). We therefore calibrated the damaging mutation rates to account for potential underlying heterogeneity between the sequencing cohorts (e.g., differences in capture targets & pipelines). For each sequencing cohort, we calculated the observed/expected synonymous DNM rate ratio (*versus* gnomAD model) (GeneDX= 1.01, DDD=1.09, RUMC = 0.97, DEE=0.9, ID=1.05); the mutation rates calculated from gnomAD model were then multiplied by these ratios to obtain calibrated mutation rates for each sequencing cohort. We then calculated the expected number of DNMs from a poisson distribution:

$$Expected\ DNM\ count\ =\ sum(rpois(n\ =\ N, lambda\ =\ mu\ *\ k)$$

Where `N` is the sample size (per sequencing cohort, per sex), `mu` is the mutation rate (from the gnomAD model), and `k` is the scaling factor (observed/expected synonymous rate). (`rpois` is an `R` function from `stats` package that generates random samples from a poisson distribution with a given rate parameter `lambda`). We repeated this procedure 5,000 times and took the median expected count. The counts were then summed across all sub-cohorts to get the expected DNM counts for 17,698 males and 13,867 females.

## 5.2. Sex-stratified comparisons

### 5.2.1. De novo mutations

We used the ratio between the rate of DNMs in the probands and the siblings (or female and male probands in direct comparisons) to evaluate the enrichment of a specific class of DNMs in a gene set or exome-wide:

$$DNM\ rate\ ratio\ =\ \frac{\#DNM\ in\ probands\ /\ \#\ probands}{\#\ DNM\ in\ siblings\ /\ \#\ siblings}$$

To test for significance, we compared the counts DNMs in the probands to the counts in their siblings in `R`:

```
binom.test(x= #DNMs_in_probands, n= (#DNMs_in_probands +
#DNMs_in_siblings), p= #probands/(#probands + #siblings),
alternative="two.sided")
```

We note that the measure used to test the relative enrichment in the binomial test is the ***fraction*** of DNMs in ***probands*** among ***all*** DNMs.

$$Fraction = \frac{\#DNM\ in\ probands}{\#\ DNM\ in\ siblings} = \frac{\#DNM\ in\ probands}{\#\ Total\ DNMs - \#\ DNMs\ in\ probands}$$

It is different from the DNM ***rate ratio*** (a risk ratio) between the ***probands*** and ***siblings***.

$$Rate\ Ratio = \frac{\#DNM\ in\ probands\ *\ \#\ siblings}{\#\ DNM\ in\ siblings\ *\ \#\ probands}$$

Here, we report the enrichment as rate ratios as these are easy to interpret and can be used for DNMs, inherited and case-control variants to express fold-enrichment. It is possible to convert between the rate ratio and fraction as follows:

$$Rate\ Ratio = \frac{\#DNM\ in\ probands\ *\ \#\ siblings}{(\#\ Total\ DNMs - \#\ DNM\ in\ probands) *\ \#\ probands}$$

$$Rate\ Ratio = \frac{\#DNM\ in\ probands\ *\ \#\ siblings}{(\#\ Total\ DNMs *\ \#\ probands) - (\#\ DNM\ in\ probands *\ \#\ probands)}$$

$$Rate\ Ratio = \frac{(\#DNM\ in\ probands\ /\ \#\ Total\ DNMs)\ *\ \#\ siblings}{\#\ probands - (\#\ DNM\ in\ probands/\ \#\ Total\ DNMs) *\ \#\ probands}$$

$$Rate\ Ratio = \frac{(\#DNM\ in\ probands\ /\ \#\ Total\ DNMs)\ *\ \#\ siblings}{(1 - (\#\ DNM\ in\ probands/\ \#\ Total\ DNMs)\ )\ *\ \#\ probands}$$

$$Rate\ Ratio = \frac{Fraction *\ \#\ siblings}{(1 - Fraction\ )\ *\ \#\ probands}$$

Although we calculated the rate ratios directly from the observed counts, the above equation is useful for estimating the confidence intervals with the same test used to calculate the *p*-values; the binomial test returns the upper and lower bounds of the 95% CI of the observed fraction. We converted these bounds to equivalent rate ratios by plugging them into this formula:

$$DNM\ ratio\ CI = \frac{Fraction\ CI_{binomial\ test} * \#\ siblings}{(1 - Fraction\ CI_{binomial\ test}) * \#\ probands}$$

### 5.2.2. Over-transmission

To assess over-transmission in the probands, we calculated the ratio between transmitted untransmitted parental alleles:

$$Transmitted/\ Untransmitted\ ratio = \frac{\#\ parental\ alleles\ transmitted\ to\ the\ probands}{\#\ untransmitted\ parental\ alleles}$$

We then compared the significance of the difference in the counts of transmitted and untransmitted alleles:

```
binom.test(
x=#variants_transmitted_to_probands,
n=#variants_transmitted_to_probands + #untransmitted_variants,
p=0.5,alternative=”two.sided”)
```

We scaled the 95% CIs from the binomial test as follows:

$$Tr./\ Ut.\ ratio\ CI = \frac{Fraction\ CI_{binomial\ test}}{1 - Fraction\ CI_{binomial\ test}}$$

For ultra-rare inherited variants, this was used to test all parental alleles (maternal+paternal) as well as maternal and paternal alleles separately.

### 5.2.3. Case-control comparisons

Variants were compared between cases and controls in a similar manner to DNMs, i.e., using the sample size to derive the expected fraction of variants in the cases. These case-control comparisons are more sensitive to differences in population architecture than comparisons of *de novo* mutations and transmitted-untransmitted alleles. When testing the enrichment of ultra-rare variants in cases versus controls in a regression framework, synonymous variant counts can be used as a covariate along with principal components. When using a binomial test, the expected variant rates can be adjusted for population differences by using the synonymous variants. Therefore, the damaging variant comparisons were additionally adjusted by the synonymous variant counts as follows:

$$Case/Control\ rate\ ratio\ = \frac{\#\ damaging\ variants\ in\ cases\ /\ \#\ synonymous\ variants\ in\ cases}{\#\ damaging\ variants\ in\ controls\ /\ \#\ synonymous\ variants\ in\ controls}$$

```
binom.test(x= #damaging_in_cases, n= (#damaging_in_cases +
#damaging_in_ctrls), p= #syn_in_cases/(#syn_in_cases +
#syn_in_ctrls), alternative=”two.sided”)
```

$$Case/Ctrl\ CI\ = \frac{Fraction\ CI_{binomial\ test} * \#synonymous\ in\ controls}{(1 - Fraction\ CI_{binomial\ test}) * \#synonymous\ in\ cases}$$

Additionally, we calculated the rates of all ultra-rare parental alleles (transmitted & untransmitted) across all mothers and fathers in these child-parent pairs, then compared the variant rates in the mothers to the corresponding rates in the fathers using a binomial test. As these are similar to case-control analyses, we used synonymous variant counts as a reference point.

$$Mothers/Fathers\ rate\ ratio\ = \frac{\#\ damaging\ variants\ in\ mothers\ /\ \#\ synonymous\ variants\ in\ mothers}{\#\ damaging\ variants\ in\ fathers\ /\ \#\ synonymous\ variants\ in\ mothers}$$

```
binom.test(x= #damaging_in_mothers, n= (#damaging_in_mothers +
#damaging_in_fathers), p= #syn_in_mothers/(#syn_in_mothers +
#syn_in_fathers), alternative=”two.sided”)
```

$$Mothers/Fathers\ CI\ = \frac{Fraction\ CI_{binomial\ test} * \#synonymous\ in\ mothers}{(1 - Fraction\ CI_{binomial\ test}) * \#synonymous\ in\ fathers}$$

## 5.3. Sex differences in enrichment

Sex differences were assessed by comparing autistic females to autistic males in the same manner as we compared autistic individuals to their siblings. Rate ratios > 1 indicate higher variant rates in autistic females compared to autistic males. To test for significant differences in de novo mutation counts, we compared the DNM counts in females and males:

```
binom.test(x= #DNMs_in_females,
n= (#DNMs_in_males + #DNMs_in_females),
p= #females/(#males + #females), alternative="two.sided")
```

$$DNM\ ratio\ CI = \frac{Fraction\ CI_{binomial\ test} * \#\ Females}{(1 - Fraction\ CI_{binomial\ test}) * \#\ Males}$$

Case-control variants were tested in the same manner as DNMs. For inherited variants, we compared the counts of parental alleles transmitted to females and males:

```
binom.test(x=#variants_transmitted_to_females,
n= #variants_transmitted_to_males_or_females,
p= #all_parental_alleles_in_females/#all_parental_alleles,
alternative="two.sided")
```

$$Tr./Ut.\ ratio\ CI = \frac{Fraction\ CI_{binomial\ test} * \#\ parental\ alleles\ in\ females}{(1 - Fraction\ CI_{binomial\ test}) * \#\ parental\ alleles\ in\ males}$$

This was also used to test for sex differences in over-transmission of ultra-rare maternal or paternal alleles (i.e. transmission of maternal alleles to daughters *versus* to sons, paternal alleles to daughters *versus* to sons), thus testing indirectly for parent of origin effects, which would appear as significant sex differences in over-transmission from one group of parents but not the other. To examine parent of origin effects directly (maternal *versus* paternal alleles), we used the following scheme (this example shows how to test maternal *versus* paternal transmission to daughters):

```
binom.test(
x= #variants_transmitted_from_mothers_to_daughters,
n= #variants_transmitted_from_mothers_to_daughters+
   #variants_transmitted_from_fathers_to_daughters,
p= #transmitted_and_untransmitted_maternal_variants/
  (#transmitted_and_untransmitted_maternal_variants+
   #transmitted_and_ultransmitted_paternal_variants),
alternative="two.sided")
```

$$Tr./Ut.\ ratio\ CI\ =\ \frac{Fraction\ CI_{binomial\ test} * \#\ maternal\ alleles}{(1 - Fraction\ CI_{binomial\ test}) * \#\ paternal\ alleles}$$

## 5.4. Meta-analysis between ASC & SPARK cohorts

We performed a fixed-effect inverse-variance-weighted meta-analysis of the risk ratios (rate ratios or transmitted/untransmitted ratios) in ASC & SPARK using `metagen` function from `meta` package[34] in `R`:

```
metagen(sm = "RR",
      fixed = TRUE,
      studlab = Cohort,
      TE = Risk_Ratio,
      level.ci = 0.95,
      lower = Risk_Ratio_CIL,
      upper = Risk_Ratio_CIU,
      pval = Risk_Ratio_Pval,
      method.tau = "PM")
```

## 5.5. Correcting for multiple testing

We adjusted the p-values from each experiment (e.g. 54 tests when performing exome-wide enrichment testing) for the Family-wise (Experiment-wise) Error Rate using Bonferroni correction. We also performed more lenient adjustment for False Discovery Rate using Benjamini-Hochberg method. Both were performed in R:

```
p.adjust(method='bonferroni')
p.adjust(method='BH')
```

# 6. Measuring effect sizes on the liability scale

## 6.1. Standard (Differing Thresholds) Liability Threshold Model

We compared the change in trait liability for each variant class to reflect the deviation from population mean liability that would result from carrying a variant in that class. The relative differences in variant frequency between the probands and siblings was converted to Z scores on the liability scale, assuming the liability is normally distributed and centered around zero in the general population, with differing thresholds in males and females (Figure

S5A). The mathematical derivation is explained in Ref[30]. Here, we summarize the concept behind this procedure for those less familiar with the statistical concepts:

- In a Liability Threshold Model assuming additive genetic risk that is normally distributed in the population, the threshold is the point that forms the boundary of an area under the normal distribution curve that is equivalent to the trait prevalence (Figure S5A). For example, an autism prevalence of 0.025 indicates that the distance between the population mean risk and the threshold is 1.96 standardized units, putting 2.5% of the population in the right tail.
- We can work out the distance (in standardized units) between the population mean (zero, given how the model is defined) and the threshold using the inverse of the standard normal cumulative distribution function ($\Phi^{-1}$), or '*norminv*' in short. The threshold in the population equals the average liability in the population (i.e., zero) + *norminv*(Prevalence) as shown in Figure S5A. The inverse normal function $\Phi^{-1}$ is implemented in `R` using the function `qnorm`, which returns the lower tail by default, whereas the prevalence reflects the upper tail (fraction of individuals *above* the threshold). We can get the upper tail by passing an argument to the function ('`qnorm(Prevalence,lower.tail=FALSE)`) or simply by using the complement of the prevalence, `qnorm(1 - Prevalence).`
  - Now we move to the individuals harboring a certain class of variants, for instance damaging PTVs. The liability in this sub-population can also be approximated by a normal distribution, and the mean of this distribution will be the *average PTV liability.* This average variant liability is a measure of the average effect size of PTVs on the liability scale. Individuals harboring PTVs who are autistic will form the upper tail above a certain threshold, and non-autistic individuals harboring PTVs will be in the lower tail (Figure S5A).
  - Whereas the threshold in the population liability distribution reflects the prevalence in the population, the threshold in the PTV liability distribution will reflect autism *prevalence among the individuals harboring PTVs* in the general population. In accordance with the original derivation in ref,[34] we refer to this fraction as the 'penetrance'.
  - Similar to the prevalence, the penetrance is a cumulative density and as such can be converted to equivalent standardized units in the PTV liability distribution using the inverse normal cumulative density distribution function ($\Phi^{-1}$). The threshold here equals the (unknown) average PTV liability + *norminv*(Penetrance). We can get this is `R` using `qnorm(1 - Penetrance).`

- By leveraging the fact that the threshold in both distributions is the same, we can now estimate the distance between the population mean (zero) and the (unknown) mean protein-truncating variant liability. As shown in Figure S5A, we can write:
  threshold = `qnorm(1 - Prevalence)` = *average PTV liability* + `qnorm(1 - Penetrance)`
- The prevalence in the general population is a known parameter. Autism is diagnosed in 1 in 40 male individuals (a prevalence of 2.5% in males), with a 1:4 male-to-female ratio (prevalence in females = 0.25 x 2.5% = 0.625%). Previous estimates suggested that about one third of autistic individuals in the population have profound deficits with cognitive impairment [35,36]. Among 11,630 autistic individuals in SPARK who could be classified, ~ 36% fell in the autism with motor or cognitive impairment group (35% in males & 40% in females). For estimating liability, we scaled the sex-specific population prevalence using these percentages, i.e., using a prevalence estimate of 0.88% in males (35% x 2.5%) and 0.25% in females (40% x 0.625%) for liability calculations in the autism with motor or cognitive impairment group; for the autism without motor or cognitive impairment group, we used a prevalence of 1.62% in males (2.5% - 0.88%) and 0.38% in females (0.625% - 0.25%). For directly comparing those with motor or cognitive impairment to those without these co-occurring difficulties, we used a prevalence of 0.4 in females and 0.35 in males.
- The prevalence among the individuals harboring PTVs, on the other hand, is not known. We can, however, convert the variant rates in the study cohort to population estimates, and convert these population estimates to penetrance estimates. Specifically, the penetrance is the ratio between PTV rate among autistic individuals and the overall frequency of PTVs in the population. The overall population frequency of PTVs can, in turn, be estimated from the study cohort, namely by summing the frequency of PTVs in the probands weighed (multiplied) by their fraction in the population (trait prevalence) and the frequency of PTVs in the siblings weighed by their relative fraction as well (1 minus the trait prevalence).
- Now that we have the prevalence estimates both in the general population and among individuals harboring PTVs (penetrance), we estimate the average PTV liability as `qnorm(1 - Prevalence)- qnorm(1 - Penetrance)`.

- The liability stratified by sex was measured in `R` using these formulas:

$$Rate_{Population} = Prevalence * Rate_{Probands} + (1 - Prevalence) * Rate_{Siblings}$$

$$Penetrance = \frac{Rate_{Probands}}{Rate_{Population}} * Prevalence$$

$$Z = qnorm(1 - Prevalence) - qnorm(1 - Penetrance)$$

- The *p*-values were those obtained from the binomial tests detailed above. The standard error, and then the confidence intervals around Z, were estimated from these *p*-values:

  $$Q = qnorm(1 - \frac{Binomial\ test\ P\ value}{2})$$

  $$SE = \frac{abs(Z)}{Q}$$

  $$95\%\ CI = Z \pm (1.96 * SE)$$

- The difference between males and females liability estimates was measured as follows:

  $$Z_{Difference} = Z_{Females} - Z_{Males}$$

  $$SE_{Difference} = sqrt(SE^2_{Females} + SE^2_{Males})$$

  $$Q_{Difference} = \frac{abs(Z_{Difference})}{SE_{Difference}}$$

  $$P_{Difference} = 2 * (1 - pnorm(Q_{Difference}))$$

- To test for parent of origin effects, we calculated the difference between the effect sizes of paternal or maternal alleles transmitted to female or male children (daughters/sons).

- The inverse-variance-weighted average from both sexes and its confidence interval was measured as follows:

  $$Z_{Both} = (\frac{Z_{Females}}{SE^2_{Females}} + \frac{Z_{Males}}{SE^2_{Males}}) \div (\frac{1}{SE^2_{Females}} + \frac{1}{SE^2_{Males}})$$

  $$SE_{Both} = 1 \div sqrt(\frac{1}{SE^2_{Females}} + \frac{1}{SE^2_{Males}})$$

  $$95\%\ CI_{Both} = Z_{Both} \pm (1.96 * SE_{Both})$$

- The meta-analysis *p*-value was obtained using Fisher's method implemented in the `metap` package[34]:

$$P_{Both} = metap::sumlog(P_{Females}, P_{Males})$$

- The *p*-values for each experiment were corrected for Family-wise Error Rate (Bonferroni correction) and False Discovery Rate (Benjamini-Hochberg correction), as shown for the risk ratios.

## 6.2. Differing Variance Liability Model

The sex difference in a trait prevalence can be explained by the two sexes having differing variances instead of differing thresholds or means.[37] When the parameters of the liability distribution vary between the sexes, the inverse cumulative density function with a given mean and standard deviation ($\Phi_{\mu,\sigma}^{-1}$) is used to determine the threshold (θ) from the prevalence. Given that the variance of the distribution is different for the two sexes, the scale is also different, and the threshold will be in Z score units relative to the standard deviation of the underlying distribution ($\theta_m$ & $\theta_f$). However, when we model the difference in prevalence as the outcome of differences in variance alone, the mean and the threshold are the same in both sexes. This entails that the distance between the mean, which can be considered the reference point (zero), and the threshold ($\theta_m$ & $\theta_f$) is equal on both scales ($\theta = \theta_m = \theta_f$), and can in turn be used as a measuring unit across all scales. In other words, we can express the effect sizes as fractions of the threshold by dividing the measured variant liability in one sex by the threshold for in its scale. This in turn allows the projection of the effect sizes from one scale with a certain standard deviation to another scale with different standard deviation by multiplying the fraction by the threshold of the second scale.

To project the effect sizes from the two liability distributions with prevalence $\psi_m$ & $\psi_f$ and standard deviations $\sigma_m$ & $\sigma_f$ to a population scale with with a given prevalence (ψ) and standard deviation = 1, we did the following in `R`:

- We estimated the threshold on each scale (population distribution with variance = $\sigma^2$, males with variance = $\sigma_m^2$, females with variance = $\sigma_f^2$, mean = 0 on all scales):

$$\theta = qnorm(1 - \psi, sd = 1)$$

$$\theta_m = qnorm(1 - \psi_m, sd = \sigma_m)$$

$$\theta_f = qnorm(1 - \psi_f, sd = \sigma_f)$$

- As shown in section 6.1 above, we estimated the prevalence of autism among those harboring damaging variants in the population ('penetrance') in males ($\rho_m$) and females ($\rho_f$) and then the corresponding deviation from the threshold:

$$\vartheta_m = qnorm(1 - \rho_m, sd = \sigma_m)$$

$$\vartheta_f = qnorm(1 - \rho_f, sd = \sigma_f)$$

- The liability is the difference between these two Z scores (Figure S5A), however these are on different scales relative to their standard deviation (Figure S5B). To normalize the scores, we divide them by the corresponding threshold on each scale ($\theta_m$ or $\theta_f$), which means that they will be expressed as a fraction of the threshold. The threshold is the same across all scales. To have both effect sizes on a unified population scale, we multiply these fractions by the threshold ($\theta$):

$$Z_m = (\theta_m - \vartheta_m) * \theta \div \theta_m = \theta - \vartheta_m * \theta \div \theta_m$$

$$Z_f = (\theta_f - \vartheta_f) * \theta \div \theta_f = \theta - \vartheta_f * \theta \div \theta_f$$

# 7. Gene set enrichment

## 7.1. Gene sets

The Simons Foundation Autism Research Initiative (SFARI) database provides curated scores for 1,172 genes that reflect the strength of evidence linking each gene to autism risk.[38] There are 3,58 autosomal and 47 X-linked SFARI Category 1 (high-confidence) & S (syndromic) genes with strong evidence of association with autism (354 autosomal genes included in the current analysis cohort after QC). We tested the enrichment of DNMs and rare inherited variants after removing these genes as well as in this gene set only (216 in the 1st LOEUF decile, 62 in the 2nd decile, 24 in the 3rd decile).

Genes with sex-biased expression in the fetal cortex were obtained from a recent study by Kissel and and colleagues,[10] who meta-analyzed two large fetal brain bulk RNA-seq datasets: BrainVar project (dorsolateral prefrontal cortex) and UCLA dataset (cortex). The study identified 101 significant genes with FDR < 0.1 from a meta-analysis of two datasets but, according to the study, these genes did not overlap known autism predisposition genes. To maximize sensitivity in our analysis, we used the data presented by the authors in Table S4 (see the Supplements of Ref[10]) to create a list of genes with similar direction of effects in the two datasets (t_UCLA * t_BV > 0) that achieved nominal significance in the meta-analysis (invNorm_rawPval < 0.05), then split these into female-biased genes (t_UCLA < 0 & t_BV < 0) and male-biased genes (t_UCLA > 0 & t_BV > 0). These genes were filtered further for protein-coding autosomal genes that were included in our analysis, resulting in

117 female-biased (8 in the 1st LOEUF decile, 15 in the 2nd decile, 11 in the 3rd decile) and 305 male-biased genes (39 in 1st LOEUF decile, 49 in the 2nd decile, 42 in the 3rd decile).

Genes with significant sex-biased expression in the adult human cortex were obtained from a recent study by Fass and colleagues.[11] They examined two large collections of postmortem brain RNA sequencing data, the Genotype-Tissue Expression project (GTEx, cortex) and the CommonMind Consortium (CMC, frontocortical samples), producing a meta-analyzed set of sex-differential expression. Similar to the strategy outlined above, we used the results of the meta-analysis presented by the authors in Supplemental Table 4 (see the Supplements of Ref[11]) to create a list of genes that had the same direction of effect in GTEx and CMC and a nominally significant combined P-value (pvalue < 0.05). The gene sets were composed of 2,427 female-biased (381 in the 1st LOEUF decile, 344 in the 2nd decile, 319 in the 3rd decile) and 2,852 male-biased genes (483 in 1st LOEUF decile, 443 in the 2nd decile, 416 in the 3rd decile).

## 7.2. Gene set enrichment versus matched genes

For each tested gene set, we selected a matched set (see 7.3 below) and counted DNMs, transmitted and untransmitted variants; we repeated this procedure 10,000 times with replacement and took the average ratio (rate ratio between DNM counts in probands and siblings or transmitted to untransmitted ratio in the probands); we then used this ratio as the expected ratio in a binomial test as described above. Specifically, we tested the difference between the rate of DNMs between probands and siblings against the permutation-averaged expected ratio for this gene set (instead of the sample size ratio used in the exome-wide analyses), and similarly tested rare variant over-transmission against the permutation-averaged expected transmitted-to-untransmitted ratio for the given gene set (instead of 0.5 as used in the exome-wide analysis). We also used the average variant rates across these 10,000 permutations instead of the rate in siblings to estimate the variant liability attributed to a gene set in excess of what is expected for matched genes.

## 7.3. Selecting random sets of matching genes

We followed a procedure similar to that previously used by Ouwenga and Dougherty[39] to select sets of control genes for gene set burden analysis. We used a multi-dimensional kernel density estimator (KDE) from the `ks` package[40] in `R` to select these control genes. First, we created a table of features (coding length, brain expression level, and LOEUF bins) and used it to build a 3D KDE. Next, we used the test gene set to build a density distribution and evaluated the remaining genes (not in the gene set) on this

distribution. We then evaluated these remaining genes on a density distribution built using all genes rather than the gene set genes. The ratio of the two estimates was then used as a sampling weight. Here we show the `R` code used to implement this:

```
kde_set_genes = ks::kde(
                x = features_set_genes,
                eval.points = features_remaining_genes
                )
                # feature_x_genes: table gene x length, expr., loeuf
kde_all_genes = ks::kde(
                x = features_all_genes,
                eval.points = features_remaining_genes
                )
kde_weights =  kde_set_genes$estimate ÷ kde_all_genes$estimate
genes_random_idx = sample(
                1:length(remaining_genes),
                length(set_genes),
                prob =kde_weights
                )
genes_randoms = remaining_genes[genes_random_idx]
```

# 8. Supplemental References


1. Antaki, D., Guevara, J., Maihofer, A.X., Klein, M., Gujral, M., Grove, J., Carey, C.E., Hong, O., Arranz, M.J., Hervas, A., et al. (2022). A phenotypic spectrum of autism is attributable to the combined effects of rare variants, polygenic risk and sex. Nat. Genet. *54*, 1284–1292. https://doi.org/10.1038/s41588-022-01064-5.
2. Zhou, X., Feliciano, P., Shu, C., Wang, T., Astrovskaya, I., Hall, J.B., Obiajulu, J.U., Wright, J.R., Murali, S.C., Xu, S.X., et al. (2022). Integrating de novo and inherited variants in 42,607 autism cases identifies mutations in new moderate-risk genes. Nat. Genet. *54*, 1305–1319. https://doi.org/10.1038/s41588-022-01148-2.
3. Wang, T., Kim, C.N., Bakken, T.E., Gillentine, M.A., Henning, B., Mao, Y., Gilissen, C., The SPARK Consortium, Nowakowski, T.J., Eichler, E.E., et al. (2022). Integrated gene analyses of de novo variants from 46,612 trios with autism and developmental disorders. Proc. Natl. Acad. Sci. *119*, e2203491119. https://doi.org/10.1073/pnas.2203491119.
4. Jacquemont, S., Coe, B.P., Hersch, M., Duyzend, M.H., Krumm, N., Bergmann, S., Beckmann, J.S., Rosenfeld, J.A., and Eichler, E.E. (2014). A Higher Mutational Burden in Females Supports a “Female Protective Model” in Neurodevelopmental Disorders. Am. J. Hum. Genet. *94*, 415–425. https://doi.org/10.1016/j.ajhg.2014.02.001.
5. Zhao, X., Leotta, A., Kustanovich, V., Lajonchere, C., Geschwind, D.H., Law, K., Law, P., Qiu, S., Lord, C., Sebat, J., et al. (2007). A unified genetic theory for sporadic and inherited autism. Proc. Natl. Acad. Sci. U. S. A. *104*, 12831–12836. https://doi.org/10.1073/pnas.0705803104.
6. Krumm, N., Turner, T.N., Baker, C., Vives, L., Mohajeri, K., Witherspoon, K., Raja, A., Coe, B.P., Stessman, H.A., He, Z.-X., et al. (2015). Excess of rare, inherited truncating mutations in autism. Nat. Genet. *47*, 582–588. https://doi.org/10.1038/ng.3303.
7. Iossifov, I., Levy, D., Allen, J., Ye, K., Ronemus, M., Lee, Y.-H., Yamrom, B., and Wigler, M. (2015). Low load for disruptive mutations in autism genes and their biased transmission. Proc. Natl. Acad. Sci. U. S. A. *112*, E5600-5607. https://doi.org/10.1073/pnas.1516376112.
8. Cui, W., Xue, H., Wei, L., Jin, J., Tian, X., and Wang, Q. (2021). High heterogeneity undermines generalization of differential expression results in RNA-Seq analysis. Hum. Genomics *15*, 7. https://doi.org/10.1186/s40246-021-00308-5.
9. Sturm, G., List, M., and Zhang, J.D. (2021). Tissue heterogeneity is prevalent in gene expression studies. NAR Genomics Bioinforma. *3*, lqab077. https://doi.org/10.1093/nargab/lqab077.
10. Kissel, L.T., Pochareddy, S., An, J.-Y., Sestan, N., Sanders, S.J., Wang, X., and Werling, D.M. (2024). Sex-Differential Gene Expression in Developing Human Cortex and Its Intersection With Autism Risk Pathways. Biol. Psychiatry Glob. Open Sci. *4*, 100321. https://doi.org/10.1016/j.bpsgos.2024.100321.
11. Fass, S.B., Mulvey, B., Chase, R., Yang, W., Selmanovic, D., Chaturvedi, S.M., Tycksen, E., Weiss, L.A., and Dougherty, J.D. (2024). Relationship between sex biases in gene expression and sex biases in autism and Alzheimer’s disease. Biol. Sex Differ. *15*, 47. https://doi.org/10.1186/s13293-024-00622-2.
12. Wingo, A.P., Liu, Y., Gerasimov, E.S., Vattathil, S.M., Liu, J., Cutler, D.J., Epstein, M.P., Blokland, G.A.M., Thambisetty, M., Troncoso, J.C., et al. (2023). Sex differences in brain protein expression and disease. Nat. Med. *29*, 2224–2232. https://doi.org/10.1038/s41591-023-02509-y.
13. Fu, J.M., Satterstrom, F.K., Peng, M., Brand, H., Collins, R.L., Dong, S., Wamsley, B., Klei, L., Wang, L., Hao, S.P., et al. (2022). Rare coding variation provides insight into the genetic architecture and phenotypic context of autism. Nat. Genet. *54*, 1320–1331. https://doi.org/10.1038/s41588-022-01104-0.

14. Deciphering Developmental Disorders Study, Kaplanis, J., Samocha, K.E., Wiel, L., Zhang, Z., Arvai, K.J., Eberhardt, R.Y., Gallone, G., Lelieveld, S.H., Martin, H.C., et al. (2020). Evidence for 28 genetic disorders discovered by combining healthcare and research data. Nature *586*, 757–762. https://doi.org/10.1038/s41586-020-2832-5.
15. Appenzeller, S., Balling, R., Barisic, N., Baulac, S., Caglayan, H., Craiu, D., De Jonghe, P., Depienne, C., Dimova, P., Djémié, T., et al. (2014). De Novo Mutations in Synaptic Transmission Genes Including DNM1 Cause Epileptic Encephalopathies. Am. J. Hum. Genet. *95*, 360–370. https://doi.org/10.1016/j.ajhg.2014.08.013.
16. Rauch, A., Wieczorek, D., Graf, E., Wieland, T., Endele, S., Schwarzmayr, T., Albrecht, B., Bartholdi, D., Beygo, J., Di Donato, N., et al. (2012). Range of genetic mutations associated with severe non-syndromic sporadic intellectual disability: an exome sequencing study. Lancet Lond. Engl. *380*, 1674–1682. https://doi.org/10.1016/S0140-6736(12)61480-9.
17. De Ligt, J., Willemsen, M.H., Van Bon, B.W.M., Kleefstra, T., Yntema, H.G., Kroes, T., Vulto-van Silfhout, A.T., Koolen, D.A., De Vries, P., Gilissen, C., et al. (2012). Diagnostic Exome Sequencing in Persons with Severe Intellectual Disability. N. Engl. J. Med. *367*, 1921–1929. https://doi.org/10.1056/NEJMoa1206524.
18. Wright, C.F., Fitzgerald, T.W., Jones, W.D., Clayton, S., McRae, J.F., van Kogelenberg, M., King, D.A., Ambridge, K., Barrett, D.M., Bayzetinova, T., et al. (2015). Genetic diagnosis of developmental disorders in the DDD study: a scalable analysis of genome-wide research data. The Lancet *385*, 1305–1314. https://doi.org/10.1016/S0140-6736(14)61705-0.
19. Loomes, R., Hull, L., and Mandy, W.P.L. (2017). What Is the Male-to-Female Ratio in Autism Spectrum Disorder? A Systematic Review and Meta-Analysis. J. Am. Acad. Child Adolesc. Psychiatry *56*, 466–474. https://doi.org/10.1016/j.jaac.2017.03.013.
20. Foster, I. (2011). Globus Online: Accelerating and Democratizing Science through Cloud-Based Services. IEEE Internet Comput. *15*, 70–73. https://doi.org/10.1109/MIC.2011.64.
21. Chorbadjiev, L., Cokol, M., Weinstein, Z., Shi, K., Fleisch, C., Dimitrov, N., Mladenov, S., Xu, S., Hall, J., Ford, S., et al. (2024). The Genotype and Phenotypes in Families (GPF) platform manages the large and complex data at SFARI (Bioinformatics) https://doi.org/10.1101/2024.02.08.579330.
22. McLaren, W., Gil, L., Hunt, S.E., Riat, H.S., Ritchie, G.R.S., Thormann, A., Flicek, P., and Cunningham, F. (2016). The Ensembl Variant Effect Predictor. Genome Biol. *17*, 122. https://doi.org/10.1186/s13059-016-0974-4.
23. Genome Aggregation Database Consortium, Karczewski, K.J., Francioli, L.C., Tiao, G., Cummings, B.B., Alföldi, J., Wang, Q., Collins, R.L., Laricchia, K.M., Ganna, A., et al. (2020). The mutational constraint spectrum quantified from variation in 141,456 humans. Nature *581*, 434–443. https://doi.org/10.1038/s41586-020-2308-7.
24. The 1000 Genomes Project Consortium (2015). A global reference for human genetic variation. Nature *526*, 68–74. https://doi.org/10.1038/nature15393.
25. Chang, C.C., Chow, C.C., Tellier, L.C., Vattikuti, S., Purcell, S.M., and Lee, J.J. (2015). Second-generation PLINK: rising to the challenge of larger and richer datasets. GigaScience *4*, 7. https://doi.org/10.1186/s13742-015-0047-8.
26. Manichaikul, A., Mychaleckyj, J.C., Rich, S.S., Daly, K., Sale, M., and Chen, W.-M. (2010). Robust relationship inference in genome-wide association studies. Bioinformatics *26*, 2867–2873. https://doi.org/10.1093/bioinformatics/btq559.
27. Van der Auwera, G.A., Carneiro, M.O., Hartl, C., Poplin, R., del Angel, G., Levy-Moonshine, A., Jordan, T., Shakir, K., Roazen, D., Thibault, J., et al. (2013). From FastQ Data to High-Confidence Variant Calls: The Genome Analysis Toolkit Best Practices Pipeline: The Genome Analysis Toolkit Best Practices Pipeline. In Current Protocols in Bioinformatics, A. Bateman, W. R. Pearson, L. D. Stein, G. D. Stormo, and J. R. Yates, eds. (John Wiley & Sons, Inc.), p. 11.10.1-11.10.33.
28. Wright, M.N., and Ziegler, A. (2017). **ranger** : A Fast Implementation of Random Forests for High Dimensional Data in *C++* and *R*. J. Stat. Softw. *77*.

https://doi.org/10.18637/jss.v077.i01.
29. Kuhn, M. (2008). Building Predictive Models in *R* Using the **caret** Package. J. Stat. Softw. *28*. https://doi.org/10.18637/jss.v028.i05.
30. Satterstrom, F.K., Kosmicki, J.A., Wang, J., Breen, M.S., De Rubeis, S., An, J.-Y., Peng, M., Collins, R., Grove, J., Klei, L., et al. (2020). Large-Scale Exome Sequencing Study Implicates Both Developmental and Functional Changes in the Neurobiology of Autism. Cell *180*, 568-584.e23. https://doi.org/10.1016/j.cell.2019.12.036.
31. Hail Team (2022). Hail. Version 0.2.
32. Danecek, P., Bonfield, J.K., Liddle, J., Marshall, J., Ohan, V., Pollard, M.O., Whitwham, A., Keane, T., McCarthy, S.A., Davies, R.M., et al. (2021). Twelve years of SAMtools and BCFtools. GigaScience *10*, giab008. https://doi.org/10.1093/gigascience/giab008.
33. Satterstrom, F.K., Walters, R.K., Singh, T., Wigdor, E.M., Lescai, F., Demontis, D., Kosmicki, J.A., Grove, J., Stevens, C., Bybjerg-Grauholm, J., et al. (2019). Autism spectrum disorder and attention deficit hyperactivity disorder have a similar burden of rare protein-truncating variants. Nat. Neurosci. *22*, 1961–1965. https://doi.org/10.1038/s41593-019-0527-8.
34. Balduzzi, S., Rücker, G., and Schwarzer, G. (2019). How to perform a meta-analysis with R: a practical tutorial. Evid. Based Ment. Health *22*, 153–160. https://doi.org/10.1136/ebmental-2019-300117.
35. Dougherty, J.D., Marrus, N., Maloney, S.E., Yip, B., Sandin, S., Turner, T.N., Selmanovic, D., Kroll, K.L., Gutmann, D.H., Constantino, J.N., et al. (2022). Can the “female protective effect” liability threshold model explain sex differences in autism spectrum disorder? Neuron *110*, 3243–3262. https://doi.org/10.1016/j.neuron.2022.06.020.
36. Zeidan, J., Fombonne, E., Scorah, J., Ibrahim, A., Durkin, M.S., Saxena, S., Yusuf, A., Shih, A., and Elsabbagh, M. (2022). Global prevalence of autism: A systematic review update. Autism Res. *15*, 778–790. https://doi.org/10.1002/aur.2696.
37. Falconer, D.S. (1967). The inheritance of liability to diseases with variable age of onset, with particular reference to diabetes mellitus. Ann. Hum. Genet. *31*, 1–20. https://doi.org/10.1111/j.1469-1809.1967.tb02015.x.
38. Abrahams, B.S., Arking, D.E., Campbell, D.B., Mefford, H.C., Morrow, E.M., Weiss, L.A., Menashe, I., Wadkins, T., Banerjee-Basu, S., and Packer, A. (2013). SFARI Gene 2.0: a community-driven knowledgebase for the autism spectrum disorders (ASDs). Mol. Autism *4*, 36. https://doi.org/10.1186/2040-2392-4-36.
39. Ouwenga, R.L., and Dougherty, J. (2015). Fmrp targets or not: long, highly brain-expressed genes tend to be implicated in autism and brain disorders. Mol. Autism *6*, 16. https://doi.org/10.1186/s13229-015-0008-1.
40. Duong, T. (2022). ks: Kernel Smoothing. R package version 1.14.0.
