# Supplementary material for: Contribution of autosomal rare and de novo variants to sex differences in autism
Source: Am J Hum Genet. 2025 Feb 14;112(3):599–614. doi: 10.1016/j.ajhg.2025.01.016 (PMC11947420; doi:10.1016/j.ajhg.2025.01.016)
Supplement: Document S3. Article plus supplemental information [file mmc3.pdf]

Contribution of autosomal rare and *de novo* variants to sex differences in autism

Authors

Mahmoud Koko, F. Kyle Satterstrom, Autism Sequencing Consortium, APEX consortium, Varun Warrier, Hilary Martin

Correspondence

vw260@medschl.cam.ac.uk (V.W.),
hcm@sanger.ac.uk (H.M.)

This work evaluates the impact of rare coding variants on autism predisposition in 47,061 autistic individuals. It shows that the effect sizes of damaging autosomal variants on the liability scale do not differ significantly by sex, whether exome-wide or in genes sex-differentially expressed in the adult or fetal cortex.

Koko et al., 2025, The American Journal of Human Genetics *112*, 599–614
March 6, 2025

© 2025 The Authors. Published by Elsevier Inc. on behalf of American Society of Human Genetics.

https://doi.org/10.1016/j.ajhg.2025.01.016

CellPress

Contribution of autosomal rare and *de novo* variants to sex differences in autism

Mahmoud Koko,[1] F. Kyle Satterstrom,[2,3,4] Autism Sequencing Consortium,[6] APEX consortium,[6] Varun Warrier,[5,*] and Hilary Martin[1,*]

Summary

Autism is four times more prevalent in males than females. To study whether this reflects a difference in genetic predisposition attributed to autosomal rare variants, we evaluated sex differences in effect size of damaging protein-truncating and missense variants on autism predisposition in 47,061 autistic individuals using a liability model with differing thresholds. Given the sex differences in the rates of cognitive impairment among autistic individuals, we also compared effect sizes of rare variants between individuals with and without cognitive impairment or motor delay. Although these variants mediated different likelihoods of autism with versus without cognitive or motor difficulties, their effect sizes on the liability scale did not differ significantly by sex exome wide or in genes sex-differentially expressed in the cortex. *De novo* mutations were enriched in genes with male-biased expression in the adult cortex, but these genes did not show a significant sex difference on the liability scale, nor did the liability conferred by these genes differ significantly from other genes with similar loss-of-function intolerance and sex-averaged cortical expression. Exome-wide female bias in *de novo* protein-truncating mutation rates on the observed scale was driven by high-confidence and syndromic autism-predisposition genes. In summary, autosomal rare and damaging coding variants confer similar liability for autism in females and males.

Introduction

Large-scale rare variant association studies in autism have shown that rare protein-coding variants contribute significantly to autism liability. Genes associated with autism—particularly those with strong statistical or molecular evidence—are enriched for loss-of-function (LoF)-intolerant genes expressed in the brain.[1–3] It remains unclear whether rare variants contribute to the sex bias in autism; it is approximately four times more prevalent in males. This sex bias is less pronounced when autism is accompanied by cognitive impairment or motor developmental delay.[4,5]

The genetic underpinnings of autism include both rare and common variants.[6–8] The collective effect of the different genetic factors predisposing to autism on the trait prevalence is typically studied using the liability threshold model.[4] This model is often used to explain how additive genetic factors relate to a dichotomous diagnosis—by postulating that the combined effect of these predisposition factors in the population is normally distributed and that individuals diagnosed with autism will have exceeded a certain diagnostic threshold.[9] Under this model, a sex difference in population prevalence may arise either because there is a higher threshold in females relative to males so that females require a higher genetic predisposition to be diagnosed[10] or due to a single threshold with sex-biased effect sizes (gene-by-sex interaction) causing the same set of variants to push males, but not females, past the threshold.[11] Unlike comparisons on the observed scale (e.g., fold enrichment in variant rates in females versus males), examining effect sizes on the liability scale allows for a direct comparison between groups with different proportions of individuals with the trait.

Previous work on 12,270 autistic individuals showed that females have a higher burden of rare damaging variants.[12] However, it was not clarified if those observed differences translate into differences in liability. A separate analysis of overlapping cohorts (11,986 autistic individuals) by the Autism Sequencing Consortium (ASC) suggested that the observed significant sex differences in *de novo* mutation (DNM) enrichment did not translate into differences in the average effect size attributed to these damaging variants on the liability scale.[2] Importantly, autistic individuals show different rates of DNMs and over-transmission of damaging rare alleles depending on the presence of co-occurring cognitive impairment or dysmorphism.[13,14] More recent ASC work on 20,627 autistic individuals did not show evidence for gene-by-sex interaction among those harboring rare variants in autosomal genes significantly associated with autism and suggested that sex and phenotypic severity additively associate with rare variant burden in these autism-predisposition genes.[1] Given that a larger proportion of autistic females than males have co-occurring cognitive impairment, potentially because of an underdiagnosis of autistic females with otherwise typical cognitive development,[15,16]

[1]Human Genetics, Wellcome Sanger Institute, Hinxton, Cambridgeshire CB10 1SA, UK; [2]Program in Medical and Population Genetics, Broad Institute of MIT and Harvard, Cambridge, MA, USA; [3]Stanley Center for Psychiatric Research, Broad Institute of MIT and Harvard, Cambridge, MA, USA; [4]Analytic and Translational Genetics Unit, Department of Medicine, Massachusetts General Hospital, Boston, MA, USA; [5]Department of Psychiatry, Autism Research Centre, University of Cambridge, Cambridge, Cambridgeshire CB2 8AH, UK
[6]Further details can be found in the supplemental information
*Correspondence: vw260@medschl.cam.ac.uk (V.W.), hcm@sanger.ac.uk (H.M.)
https://doi.org/10.1016/j.ajhg.2025.01.016.
© 2025 The Authors. Published by Elsevier Inc. on behalf of American Society of Human Genetics.
This is an open access article under the CC BY license (http://creativecommons.org/licenses/by/4.0/).

it is unclear if the observed sex difference in rare variant rates is simply a reflection of differences in the proportion of individuals with cognitive impairment between sexes.[17,18]

Larger, more recently released cohorts like the Simons Foundation Powering Autism Research for Knowledge (SPARK), which includes more than 40,000 autistic individuals, offer a chance to examine rare variant liability and its relation to autism and co-occurring conditions in depth. Here, we meta-analyzed the ASC and SPARK datasets[1,14] of exome-sequenced samples to explore whether there is a sex difference in rare variant liability, both exome wide and in focused gene sets of high-confidence autism-associated genes and genes with sex-biased expression in the fetal and adult cortex (Figure S1). We performed sex-stratified analyses of rare missense and protein-truncating variants (PTVs) in exonic coding regions in 47,061 autistic individuals and 25,593 siblings or control individuals not diagnosed with autism from cohorts curated by SPARK and the ASC (Table S1). We then explored the relationship between the sex differences in liability and cognitive or motor difficulties co-occurring with autism.

Subjects and methods

A note on terminology

We use neutral terminology, including "autistic individuals," throughout the manuscript, in line with the preferences of a large number of autistic people. However, we use standard statistical terminology (e.g., liability, liability threshold model, risk ratio, and gene burden) to be consistent with other literature.

Ethics and approvals

We confirm that the datasets used for this study were obtained from research projects complying with relevant ethical regulations. The procedures followed were in accordance with the ethical standards of the responsible committee(s) on human experimentation (institutional and national), and proper informed consent was obtained. The ASC studies[1,2] were approved by Mass General Brigham Human Research Committee Institutional Review Board (IRB) protocol nos. 2012P001018 and 2013P000323. Access to SPARK phenotypic and genetic data[14] was approved by the Simons Foundation Autism Research Initiative (SFARI). SPARK participants were recruited under Western IRB protocol no. 20151664.

SPARK cohort

We used the second version of the integrated whole-exome sequencing data release[14] ("iWES2"; Figure S2) which spanned five sequencing waves (WES1–5) encompassing 106,744 individuals (44,304 of them were diagnosed with autism, and the rest were non-autistic parents, siblings, and a few extended family members). These included 25,386 trios (18,172 autistic individuals and 7,214 not diagnosed with autism), 23,346 samples with one sequenced parent (17,644 autistic individuals and 5,702 not diagnosed with autism), and 58,012 samples without parental sequences (8,488 autistic individuals and 49,524 not diagnosed with autism—with the latter group formed mostly of parents of other individuals in the trio-/pair-sequenced groups, i.e., few multi-generational families). (See Tables S3 and S4 for sample size in the different genetic ancestry groups.)

First, we performed exome quality control (QC) on all samples in iWES2, as detailed in supplemental methods section 1. Briefly, we annotated the variant calls with coding consequences on Matched Annotation from NCBI and Ensembl (MANE) transcripts (Ensembl release 108; genome build GRCh38) and filtered for variants having synonymous or more damaging consequences, prioritizing the most severe consequence when two genes were affected. We removed variants failing a random forest quality filter (Figure S3); genotypes with low depth ($DP < 10$), low genotype quality ($GQ < 10$), or low variant allele fraction ($VAF < 0.25$); and outlier samples on these metrics: total and singleton variant count, transition-transversion ratio, insertion-deletion ratio, and heterozygous-homozygous ratio.

We then excluded all samples that were potentially part of the ASC cohort (by removing all individuals in SPARK who indicated their previous participation in ASC studies), parents and siblings reported to have a developmental disorder/motor delay or cognitive impairment, and autistic parents. We then defined a set of maximally unrelated probands and maximally unrelated siblings by incrementally removing individuals with the highest number of related people (within each of these two subsets) while preferentially retaining females. Following QC, we evaluated the genotypes of 20,236 trio-sequenced individuals (13,473 with autism and 6,763 not diagnosed with autism) to identify rare DNMs and inherited variants (SPARK and gnomAD minor-allele frequency $< 0.1\%$; see supplemental methods section 3.2 for details).

We also did supplementary analyses in which we examined ultra-rare inherited variants (in one SPARK family and not in gnomAD) in an additional 18,816 child-parent pairs with one sequenced parent (13,435 with autism and 5,381 not diagnosed with autism) and ultra-rare variants (allele frequency $< 0.005\%$) of undetermined origin in 8,905 individuals without sequenced parents (6,533 with autism and 2,372 not diagnosed with autism). Further details on rare and ultra-rare variant filtering are available in supplemental methods section 4. (See Table S5 for the sample size after QC.)

ASC cohort

The QC of this dataset is described elsewhere in the context of a large rare variant association analysis.[1] This previous analysis primarily examined both sexes jointly using data from the Simons Simplex Collection and smaller ASC family-based cohorts (Figure S4), the SPARK Pilot and first exome sequencing wave (WES1), and Swedish and Danish case-control cohorts. From the ASC, we received sex-stratified gene-level rare variant counts[1,2] (gnomAD minor-allele frequency $< 0.1\%$) grouped by their mode of inheritance into DNMs (in probands or siblings) or inherited variants (transmitted or untransmitted in the probands).

DNM counts came from 10,488 individuals in the ASC family-based cohort only (8,028 autistic individuals and 2,460 not diagnosed with autism) and did not include those ascertained in SPARK Pilot and WES1 families and so were independent of the SPARK iWES2 dataset presented in the previous section. Some of the DNMs in the ASC cohort were collated from older studies and did not have accompanying information on inherited alleles. Therefore, inherited variants were evaluated in 9,929 of the 10,488 individuals for whom we had DNMs (7,570 autistic children and 2,359 siblings). We also obtained ultra-rare variant counts (allele frequency $= \sim 0.005\%$) from 14,188 individuals from the ASC

case-control cohorts (5,591 autistic individuals and 8,597 not diagnosed with autism). Table S2 shows the number of probands, siblings, and parents across the different ASC sites. See supplemental methods sections 3.1 (trios dataset) and 4.2 (case-control dataset) for more details.

De novo and inherited variants

We analyzed DNMs, rare transmitted variants, and rare untransmitted variants annotated as damaging PTVs, damaging missense variants, or synonymous variants. The analysis was limited to 17,296 protein-coding genes annotated in both the ASC and SPARK after QC. PTVs were considered damaging if they occurred in 1,742 highly LoF-intolerant genes in the most-constrained decile for the LoF observed over expected upper bound fraction (LOEUF) score, and missense variants were considered damaging if they had a missense badness, PolyPhen, and constraint (MPC) score $\geq$ 2 (in all genes). For additional sensitivity analyses, we relaxed the filtering threshold to include PTVs in the 2nd or 3rd LOEUF deciles (1,765 and 1,781 genes, respectively) and missense variants with MPC scores $\geq$1.

We carried out sex-stratified comparisons (autistic individuals against sex-matched siblings) as well as direct comparisons between sexes (autistic females versus autistic males) as described in the next section. The primary analysis of *de novo* and rare inherited variants (allele frequency $<$ 0.1%) was performed in the trio-sequenced individuals in both SPARK and ASC (21,501 autistic individuals and 9,223 siblings not diagnosed with autism). (See Table S6 for a list of trio-sequenced samples and Table S7 for a list of these DNMs.) The remaining data (individuals with sequence data from one or neither parent) were used for analyses of ultra-rare variants.

Exome-wide enrichment

The following statistical analyses are described in detail in supplemental methods section 5 and summarized briefly here. We used the ratio between the rate of DNMs in the probands (DNMs per sample) and the rate of DNMs in the siblings as a measure of enrichment.[1,2] For inherited variants, we calculated the ratio between parental alleles transmitted to the probands and the remaining untransmitted alleles. A ratio of 1 in the context of DNM analysis means that the probands and siblings have equal rates of rare DNMs; in the context of transmission analysis, it means that there is transmission equilibrium (half of the rare parental alleles are transmitted to the probands). For simplicity, we may refer to both ratios as the “rate ratio.”

To test for the significance of observed deviations from a DNM rate ratio 1, we used a two-sided binomial exact test to compare the DNM counts in the probands and the siblings. This tested whether the proportion of DNMs seen in the probands (from all DNMs in the probands and siblings) is significantly different from the proportion expected given their sample size (expected rate = $n_{\text{probands}}/(n_{\text{probands}} + n_{\text{siblings}})$). For inherited variants, we used a two-sided binomial exact test to compare the counts of transmitted and untransmitted alleles in the probands, examining whether the fraction of parental alleles transmitted to the probands was significantly different from 0.5. We obtained the confidence intervals (CIs) for the rate ratio from these binomial tests. Comparisons of variant rates between autistic individuals and control subjects in the case-control dataset were evaluated the same way as DNMs.

In direct tests of autistic females and males, we used the same method described above (a binomial test) to compare the fraction of total DNM counts (i.e., total in autistic males and females) that were observed in autistic females with the fraction expected given the fraction of all autistic individuals that were female. For transmission analysis, we calculated the ratio between the total number of parental alleles in autistic females and the total number in both autistic males and females and used this as the expected ratio for a binomial test comparing the transmitted variant counts in autistic females and the total transmitted variants (in both autistic males and females).

We performed these tests separately for each sex in SPARK and ASC and meta-analyzed the rate ratios for the sex-stratified comparisons using the inverse variance-weighted average of the rate ratios. In these exome-wide comparisons, the p values from the binomial test were conservatively corrected for 54 tests (using Bonferroni correction) from these groups: three sex-stratified comparisons (males, females, and sex difference), three cohorts (ASC, SPARK, and meta-analysis of both), three variant classes (synonymous, missense, and protein truncating), and two inheritance models (*de novo* and transmitted). We also used the Benjamini-Hochberg false discovery rate (FDR) adjustment, as the Bonferroni correction is conservative given the non-independence of the meta-analyzed estimates. We used asterisks in the figures to indicate whether the p values were $<$0.05 after Bonferroni correction (***), after FDR adjustment (**), or only before correction (*).

Variant liability

Assuming that autism liability is additive and normally distributed in the general population, the difference in the average liability in individuals who harbor particular variants and the average in the general population is a measure of the average effect size of these variants, i.e., an estimate of how far this group of variants pushes individuals harboring them (on average) on the liability scale. This variant liability can be estimated from variant rates in the study cohorts, as detailed previously.[2] The procedure we used to calculate the estimates is depicted in Figure S5 and detailed in supplemental methods section 6.1.

We used an autism population prevalence estimate of 2.5% in males (1 in 40), with a male-to-female prevalence ratio[19] of 4:1. We took the p values obtained from a binomial test comparing the variant counts in the probands and siblings (outlined above) and estimated the standard errors of the average liability estimates and, subsequently, the 95% CIs. These calculations were performed separately for each sex and each cohort and then meta-analyzed between cohorts. To directly compare autistic females and males, we calculated the difference between the variant liability estimates obtained separately in female and male probands (Z score difference). We corrected the p values for multiple testing in a similar manner to the exome-wide enrichment (54 tests).

Moreover, we explored whether removing 354 high-confidence and syndromic autosomal genes curated by the SFARI[20]—hereafter, SFARI genes—would uncover any sex-biased exome-wide variant liability. Under a model where females have a higher liability threshold than males (a "different-threshold" model), the sex ratio of damaging mutations in a gene or group of genes (i.e., higher rates in females) would be inversely correlated with the penetrance of those mutations,[4] with highly penetrant mutations being more prevalent in females. Because SFARI genes constitute a group of genes with higher autism penetrance relative to the remaining genes, they would be expected to show a higher sex bias on the observed scale (but similar effects on liability). Removing them

would thus enrich the analysis with genes mediating lower risk for autism, helping to highlight any underlying sex bias in effect sizes on the liability scale if it exists. PTVs in SFARI gene set were considered damaging if they occurred in 218 SFARI genes that are highly LoF intolerant (in the most-intolerant LOEUF decile), whereas missense variants were considered damaging if they had an MPC score ≥ 2 (in all 354 genes).

Autism with co-occurring cognitive difficulties

To explore how genetic architecture differed by phenotype, we split the autistic individuals in the ASC and SPARK cohorts into those with autism with co-occurring cognitive impairment and those with autism who otherwise had typical cognitive development or did not have reported information to allow classification (see supplemental methods section 2). We then compared each of these with siblings not diagnosed with autism as control subjects.

In the ASC cohort, we leveraged predefined categories of coexisting cognitive difficulties. Here, we separately tested rare variant enrichment in autistic individuals with co-occurring cognitive impairment (defined by the ASC as a full-scale intelligence quotient (IQ) score < 70, a Human Phenotype Ontology term indicating intellectual disability/cognitive impairment, or an International Classification of Diseases code indicating this diagnosis). The remaining autistic individuals with unknown cognitive impairment status and those with borderline and average IQs were grouped together. For the DNM analysis, there were 1,519 autistic males and 387 females with cognitive impairment ($\sim$23% of 6,615 autistic males and $\sim$27% of 1,413 autistic females; relative risk of cognitive impairment in females versus males = 1.19). For the over-transmission analyses, which did not include data from previously published work focused on DNMs, there were 1,357 autistic males and 335 females with cognitive impairment ($\sim$22% of 6,249 autistic males and $\sim$25% of 1,321 autistic females; relative risk = 1.17). (See Figure S4 and Table S11 for the percentage of autistic individuals with cognitive impairment across the contributing ASC sites.)

We classified SPARK individuals in a roughly similar manner to the ASC, i.e., one group for those with cognitive impairment (reported diagnosis of cognitive impairment or an IQ < 70) and another group for those without cognitive impairment or with an unknown cognitive impairment status. The proportions of those with cognitive impairment in SPARK trios were $\sim$23% among 10,482 males ($n = 2{,}424$) and $\sim$26% among 2,991 females ($n = 775$), with a relative risk of 1.12.

When estimating liability in the group with autism and cognitive impairment, we scaled the sex-specific population prevalence using the observed percentages of autistic individuals with cognitive impairment, i.e., using prevalence estimates of $\sim$0.58% in males (23% × 2.5%) and $\sim$0.16% in females (26% × 0.625%) for liability calculations; we subtracted these estimates from the total autism prevalence for each sex to estimate the liability in the second group of autistic individuals without cognitive impairment or with unknown cognitive impairment status. Male-specific Z scores were subtracted from female-specific estimates to assess the sex difference.

This phenotypic grouping did not consider motor delay, another important co-occurring condition.[2] To address these deficiencies, we performed a separate analysis in SPARK, in which we had more detailed phenotype data. Specifically, we defined two groups of autistic individuals with cognitive or motor impairment or without these conditions, excluding those with unknown status (see supplemental methods section 2.2).

Here, SPARK individuals reported to have an IQ < 80, cognitive impairment (reported professional diagnosis of an intellectual disability, cognitive impairment, global developmental delay, or borderline intellectual functioning), or motor delay (reported professional diagnosis of delay in walking or developmental coordination disorder) constituted the “autism with motor or cognitive impairment” group (4,209 trio sequenced, 4,714 with one sequenced parent, and 1,778 without sequenced parents). We chose the IQ cutoff of 80 since it was previously suggested that defining cognitive impairment in SPARK based on this cutoff minimizes the grouping of average and borderline IQ individuals together and that a diagnosis of an intellectual disability does not necessarily require an IQ < 70.[21] Individuals reported not to have any of these co-occurring conditions formed the “autism without motor or cognitive impairment” group (7,420 trio sequenced, 6,938 with one sequenced parent, and 2,141 without sequenced parents), whereas those with missing data on these phenotypes (1,844 trio sequenced, 1,756 with one sequenced parent, and 2,615 without sequenced parents) were considered unclassified.

Among 11,630 autistic individuals in SPARK who could be classified, $\sim$36% fell in the autism with motor or cognitive impairment group (among trios: 35% in males and 40% in females). The liability was estimated using prevalence estimates of $\sim$0.88% in males (35% × 2.5%) and 0.25% in females (40% × 0.625%) in the autism with motor or cognitive impairment group; for the autism without motor or cognitive impairment group, we used prevalence estimates of $\sim$1.63% in males (2.5%–0.88%) and $\sim$0.38% in females (0.625%–0.25%). Male-specific Z scores were subtracted from female-specific estimates to assess the sex difference.

Gene set burden

We evaluated the rare variant burden in SFARI genes and genes with sex-biased expression derived from meta-analyses of two bulk RNA sequencing (RNA-seq) datasets of human fetal cortical tissues[22] or two bulk RNA-seq datasets of adult human cortical tissues.[23] We examined these gene sets directly and also gauged the extent of the observed enrichment against the burden expected for a similarly sized gene set selected from the remaining protein-coding genes. For each tested gene set, we selected a random gene set matched for LoF constraint, brain expression, and coding sequence length distribution (described further in supplemental methods section 7.3) and counted DNMs, transmitted variants, and untransmitted variants. We repeated this procedure 10,000 times with replacement and took the average ratio (rate ratio between DNM counts in probands and siblings or transmitted-to-untransmitted ratio in the probands) and then used this ratio as the expected ratio in a binomial test as described above. Specifically, we tested the difference between the rate of DNMs between probands and siblings against the permutation-averaged expected ratio for this gene set (instead of the sample size ratio used in the exome-wide analyses), and we similarly tested rare variant over-transmission against the permutation-averaged expected transmitted-to-untransmitted ratio for the given gene set (instead of 0.5 as used in the exome-wide analysis). We also used the average variant rates across these 10,000 permutations instead of the rate in siblings to estimate the variant liability attributed to a gene set in excess of what is expected for matched genes.

Liability to coexisting developmental difficulties

Co-occurring cognitive and motor difficulties are more prevalent among autistic females, and a direct comparison between autistic

individuals with co-occurring motor/cognitive difficulties versus those without coexisting difficulties could help understand whether there is sex-biased liability to having such phenotypes. Here, we used a different-threshold model where these coexisting conditions were the trait, autistic individuals with these conditions were the probands, and autistic individuals without these conditions were the control subjects. We performed these comparisons in SPARK only (i.e., removing those with unknown information) rather than comparing those with cognitive impairment in SPARK and ASC to those without cognitive impairment or with unknown cognitive impairment status, as this grouping would probably underestimate the differences between the two groups (making the interpretation of results difficult). We estimated the sex-stratified liability of having motor or cognitive impairment among autistic individuals assuming prevalences of 0.4 in females and 0.35 in males (the observed prevalences in SPARK trios). To validate the conclusions from this analysis that may be confounded by the use of autistic probands as control subjects (e.g., collider bias), we leveraged published DNMs from 31,565 children ascertained for various neurodevelopmental disorders (NDDs)[14] and compared the observed counts of damaging protein-truncating and missense DNMs to the expected counts from a mutational model (see supplemental methods section 3.1) in order to estimate the liability attributed to these variants assuming NDDs have population prevalences[24,25] of 2% in males and 1.5% in females (the observed sex ratio in this NDD cohort was ~1.3).

Results

We examined the burden of autosomal *de novo* and rare inherited variants (minor-allele frequency < 0.1%) exome wide and in specific gene sets in a cohort of 21,501 autistic individuals (13,473 from SPARK and 8,028 from ASC) and 9,223 siblings (6,763 from SPARK and 2,460 from ASC) (see Figure S1 for an outline of all analyses). In these trio-sequenced individuals, the sex-stratified synonymous variant rates (variants per sample) were comparable between the autistic probands and siblings not diagnosed with autism (i.e., rate ratio not significantly different from 1), whereas the rates of *de novo* high-confidence PTVs in highly LoF-intolerant genes (hereafter, damaging PTVs) and missense variants with an MPC score ≥ 2 (damaging missense) were higher in autistic probands. Autistic females showed higher rates of damaging variants than autistic males, particularly those occurring *de novo*, albeit to different degrees in SPARK and ASC (see Table S12 and supplemental results section 1 for details). Over-transmission was most noticeable in damaging PTVs (Figure S6). Additional analyses of ultra-rare variants in 13,435 autistic individuals with sequence data from one parent and 12,125 autistic individuals without parental sequence data also showed an enrichment in damaging PTVs (Figure S7; Table S13).

Our comparisons of DNMs and inherited variants between sexes (Figure 1; Table S8), which we describe in more detail in supplemental results section 2, recapitulated the known sex-differential patterns of enrichment of damaging PTVs (in the 1st LOEUF decile) and missense variants (MPC ≥ 2) in these cohorts.[1,2,12,14] Previous work in a subset of the current ASC cohort[2] showed that the sex bias in DNM rates was strongest when examining PTVs in highly LoF-intolerant genes (defined in that work as a probability of LoF intolerance [pLI] ≥ 0.995) but not significant in less intolerant genes. Similarly, we do not find a significant sex bias on the observed scale when testing other genes in the 2nd and 3rd LOEUF deciles or missense DNMs with lower MPC scores (MPC ≥ 1) (Figure S8; Table S14). However, these comparisons (rate ratios) do not take into account the differences in trait prevalence. Therefore, we next examined whether the sex differences on the observed scale translate into sex differences in liability, which allows comparing effect sizes between groups with different trait prevalences.

Sex differences in genetic liability conferred by rare variants exome wide

Here, we focus on the meta-analyzed (ASC and SPARK) cohort (Figure 1A) with a total sample size of 4,404 autistic females (versus 4,707 female siblings) and 17,097 autistic males (versus 4,516 male siblings). Despite the relatively higher enrichment (rate ratio) of damaging protein-truncating and missense DNMs in autistic females compared to males (Figure 1B), we did not find statistically significant differences between the male-derived and female-derived liability estimates, neither when testing damaging PTVs in the 1st LOEUF decile and missense variants with MPC scores ≥ 2 (Figure 1) nor when using relaxed filters to include PTVs in the 2nd and 3rd LOEUF deciles and missense variants with MPC scores ≥ 1 (Figure S8).

Specifically, the effect sizes of damaging protein-truncating DNMs (1st LOEUF decile) on the liability scale were not significantly different between autistic females and autistic males ($Z_{\text{sex-difference}} = 0.90$; 95% CI = −0.0684 to 0.25; $p = 0.27$), nor were the effect sizes of damaging missense DNMs (MPC ≥ 2) ($Z_{\text{sex-difference}} = 0.093$; 95% CI = −0.014 to 0.20; $p = 0.087$). Similarly, the liability was not significantly different between sexes when comparing DNM rates between autistic probands and all siblings (instead of sex-matched siblings) or sex-discordant siblings (Figure S9; Table S15).

Rare inherited damaging PTVs conveyed significant liabilities in females and males, but these effect sizes were not significantly different between the two sexes ($Z_{\text{sex-difference}} = 0.013$; 95% CI = −0.037 to 0.062; $p = 0.62$). Inherited damaging missense variants had higher liability in males compared to females. However, the difference was small and only significant before correcting for 54 multiple tests ($Z_{\text{sex-difference}} = -0.013$, 95% CI = −0.00052 to −0.026; $p = 0.041$; FDR-adjusted $p = 0.096$; Bonferroni-corrected $p = 1$). As detailed in supplemental results section 2.2.3, there was a small imbalance in transmitted and untransmitted synonymous alleles in males ($Z_{\text{males}} = 0.0016$; 95% CI = 0.0007 to 0.0026), but it is unlikely to affect the main conclusions (i.e., no significant sex differences).

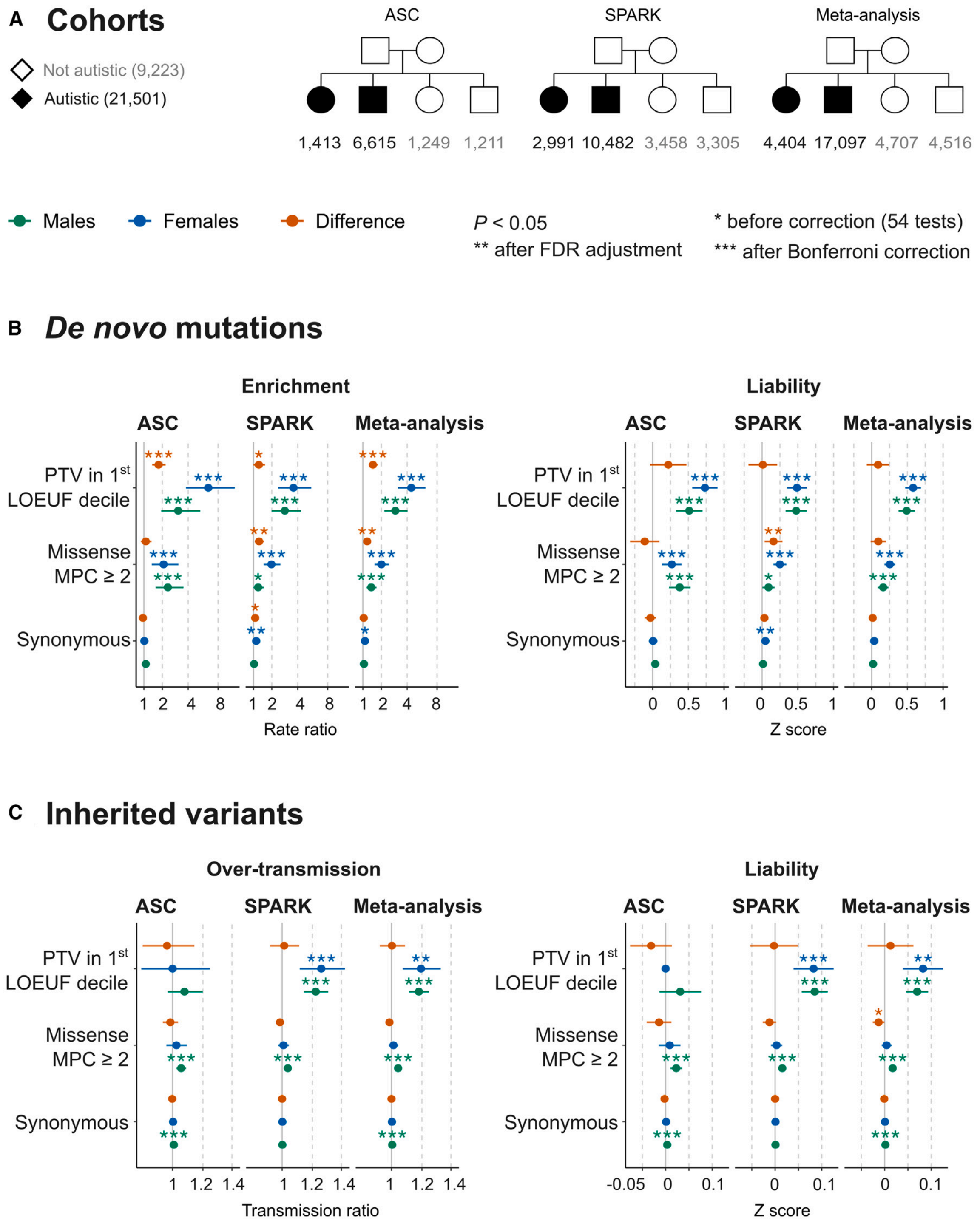

Figure 1. Exome-wide rare variant burden and liability in SPARK and ASC trio-sequenced cohorts

(A) The sample size of the trio-sequenced individuals in the Simons Foundation Powering Autism Research for Knowledge (SPARK) study and the Autism Sequencing Consortium (ASC) cohorts.

(B) Sex-stratified DNM enrichment and liability. To obtain sex-specific effect sizes on the observed scale, the average DNM rate per trio in autistic males (green dots) or females (blue dots) was divided by the average rate in sex-matched siblings not diagnosed with autism (rate ratio; left). Corresponding effect sizes on the liability scale (Z score; right) were measured as explained in Figure S5A and supplemental methods section 6. For sex differences in enrichment (red dots), the observed DNM rate in autistic females was divided by the rate in autistic males (a rate ratio > 1 thus indicates that females show a higher enrichment). The sex difference in variant liability was estimated by subtracting the Z scores of the male-only analysis from the female-only Z scores (a Z score > 0 indicates that females show a higher effect size on the liability scale).

(C) Over-transmission and liability of inherited variants. These were assessed using similar comparisons between parental alleles transmitted to autistic individuals and untransmitted alleles (see subjects and methods). Error bars show 95% confidence intervals. See supplemental results sections 2.1.3 and 2.2.3 for further details on synonymous variant imbalances.

The sex-stratified cohort-level effect sizes in Figure 1 were generally consistent with the meta-analyzed estimates except for inherited damaging PTVs; these conveyed significant liability only in SPARK (Figure 1C). Although the ASC cohort did not show a significant enrichment in inherited damaging PTVs exome wide, it was significantly enriched for inherited damaging PTVs in known autism-predisposition genes (i.e., high-confidence PTVs in 218 highly LoF-intolerant genes among 354 SFARI genes; Figure S11; supplemental results section 2.2). Conversely, removing SFARI genes did not change the overall conclusions regarding the (lack of) sex differences in the average liability of DNM (Figure S10) and rare inherited variants (Figure S11). (See Table S16 for details.)

Further details on cohort-level sex differences in variant rates and liability among trios are presented in supplemental results sections 2.1 and 2.2. We note that synonymous DNMs in autistic females in SPARK showed an association with autism ($Z = 0.046$; 95% CI = 0.01 to 0.082; $p = 0.011$) that did not persist in the meta-analysis ($Z = 0.036$; 95% CI = 0.0055 to 0.067; $p = 0.052$) (see "liability" in Figure 1B). Restricting to ultra-rare alleles in SPARK (Figure S12; Table S17) controlled this spurious signal in synonymous DNMs (see supplemental results section 2.1.4).

An exome-wide analysis in other subsets of the SPARK and ASC cohorts reiterated the findings from the trio analysis. In brief, the average effect size of ultra-rare inherited variants in autistic individuals with sequence data from one parent in SPARK did not differ significantly between sexes after correction for multiple testing (Figure S13; Table S18; supplemental results section 2.3.1). Similarly, there was no significant sex difference in the average liability conferred by ultra-rare variants in the ASC case-control cohorts or in the autistic individuals without parental sequence data in SPARK (Figure S14; Table S19; supplemental results section 2.3.2).

To test whether there are differences between mothers and fathers in the burden, transmission, and liability of ultra-rare parental alleles (seen in one parent and not in gnomAD), we leveraged data from trio-sequenced child-parent pairs in ASC ($n = 7{,}570 \times 2$) and SPARK ($n = 13{,}473 \times 2$), along with other duo-sequenced pairs in SPARK ($n = 13{,}435$), and evaluated parent-of-origin effects in 55,521 autistic-child nonautistic-parent pairs. Although the mothers of autistic individuals had a significantly higher burden of damaging ultra-rare protein-truncating (rate ratio = 1.15, 95% CI = 1.08 to 1.22, $p = 2.67 \times 10^{-6}$) and damaging missense ultra-rare (rate ratio = 1.06, 95% CI = 1.03 to 1.09, $p = 3.0 \times 10^{-5}$) variants compared to the fathers (Figure S15; Table S20), the transmission ratios and the effect sizes of inherited alleles on the liability scale did not differ significantly by the sex of the parent ($p > 0.05$) (Figure S16; Table S21). (See supplemental results section 2.3.3 for details.)

To recapitulate, the average liability conferred by damaging *de novo* protein-truncating and missense mutations as well as inherited PTVs did not show a significant sex difference in a meta-analysis of trio-sequenced individuals from ASC and SPARK. Inherited damaging missense variants conferred higher liability in males, but this difference was very small in magnitude and only significant before accounting for multiple testing. It was also not seen when examining the transmission of ultra-rare variants in a separate set of autistic individuals from SPARK with sequence data from one parent, when examining all trio-/duo-sequenced child-parent pairs together, or in the remaining case-control analyses (ASC case-control cohorts and autistic individuals without parental sequence data in SPARK). Next, we examined whether rare variant liability differs when accounting for co-occurring cognitive difficulties.

Exome-wide burden in autistic individuals with or without cognitive difficulties

Both SPARK and ASC cohorts included a mixture of autistic individuals with varying degrees of cognitive difficulties.[1,14] There is a higher likelihood of co-occurring cognitive impairment among autistic females compared to autistic males (relative risks of 1.17 in the ASC cohort and 1.12 in SPARK); autistic individuals with cognitive impairment, in turn, have a higher likelihood of harboring high-impact DNMs than those without cognitive impairment.[14] We hypothesized that the sex differences in damaging DNM rate ratios seen when examining a mix of individuals with and without cognitive difficulties may reflect a difference in the relative frequency of cognitive impairment between the sexes. We thus performed exome-wide comparisons in ASC and SPARK trios stratified by co-occurring cognitive impairment. Specifically, we compared a group of autistic individuals with coexisting cognitive impairment and another group of individuals without cognitive impairment or with an unknown status (since these could not be distinguished using the available ASC data) to the same set of siblings and then meta-analyzed the outcomes between ASC and SPARK (Figure 2A).

In this meta-analysis (Figure 2; Table S9), damaging protein-truncating DNMs showed sex differences on the observed scale among those with cognitive impairment (rate $\text{ratio}_{\text{sex-difference}} = 1.68$, 95% CI = 1.28 to 2.20, $p = 1.8 \times 10^{-4}$, Bonferroni-corrected $p = 0.02$) and, to a lesser extent, those without cognitive difficulties (rate $\text{ratio}_{\text{sex-difference}} = 1.4$, 95% CI = 1.14 to 1.71, $p = 0.0012$, FDR-adjusted $p = 0.0037$, Bonferroni-corrected $p = 0.12$). There was no significant sex difference in damaging missense DNM rates between those with cognitive impairment (rate $\text{ratio}_{\text{sex-difference}} = 1.06$; 95% CI = 0.80. to 1.39; $p = 0.69$). However, damaging missense DNMs showed significantly higher rates in females than males without cognitive impairment (rate $\text{ratio}_{\text{sex-difference}} = 1.27$, 95% CI = 1.06 to 1.51, $p = 0.0080$, FDR-adjusted $p = 0.021$, Bonferroni-corrected $p = 0.86$). On the liability scale, the meta-analyzed effect sizes of damaging protein-truncating and

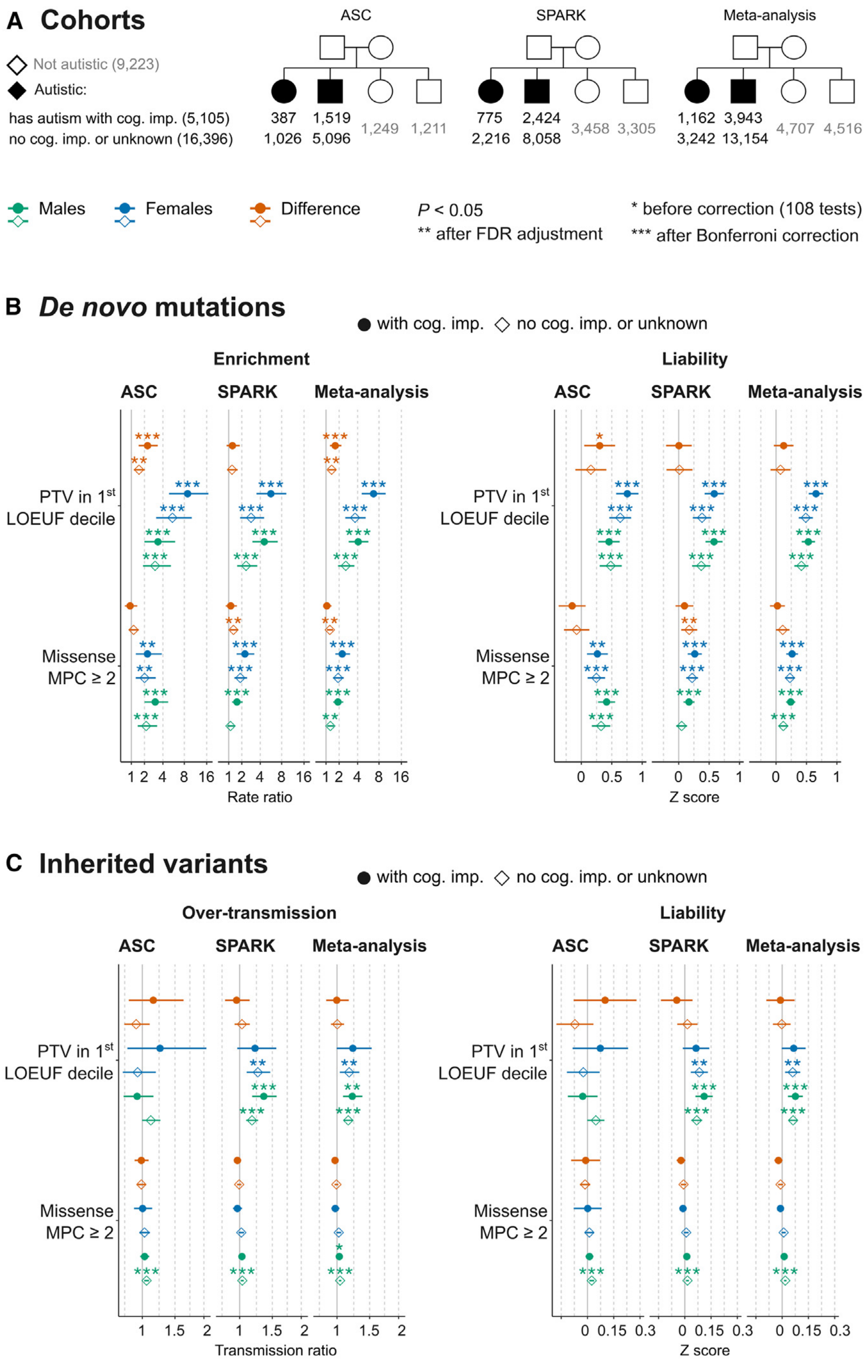

Figure 2. Sex differences in the association between exome-wide burden of damaging *de novo* and rare variants and co-occurring intellectual disability in ASC and SPARK

Trio-sequenced individuals from the Autism Sequencing Consortium (ASC) and Simons Foundation Powering Autism Research for Knowledge (SPARK) study cohorts were divided into those who have co-occurring cognitive impairment (cog. imp.; solid dots) and those who do not have cognitive impairment or have missing information (no cog. imp. or unknown; blank diamonds). The sample size of these subgroups is given in (A). In each group, we examined the risk ratio and average liability attributed to damaging DNMs (versus the same group of siblings) (B) and rare inherited variants (transmitted versus untransmitted) (C). Variant burden and liability estimates were meta-analyzed between ASC and SPARK. Sex differences in DNM rate ratios were estimated by direct comparisons of autistic females and males (ratio > 1 means that females have a higher DNM rate), and sex differences in the effect sizes on the liability scale were estimated by subtracting the *Z* scores (score > 0 means that females have a higher effect size).

missense DNMs did not differ significantly between the two sexes after stratifying by cognitive impairment ($p > 0.05$) (Figure 2B). Rare inherited variants did not show significant sex differences in over-transmission and liability (Figure 2C). The cohort-level analyses revealed more nuanced patterns, which we discuss in detail in supplemental results section 3.1.

To follow up on the finding that there was no significant exome-wide sex difference in DNM burden when removing SFARI genes in our analysis of all autistic individuals (Figure S10B), we performed a similar analysis (i.e., removing SFARI genes) in those with and without cognitive impairment. For this, we meta-analyzed ASC and SPARK and included additional relaxed variant filters to examine variants with smaller effect sizes. We did not find any significant sex differences in the burden of damaging *de novo* and rare inherited variants, neither on the observed nor the liability scale, reiterating that the observed exome-wide differences in DNM rates are driven primarily by SFARI genes (Figure S17; Table S22).

On the other hand, SFARI genes—when examined separately—had significantly higher damaging DNM rates in females versus males with cognitive impairment (also in those without cognitive difficulties but to a lesser extent), and the sex bias in DNM rates was significantly higher than what is expected from matched genes (Table S23). Still, on the liability scale, the meta-analyzed effect sizes of these DNMs did not differ significantly between the two sexes (Figure S18), nor did the liability conveyed by rare inherited variants in this gene set (Figure S19). Further details are given in supplemental results section 3.2. Moreover, we did not find any significant sex differences in the liability conveyed by *de novo* and rare inherited variants in sex-biased differentially expressed genes in the human fetal cortex (117

female-biased and 305 male-biased genes) or adult cortex (2,427 female-biased and 2,852 male-biased genes) after correction for multiple testing (Figures S20–S27; Tables S24–S27). The average effect sizes of these sex-differentially expressed genes (on both scales) were not significantly higher than what are measured in similarly sized gene sets matched for coding length, LoF constraint, and sex-averaged expression levels (see supplemental results section 3.3).

We repeated the exome-wide comparisons in SPARK subcohorts stratified by co-occurring cognitive difficulties and motor delay (both versus the same set of siblings not diagnosed with autism in SPARK) while removing those with unknown motor/cognitive impairment status (Figure 3A). These comparisons are detailed in supplemental results section 4.1. In brief, among those with motor/cognitive difficulties (Figure 3; Table S10), we found similar results (i.e., comparable effect sizes on the liability scale) to those seen in probands with cognitive impairment (Figure 2). The results in SFARI genes (Figure S29; Table S29) were also congruent with what we saw when stratifying by cognitive impairment (Figure S18; Table S22) (for details, see supplemental results section 4.2).

Compared to sex-matched siblings, synonymous DNMs were more prevalent in autistic females without motor or cognitive impairment ($Z = 0.069$; 95% CI = 0.028 to 0.11; $p = 9.9 \times 10^{-4}$) but not in autistic females with these conditions ($Z = -0.018$; 95% CI = −0.068 to 0.033; $p = 0.50$) (see "liability" in Figure 3B). This spurious association was not seen when evaluating ultra-rare DNMs in samples well-matched on genetic ancestry ($Z = 0.029$; 95% CI = −0.041 to 0.099; $p = 0.42$) (see Figure S28, Table S28, and supplemental results section 4.1.3).

In SPARK, there was no significant sex difference in damaging DNM rates (observed scale) after stratification by coexisting cognitive impairment despite the more stringent grouping (removing those with unknown status and considering motor difficulties) (SPARK in Figures 2B and 3B). As this is different from what is seen in the ASC cohort (ASC in Figure 2B), we ran a permutation analysis in SPARK (see supplemental results section 4.2.3 for details), which suggested that the difference in rates of coexisting difficulties between males and females does not drive the sex differences in DNM rates on the observed scale (Figure S30; Table S30). The liability conveyed by damaging rare inherited variants when considering both cognitive and motor difficulties (Figure 3C) was comparable to when we stratified the cohort by cognitive impairment only (Figure 2C). Similarly, there were no significant sex differences in the effect sizes of these variants after accounting for multiple testing. We did not observe significant sex differences in the rate ratios of ultra-rare damaging variants in the remaining autistic individuals in SPARK with one sequenced parent (Figure S31; Table S31) or without sequenced parents (Figure S32; Table S32) after stratifying by cognitive or motor impairment (see supplemental results section 4.3).

In summary, we have seen in a meta-analysis of SPARK and ASC family-based cohorts that the exome-wide effect sizes of damaging *de novo* and rare inherited variants on the liability scale did not differ significantly between autistic males and females—either before or after stratifying by coexisting cognitive difficulties. Sex differences in the genetic burden on the observed scale were most prominent for damaging protein-truncating DNMs and among those with coexisting cognitive impairment. These differences were driven by high-confidence and syndromic-autism-predisposition genes, which had significantly higher effect sizes than similarly sized genes matched for LoF constraint and brain expression.

Rare variants are insufficient to reach the liability threshold for autism

A key question is whether the genetic predisposition conveyed by damaging *de novo* and rare variants is sufficient by itself to cause autism, which would require crossing a liability threshold of ~2 units in males and higher in females. For this, we consider here the effect sizes of protein-truncating DNMs in the 1st LOEUF decile (the most impactful class) (Figure 2B). In those with autism and cognitive impairment, the exome-wide average effect sizes were 0.66 in females (95% CI = 0.54 to 0.78) and 0.53 in males (95% CI = 0.42 to 0.64), and the effect sizes in SFARI genes were 1.34 units in females (95% CI = 1.1 to 1.57) and 1.26 in males (95% CI = 1.03 to 1.50). Smaller effect sizes were observed in those without cognitive impairment or with an unknown status (~0.5 exome wide and ~1 in autism-predisposition genes). We obtained similar estimates when we examined autistic individuals with or without motor/cognitive difficulties in SPARK (Figures 3B and S29). Thus, in none of these groups of individuals are these rare variants sufficient to cause autism by themselves.

To gain a better understanding of the sex differences in genetic predisposition to cognitive and motor difficulties co-occurring with autism, we compared probands with motor/cognitive impairment to those without these coexisting difficulties in SPARK. In contrast to the former comparisons against non-autistic siblings, which tested the liability conferred to autism *per se,* this analysis measures the extent of the liability to coexisting difficulties in those already diagnosed with autism and whether it differs by sex. Here, we used a different-threshold model where these coexisting difficulties have prevalences of 0.35 among autistic males and 0.40 among females, i.e., females have a lower threshold because there are more females than males with motor/cognitive impairment.

Under this model, the observed 2-fold enrichment of protein-truncating DNMs (in the 1st LOEUF decile) in autistic individuals with versus without motor or cognitive impairment translates to 0.39 units on the liability scale in females (95% CI = 0.11 to 0.66) and 0.45 units in males (95% CI = 0.30 to 0.61) (Figure 3B)—a sufficient amount to reach the threshold to have motor or cognitive

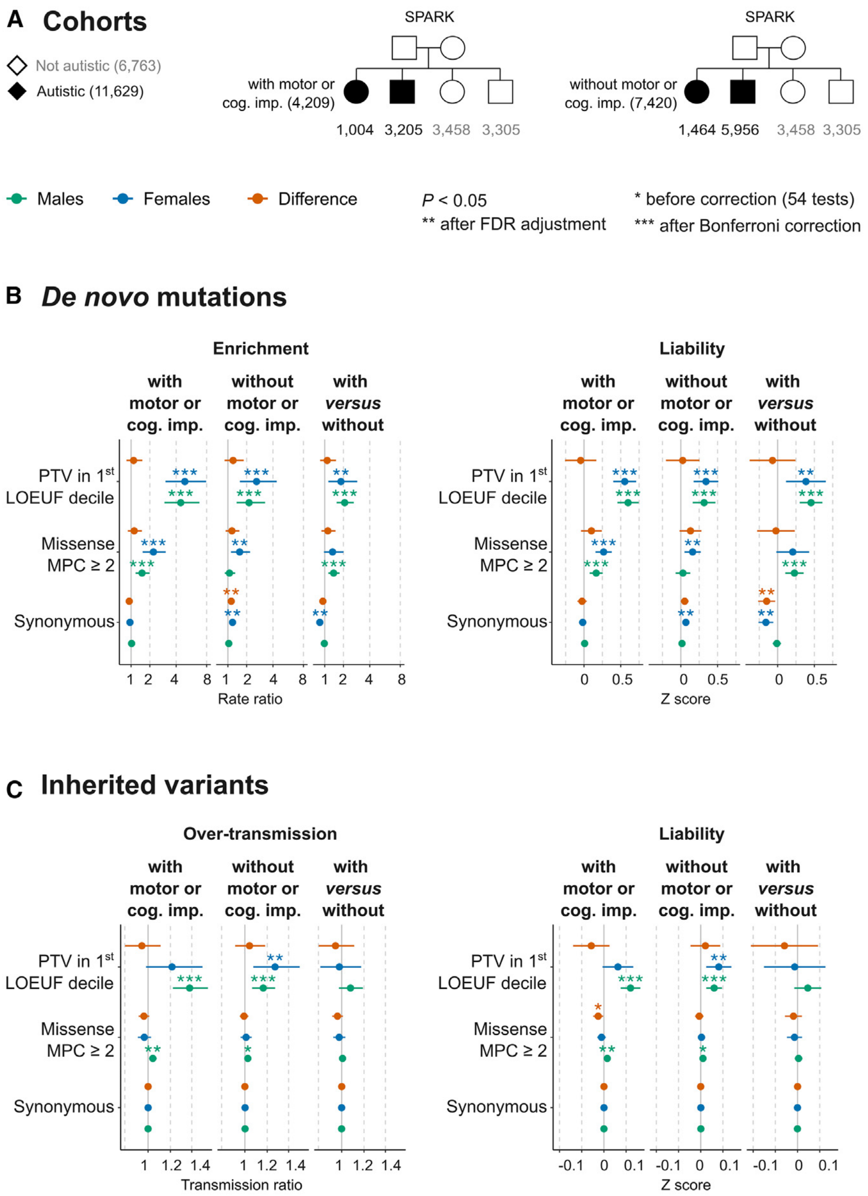

Figure 3. Rare variant burden in autistic individuals with and without cognitive impairment or motor delay in SPARK trio-sequenced cohort

(A) The sample size in two sub-cohorts of trio-sequenced individuals from the Simons Foundation Powering Autism Research for Knowledge (SPARK) study divided based on the presence of co-occurring motor developmental delays or cognitive impairment ("motor or cog. imp.").

(B) Sex-stratified observed DNM rates (left) and average liability (right) (see subjects and methods). In addition to the comparisons versus siblings, autistic probands with motor or cognitive difficulties were compared directly to sex-matched autistic individuals without these co-occurring conditions ("with versus without").

(C) Over-transmission of rare inherited variants (left) and their average liability (right) (see subjects and methods). See supplemental results section 4.1.3 for details on the imbalance of synonymous variants. Limiting the analysis in (B) to ultra-rare DNMs in ancestry-matched autistic females and siblings showed well-balanced synonymous DNM burden ($p = 0.42$; Figure S28).

impairment in autistic individuals (~0.25 units in females and ~0.39 in males). In SFARI genes, the effect sizes were 0.67 in females (95% CI = 0.30 to 1.03) and 0.57 in males (95% CI = 0.35 to 0.80) (Figure S29). The sex difference was not significant, either exome wide ($p = 0.66$) or in SFARI genes ($p = 0.67$). Seeking further validation, we estimated the liability conferred by damaging DNMs in SFARI genes in 31,565 trios diagnosed with NDDs not specifically ascertained for autism[14] (see supplemental results section 4.4). The effect sizes were indeed sufficient to reach the liability threshold for NDDs and not significantly different between the sexes in this independent cohort (Figure S33; Table S33).

To sum up, damaging protein-truncating DNMs in SFARI autism-predisposition genes confer similar liabilities in both sexes that are enough to reach the threshold for having neurodevelopmental conditions in general or coexisting motor and cognitive difficulties in autistic individuals in particular. However, they do not confer enough liability to reach the threshold for autism *per se* in either sex, regardless of whether these individuals have coexisting motor or cognitive difficulties or not.

Discussion

We examined the sex differences in rare autosomal coding variant rates both exome wide and in specific gene sets in 47,061 autistic individuals from two large autism cohorts,[1,14] showing that the average liability attributed to damaging rare variants exome wide and in genes with sex-biased expression in the cortex is not statistically significantly different between males and females.

The sex differences in DNM rates on the observed scale did not translate into differences in variant liability between sexes (Figure 1B) and were driven by known autism-predisposition genes (Figure S10), which also increase the chance of other developmental disorders affecting motor and cognitive skills (Figure S33). We did not find evidence that the sex differences in damaging DNM rate ratios seen when examining a mix of individuals with and without motor and cognitive difficulties reflect a difference in the relative frequency of these endo-phenotypes between the sexes. Autistic individuals in the ASC cohort still showed enrichment of DNMs in females versus males even when restricting to those with cognitive

impairment (Figure 2B). Although we did not see a significant sex difference in the rates of damaging protein-truncating DNMs in SPARK when autistic individuals were stratified by coexisting cognitive impairment (Figure 2B) or motor/cognitive difficulties (Figure 3B), a permutation analysis suggested that co-occuring difficulties—alone—cannot account for the sex differences on the observed scale (Figure S30). Thus, it seems likely that the difference in damaging DNM rates between the sexes is, instead, a reflection of the different thresholds under the liability threshold model, which we discuss further below.

SFARI high-confidence and syndromic-autism-predisposition genes drove the exome-wide female bias in DNM burden on the observed scale (Figure S10), showing substantially higher DNM rates compared to matched genes with comparable constraint and brain expression (Figure S18). This is consistent with our recent observation (in a smaller subset of SPARK and Simons Simplex Collection) that the female excess in damaging DNMs is explained by a small set of developmental genes and is not accounted for fully by co-occuring difficulties.[26] The effect sizes of damaging variants in these genes on the liability scale were also higher than those estimated for matched genes but did not differ significantly by sex (Figure S18). On the other hand, the liability attributed to damaging *de novo* and rare variants in a small set of sex-differentially expressed genes in the fetal cortex[22] or a larger set of genes with sex-biased expressed in the adult cortex[23] did not differ significantly from what is expected from matched genes (Figures S20–S27). Among these differentially expressed genes, the strongest enrichment and female bias on the observed scale (rate ratios) was seen in protein-truncating DNMs in genes showing male-biased expression in the adult cortex, but this was attributable to their overlap with LoF-intolerant genes and genes with high expression in brain (Figure S20). This is in line with previous findings that long highly brain-expressed genes overlap significantly with autism-predisposition genes and explain their over-representation among *Fragile X messenger ribonucleoprotein 1*-binding targets[27] (*FMR1* [MIM: 309550]). The enrichment of sex-differentially expressed genes in damaging DNMs is likely a reflection of their enrichment in neuronal genes, which has been previously noted.[23] The findings of these analyses of differentially expressed genes should be interpreted cautiously, as there are several caveats, which we discuss briefly in supplemental results section 3.3.4.

Protein-disrupting alterations confer the highest predisposition for autism among rare short coding variants.[1,12,14] Since genes implicated through *de novo* association are generally developmental disorder genes (e.g., SFARI genes), it is unclear whether they increase the predisposition for autism *per se*. Under a different-threshold liability threshold model, in which autism predisposition is assumed to be additive and normally distributed (Figure S5), an autism prevalence of 2.5% among males in the general population puts the threshold for autism diagnosis at 1.96 standardized units (2.5 units in females assuming ~4:1 ratio). (See Figure S5 and supplemental methods section 6.1.) We estimate that the liability conveyed by protein-truncating DNMs (in highly LoF-constrained genes) alone is insufficient to reach the threshold for autism diagnosis in the absence of other factors such as, for example, a high polygenic score for autism. This was true for the liabilities estimated in those with autism and motor/cognitive difficulties or without these coexisting conditions in SPARK (Figure 3), when meta-analyzing SPARK and ASC stratified by cognitive impairment (Figure 2), and when considering protein-truncating DNMs in SFARI genes in all these groups (Figures S18 and S29).

On the other hand, the analysis we carried out in SPARK to estimate the liability to co-occurring motor and cognitive difficulties suggests that damaging protein-truncating DNMs have an average effect size that conveys enough risk to cause these co-occurring difficulties when they are seen in approximately one-third of the SPARK cohort of autistic children—a proportion similar to that of the whole population of autistic individuals.[4] Protein-truncating DNMs in SFARI genes conferred enough predisposition to cognitive and motor difficulties among autistic individuals in SPARK so that an autistic individual harboring such a variant will have exceeded the liability threshold for these co-occurring difficulties (Figure S29), reflecting how cognitive or motor difficulties are key phenotypic presentations associated with harboring damaging variants in these genes. This was corroborated by observations in an independent cohort of trios ascertained for various NDDs rather than autism specifically—where these genes conferred genetic predisposition on par with genes curated for their association with developmental disorders (DDG2P genes[28]) (Figure S33). Thus, our results suggest that damaging DNMs are insufficient by themselves to cause autism, but they can cause cognitive or motor impairment (NDDs). This fits with the observation that most autism-predisposition genes are known to cause motor disorders or intellectual disability with high penetrance (most are DDG2P genes), but the penetrance of autism in most of these genes is often low to moderate.[4,29] Hence, autism is likely an incompletely penetrant, complex phenotype within the phenotypic spectrum of these developmental genes.

The difference in the proportion of autistic females versus autistic males with cognitive impairment could be explained by the presence of genetic risk factors with similar effect sizes in the two sexes that are highly penetrant for cognitive impairment but are more frequent in autistic females than autistic males due to the different thresholds for autism. In particular, as noted above, protein-truncating DNMs in highly LoF-intolerant genes or SFARI genes have—on average—moderate penetrance for autism but high penetrance for cognitive impairment. The liability threshold for diagnosing cognitive impairment is relatively similar between the sexes, as suggested by the low sex bias in neurodevelopmental conditions

featuring profound cognitive difficulties.[24,25,30,31]; For instance, there are 1.6 males for every female in the Deciphering Developmental Disorders study.[32] Females reaching the (high) threshold on the liability scale for autism diagnosis are more likely than both non-autistic females and autistic males to harbor damaging protein-truncating DNMs (evidenced by the higher risk ratio for carrying these DNMs on the observed scale; Figure 1B), and in turn, more autistic females than autistic males are likely to have reached the threshold for cognitive impairment as well (given the high penetrance of these DNMs for cognitive impairment in both sexes). It is plausible that the sex difference in the rates of autism with coexisting cognitive impairment may partly reflect biased diagnostic sensitivities, e.g., because females typically show autistic traits that are less likely (than autistic traits seen typically in males) to prompt clinical evaluation if they have otherwise typical cognitive development.[33] However, epidemiological data suggest that the higher proportion of autistic females with cognitive impairment (relative to autistic males) is more likely a true property of the autistic spectrum,[34] and this was observed to various degrees in several autism cohorts with different ascertainment strategies, e.g., in SPARK and among most of the ASC sites (Figure S4).

Our analysis relied on a standard liability threshold model assuming equal variance of the liability distribution in males and females.[4,10] This model is easily interpretable and allows direct comparisons between the two sexes on the same scale. Under this different-threshold model, females require a higher load of additive risk elements to reach their higher threshold; the equal effect sizes on the liability scale, and the sex-differential rates of highly penetrant variants, are consistent with the assumptions of the different-threshold model. However, the liability conveyed by rare variants alone is not sufficient to reach the threshold for autism diagnosis in the absence of other factors, even when lower thresholds in females (sex ratios of 3:1 and 2:1) are considered (Figure S34; Table S34). The genetic predisposition from rare variants could be complemented by other factors, e.g., common variants. It has been postulated that common variants may drive most of the genetic predisposition for autism,[35] although this varies substantially between cohorts and methodologies. Common variants, as measured by polygenic indices, may play a larger role among phenotypic groups that show relatively more male bias; we have previously shown that the association between an autism diagnosis and an autism polygenic score capturing a proportion of the predisposition from common variants is more pronounced in autistic individuals with few motor and cognitive developmental difficulties than in those with several developmental disabilities.[26] Moreover, we previously saw that the sex difference in polygenic over-transmission (i.e., higher deviation from mid-parental polygenic scores in females) was evident only when examining autistic individuals without cognitive impairment,[26,36] the group with the more pronounced difference in liability thresholds. Notably, we find significantly higher rates of damaging ultra-rare variants in the mothers compared to the fathers of autistic individuals (Figure S15), similar to what was shown for common inherited alleles.[36] However, we do not see significant differences in the effect sizes of these alleles when transmitted to their children (Figure S16).

Although our results are consistent with the different-threshold liability threshold model, alternative models with less restrictive assumptions, e.g., higher variance in males,[4,37] may better capture the true underlying distribution of autism predisposition in the population. While sex differences in autism prevalence may result from combined differences in the mean and variance of the liability (Figure S5B), the observations made in a population-based analysis in ~1 million Swedish individuals best fitted a model with higher variance in males—and also suggested that autism heritability may be lower in females than males.[38] Curiously, the effect sizes of rare inherited damaging variants on the liability scale were significantly higher in males when we tested a model that assumes that the variance in males is ~2–3 times higher than in females (Figure S35; Table S35), although DNMs did not show any significant difference in their effect sizes. We take from this that the effect sizes of damaging DNMs are indeed similar between males and females, whereas better modeling of liability may uncover more nuanced differences in the liability attributed to inherited variants.

With all models, the contribution of any sex differences in the effect size of rare and common variants—or lack thereof—to the sex-biased prevalence of autism should be interpreted in light of the small phenotypic variance they explain.[39] The high familial and twin heritability of autism[38,40,41] (~70%–90%) suggests a major genetic contribution. Rare protein-coding variants, variants in conserved non-coding regions, copy-number alterations, and tandem repeats explain ~10% of the variance.[1,12,42–44] Additive SNP heritability from common variants as estimated from a recent autism genome-wide association study (GWAS)[45] is also ~10%. Finding where the missing heritability lies (e.g., with more powerful association studies across the spectrum of allele frequencies and coding/non-coding variation types) is therefore essential to develop reliable proxies for genetic liability and model sex differences properly. Furthermore, it is also essential to understand non-additive effects and the role of non-genetic predisposition like prenatal exposures (sex hormones), postnatal (social) environment, sex differences in autism presentations, and biases in assessment tools, referral, and diagnosis leading to under-/mis-diagnosis in females.[33,39,46–48] Further insights may be gleaned from studying quantitative autistic traits in population samples.[49,50]

A strength of this study is that we included samples from diverse populations. In theory, since our core analyses were focused on within-family analyses of DNMs and inherited variants, the inclusion of individuals of different ancestries should not create spurious stratification effects. However,

in practice, this resulted in a subtle association of synonymous DNMs with autism diagnosis in SPARK (Figure 1A), discussed in supplemental results sections 2.1.3 and 4.1.3. The imbalance in synonymous DNMs was most prominent between autistic females without motor delay or cognitive impairment and sex-matched siblings not diagnosed with autism (Figure 3B). It is unlikely that this subtle imbalance biased the outcomes for damaging protein-truncating DNMs. Another limitation of our study is that we only examined rare variants on the autosomes, ignoring the sex chromosomes. We note that recent large-scale gene association studies from the ASC did not include sex chromosomes,[1,2] so the contribution of sex-linked genes may be underestimated. Nonetheless, it seems unlikely that large-effect rare variants on the sex chromosomes are a major driver of the sex difference in autism,[17] at least among those with co-occurring motor and cognitive impairments, since our previous work in the Deciphering Developmental Disorders cohort found that rare Mendelian-acting coding variants in the X chromosome contributed similarly in males and females and did not explain the observed 1.6:1 male bias.[32]

To summarize, deleterious *de novo* and rare inherited autosomal coding variants confer similar liability for autism in females and males under a different-threshold model. These variants, particularly *de novo* protein-truncating mutations, increase the liability for co-occurring motor or cognitive impairment significantly more than autism with otherwise typical motor and cognitive development. Autosomal DNMs with large effect sizes are therefore unlikely to explain the observed sex differences in autism prevalence. Future studies with larger sample sizes, considering the contribution of both autosomal and sex-linked alleles across the frequency spectrum of rare and common variants, may capture additional predisposing variants with small effect sizes that contribute to the sex differences in autism.

Data and code availability

This study did not generate new exome or phenotype datasets. The supplemental information contains Figures S1–S35, supplemental results, and supplemental methods. The code used for the main analyses is included in the supplemental methods. Tables S1–S35 (including the data plotted in the figures) are available in a separate supplemental file. SPARK phenotypes and exome data are available for approved users through SFARI Base (https://www.sfari.org/resource/sfari-base/). The ASC data used in this study are available for approved users at NHGRI AnVIL (https://anvilproject.org/data) with the accession ID: phs000298.

Consortia

The members of the Autism Sequencing Consortium are Branko Aleksic, Mykyta Artomov, Mafalda Barbosa, Elisa Benetti, Catalina Betancur, Monica Biscaldi-Schafer, Anders D. Børglum, Harrison Brand, Alfredo Brusco, Joseph D. Buxbaum, Gabriele Campos, Simona Cardaropoli, Diana Carli, Angel Carracedo, Marcus C.Y. Chan, Andreas G. Chiocchetti, Brian H.Y. Chung, Brett Collins, Ryan L. Collins, Edwin H. Cook, Hilary Coon, Claudia I.S. Costa, Michael L. Cuccaro, David J. Cutler, Mark J. Daly, Silvia De Rubeis, Bernie Devlin, Ryan N. Doan, Enrico Domenici, Shan Dong, Chiara Fallerini, Montserrat Fernández-Prieto, Giovanni Battista Ferrero, Christine M. Freitag, Jack M. Fu, J. Jay Gargus, Sherif Gerges, Elisa Giorgio, Ana Cristina Girardi, Stephen Guter, Emily Hansen-Kiss, Gail E. Herman, Irva Hertz-Picciotto, David M. Hougaard, Christina M. Hultman, Suma Jacob, Miia Kaartinen, Lambertus Klei, Alexander Kolevzon, Itaru Kushima, So Lun Lee, Terho Lehtimäki, Lindsay Liang, Carla Lintas, Alicia Ljungdahl, Caterina Lo Rizzo, Yunin Ludena, Patricia Maciel, Behrang Mahjani, Nell Maltman, Marianna Manara, Dara S. Manoach, Gal Meiri, Idan Menashe, Judith Miller, Nancy Minshew, Matthew Mosconi, Rachel Nguyen, Norio Ozaki, Aarno Palotie, Mara Parellada, Maria Rita Passos-Bueno, Lisa Pavinato, Minshi Peng, Margaret Pericak-Vance, Antonio M. Persico, Isaac N. Pessah, Kaija Puura, Abraham Reichenberg, Alessandra Renieri, Kathryn Roeder, Stephan J. Sanders, Sven Sandin, F. Kyle Satterstrom, Stephen W. Scherer, Sabine Schlitt, Rebecca J. Schmidt, Lauren Schmitt, Katja Schneider-Momm, Paige M. Siper, Laura Sloofman, Moyra Smith, Christine R. Stevens, Pål Suren, James S. Sutcliffe, John A. Sweeney, Michael E. Talkowski, Flora Tassone, Karoline Teufel, Elisabetta Trabetti, Slavica Trajkova, Maria del Pilar Trelles, Brie Wamsley, Jaqueline Y.T. Wang, Lauren A. Weiss, Mullin H.C. Yu, and Ryan Yuen.

The members of the APEX Consortium are Deep Adhya, Carrie Allison, Bonnie Ayeung, Rosie Bamford, Simon Baron-Cohen, Richard Bethlehem, Tal Biron-Shental, Graham Burton, Wendy Cowell, Jonathan Davies, Dori Floris, Alice Franklin, Lidia Gabis, Daniel Geschwind, David M. Greenberg, Yuanjun Gu, Alexandra Havdahl, Alexander Heazell, Rosemary Holt, Matthew Hurles, Yumnah Khan, Meng-Chuan Lai, Madeline Lancaster, Michael Lombardo, Hilary Martin, Jose Gonzalez Martinez, Jonathan Mill, Mahmoud Koko Musa, Kathy Niakan, Adam Pavlinek, Lucia Dutan Polit, Marcin Radecki, David Rowitch, Laura Sichlinger, Deepak Srivastava, Alexandros Tsompanidis, Florina Uzefovsky, Varun Warrier, Elizabeth Weir, Xinhe Zhang.

Acknowledgments

We thank the participants and investigators who contributed to the datasets of the Simons Foundation Powering Autism Research for Knowledge (SPARK) project, the Autism Sequencing Consortium (ASC), the Simons Simplex Collection (SSC), and the Lundbeck Foundation Initiative for Integrative Psychiatric Research (iPSYCH) project. This work was supported by the Simons Foundation Autism Research Initiative (SFARI) through grant RNAG/669 G10 9280 to H.M., V.W., and other principal investigators of the Autism Prenatal Sex Differences Consortium (APEX). The ASC received support from SFARI (574598, 736613,

and 647371 to S.J.S.; 575097 to B.D. and K.R.; 573206 to M.E.T. and M.J.D.; 571009 to J.D.; and 606362 and 608540 to M.E.T., M.J.D., J.D.B., B.D., K.R., and S.J.S., all from the consortia author list), NHGRI (HG008895 to M.J.D., S.G., and M.E.T. from the consortia author list), NIMH (MH115957 and MH123155 to M.E.T.; MH111658 and MH057881 to B.D.; MH097849, MH111661, and MH100233 to J.D.B.; MH109900 and MH123184 to K.R.; MH111660 and MH129722 to M.J.D.; and MH111662 and MH100027 to S.J.S., all from the consortia author list), NICHD (HD081256 and HD096326 to M.E.T. from the consortia author list), AMED (JP21WM0425007 to N.O. from the consortia author list), and the Beatrice and Samuel Seaver Foundation. This research was funded in whole, or in part, by the Wellcome Trust (grant number 220540/Z/20/A). For the purpose of Open Access, the authors have applied a CC-BY public copyright licence to any Author Accepted Manuscript version from this submission.

Author contributions

Study design, H.M., V.W., M.K., and the APEX Consortium; samples and data generation, the Autism Sequencing Consortium; quality control and data preparation, M.K. and F.K.S.; analysis, M.K.; writing – draft, M.K. and H.M.; writing – editing and critical revisions, H.M., M.K., F.K.S., and V.W.; study direction and supervision, H.M. and V.W.

Declaration of interests

The authors declare no competing interests.

Web resources

OMIM, http://www.omim.org
NHGRI AnVIL, https://anvilproject.org/data
SFARI Base, https://www.sfari.org/resource/sfari-base/

Supplemental information

Supplemental information can be found online at https://doi.org/10.1016/j.ajhg.2025.01.016.

Received: May 7, 2024
Accepted: January 21, 2025
Published: February 14, 2025

References

1. Fu, J.M., Satterstrom, F.K., Peng, M., Brand, H., Collins, R.L., Dong, S., Wamsley, B., Klei, L., Wang, L., Hao, S.P., et al. (2022). Rare coding variation provides insight into the genetic architecture and phenotypic context of autism. Nat. Genet. *54*, 1320–1331. https://doi.org/10.1038/s41588-022-01104-0.
2. Satterstrom, F.K., Kosmicki, J.A., Wang, J., Breen, M.S., De Rubeis, S., An, J.-Y., Peng, M., Collins, R., Grove, J., Klei, L., et al. (2020). Large-Scale Exome Sequencing Study Implicates Both Developmental and Functional Changes in the Neurobiology of Autism. Cell *180*, 568–584.e23. https://doi.org/10.1016/j.cell.2019.12.036.
3. De Rubeis, S., He, X., Goldberg, A.P., Poultney, C.S., Samocha, K., Cicek, A.E., Kou, Y., Liu, L., Fromer, M., Walker, S., et al. (2014). Synaptic, transcriptional and chromatin genes disrupted in autism. Nature *515*, 209–215. https://doi.org/10.1038/nature13772.
4. Dougherty, J.D., Marrus, N., Maloney, S.E., Yip, B., Sandin, S., Turner, T.N., Selmanovic, D., Kroll, K.L., Gutmann, D.H., Constantino, J.N., and Weiss, L.A. (2022). Can the “female protective effect” liability threshold model explain sex differences in autism spectrum disorder? Neuron *110*, 3243–3262. https://doi.org/10.1016/j.neuron.2022.06.020.
5. Zeidan, J., Fombonne, E., Scorah, J., Ibrahim, A., Durkin, M.S., Saxena, S., Yusuf, A., Shih, A., and Elsabbagh, M. (2022). Global prevalence of autism: A systematic review update. Autism Res. *15*, 778–790. https://doi.org/10.1002/aur.2696.
6. Chaste, P., Roeder, K., and Devlin, B. (2017). The Yin and Yang of Autism Genetics: How Rare De Novo and Common Variations Affect Liability. Annu. Rev. Genomics Hum. Genet. *18*, 167–187. https://doi.org/10.1146/annurev-genom-083115-022647.
7. Cirnigliaro, M., Chang, T.S., Arteaga, S.A., Pérez-Cano, L., Ruzzo, E.K., Gordon, A., Bicks, L.K., Jung, J.-Y., Lowe, J.K., Wall, D.P., and Geschwind, D.H. (2023). The contributions of rare inherited and polygenic risk to ASD in multiplex families. Proc. Natl. Acad. Sci. *120*, e2215632120. https://doi.org/10.1073/pnas.2215632120.
8. Klei, L., McClain, L.L., Mahjani, B., Panayidou, K., De Rubeis, S., Grahnat, A.-C.S., Karlsson, G., Lu, Y., Melhem, N., Xu, X., et al. (2021). How rare and common risk variation jointly affect liability for autism spectrum disorder. Mol. Autism. *12*, 66. https://doi.org/10.1186/s13229-021-00466-2.
9. Carter, C.O. (1961). THE INHERITANCE OF CONGENITAL PYLORIC STENOSIS. Br. Med. Bull. *17*, 251–254. https://doi.org/10.1093/oxfordjournals.bmb.a069918.
10. Falconer, D.S. (1965). The inheritance of liability to certain diseases, estimated from the incidence among relatives. Ann. Hum. Genet. *29*, 51–76. https://doi.org/10.1111/j.1469-1809.1965.tb00500.x.
11. Zhu, C., Ming, M.J., Cole, J.M., Edge, M.D., Kirkpatrick, M., and Harpak, A. (2023). Amplification is the primary mode of gene-by-sex interaction in complex human traits. Cell Genom. *3*, 100297. https://doi.org/10.1016/j.xgen.2023.100297.
12. Antaki, D., Guevara, J., Maihofer, A.X., Klein, M., Gujral, M., Grove, J., Carey, C.E., Hong, O., Arranz, M.J., Hervas, A., et al. (2022). A phenotypic spectrum of autism is attributable to the combined effects of rare variants, polygenic risk and sex. Nat. Genet. *54*, 1284–1292. https://doi.org/10.1038/s41588-022-01064-5.
13. Chan, A.J.S., Engchuan, W., Reuter, M.S., Wang, Z., Thiruvahindrapuram, B., Trost, B., Nalpathamkalam, T., Negrijn, C., Lamoureux, S., Pellecchia, G., et al. (2022). Genome-wide rare variant score associates with morphological subtypes of autism spectrum disorder. Nat. Commun. *13*, 6463. https://doi.org/10.1038/s41467-022-34112-z.
14. Zhou, X., Feliciano, P., Shu, C., Wang, T., Astrovskaya, I., Hall, J.B., Obiajulu, J.U., Wright, J.R., Murali, S.C., Xu, S.X., et al. (2022). Integrating de novo and inherited variants in 42,607 autism cases identifies mutations in new moderate-risk genes. Nat. Genet. *54*, 1305–1319. https://doi.org/10.1038/s41588-022-01148-2.
15. Loomes, R., Hull, L., and Mandy, W.P.L. (2017). What Is the Male-to-Female Ratio in Autism Spectrum Disorder? A Systematic Review and Meta-Analysis. J. Am. Acad. Child Adolesc. Psychiatry *56*, 466–474. https://doi.org/10.1016/j.jaac.2017.03.013.

16. Ratto, A.B., Kenworthy, L., Yerys, B.E., Bascom, J., Wieckowski, A.T., White, S.W., Wallace, G.L., Pugliese, C., Schultz, R.T., Ollendick, T.H., et al. (2018). What About the Girls? Sex-Based Differences in Autistic Traits and Adaptive Skills. J. Autism Dev. Disord. *48*, 1698–1711. https://doi.org/10.1007/s10803-017-3413-9.
17. Jacquemont, S., Coe, B.P., Hersch, M., Duyzend, M.H., Krumm, N., Bergmann, S., Beckmann, J.S., Rosenfeld, J.A., and Eichler, E.E. (2014). A Higher Mutational Burden in Females Supports a "Female Protective Model" in Neurodevelopmental Disorders. Am. J. Hum. Genet. *94*, 415–425. https://doi.org/10.1016/j.ajhg.2014.02.001.
18. Zhang, Y., Li, N., Li, C., Zhang, Z., Teng, H., Wang, Y., Zhao, T., Shi, L., Zhang, K., Xia, K., et al. (2020). Genetic evidence of gender difference in autism spectrum disorder supports the female-protective effect. Transl. Psychiatry *10*, 4. https://doi.org/10.1038/s41398-020-0699-8.
19. Maenner, M.J., Shaw, K.A., Bakian, A.V., Bilder, D.A., Durkin, M.S., Esler, A., Furnier, S.M., Hallas, L., Hall-Lande, J., Hudson, A., et al. (2021). Prevalence and Characteristics of Autism Spectrum Disorder Among Children Aged 8 Years — Autism and Developmental Disabilities Monitoring Network, 11 Sites, United States, 2018. MMWR. Surveill. Summ. *70*, 1–16. https://doi.org/10.15585/mmwr.ss7011a1.
20. Abrahams, B.S., Arking, D.E., Campbell, D.B., Mefford, H.C., Morrow, E.M., Weiss, L.A., Menashe, I., Wadkins, T., Banerjee-Basu, S., and Packer, A. (2013). SFARI Gene 2.0: a community-driven knowledgebase for the autism spectrum disorders (ASDs). Mol. Autism. *4*, 36. https://doi.org/10.1186/2040-2392-4-36.
21. Shu, C., Green Snyder, L., Shen, Y., Chung, W.K.; and SPARK Consortium (2022). Imputing cognitive impairment in SPARK, a large autism cohort. Autism Res. *15*, 156–170. https://doi.org/10.1002/aur.2622.
22. Kissel, L.T., Pochareddy, S., An, J.-Y., Sestan, N., Sanders, S.J., Wang, X., and Werling, D.M. (2024). Sex-Differential Gene Expression in Developing Human Cortex and Its Intersection With Autism Risk Pathways. Biol. Psychiatry Glob. Open Sci. *4*, 100321. https://doi.org/10.1016/j.bpsgos.2024.100321.
23. Fass, S.B., Mulvey, B., Chase, R., Yang, W., Selmanovic, D., Chaturvedi, S.M., Tycksen, E., Weiss, L.A., and Dougherty, J.D. (2024). Relationship between sex biases in gene expression and sex biases in autism and Alzheimer's disease. Biol. Sex Differ. *15*, 47. https://doi.org/10.1186/s13293-024-00622-2.
24. McKenzie, K., Milton, M., Smith, G., and Ouellette-Kuntz, H. (2016). Systematic Review of the Prevalence and Incidence of Intellectual Disabilities: Current Trends and Issues. Curr. Dev. Disord. Rep. *3*, 104–115. https://doi.org/10.1007/s40474-016-0085-7.
25. Piton, A., Redin, C., and Mandel, J.-L. (2013). XLID-Causing Mutations and Associated Genes Challenged in Light of Data From Large-Scale Human Exome Sequencing. Am. J. Hum. Genet. *93*, 368–383. https://doi.org/10.1016/j.ajhg.2013.06.013.
26. Warrier, V., Zhang, X., Reed, P., Havdahl, A., Moore, T.M., Cliquet, F., Leblond, C.S., Rolland, T., Rosengren, A.; and EU-AIMS LEAP (2022). Genetic correlates of phenotypic heterogeneity in autism. Nat. Genet. *54*, 1293–1304. https://doi.org/10.1038/s41588-022-01072-5.
27. Ouwenga, R.L., and Dougherty, J. (2015). Fmrp targets or not: long, highly brain-expressed genes tend to be implicated in autism and brain disorders. Mol. Autism. *6*, 16. https://doi.org/10.1186/s13229-015-0008-1.
28. Wright, C.F., Fitzgerald, T.W., Jones, W.D., Clayton, S., McRae, J.F., van Kogelenberg, M., King, D.A., Ambridge, K., Barrett, D.M., Bayzetinova, T., et al. (2015). Genetic diagnosis of developmental disorders in the DDD study: a scalable analysis of genome-wide research data. Lancet *385*, 1305–1314. https://doi.org/10.1016/S0140-6736(14)61705-0.
29. Abrahams, B.S., and Geschwind, D.H. (2008). Advances in autism genetics: on the threshold of a new neurobiology. Nat. Rev. Genet. *9*, 341–355. https://doi.org/10.1038/nrg2346.
30. Maulik, P.K., Mascarenhas, M.N., Mathers, C.D., Dua, T., and Saxena, S. (2011). Prevalence of intellectual disability: A meta-analysis of population-based studies. Res. Dev. Disabil. *32*, 419–436. https://doi.org/10.1016/j.ridd.2010.12.018.
31. Nair, R., Chen, M., Dutt, A.S., Hagopian, L., Singh, A., and Du, M. (2022). Significant regional inequalities in the prevalence of intellectual disability and trends from 1990 to 2019: a systematic analysis of GBD 2019. Epidemiol. Psychiatr. Sci. *31*, e91. https://doi.org/10.1017/S2045796022000701.
32. Martin, H.C., Gardner, E.J., Samocha, K.E., Kaplanis, J., Akawi, N., Sifrim, A., Eberhardt, R.Y., Tavares, A.L.T., Neville, M.D.C., Niemi, M.E.K., et al. (2021). The contribution of X-linked coding variation to severe developmental disorders. Nat. Commun. *12*, 627. https://doi.org/10.1038/s41467-020-20852-3.
33. Werling, D.M., and Geschwind, D.H. (2013). Sex differences in autism spectrum disorders. Curr. Opin. Neurol. *26*, 146–153. https://doi.org/10.1097/WCO.0b013e32835ee548.
34. Dalsgaard, S., Thorsteinsson, E., Trabjerg, B.B., Schullehner, J., Plana-Ripoll, O., Brikell, I., Wimberley, T., Thygesen, M., Madsen, K.B., Timmerman, A., et al. (2020). Incidence Rates and Cumulative Incidences of the Full Spectrum of Diagnosed Mental Disorders in Childhood and Adolescence. JAMA Psychiatr. *77*, 155–164. https://doi.org/10.1001/jamapsychiatry.2019.3523.
35. Gaugler, T., Klei, L., Sanders, S.J., Bodea, C.A., Goldberg, A.P., Lee, A.B., Mahajan, M., Manaa, D., Pawitan, Y., Reichert, J., et al. (2014). Most genetic risk for autism resides with common variation. Nat. Genet. *46*, 881–885. https://doi.org/10.1038/ng.3039.
36. Wigdor, E.M., Weiner, D.J., Grove, J., Fu, J.M., Thompson, W.K., Carey, C.E., Baya, N., Van Der Merwe, C., Walters, R.K., Satterstrom, F.K., et al. (2022). The female protective effect against autism spectrum disorder. Cell Genom. *2*, 100134. https://doi.org/10.1016/j.xgen.2022.100134.
37. Falconer, D.S. (1967). The inheritance of liability to diseases with variable age of onset, with particular reference to diabetes mellitus. Ann. Hum. Genet. *31*, 1–20. https://doi.org/10.1111/j.1469-1809.1967.tb02015.x.
38. Sandin, S., Yip, B.H.K., Yin, W., Weiss, L.A., Dougherty, J.D., Fass, S., Constantino, J.N., Hailin, Z., Turner, T.N., Marrus, N., et al. (2024). Examining Sex Differences in Autism Heritability. JAMA Psychiatr. *81*, 673–680. https://doi.org/10.1001/jamapsychiatry.2024.0525.
39. Havdahl, A., Niarchou, M., Starnawska, A., Uddin, M., Van Der Merwe, C., and Warrier, V. (2021). Genetic contributions to autism spectrum disorder. Psychol. Med. *51*, 2260–2273. https://doi.org/10.1017/S0033291721000192.
40. Sandin, S., Lichtenstein, P., Kuja-Halkola, R., Hultman, C., Larsson, H., and Reichenberg, A. (2017). The Heritability of

Autism Spectrum Disorder. JAMA *318*, 1182–1184. https://doi.org/10.1001/jama.2017.12141.

41. Tick, B., Bolton, P., Happé, F., Rutter, M., and Rijsdijk, F. (2016). Heritability of autism spectrum disorders: a meta-analysis of twin studies. J. Child Psychol. Psychiatry *57*, 585–595. https://doi.org/10.1111/jcpp.12499.
42. Pinto, D., Pagnamenta, A.T., Klei, L., Anney, R., Merico, D., Regan, R., Conroy, J., Magalhaes, T.R., Correia, C., Abrahams, B.S., et al. (2010). Functional impact of global rare copy number variation in autism spectrum disorders. Nature *466*, 368–372. https://doi.org/10.1038/nature09146.
43. Sanders, S.J., He, X., Willsey, A.J., Ercan-Sencicek, A.G., Samocha, K.E., Cicek, A.E., Murtha, M.T., Bal, V.H., Bishop, S.L., Dong, S., et al. (2015). Insights into Autism Spectrum Disorder Genomic Architecture and Biology from 71 Risk Loci. Neuron *87*, 1215–1233. https://doi.org/10.1016/j.neuron.2015.09.016.
44. Trost, B., Engchuan, W., Nguyen, C.M., Thiruvahindrapuram, B., Dolzhenko, E., Backstrom, I., Mirceta, M., Mojarad, B.A., Yin, Y., Dov, A., et al. (2020). Genome-wide detection of tandem DNA repeats that are expanded in autism. Nature *586*, 80–86. https://doi.org/10.1038/s41586-020-2579-z.
45. Grove, J., Ripke, S., Als, T.D., Mattheisen, M., Walters, R.K., Won, H., Pallesen, J., Agerbo, E., Andreassen, O.A., Anney, R., et al. (2019). Identification of common genetic risk variants for autism spectrum disorder. Nat. Genet. *51*, 431–444. https://doi.org/10.1038/s41588-019-0344-8.
46. Mitra, I., Tsang, K., Ladd-Acosta, C., Croen, L.A., Aldinger, K.A., Hendren, R.L., Traglia, M., Lavillaureix, A., Zaitlen, N., Oldham, M.C., et al. (2016). Pleiotropic Mechanisms Indicated for Sex Differences in Autism. PLoS Genet. *12*, e1006425. https://doi.org/10.1371/journal.pgen.1006425.
47. Baron-Cohen, S., Tsompanidis, A., Auyeung, B., Nørgaard-Pedersen, B., Hougaard, D.M., Abdallah, M., Cohen, A., and Pohl, A. (2020). Foetal oestrogens and autism. Mol. Psychiatry *25*, 2970–2978. https://doi.org/10.1038/s41380-019-0454-9.
48. Baron-Cohen, S., Auyeung, B., Nørgaard-Pedersen, B., Hougaard, D.M., Abdallah, M.W., Melgaard, L., Cohen, A.S., Chakrabarti, B., Ruta, L., and Lombardo, M.V. (2015). Elevated fetal steroidogenic activity in autism. Mol. Psychiatry *20*, 369–376. https://doi.org/10.1038/mp.2014.48.
49. Lyall, K. (2023). What are quantitative traits and how can they be used in autism research? Autism Res. *16*, 1289–1298. https://doi.org/10.1002/aur.2937.
50. Robinson, E.B., Koenen, K.C., McCormick, M.C., Munir, K., Hallett, V., Happé, F., Plomin, R., and Ronald, A. (2011). Evidence That Autistic Traits Show the Same Etiology in the General Population and at the Quantitative Extremes (5%, 2.5%, and 1%). Arch. Gen. Psychiatry *68*, 1113–1121. https://doi.org/10.1001/archgenpsychiatry.2011.119.

The American Journal of Human Genetics, Volume *112*

Supplemental information

Contribution of autosomal rare and *de novo* variants to sex differences in autism

Mahmoud Koko, F. Kyle Satterstrom, Autism Sequencing Consortium, APEX consortium, Varun Warrier, and Hilary Martin

Consortia members and affiliations

Autism Sequencing Consortium (ASC)

- Branko Alexie: Department of Psychiatry, Graduate School of Medicine, Nagoya University, Nagoya, Japan
- Mykyta Artomov: Center for Genomic Medicine, Massachusetts General Hospital, Boston, MA, USA; Program in Medical and Population Genetics, Broad Institute of MIT and Harvard, Cambridge, MA, USA; Stanley Center for Psychiatric Research, Broad Institute of MIT and Harvard, Cambridge, MA, USA; Harvard Medical School, Boston, MA, USA
- Mafalda Barbosa: The Mindich Child Health and Development Institute, Icahn School of Medicine at Mount Sinai, New York, NY, USA; Department of Genetics and Genomic Sciences, Icahn School of Medicine at Mount Sinai, New York, NY, USA
- Elisa Benetti: Med Biotech Hub and Competence Center, Department of Medical Biotechnologies, University of Siena, Siena, Italy; Medical Genetics, , University of Siena, Siena, Italy
- Catalina Betancur: Sorbonne Université, INSERM, CNRS, Neuroscience Paris Seine, Institut de Biologie Paris Seine, Paris, France
- Monica Biscaldi-Schafer: Department of Child and Adolescent Psychiatry, Psychosomatics and Psychotherapy, Goethe University Frankfurt, Frankfurt, Germany
- Anders D. Børglum: The Lundbeck Foundation Initiative for Integrative Psychiatric Research, iPSYCH, Aarhus, Denmark; Department of Biomedicine—Human Genetics, Aarhus University, Aarhus, Denmark; Center for Genomics and Personalized Medicine, Aarhus, Denmark; Bioinformatics Research Centre, Aarhus University, Aarhus, Denmark
- Harrison Brand: Center for Genomic Medicine, Massachusetts General Hospital, Boston, MA, USA; Program in Medical and Population Genetics, Broad Institute of MIT and Harvard, Cambridge, MA, USA; Department of Neurology, Massachusetts General Hospital and Harvard Medical School, Boston, MA, USA; Pediatric Surgical Research Laboratories, Department of Surgery, Massachusetts General Hospital, Boston, MA, USA
- Alfredo Brusco: Department of Medical Sciences, University of Torino, Turin, Italy; Medical Genetics Unit, ‘Città della Salute e della Scienza’ University Hospital, Turin, Italy
- Joseph D. Buxbaum: Seaver Autism Center for Research and Treatment, Icahn School of Medicine at Mount Sinai, New York, NY, USA; Department of Psychiatry, Icahn School of Medicine at Mount Sinai, New York, NY, USA; The Mindich Child Health and Development Institute, Icahn School of Medicine at Mount Sinai, New York, NY, USA; Department of

Genetics and Genomic Sciences, Icahn School of Medicine at Mount Sinai, New York, NY, USA; Friedman Brain Institute, Icahn School of Medicine at Mount Sinai, New York, NY, USA; Department of Neuroscience, Icahn School of Medicine at Mount Sinai, New York, NY, USA

- Gabriele Campos: Centro de Pesquisas sobre o Genoma Humano e Células tronco, Instituto de Biociências, Universidade de São Paulo, São Paulo, Brazil
- Simona Cardaropoli: Department of Public Health and Pediatrics, University of Torino, Turin, Italy
- Diana Carli: Department of Public Health and Pediatrics, University of Torino, Turin, Italy
- Angel Carracedo: Grupo de Medicina Xenómica, Centro de Investigación en Red de Enfermedades Raras (CIBERER), CIMUS, Universidade de Santiago de Compostela, Santiago de Compostela, Spain; Fundación Pública Galega de Medicina Xenómica, Servicio Galego de Saúde (SERGAS), Santiago de Compostela, Spain
- Marcus C. Y. Chan: Department of Pediatrics and Adolescent Medicine, Duchess of Kent Children’s Hospital, The University of Hong Kong, Hong Kong Special Administrative Region, China
- Andreas G. Chiocchetti: Department of Psychiatry, Graduate School of Medicine, Nagoya University, Nagoya, Japan
- Brian H. Y. Chung: Department of Pediatrics and Adolescent Medicine, Duchess of Kent Children’s Hospital, The University of Hong Kong, Hong Kong Special Administrative Region, China
- Brett Collins: Seaver Autism Center for Research and Treatment, Icahn School of Medicine at Mount Sinai, New York, NY, USA; Department of Psychiatry, Icahn School of Medicine at Mount Sinai, New York, NY, USA; The Mindich Child Health and Development Institute, Icahn School of Medicine at Mount Sinai, New York, NY, USA
- Ryan L. Collins: Center for Genomic Medicine, Massachusetts General Hospital, Boston, MA, USA; Program in Medical and Population Genetics, Broad Institute of MIT and Harvard, Cambridge, MA, USA; Department of Neurology, Massachusetts General Hospital and Harvard Medical School, Boston, MA, USA; Program in Bioinformatics and Integrative Genomics, Harvard Medical School, Boston, MA, USA
- Edwin H. Cook: Institute for Juvenile Research, Department of Psychiatry, University of Illinois at Chicago, Chicago, IL, USA
- Hilary Coon: Department of Internal Medicine, University of Utah, Salt Lake City, UT, USA; Department of Psychiatry, Huntsman Mental Health Institute, University of Utah, Salt Lake City, UT, USA
- Claudia I. S. Costa: Centro de Pesquisas sobre o Genoma Humano e Células tronco, Instituto de Biociências, Universidade de São Paulo, São Paulo, Brazil

- Michael L. Cuccaro: The John P Hussman Institute for Human Genomics, The University of Miami Miller School of Medicine, Miami, FL, USA
- David J. Cutler: Department of Human Genetics, Emory University School of Medicine, Atlanta, GA, USA
- Mark J. Daly: Center for Genomic Medicine, Massachusetts General Hospital, Boston, MA, USA; Program in Medical and Population Genetics, Broad Institute of MIT and Harvard, Cambridge, MA, USA; Stanley Center for Psychiatric Research, Broad Institute of MIT and Harvard, Cambridge, MA, USA; Analytic and Translational Genetics Unit, Department of Medicine, Massachusetts General Hospital, Boston, MA, USA; Harvard Medical School, Boston, MA, USA; Institute for Molecular Medicine Finland (FIMM), University of Helsinki, Helsinki, Finland
- Silvia De Rubeis: Seaver Autism Center for Research and Treatment, Icahn School of Medicine at Mount Sinai, New York, NY, USA; Department of Psychiatry, Icahn School of Medicine at Mount Sinai, New York, NY, USA; The Mindich Child Health and Development Institute, Icahn School of Medicine at Mount Sinai, New York, NY, USA; Friedman Brain Institute, Icahn School of Medicine at Mount Sinai, New York, NY, USA
- Bernie Devlin: Department of Psychiatry, University of Pittsburgh School of Medicine, Pittsburgh, PA, USA
- Ryan N. Doan: Division of Genetics and Genomics, Boston Children's Hospital, Boston, MA, USA
- Enrico Domenici: Department of Cellular, Computational and Integrative Biology, , University of Trento, Trento, Italy
- Shan Dong: Department of Psychiatry, UCSF Weill Institute for Neurosciences, University of California San Francisco, San Francisco, CA, USA
- Chiara Fallerini: Med Biotech Hub and Competence Center, Department of Medical Biotechnologies, University of Siena, Siena, Italy; Medical Genetics, , University of Siena, Siena, Italy
- Montserrat Fernández-Prieto: Grupo de Medicina Xenómica, Centro de Investigación en Red de Enfermedades Raras (CIBERER), CIMUS, Universidade de Santiago de Compostela, Santiago de Compostela, Spain; Neurogenetics group, Instituto de Investigación Sanitaria de Santiago (IDIS-SERGAS), Santiago de Compostela, Spain
- Giovanni Battista Ferrero: Department of Public Health and Pediatrics, University of Torino, Turin, Italy
- Christine M. Freitag: Department of Psychiatry, Graduate School of Medicine, Nagoya University, Nagoya, Japan
- Jack M. Fu: Center for Genomic Medicine, Massachusetts General Hospital, Boston, MA, USA; Program in Medical and Population Genetics, Broad Institute of MIT and Harvard,

Cambridge, MA, USA; Department of Neurology, Massachusetts General Hospital and Harvard Medical School, Boston, MA, USA

- J. Jay Gargus: Center for Autism Research and Translation, University of California Irvine, Irvine, CA, USA
- Sherif Gerges: Center for Genomic Medicine, Massachusetts General Hospital, Boston, MA, USA; Program in Medical and Population Genetics, Broad Institute of MIT and Harvard, Cambridge, MA, USA; Stanley Center for Psychiatric Research, Broad Institute of MIT and Harvard, Cambridge, MA, USA; Harvard Medical School, Boston, MA, USA
- Elisa Giorgio: Department of Medical Sciences, University of Torino, Turin, Italy
- Ana Cristina Girardi: Centro de Pesquisas sobre o Genoma Humano e Células tronco, Instituto de Biociências, Universidade de São Paulo, São Paulo, Brazil
- Stephen Guter: Institute for Juvenile Research, Department of Psychiatry, University of Illinois at Chicago, Chicago, IL, USA
- Emily Hansen-Kiss: Department of Diagnostic and Biomedical Sciences, University of Texas Health Science Center at Houston, School of Dentistry, Houston, TX, USA
- Gail E. Herman: The Research Institute at Nationwide Children's Hospital, Columbus, OH, USA
- Irva Hertz-Picciotto: MIND (Medical Investigation of Neurodevelopmental Disorders) Institute, University of California Davis, Davis, CA, USA
- David M. Hougaard: Department of Child and Adolescent Psychiatry, Psychosomatics and Psychotherapy, Goethe University Frankfurt, Frankfurt, Germany; Center for Neonatal Screening, Department for Congenital Disorders, Statens Serum Institut, Copenhagen, Denmark
- Christina M. Hultman: Department of Medical Epidemiology and Biostatistics, Karolinska Institutet, Stockholm, Sweden
- Suma Jacob: Institute for Juvenile Research, Department of Psychiatry, University of Illinois at Chicago, Chicago, IL, USA
- Miia Kaartinen: Department of Child Psychiatry, Tampere University and Tampere University Hospital, Tampere, Finland
- Lambertus Klei: Department of Psychiatry, University of Pittsburgh School of Medicine, Pittsburgh, PA, USA
- Alexander Kolevzon: Seaver Autism Center for Research and Treatment, Icahn School of Medicine at Mount Sinai, New York, NY, USA; Department of Psychiatry, Icahn School of Medicine at Mount Sinai, New York, NY, USA; Department of Pediatrics, Icahn School of Medicine at Mount Sinai, New York, NY, USA

- Itaru Kushima: The Lundbeck Foundation Initiative for Integrative Psychiatric Research, iPSYCH, Aarhus, Denmark; Medical Genomics Center, Nagoya University Hospital, Nagoya, Japan
- So Lun Lee: Department of Pediatrics and Adolescent Medicine, Duchess of Kent Children’s Hospital, The University of Hong Kong, Hong Kong Special Administrative Region, China
- Terho Lehtimäki: Department of Clinical Chemistry, Fimlab Laboratories and Finnish Cardiovascular Research Center-Tampere, Faculty of Medicine and Health Technology, Tampere University, Tampere, Finland
- Lindsay Liang: Department of Psychiatry, UCSF Weill Institute for Neurosciences, University of California San Francisco, San Francisco, CA, USA
- Carla Lintas: Service for Neurodevelopmental Disorders, University Campus Bio-medico of Rome, Rome, Italy
- Alicia Ljungdahl: Department of Psychiatry, UCSF Weill Institute for Neurosciences, University of California San Francisco, San Francisco, CA, USA
- Caterina Lo Rizzo: Medical Genetics, , University of Siena, Siena, Italy; Genetica Medica, Azienda Ospedaliera Universitaria Senese, Siena, Italy
- Yunin Ludena: MIND (Medical Investigation of Neurodevelopmental Disorders) Institute, University of California Davis, Davis, CA, USA
- Patricia Maciel: Life and Health Sciences Research Institute, School of Medicine, University of Minho, Braga, Portugal
- Behrang Mahjani: Seaver Autism Center for Research and Treatment, Icahn School of Medicine at Mount Sinai, New York, NY, USA; Department of Psychiatry, Icahn School of Medicine at Mount Sinai, New York, NY, USA; Department of Medical Epidemiology and Biostatistics, Karolinska Institutet, Stockholm, Sweden
- Nell Maltman: Institute for Juvenile Research, Department of Psychiatry, University of Illinois at Chicago, Chicago, IL, USA
- Marianna Manara: Medical Genetics, , University of Siena, Siena, Italy; Genetica Medica, Azienda Ospedaliera Universitaria Senese, Siena, Italy
- Dara S. Manoach: Department of Psychiatry, Massachusetts General Hospital and Harvard Medical School, Boston, MA, USA
- Gal Meiri: The Azrieli National Center for Autism and Neurodevelopment Research, Ben-Gurion University of the Negev, Beer-Sheva, Israel; Pre-School Psychiatry Unit, Soroka University Medical Center, Beer Sheva, Israel
- Idan Menashe: Department of Public Health, Ben-Gurion University of the Negev, Beer-Sheva, Israel; National Autism Research Center of Israel, Ben-Gurion University of the Negev, Beer-Sheva, Israel

- Judith Miller: Children's Hospital of Philadelphia, Philadelphia, PA, USA; Department of Psychiatry, University of Utah, Salt Lake City, UT, USA
- Nancy Minshew: Department of Psychiatry, University of Pittsburgh School of Medicine, Pittsburgh, PA, USA
- Matthew Mosconi: Life Span Institute and Kansas Center for Autism Research and Training, University of Kansas, Lawrence, KS, USA
- Rachel Nguyen: Center for Autism Research and Translation, University of California Irvine, Irvine, CA, USA
- Norio Ozaki: The Lundbeck Foundation Initiative for Integrative Psychiatric Research, iPSYCH, Aarhus, Denmark; Institute for Glyco-core Research (iGCORE), Nagoya University, Nagoya, Japan
- Aarno Palotie: Program in Medical and Population Genetics, Broad Institute of MIT and Harvard, Cambridge, MA, USA; Analytic and Translational Genetics Unit, Department of Medicine, Massachusetts General Hospital, Boston, MA, USA; Institute for Molecular Medicine Finland (FIMM), University of Helsinki, Helsinki, Finland; Psychiatric & Neurodevelopmental Genetics Unit, Department of Psychiatry, Massachusetts General Hospital, Boston, MA, USA
- Mara Parellada: Department of Child and Adolescent Psychiatry, Hospital General Universitario Gregorio Marañón, IiSGM, CIBERSAM, School of Medicine Complutense University, Madrid, Spain
- Maria Rita Passos-Bueno: Centro de Pesquisas sobre o Genoma Humano e Células tronco, Instituto de Biociências, Universidade de São Paulo, São Paulo, Brazil
- Lisa Pavinato: Department of Medical Sciences, University of Torino, Turin, Italy
- Minshi Peng: Department of Statistics and Data Science, Carnegie Mellon University, Pittsburgh, PA, USA;
- Margaret Pericak-Vance: The John P Hussman Institute for Human Genomics, The University of Miami Miller School of Medicine, Miami, FL, USA
- Antonio M. Persico: Interdepartmental Program 'Autism 0-90', 'Gaetano Martino' University Hospital, University of Messina, Messina, Italy
- Isaac N. Pessah: MIND (Medical Investigation of Neurodevelopmental Disorders) Institute, University of California Davis, Davis, CA, USA; Department of Molecular Biosciences, University of California Davis, School of Veterinary Medicine, Davis, CA, USA
- Kaija Puura: Department of Child Psychiatry, Tampere University and Tampere University Hospital, Tampere, Finland
- Abraham Reichenberg: Seaver Autism Center for Research and Treatment, Icahn School of Medicine at Mount Sinai, New York, NY, USA; Department of Psychiatry, Icahn School

of Medicine at Mount Sinai, New York, NY, USA; The Mindich Child Health and Development Institute, Icahn School of Medicine at Mount Sinai, New York, NY, USA; Department of Environmental Medicine and Public Health, Icahn School of Medicine at Mount Sinai, New York, NY, USA

- Alessandra Renieri: Med Biotech Hub and Competence Center, Department of Medical Biotechnologies, University of Siena, Siena, Italy; Medical Genetics, , University of Siena, Siena, Italy; Genetica Medica, Azienda Ospedaliera Universitaria Senese, Siena, Italy
- Kathryn Roeder: Department of Statistics and Data Science, Carnegie Mellon University, Pittsburgh, PA, USA; Computational Biology Department, Carnegie Mellon University, Pittsburgh, PA, USA
- Stephan J. Sanders: Department of Psychiatry, UCSF Weill Institute for Neurosciences, University of California San Francisco, San Francisco, CA, USA
- Sven Sandin: Seaver Autism Center for Research and Treatment, Icahn School of Medicine at Mount Sinai, New York, NY, USA; Department of Psychiatry, Icahn School of Medicine at Mount Sinai, New York, NY, USA; Department of Medical Epidemiology and Biostatistics, Karolinska Institutet, Stockholm, Sweden
- F. Kyle Satterstrom: Program in Medical and Population Genetics, Broad Institute of MIT and Harvard, Cambridge, MA, USA; Stanley Center for Psychiatric Research, Broad Institute of MIT and Harvard, Cambridge, MA, USA; Analytic and Translational Genetics Unit, Department of Medicine, Massachusetts General Hospital, Boston, MA, USA
- Stephen W. Scherer: Program in Genetics and Genome Biology, The Centre for Applied Genomics, The Hospital for Sick Children, Toronto, Ontario, Canada; Department of Molecular Genetics and McLaughlin Centre, University of Toronto, Toronto, Ontario, Canada
- Sabine Schlitt: Department of Psychiatry, Graduate School of Medicine, Nagoya University, Nagoya, Japan
- Rebecca J. Schmidt: MIND (Medical Investigation of Neurodevelopmental Disorders) Institute, University of California Davis, Davis, CA, USA
- Lauren Schmitt: Institute for Juvenile Research, Department of Psychiatry, University of Illinois at Chicago, Chicago, IL, USA
- Katja Schneider-Momm: Department of Psychiatry, Graduate School of Medicine, Nagoya University, Nagoya, Japan
- Paige M. Siper: Seaver Autism Center for Research and Treatment, Icahn School of Medicine at Mount Sinai, New York, NY, USA; Department of Psychiatry, Icahn School of Medicine at Mount Sinai, New York, NY, USA; The Mindich Child Health and Development Institute, Icahn School of Medicine at Mount Sinai, New York, NY, USA

- Laura Sloofman: Seaver Autism Center for Research and Treatment, Icahn School of Medicine at Mount Sinai, New York, NY, USA; Department of Psychiatry, Icahn School of Medicine at Mount Sinai, New York, NY, USA; The Mindich Child Health and Development Institute, Icahn School of Medicine at Mount Sinai, New York, NY, USA
- Moyra Smith: Center for Autism Research and Translation, University of California Irvine, Irvine, CA, USA
- Christine R. Stevens: Program in Medical and Population Genetics, Broad Institute of MIT and Harvard, Cambridge, MA, USA; Stanley Center for Psychiatric Research, Broad Institute of MIT and Harvard, Cambridge, MA, USA; Analytic and Translational Genetics Unit, Department of Medicine, Massachusetts General Hospital, Boston, MA, USA
- Pål Suren: Norwegian Institute of Public Health, Oslo, Norway
- James S. Sutcliffe: Department of Molecular Physiology & Biophysics and Psychiatry, Vanderbilt University School of Medicine, Nashville, TN, USA; Vanderbilt Genetics Institute, Vanderbilt University School of Medicine, Nashville, TN, USA
- John A. Sweeney: Department of Psychiatry, University of Cincinnati, Cincinnati, OH, USA
- Michael E. Talkowski: Center for Genomic Medicine, Massachusetts General Hospital, Boston, MA, USA; Program in Medical and Population Genetics, Broad Institute of MIT and Harvard, Cambridge, MA, USA; Department of Neurology, Massachusetts General Hospital and Harvard Medical School, Boston, MA, USA; Stanley Center for Psychiatric Research, Broad Institute of MIT and Harvard, Cambridge, MA, USA; Program in Bioinformatics and Integrative Genomics, Harvard Medical School, Boston, MA, USA
- Flora Tassone: MIND (Medical Investigation of Neurodevelopmental Disorders) Institute, University of California Davis, Davis, CA, USA; Department of Biochemistry and Molecular Medicine, University of California Davis, School of Medicine, Sacramento, CA, USA
- Karoline Teufel: Department of Psychiatry, Graduate School of Medicine, Nagoya University, Nagoya, Japan
- Elisabetta Trabetti: Department of Neurosciences, Biomedicine and Movement Sciences, Section of Biology and Genetics, University of Verona, Verona, Italy
- Slavica Trajkova: Department of Medical Sciences, University of Torino, Turin, Italy
- Maria del Pilar Trelles: Seaver Autism Center for Research and Treatment, Icahn School of Medicine at Mount Sinai, New York, NY, USA; Department of Psychiatry, Icahn School of Medicine at Mount Sinai, New York, NY, USA
- Brie Wamsley: Program in Neurogenetics, Department of Neurology, David Geffen School of Medicine, University of California Los Angeles, Los Angeles, CA, USA
- Jaqueline Y. T. Wang: Centro de Pesquisas sobre o Genoma Humano e Células tronco, Instituto de Biociências, Universidade de São Paulo, São Paulo, Brazil

- Lauren A. Weiss: Department of Psychiatry, UCSF Weill Institute for Neurosciences, University of California San Francisco, San Francisco, CA, USA
- Mullin H. C. Yu : Department of Pediatrics and Adolescent Medicine, Duchess of Kent Children's Hospital, The University of Hong Kong, Hong Kong Special Administrative Region, China
- Ryan Yuen: Program in Genetics and Genome Biology, The Centre for Applied Genomics, The Hospital for Sick Children, Toronto, Ontario, Canada

Autism Prenatal and Sex Differences (APEX)

- Dwaipayan Adhya: Department of Psychiatry, Autism Research Centre, University of Cambridge, Cambridge, Cambridgeshire, CB2 8AH, UK; Department of Basic and Clinical Neuroscience, Maurice Wohl Clinical Neuroscience Institute, Institute of Psychiatry, Psychology and Neuroscience, King's College London, London, UK
- Carrie Allison: Department of Psychiatry, Autism Research Centre, University of Cambridge, Cambridge, Cambridgeshire, CB2 8AH, UK
- Bonnie Ayeung: Department of Psychiatry, Autism Research Centre, University of Cambridge, Cambridge, Cambridgeshire, CB2 8AH, UK
- Rosie Bamford: University of Exeter Medical School, Exeter, Devon, EX2 5DW, UK
- Simon Baron-Cohen: Department of Psychiatry, Autism Research Centre, University of Cambridge, Cambridge, Cambridgeshire, CB2 8AH, UK
- Richard Bethlehem: Department of Psychiatry, Autism Research Centre, University of Cambridge, Cambridge, Cambridgeshire, CB2 8AH, UK; Department of Psychiatry, Brain Mapping Unit, University of Cambridge, Cambridge, Cambridgeshire, CB2 8AH, UK
- Tal Biron-Shental: Department of Obstetrics and Gynecology, Meir Medical Center, Kfar Saba, Israel; Sackler Faculty of Medicine, Tel Aviv University, Tel Aviv, Israel
- Graham Burton: Centre for Trophoblast Research, Department of Physiology, Development and Neuroscience, University of Cambridge, Cambridge, CB2 3EG, UK
- Wendy Cowell: University of Exeter Medical School, Exeter, Devon, EX2 5DW, UK
- Jonathan Davies: University of Exeter Medical School, Exeter, Devon, EX2 5DW, UK
- Dori Floris: Department of Cognitive Neuroscience, Donders Institute for Brain Cognition and Behaviour, Radboud University Medical Centre, 6525EN Nijmegen, The Netherlands; Methods of Plasticity Research, Department of Psychology, University of Zurich, Zurich, Switzerland
- Alice Franklin: University of Exeter Medical School, Exeter, Devon, EX2 5DW, UK

- Lidia Gabis: Tel Aviv University, Wolfson Hospital and Maccabi healthcare, Tel Aviv, Israel
- Daniel Geschwind: Program in Neurobehavioral Genetics, Center for Autism Research and Treatment, Semel Institute, David Geffen School of Medicine, University of California, Los Angeles, Los Angeles, CA 90095, USA; Department of Neurology, David Geffen School of Medicine, University of California, Los Angeles, 695 Charles E. Young Drive South, Los Angeles, CA 90095, USA; Department of Psychiatry, Semel Institute, David Geffen School of Medicine, University of California, Los Angeles, 695 Charles E. Young Drive South, Los Angeles, CA 90095, USA; Department of Human Genetics, David Geffen School of Medicine, University of California, Los Angeles, CA 90095, USA
- David M. Greenberg: Interdisciplinary Department of Social Sciences, Bar-Ilan University, Ramat Gan, Israel; Department of Music, Bar-Ilan University, Ramat Gan, Israel; Autism Research Centre, Department of Psychiatry, University of Cambridge, Cambridge, UK
- Yuanjun Gu: Department of Psychiatry, Autism Research Centre, University of Cambridge, Cambridge, Cambridgeshire, CB2 8AH, UK
- Alexandra Havdahl: Center for Genetic Epidemiology and Mental Health, Norwegian Institute of Public Health, Oslo, Norway; Nic Waals Institute, Lovisenberg Diakonale Hospital, Oslo, Norway; PROMENTA Research Center, Department of Psychology, University of Oslo, Oslo, Norway; MRC Integrative Epidemiology Unit, University of Bristol, Bristol, UK
- Alexander Heazell: Maternal and Fetal Health Research Centre, School of Medical Sciences, University of Manchester, Manchester, UK; Saint Mary's Hospital, Manchester University NHS Foundation Trust, Manchester, UK
- Rosemary Holt: Department of Psychiatry, Autism Research Centre, University of Cambridge, Cambridge, Cambridgeshire, CB2 8AH, UK
- Matthew Hurles: Wellcome Sanger Institute, Hinxton, Cambridgeshire, CB10 1SA, UK
- Yumnah Khan: Department of Psychiatry, Autism Research Centre, University of Cambridge, Cambridge, Cambridgeshire, CB2 8AH, UK
- Meng-Chuan Lai: Department of Psychiatry, Autism Research Centre, University of Cambridge, Cambridge, Cambridgeshire, CB2 8AH, UK; The Margaret and Wallace McCain Centre for Child, Youth & Family Mental Health and Azrieli Adult Neurodevelopmental Centre, Campbell Family Mental Health Research Institute, Centre for Addiction and Mental Health, Toronto, Canada; Department of Psychiatry and Autism Research Unit, The Hospital for Sick Children, Toronto, Canada; Department of Psychiatry, Temerty Faculty of Medicine, University of Toronto, Toronto, Canada; Department of Psychiatry, National Taiwan University Hospital and College of Medicine, Taipei, Taiwan

- Madeline Lancaster: MRC Laboratory for Molecular Biology, University of Cambridge, Cambridge, UK
- Michael Lombardo: Laboratory for Autism and Neurodevelopmental Disorders, Center for Neuroscience and Cognitive Systems, Istituto Italiano di Tecnologia, Rovereto, Italy
- Hilary Martin: Wellcome Sanger Institute, Hinxton, Cambridgeshire, CB10 1SA, UK
- Jose Gonzalez Martinez: MRC Laboratory for Molecular Biology, University of Cambridge, Cambridge, UK
- Jonathan Mill: University of Exeter Medical School, Exeter, Devon, EX2 5DW, UK
- Mahmoud Koko Musa: Wellcome Sanger Institute, Hinxton, Cambridgeshire, CB10 1SA, UK
- Kathy Niakan: Cambridge Reproduction, University of Cambridge, Cambridge, UK; The Centre for Trophoblast Research, Department of Physiology, Development and Neuroscience, University of Cambridge, Cambridge, UK; Human Embryo and Stem Cell Laboratory, The Francis Crick Institute, London, UK; Wellcome Trust-Medical Research Council Stem Cell Institute, University of Cambridge, Jeffrey Cheah Biomedical Centre, Cambridge, UK; Epigenetics Programme, Babraham Institute, Cambridge, UK
- Adam Pavlinek: Department of Basic and Clinical Neuroscience, Institute of Psychiatry, Psychology and Neuroscience, King's College London, London, UK; MRC Centre for Neurodevelopmental Disorders, King's College London, London, UK
- Lucia Dutan Polit: Department of Basic and Clinical Neuroscience, Institute of Psychiatry Psychology and Neuroscience, King's College London, London, UK; MRC Centre for Neurodevelopmental Disorders, King's College London, London, UK
- Marcin Radecki: Department of Psychiatry, Autism Research Centre, University of Cambridge, Cambridge, Cambridgeshire, CB2 8AH, UK; Social and Affective Neuroscience Group, IMT School for Advanced Studies Lucca, Lucca 55100, Italy
- David Rowitch: Wellcome-MRC Cambridge Stem Cell Institute, University of Cambridge, Cambridge CB2 0AW, UK; Department of Paediatrics, University of Cambridge, Cambridge, UK
- Laura Sichlinger: Department of Basic and Clinical Neuroscience, The Maurice Wohl Clinical Neuroscience Institute, Institute of Psychiatry, Psychology & Neuroscience, King's College London, London, UK; MRC Centre for Neurodevelopmental Disorders, Institute of Psychiatry, Psychology & Neuroscience, King's College London, London, UK
- Deepak Srivastava: Department of Basic and Clinical Neuroscience, Institute of Psychiatry Psychology and Neuroscience, King's College London, London, UK; MRC Centre for Neurodevelopmental Disorders, King's College London, London, UK
- Alexandros Tsompanidis: Department of Psychiatry, Autism Research Centre, University of Cambridge, Cambridge, Cambridgeshire, CB2 8AH, UK

- Florina Uzefovsky: Ben-Gurion University of the Negev, Beer-Sheva, 84105, Israel
- Varun Warrier: Department of Psychiatry, Autism Research Centre, University of Cambridge, Cambridge, Cambridgeshire, CB2 8AH, UK
- Elizabeth Weir: Department of Psychiatry, Autism Research Centre, University of Cambridge, Cambridge, Cambridgeshire, CB2 8AH, UK
- Xinhe Zhang: Department of Psychiatry, Autism Research Centre, University of Cambridge, Cambridge, Cambridgeshire, CB2 8AH, UK

Supplemental Figures

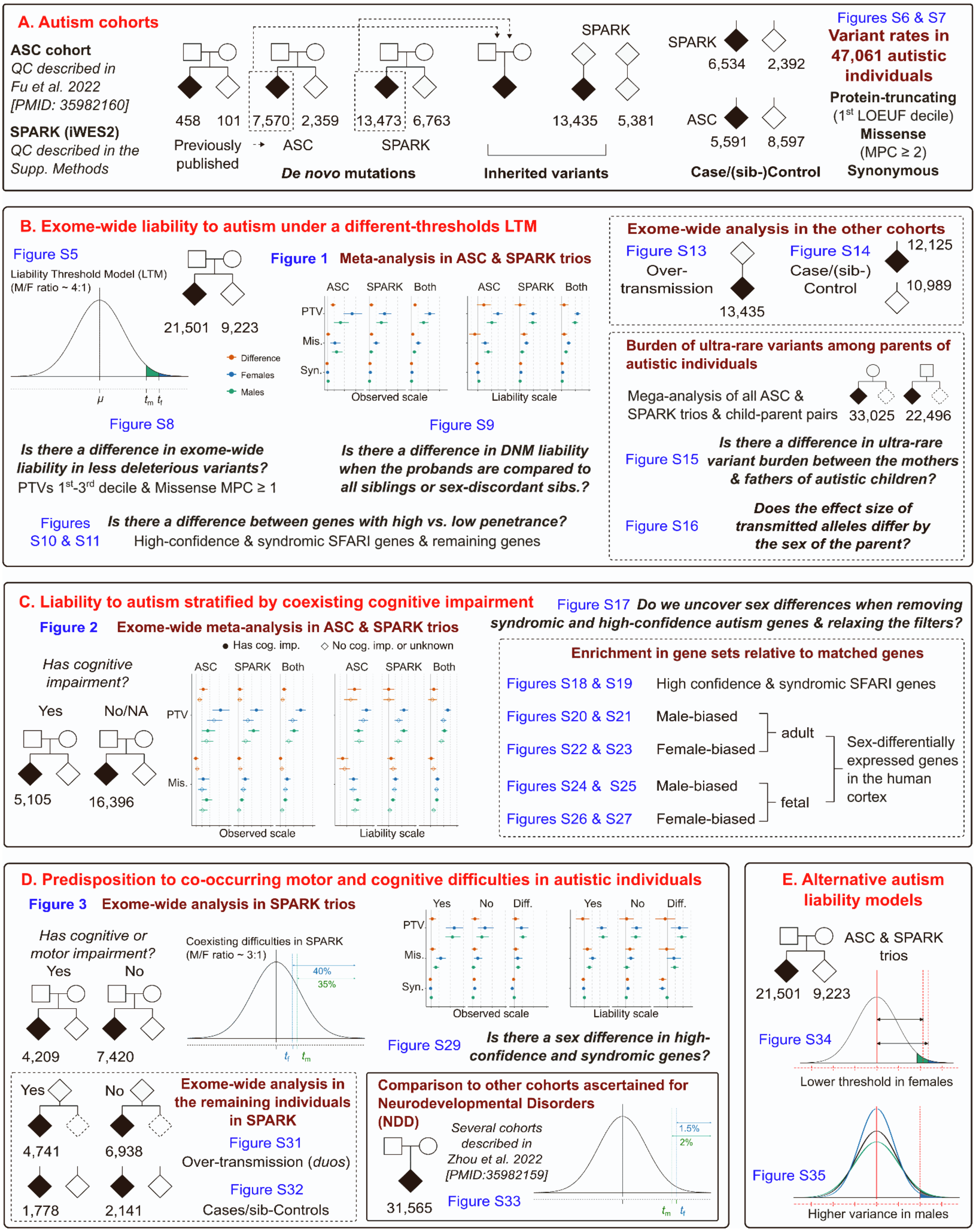

Figure S1: Flow chart summarizing the analyses in this study.

The main analyses were performed (separately) for *de novo* and rare inherited variants ascertained in the family-based cohort. Additional exome-wide analyses in the remaining individuals with one/no sequenced parent(s) are shown where applicable. For each analysis or research question, the flowchart indicates the relevant main and supplemental figures.

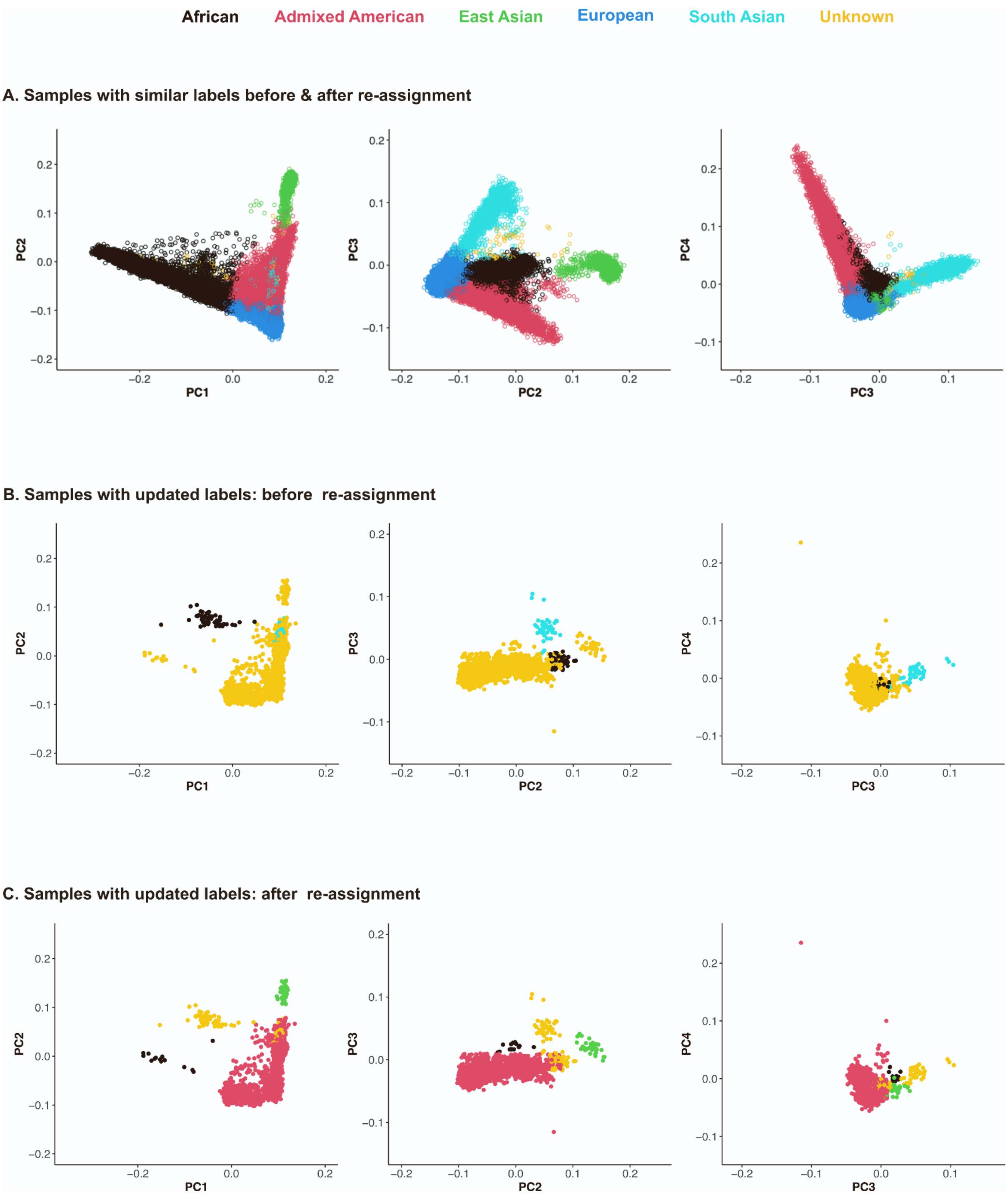

Figure S2: Population labels of SPARK samples.

Principal component analysis (PCA) was used to check the validity of the pre-assigned population labels from SPARK against the top principal components projected on the 1000 Genomes space (reference samples not shown). Most samples clustered as expected (**A**). Samples with unknown labels and those not clustering with their respective pre-assigned groups (**B**) were re-classified (**C**). See section 1.3. 'Ancestry Inference' of the Supplemental Methods and Tables S3 & S4 for details.

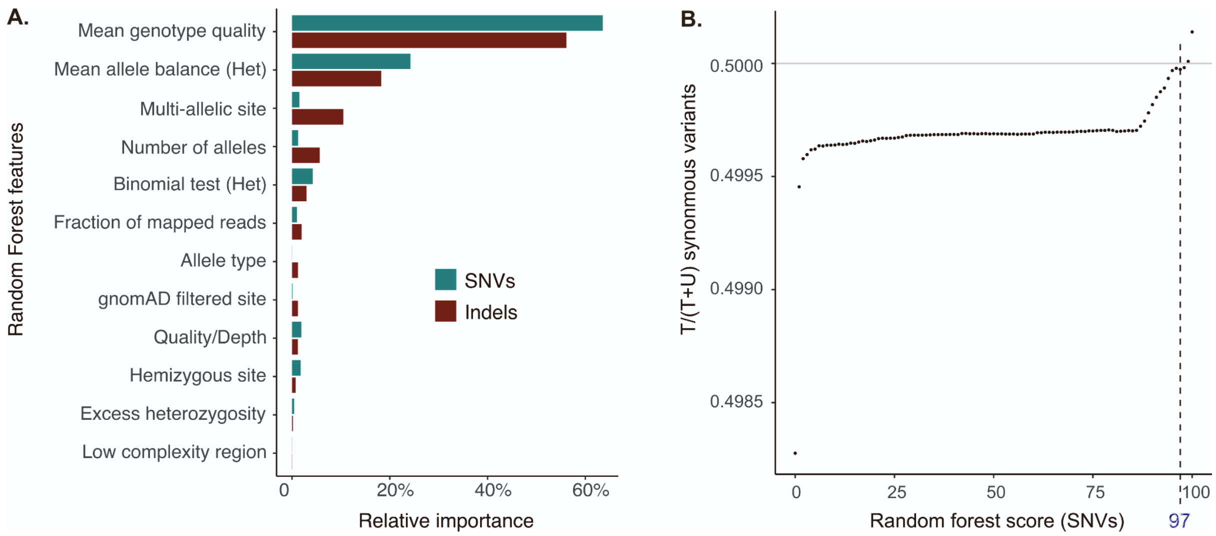

Figure S3: Assigning variant quality scores using Random Forest.

A Random Forest was trained using 12 features (**A**) to assign a variant-level quality score between 0 (lowest quality) and 100 (highest quality). The average genotype quality per variant (all genotypes) and mean allele balance per variant (heterozygous genotypes) had the highest relative importance (y-axis). The fraction of the transmitted (T) alleles from the total transmitted & untransmitted (T+U) parental synonymous variants achieved after filtering with increasing stringency (higher Random Forest scores) is shown in **B**. Single Nucleotide Variants (SNVs) with scores > 97 (dotted line) and Insertions-Deletions (Indels) with scores > 92 were retained. See section 1.6. (Variant QC) for further details.

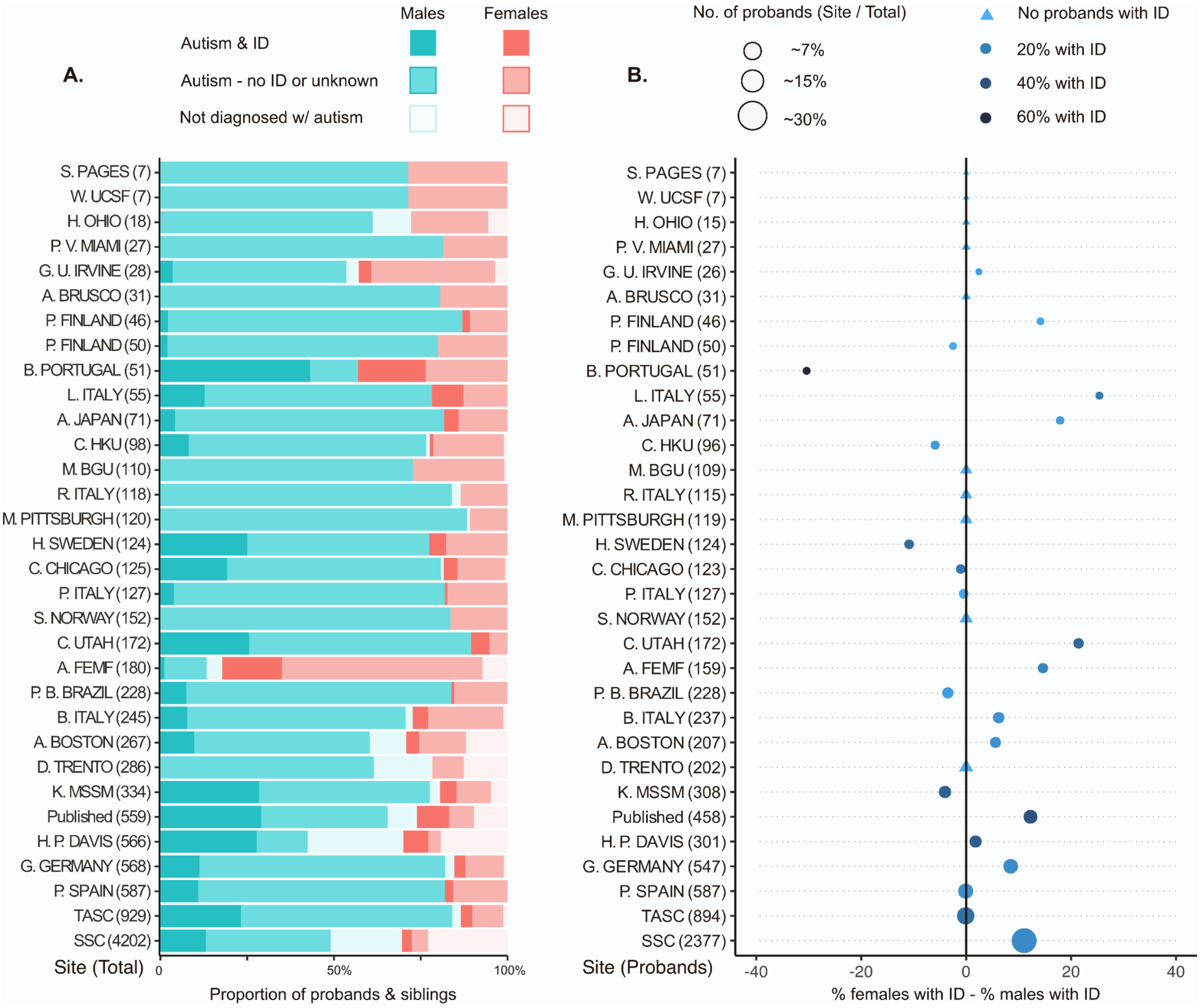

Figure S4: The proportion of autistic individuals with intellectual disability across the ASC cohorts.

A, The sample size of each sub-cohort in the ASC trios dataset and the proportion of autistic probands with intellectual disability (ID), without intellectual disability (or unknown), and siblings not diagnosed with autism. The cohorts are sorted by their size (total of probands and siblings) along the y axis. See Table S2 for these counts. **B**, The difference in the proportion of females with ID and males with ID within each sub-cohort. The 'dot' size indicates the percentage of individuals with ID in the cohort (both males and females), whereas its position along the x-axis indicates sex bias; if placed in the middle, then the % of autistic individuals with ID is equal amongst males and females, to the right means more females with ID, to the left more males with ID.

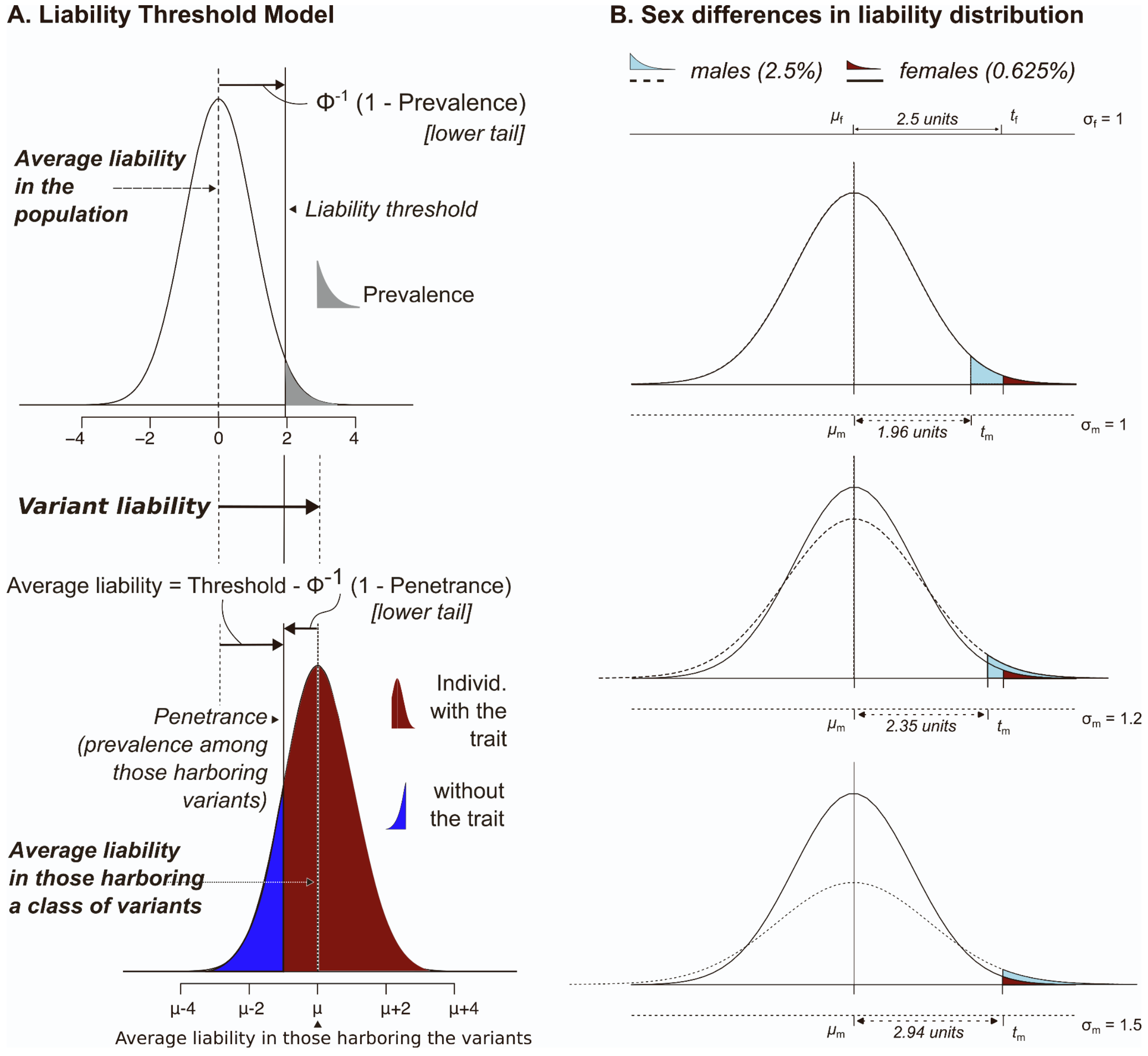

Figure S5: Estimating rare variant liability from population prevalence and variant rates.

A, The Liability Threshold Model postulates that risk factors act additively and underlie a normally distributed liability distribution in the general population (top), where the threshold determines the trait prevalence. When examining a group of rare variants (bottom), the average liability amongst all individuals harboring these variants will reflect the average effect size of the variant class. If the prevalence of the trait among individuals harboring a class of rare variants is known (‘penetrance’), the two distributions can be juxtaposed (or overlaid), leveraging the threshold as a reference point, to get an estimate of the distance between their means. See the ‘Variant liability’ section for a detailed description. **B**, Sex differences in population prevalence between two groups (e.g. males and females) can arise from differences in the threshold or the variance. The three models show how different properties of the liability distribution in females (solid line) and males (dotted line) can underlie the same male-to-female ratio. Assuming the liability distribution in females has a variance of one and a threshold that is +2.5 units from the mean (prevalence = 0.625%), a prevalence of 2.5% in males (4:1 ratio) can result from males having the same variance but a lower threshold than females (top), higher variance but the same threshold (bottom) or a combination of the two (middle).

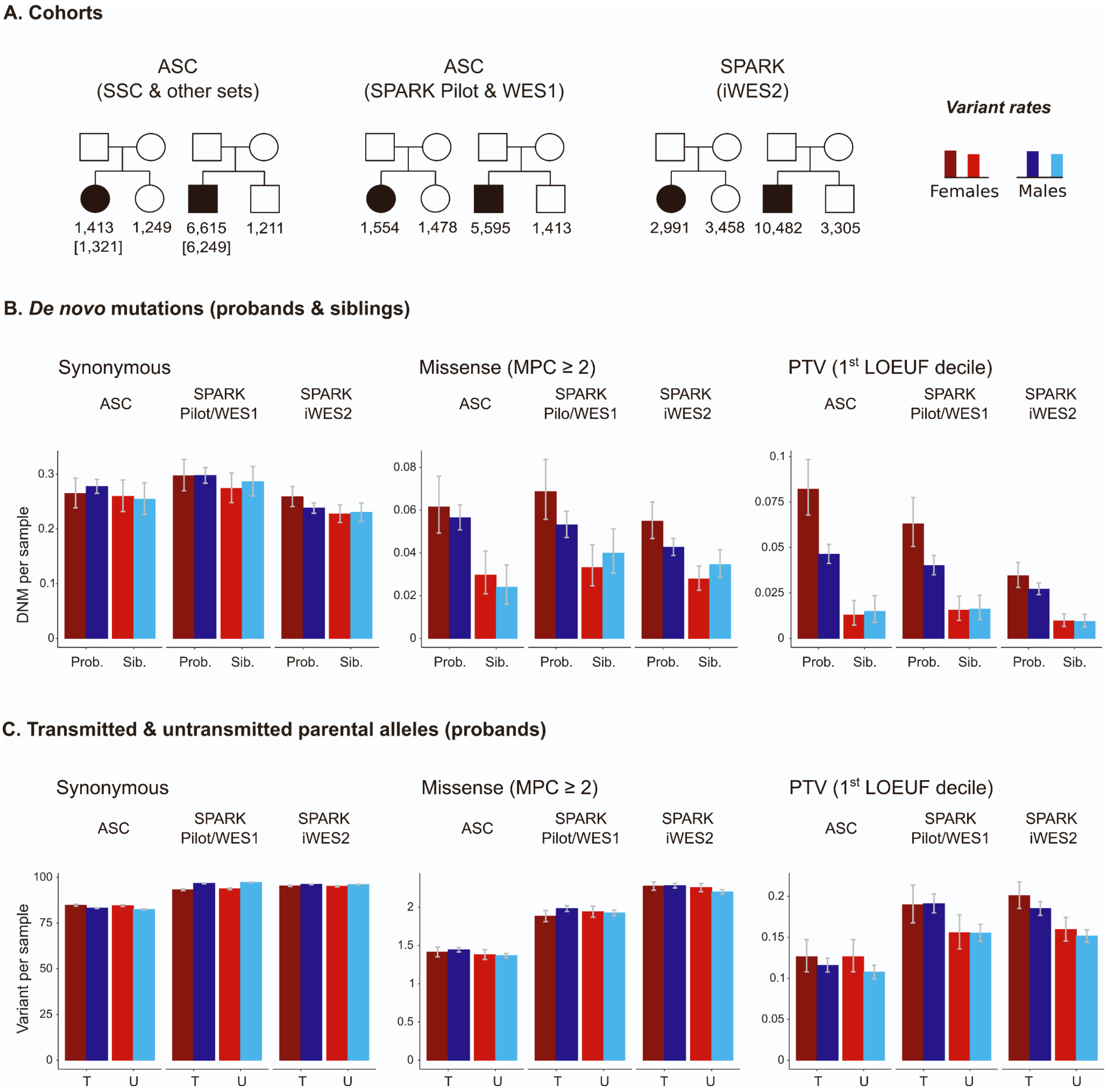

Figure S6: Rare *de novo* and inherited variant rates in the ASC & SPARK trio-sequenced cohorts.

A, The pedigrees show the sample size used for counting inherited variants & calculating *de novo* rates in the Autism Sequencing Consortium (ASC) cohort and the Simons Foundation Powering Autism Research for Knowledge (SPARK) cohort (sample size for inherited variants given between brackets as some individuals in the ASC cohort did not have information on inherited alleles). **B**, *De novo* mutation rates in the probands (prob.) and siblings (sib.). **C**, Average counts of transmitted (T) and untransmitted (U) parental alleles at rare variants (MAF < 0.1%). SPARK Pilot/WES1 is shown for comparison *versus* SPARK iWES2, as it was processed using the same pipeline used for the remaining ASC samples. The error bars indicate the 95% confidence intervals of the variant rates. See section 1 of the Supplemental Results for details.

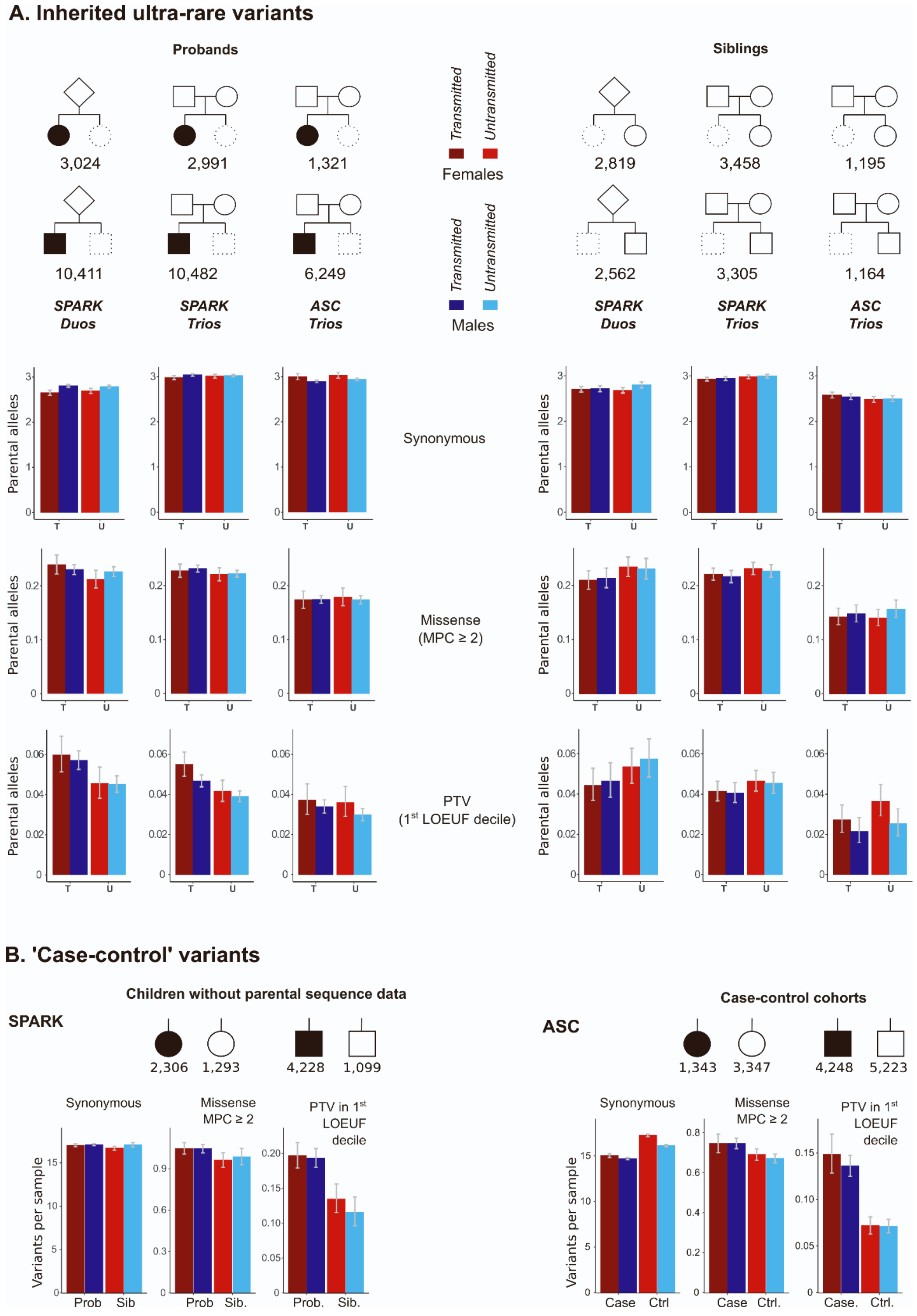

Figure S7: Ultra-rare inherited and case-control variant rates in SPARK and ASC cohorts.

A, The transmission rates of ultra-rare parental alleles (seen in one parent and not in gnomAD) in probands and siblings with exome data from one or both parents in the Simons Foundation Powering Autism Research for Knowledge (SPARK) cohort (duos & trios) and the Autism Sequencing Consortium (ASC) cohort (trios). **B**, Ultra-rare variant rates (allele frequency < 0.005%) in the remaining children in SPARK, who did not have parental sequence data, and the ASC case-control cohorts. The pedigrees show the sample size used for counting parental alleles (**A**) or case-control variants (**B**). The bar plots show the sex-stratified ultra-rare variant rates; The error bars indicate the 95% confidence intervals of the variant rates. See section 1 of the Supplemental Results for details.

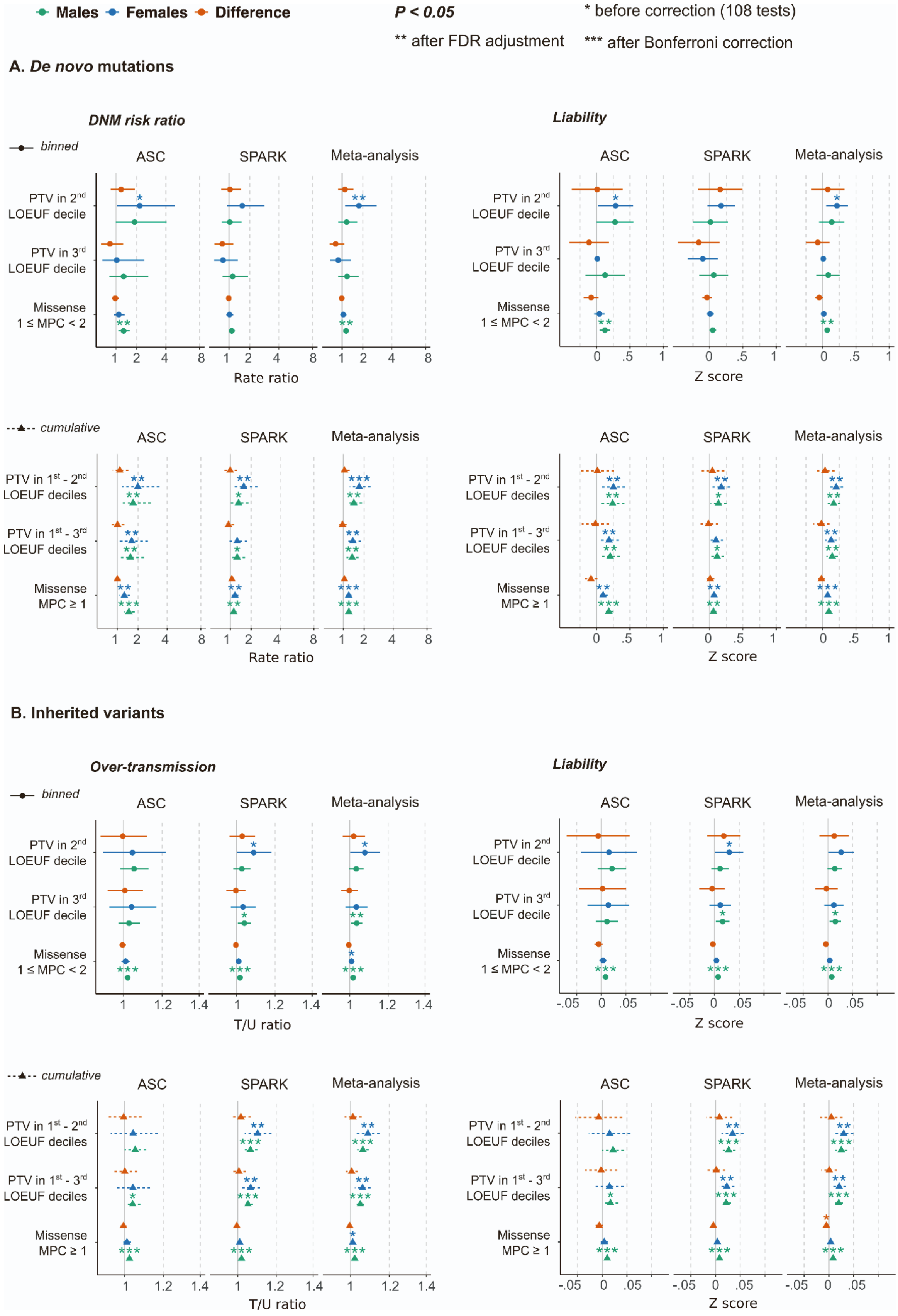

Figure S8: Sensitivity analysis for damaging *de novo* and rare inherited variants in LoF-intolerant genes.

A, Sex-stratified *de novo* mutation rate ratios (left) and liability (right) in trio-sequenced individuals in the Simons Foundation Powering Autism Research for Knowledge study (SPARK) and the Autism

Sequencing Consortium (ASC) cohorts. **B**, Over-transmission and liability of inherited variants in these cohorts. Whereas the primary analysis in Figure 1 focused on protein-truncating variants in highly-LoF-constrained genes, these analyses examined protein-truncating variants in less constrained genes and damaging missense variants with lower MPC scores. Error bars show 95% confidence intervals.

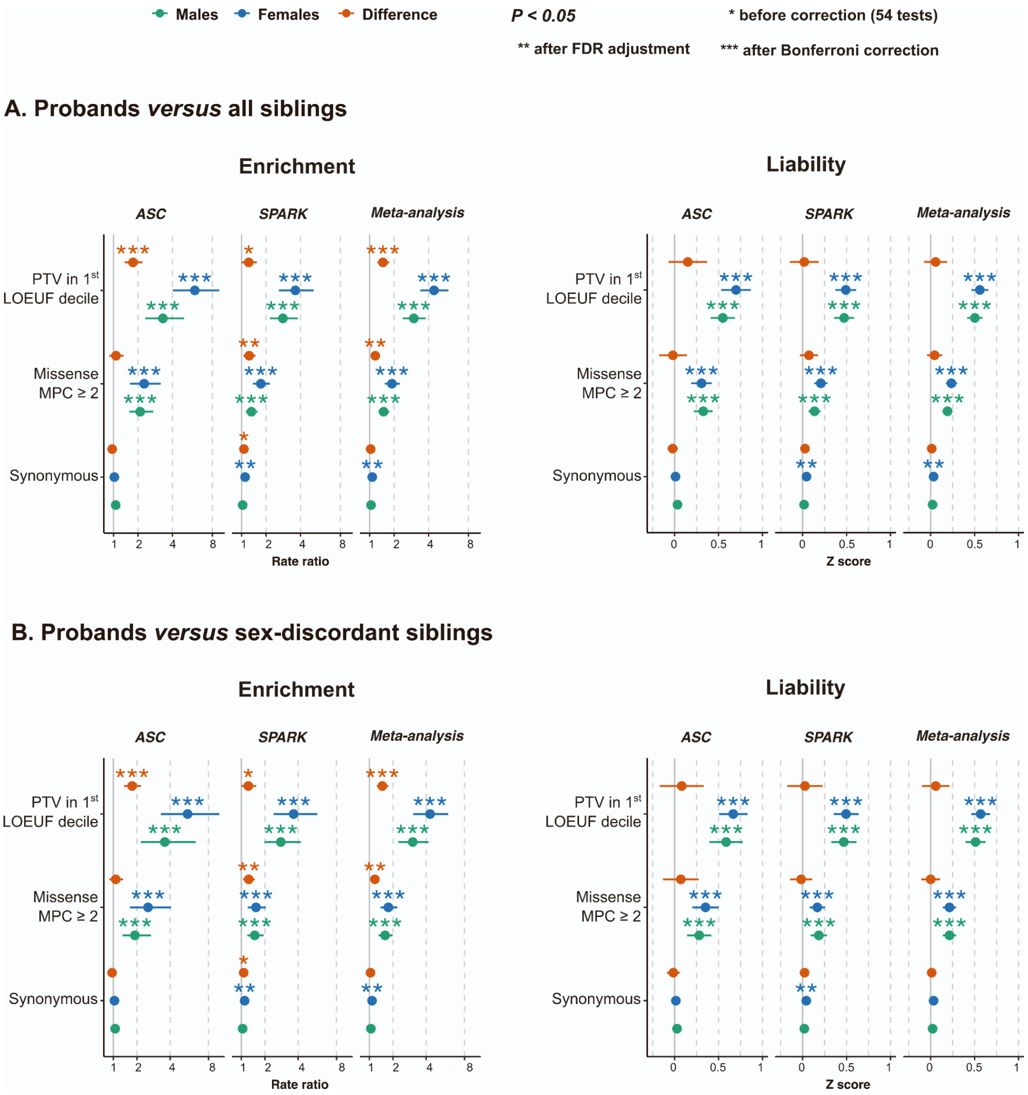

Figure S9: DNM enrichment in autistic probands *versus* all siblings and sex-discordant siblings.

Sex-stratified *de novo* mutation enrichment (left) and liability (right) were measured by comparing the DNM rates in female or male probands to the DNM rates seen in all siblings (males and females) in **A** or sex-discordant siblings (female pronands vs. male siblings, male probands vs. female siblings) in **B**, which is comparable to the analysis presented in Figure 1B (*versus* sex-matched siblings). Error bars show 95% confidence intervals. For sex differences, a rate ratio > 1 indicates that females show a higher enrichment; a Z score > 0 indicates that females show a higher effect size on the liability scale. The sex differences on the liability scale reflect the Z score difference between females and males (*versus* all siblings in **A** and *versus* sex-discordant siblings in **B**), similar to what is shown in Figure 1B (*versus* sex-alike siblings). Note that the sex differences in DNM rate ratios are obtained through direct comparisons of DNM rates of female and male probands and are therefore independent of the set of siblings used (i.e. identical values in A, B and Figure 1B). However, the differences in liability are Z-score differences, and thus represent different tests in A, B and Figure 1B.

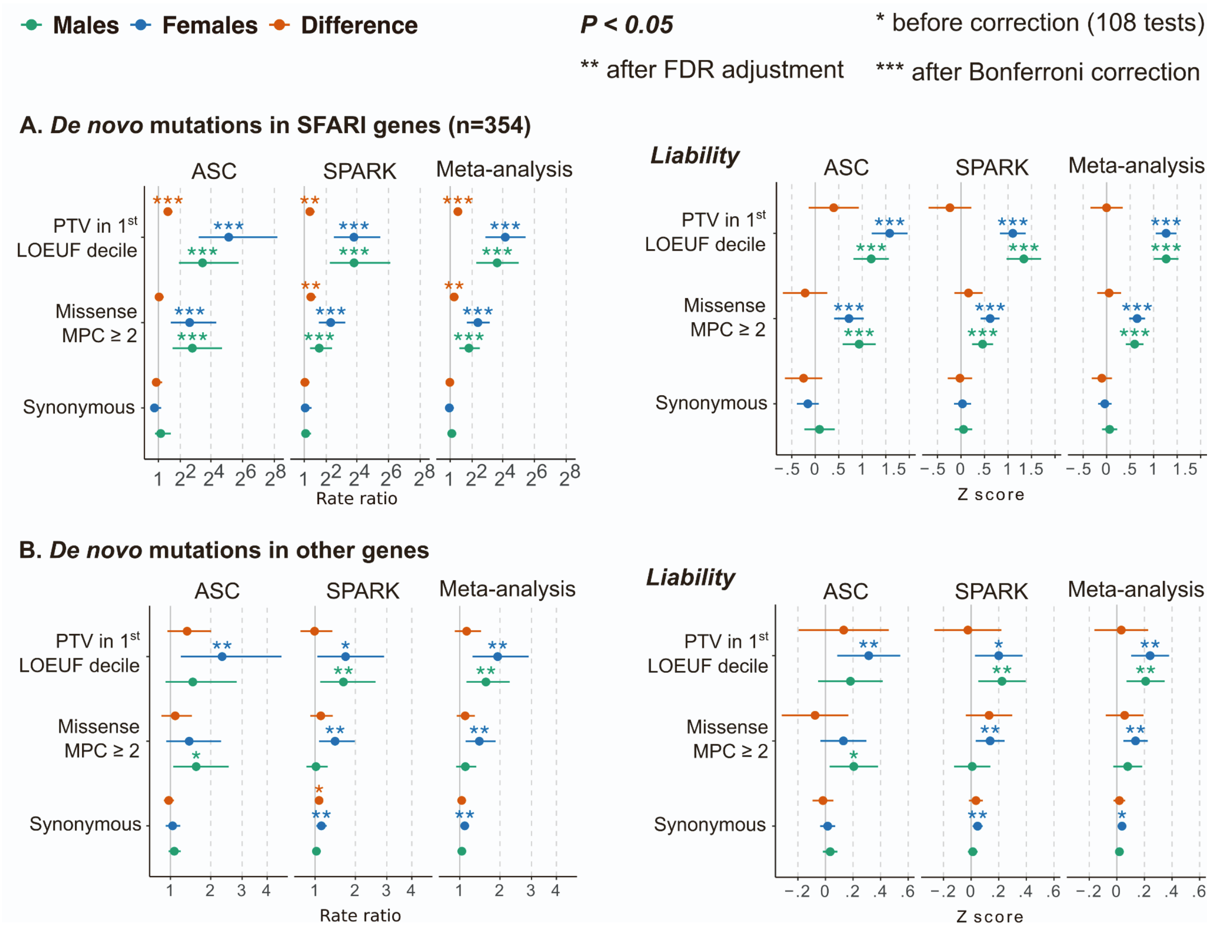

Figure S10: Enrichment of *de novo* mutations in 354 high-confidence and syndromic SFARI autism predisposition genes *versus* all other genes.

Sex-stratified *de novo* mutation rate ratios (left) and liability (right) (see Methods) in SFARI genes (**A**) and all other genes (**B**). For sex differences, a rate ratio > 1 indicates that females show a higher enrichment; a Z score > 0 indicates that females show a higher effect size on the liability scale. Error bars show 95% confidence intervals. Similar to Figure 1B; See Figure S11 for inherited variants.

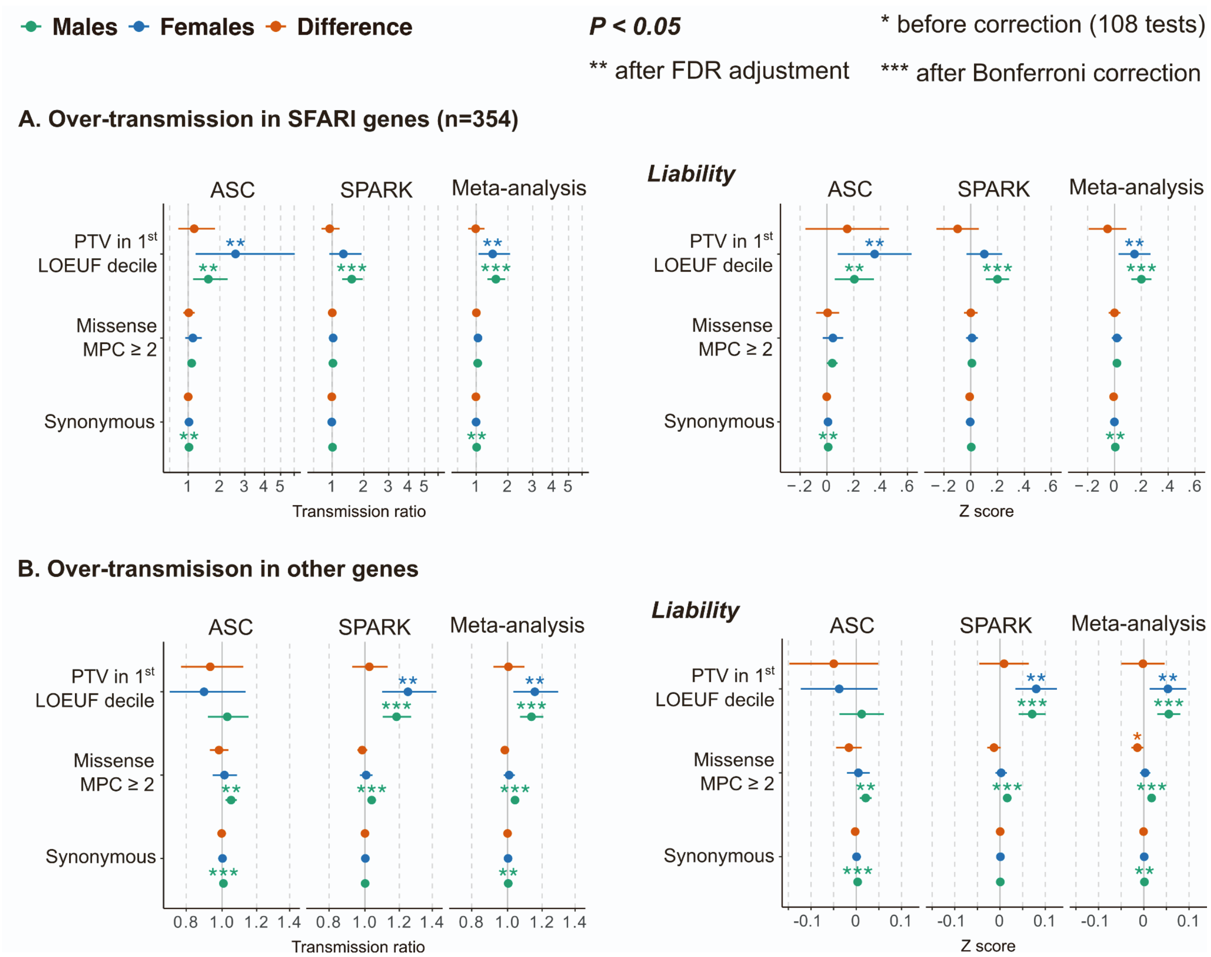

Figure S11: Over-transmission of rare inherited variants in 354 high-confidence and syndromic SFARI autism predisposition genes *versus* all other genes.

Over-transmission (left) and liability (right) of rare inherited variants in SFARI high-confidence genes (**A**) *versus* all other genes (**B**) (see Methods). Error bars show 95% confidence intervals. Note that the scale on the x-axis is different in A and B. Similar to Figure 1C. See Figure S10 for *de novo* mutations.

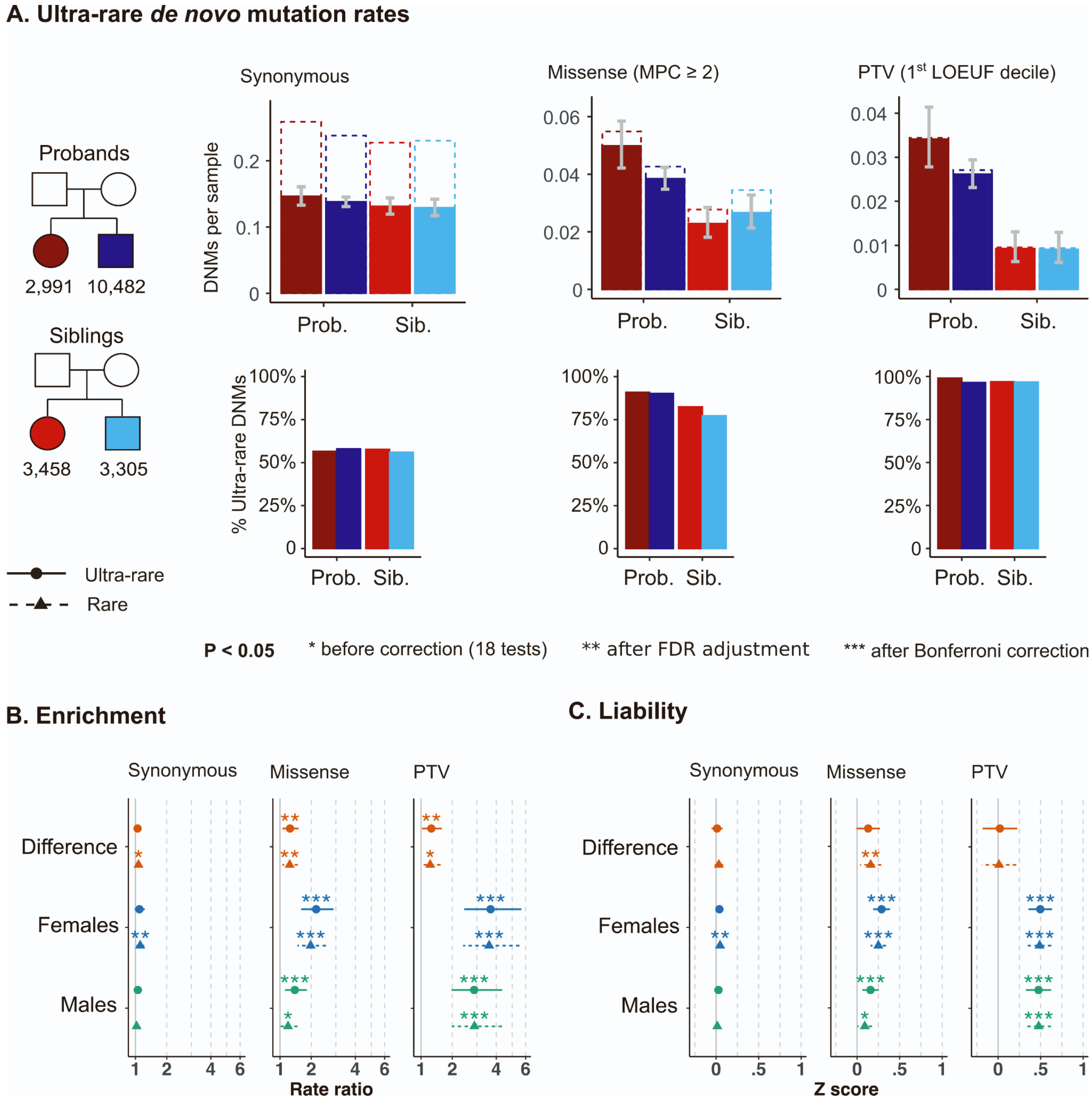

Figure S12: Enrichment of ultra-rare *de novo* mutations in SPARK trio-sequenced individuals.

A, Rates of ultra-rare *de novo* mutations (DNM) (filled bars) compared to all rare DNMs (dotted lines). Most damaging protein-truncating and missense DNMs were ultra-rare compared to about half of the synonymous DNMs. **B,** Ultra-rare DNM rate ratios between probands and siblings, compared to rare DNMs. **C**, The liability of ultra-rare and rare DNMs. There was no significant difference in the average liability of ultra-rare synonymous DNM, in contrast to the difference seen in rare DNMs. Error bars show 95% confidence intervals.

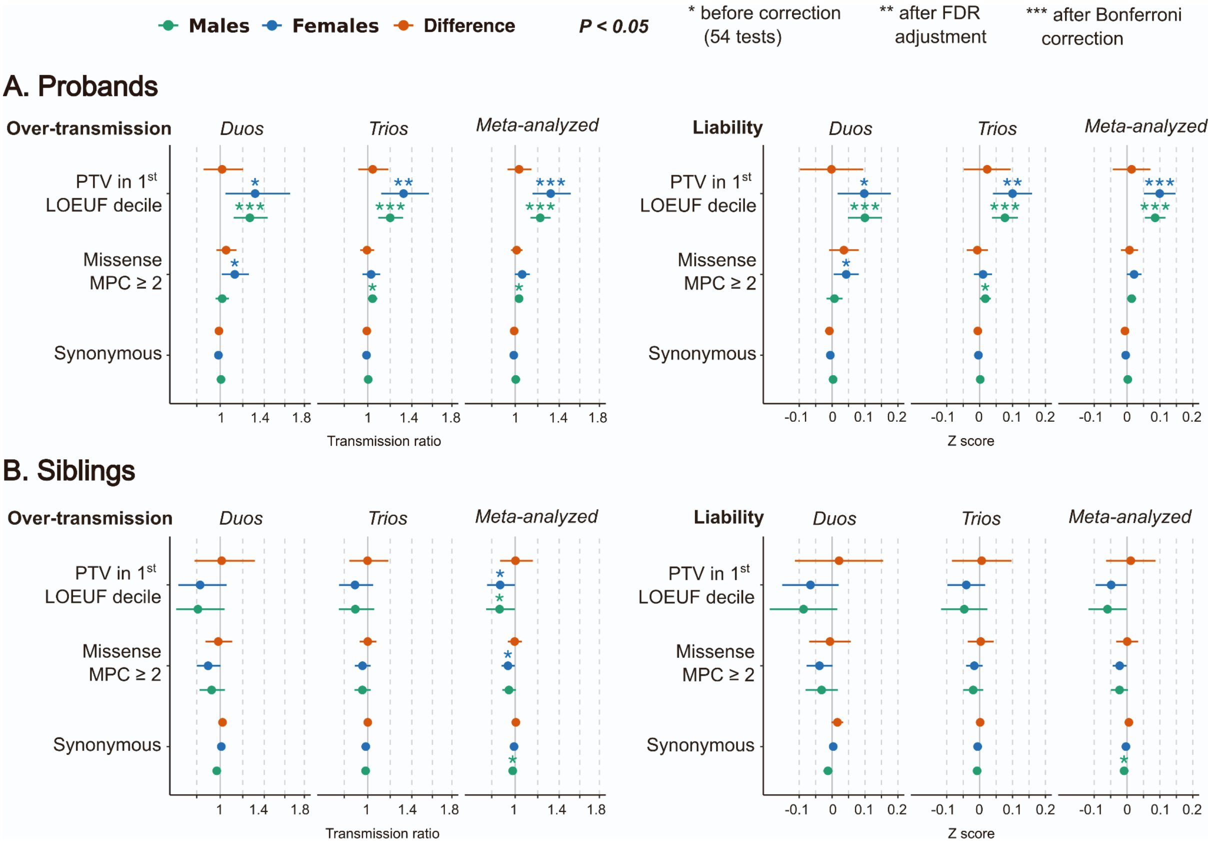

Figure S13: Over-transmission of ultra-rare variants in SPARK.

Over-transmission of ultra-rare variants (i.e. seen in one parent in the dataset, absent from gnomAD) was studied in child-parent pairs with one (duos) or two sequenced parents (trios), then meta-analyzed. This analysis was performed in probands (**A**) and siblings (**B**). Error bars show 95% confidence intervals. Related to over-transmission analysis of rare (MAF < 0.1%) variants in trios (Figure 1C). See Figure S7A for the sample size of these cohorts.

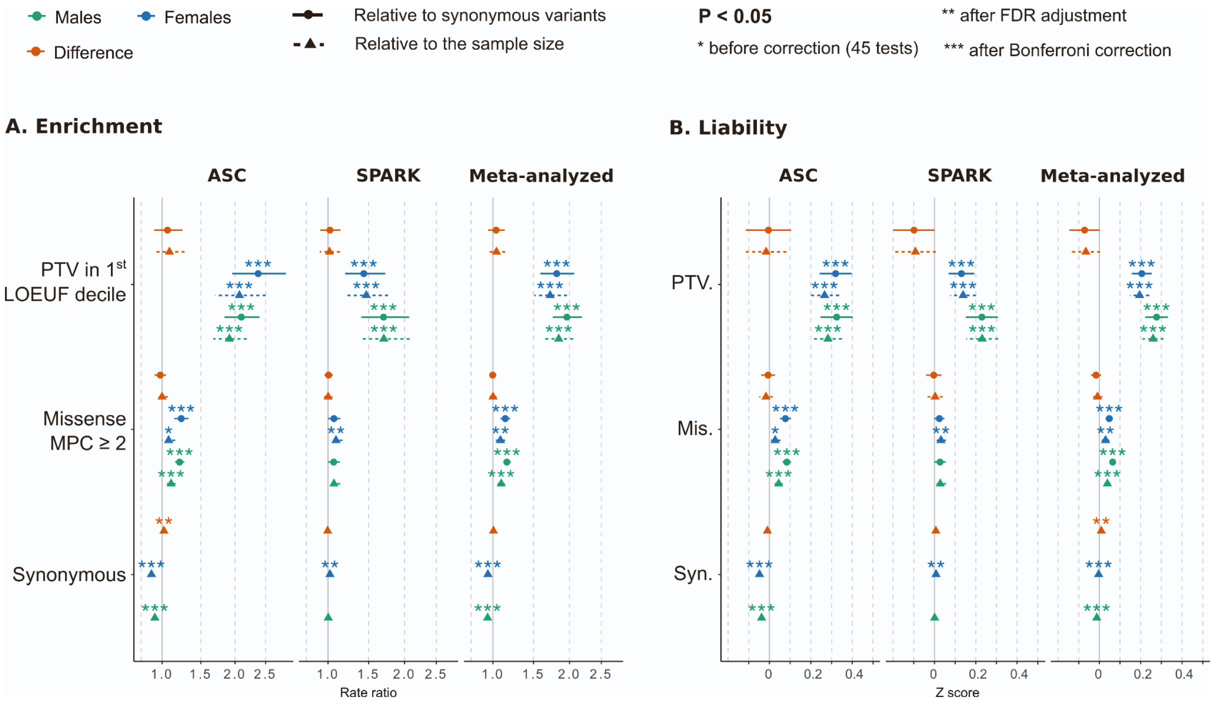

Figure S14: Enrichment of ultra-rare variants the case-control cohorts.

Ultra-rare variant rates in autism cases (ASC) and autistic probands without parental sequence data (SPARK) were compared to autism controls (ASC) or siblings not diagnosed with autism (SPARK). The error bars show the 95% confidence intervals of the effect size on the observed scale (**A**) and liability scale (**B**). The enrichment and liability were assessed relative to the sample size of the case/control cohorts (i.e. assuming that the expected rare variant burden per sample is similar across cohorts). To account for the differences in ultra-rare variant counts arising from the differences in ancestry, damaging missense and protein truncating variants were also compared to the expected rate ratio from synonymous variants (i.e. normalizing the average variant rates using synonymous variant counts). See Figure S7B for the sample sizes and ultra-rare variant rates.

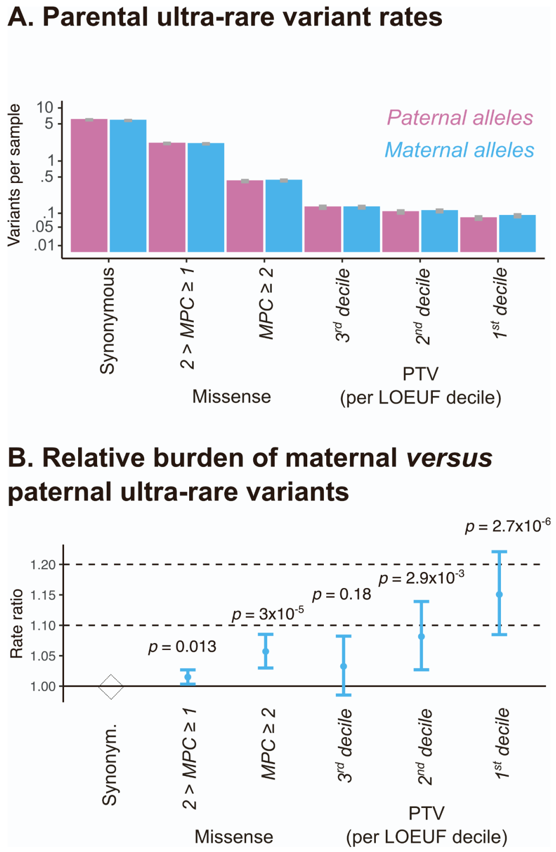

Figure S15: Parental differences in the burden of ultra-rare variants.

The transmission of ultra-rare variants (i.e. variants seen in one parent in the dataset, absent from gnomAD) was evaluated across all child-parent pairs from SPARK and ASC cohorts encompassing 34,478 (unique) autistic children (21,043 trios & 13,435 duos). Each parent-child pair was considered separately, resulting in 55,521 pairs. **A**, Ultra-rare variant rates in the mothers and fathers, pooled across all cohorts. Note the log scale on the y-axis. Error bars show 95% confidence intervals. **B**, A comparison of relative variant rates between mothers and fathers. The rate ratios, their 95% CI (error bars) and *p* values were obtained from binomial tests comparing variant counts in the mothers and fathers, and using the synonymous variant counts in mothers and fathers to derive the expected rate (hence, the rate ratio of synonymous variants is 1).

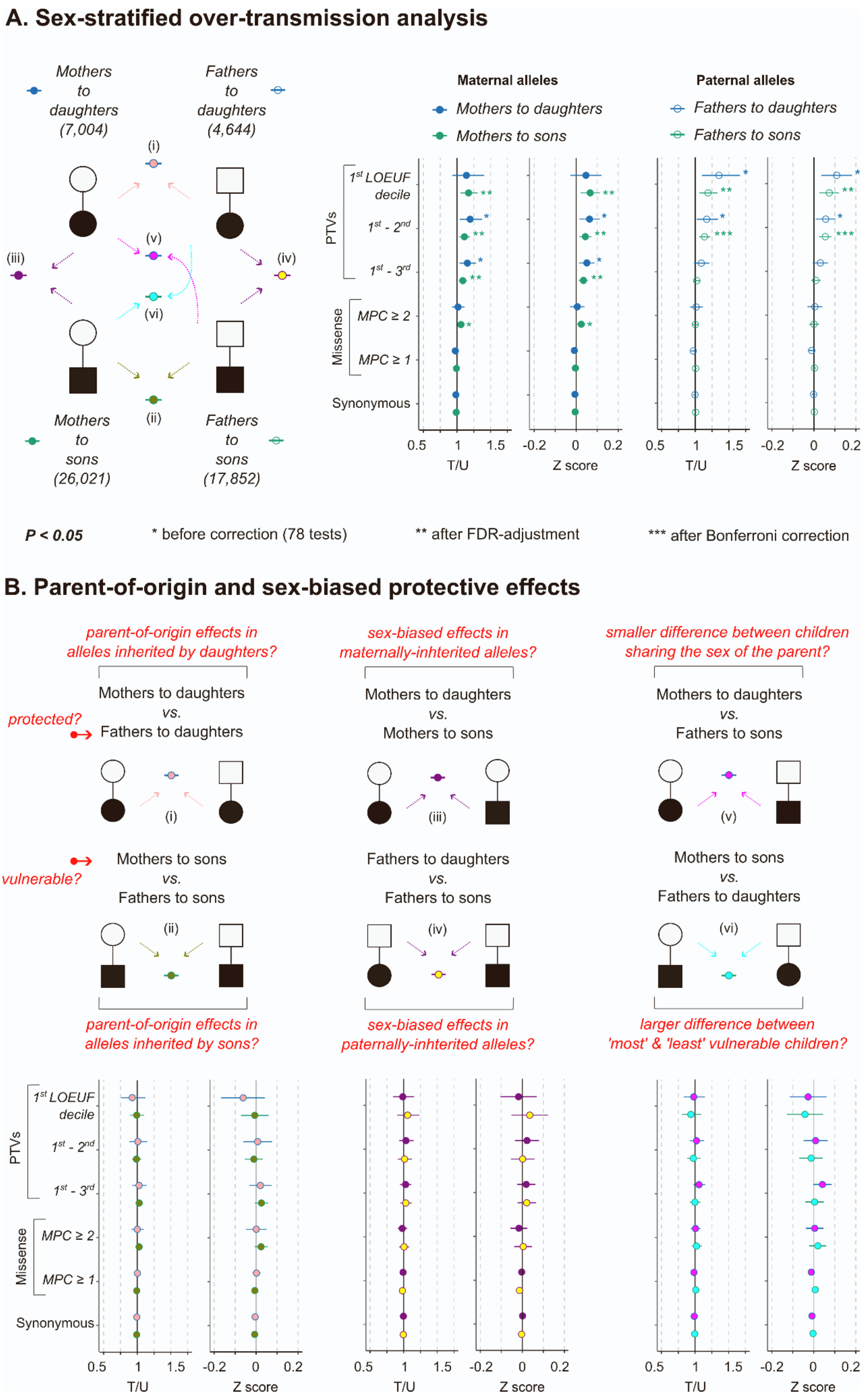

Figure S16: Parent-of-origin and sex-biased protective effects in inherited ultra-rare variants.

The transmission ratios (T/U) and effect sizes on the liability scale (Z score) of maternally-inherited and paternally-inherited alleles were examined separately in sons and daughters (**A**). This mega-analysis was performed across all child-parent pairs from trio- and duo-sequenced children in

ASC and SPARK (the number of child-parent pairs is given in schematic on the left-hand side). In this test, T/U ratio > 1 or Z score > 0 means there is evidence for over-transmission from a comparison of transmitted to untransmitted alleles. The effect sizes (on the observed and liability scales) were used to test for parent-of-origin effects and sex-biased vulnerability to damaging maternal or paternal alleles (**B)**. Six pairwise tests were performed (numbered i-v and color-coded similarly in **A** and **B**). The schematics on the top of each forest plot in **B** indicate the tested alleles and summarizes the question it attempts to answer; the effect size is given for the allele on the left-hand side in the schematic relative to the allele on the right-hand side; e.g., in test 'vi', a T/U ratio > 1 would mean that maternal alleles show more over-transmission to sons (hypothesized to be 'vulnerable') relative to paternal alleles transmitted to daughters (hypothesized to be 'protected'). In all panels, p values were obtained from binomial tests; Error bars show 95% confidence intervals.

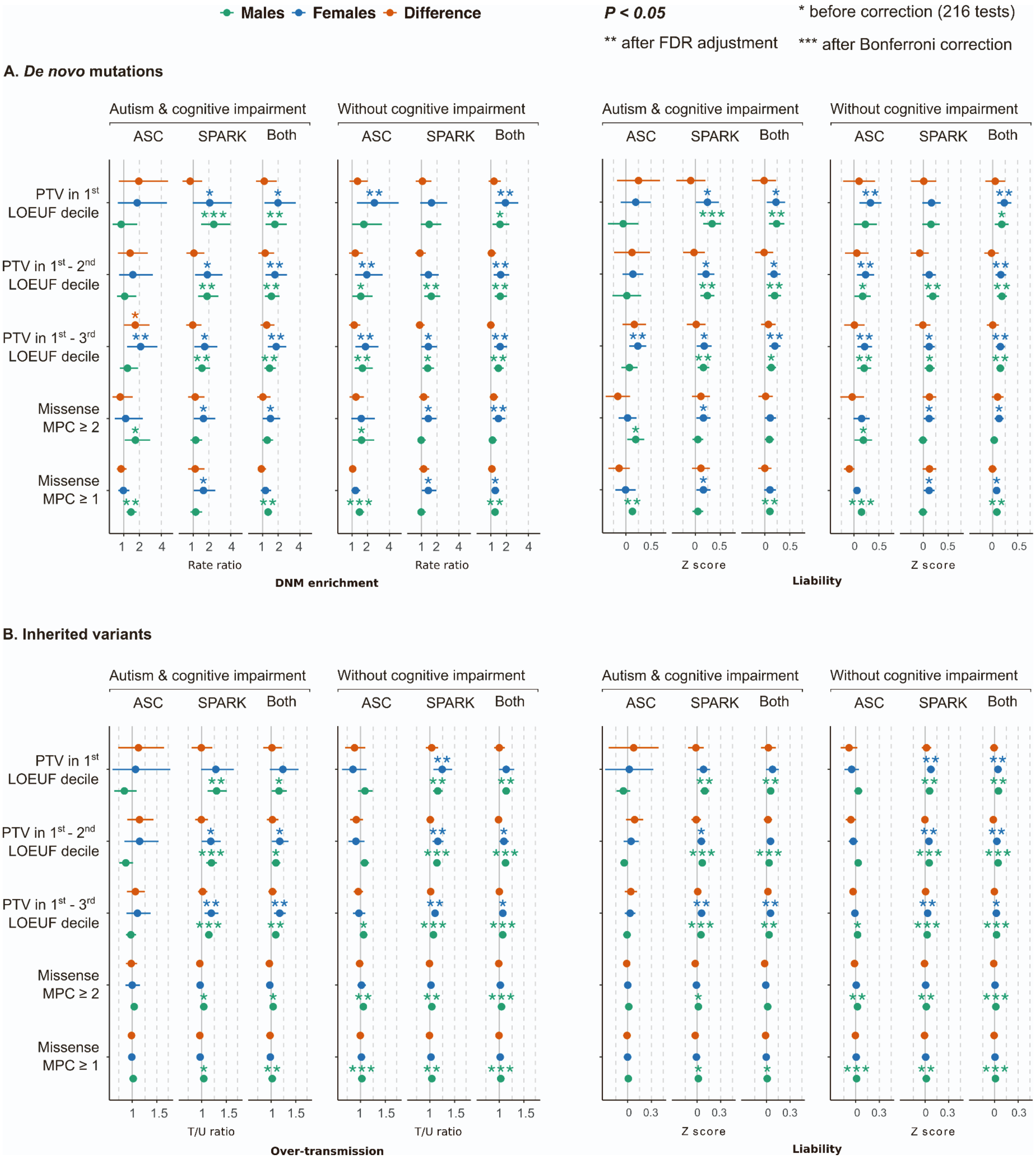

Figure S17: Exome-wide enrichment of *de novo* and rare variants after excluding high-confidence and syndromic autism risk genes amongst individuals with and without cognitive impairment.

A, Sex-stratified *de novo* mutation rate ratios (left) and liability (right) calculated after excluding SFARI genes. **B**, Over-transmission of rare damaging variants (left) and the liability conveyed by inherited alleles (right) in these phenotypic groups. The enrichment and liability was calculated separately in those with or without cognitive impairment (including those with unknown status). See Figure 3 for the sample sizes. For sex differences, a rate ratio > 1 indicates that females show a higher enrichment; a Z score > 0 indicates that females show a higher effect size on the liability scale. Error bars show 95% confidence intervals.

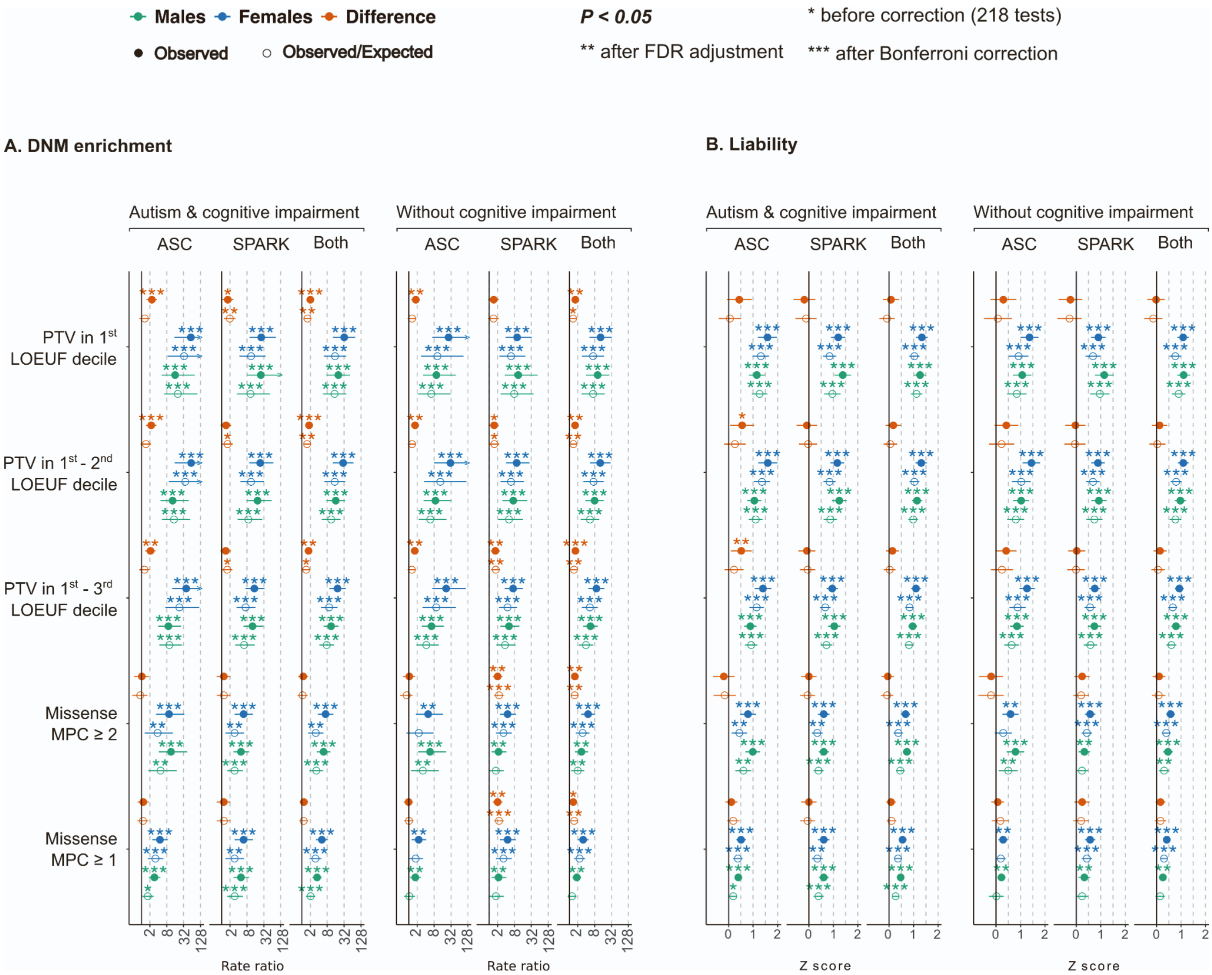

Figure S18: Meta-analysis of the burden of *de novo* mutations in SFARI genes in autistic individuals with and without cognitive impairment in ASC and SPARK.

Sex-stratified *de novo* mutation rate ratios (**A**) and liability (**B**) in SFARI high-confidence and syndromic autism risk genes calculated separately in those with or without cognitive impairment (including those with unknown status). Additional comparisons were performed to compare the observed burden and effect sizes to what is expected for a matched gene set (‘Observed/Expected’). For sex differences, a rate ratio > 1 indicates that females show a higher enrichment; a Z score > 0 indicates that females show a higher effect size on the liability scale. Error bars show 95% confidence intervals.

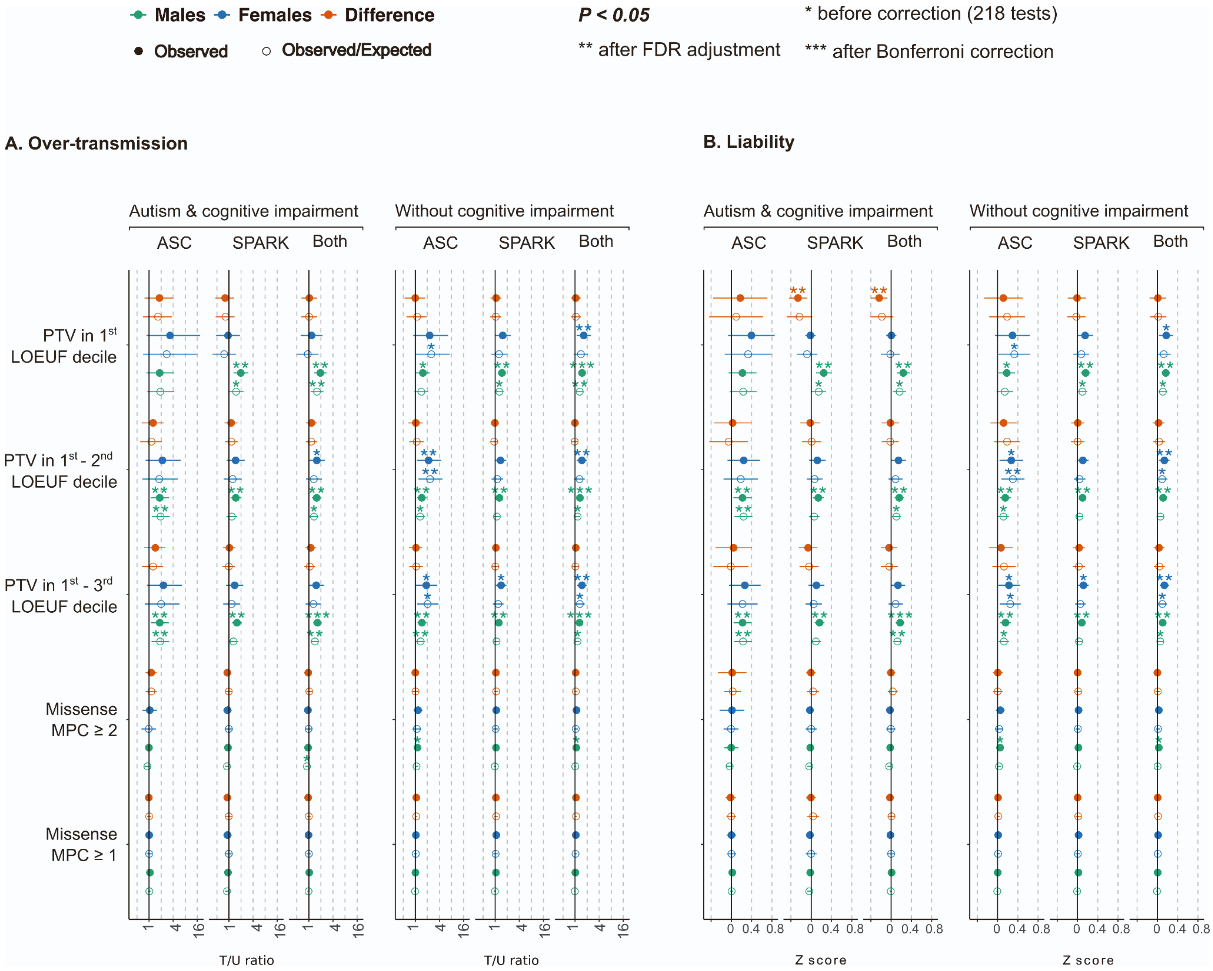

Figure S19: Meta-analysis of the burden of rare inherited variants in SFARI genes in autistic individuals with cognitive impairment in ASC and SPARK.

Over-transmission of rare damaging variants (**A**) in SFARI high-confidence and syndromic autism risk genes and the liability conveyed by inherited alleles (**B**) calculated separately in those with or without cognitive impairment (including those with unknown status). Additional comparisons were performed to compare the observed transmission rates and effect sizes to what is expected for a matched gene set (‘Observed/Expected’). For sex differences, a transmission ratio > 1 indicates that females show a higher over-transmission; a Z score > 0 indicates that females show a higher effect size on the liability scale. Error bars show 95% confidence intervals.

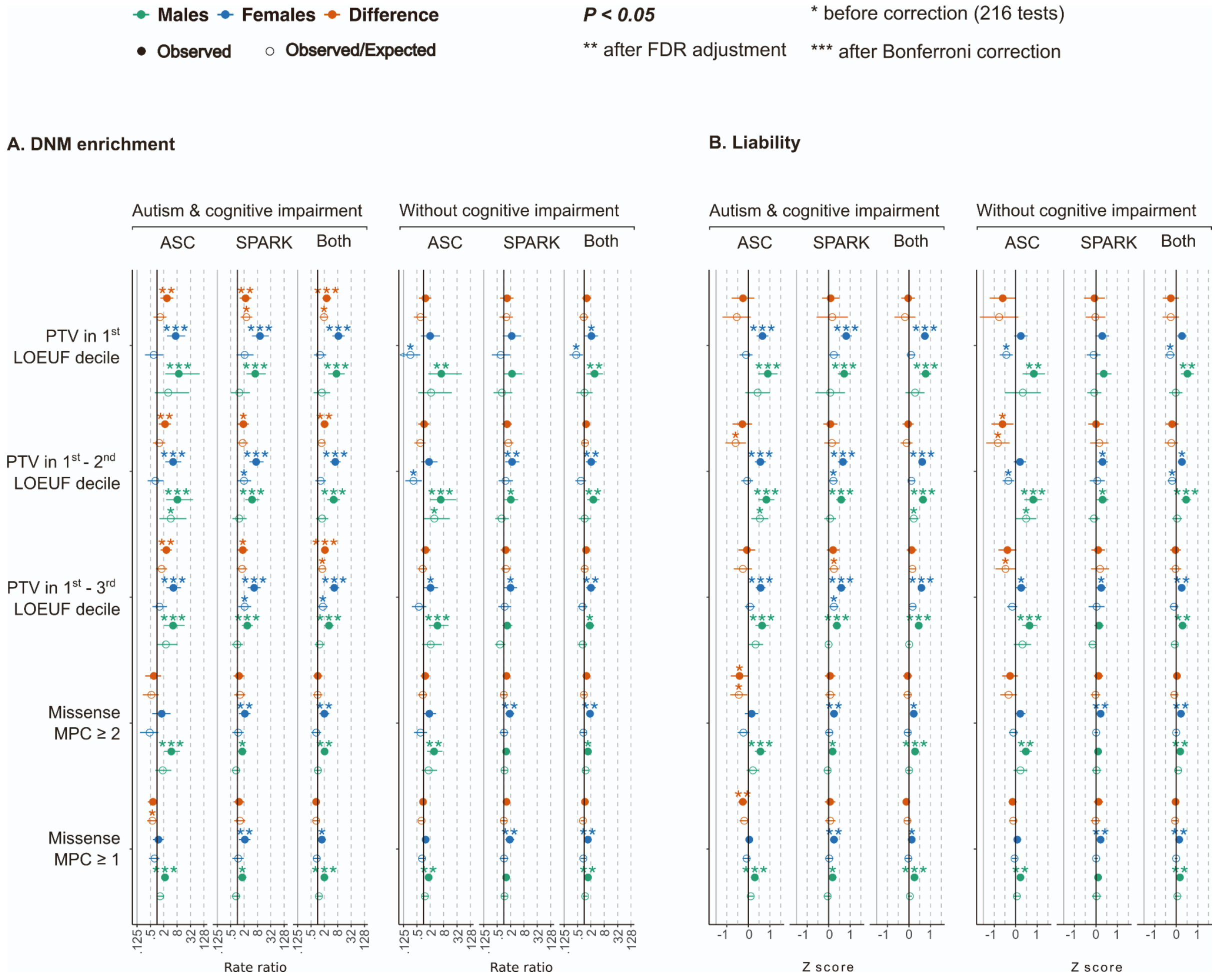

Figure S20: Burden and liability of *de novo* mutations in autosomal protein-coding genes showing male-biased expression in the human adult cortex.

A, Enrichment of damaging *de novo* mutations in a set of 2,852 sex-differentially expressed protein-coding genes with higher expression in males compared to females in two RNA-seq datasets (483 genes in the 1st LOEUF decile, 443 in the 2nd decile, 416 in the 3rd decile; see section 7.1 of the Supplemental Methods). The enrichment was calculated separately in those with or without cognitive impairment (including those with unknown status). Additional comparisons were performed to compare the observed *de novo* rates and effect sizes to what is expected for a matched gene set ('Observed/Expected'). **B**, The liability conveyed by *de novo* mutations in this gene set. Error bars show 95% confidence intervals. For sex differences, a rate ratio > 1 indicates that females show a higher burden; a Z score > 0 indicates that females show a higher effect size on the liability scale.

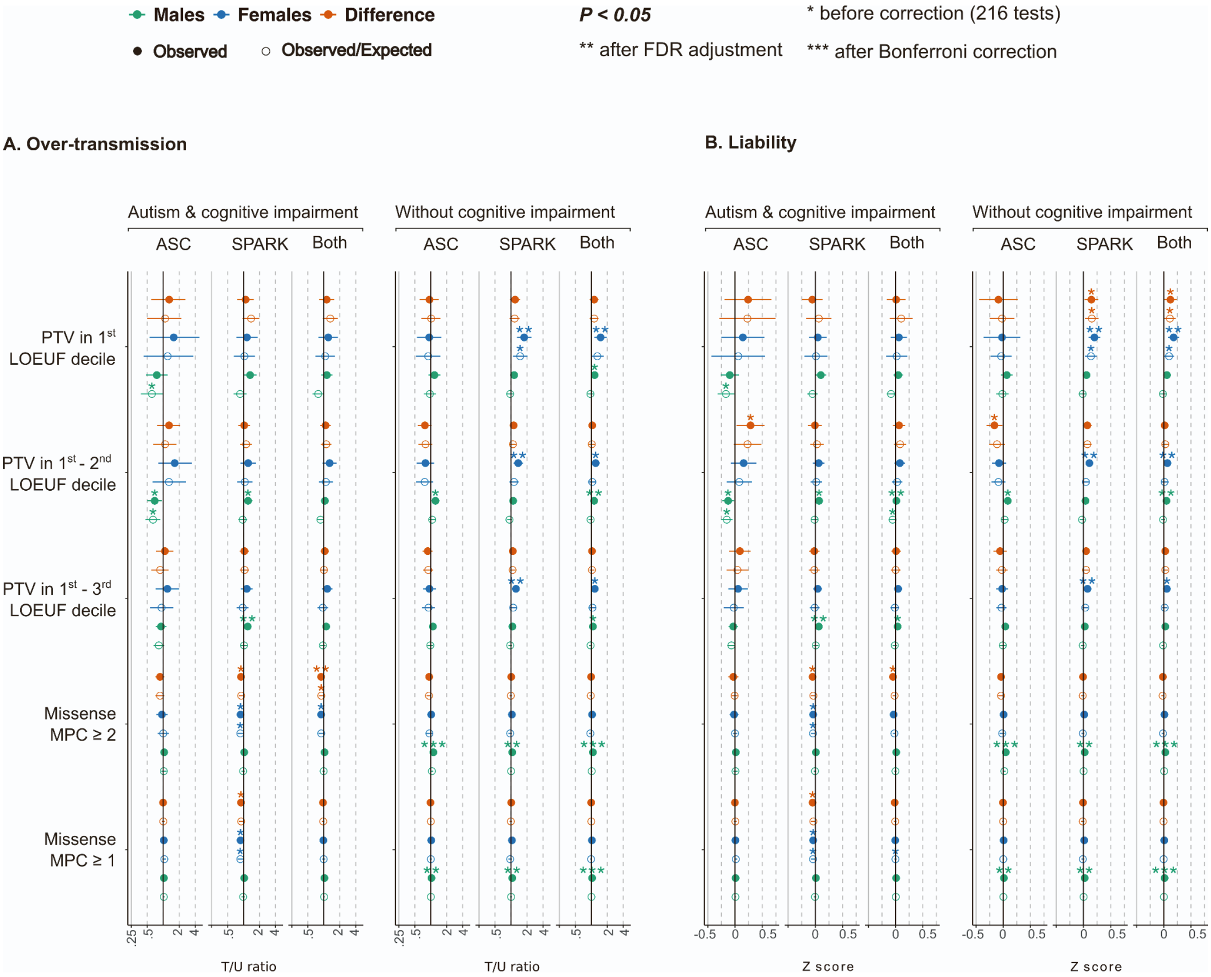

Figure S21: Over-transmission and liability of rare inherited variants in autosomal protein-coding genes showing male-biased expression in the human adult cortex.

A, Over-transmission of rare damaging variants in a set of 2,852 sex-differentially expressed protein-coding genes with higher expression in males compared to females in two RNA-seq datasets (483 genes in the 1st LOEUF decile, 443 in the 2nd decile, 416 in the 3rd decile; see section 7.1 of the Supplemental Methods). The enrichment was calculated separately in those with or without cognitive impairment (including those with unknown status). Additional comparisons were performed to compare the observed transmission rates and effect sizes to what is expected for a matched gene set (‘Observed/Expected’). **B**, The liability conveyed by inherited alleles in this gene set. Error bars show 95% confidence intervals. For sex differences, a transmission ratio > 1 indicates that females show a higher over-transmission; a Z score > 0 indicates that females show a higher effect size on the liability scale.

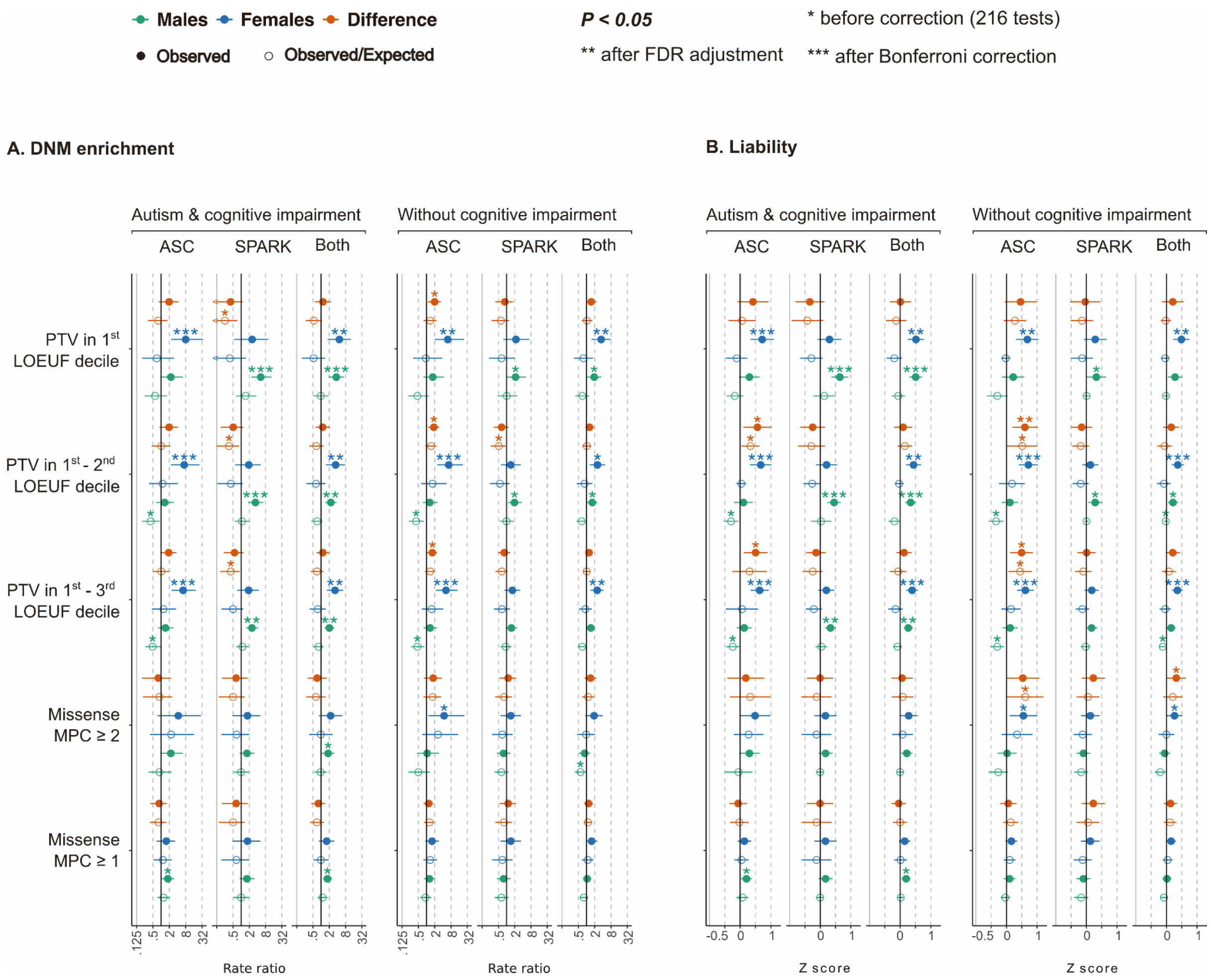

Figure S22: Burden and liability of *de novo* mutations in autosomal protein-coding genes showing female-biased expression in the human adult cortex.

A, Enrichment of damaging *de novo* mutations in a set of 2,427 sex-differentially expressed protein-coding genes with higher expression in females compared to males in two RNA-seq datasets (381 genes in the 1st LOEUF decile, 344 in the 2nd decile, 319 in the 3rd decile; see section 7.1 of the Supplemental Methods). The enrichment was calculated separately in those with or without cognitive impairment (including those with unknown status). Additional comparisons were performed to compare the observed *de novo* rates and effect sizes to what is expected for a matched gene set ('Observed/Expected'). **B**, The liability conveyed by *de novo* mutations in this gene set. Error bars show 95% confidence intervals. For sex differences, a rate ratio > 1 indicates that females show a higher burden; a Z score > 0 indicates that females show a higher effect size on the liability scale.

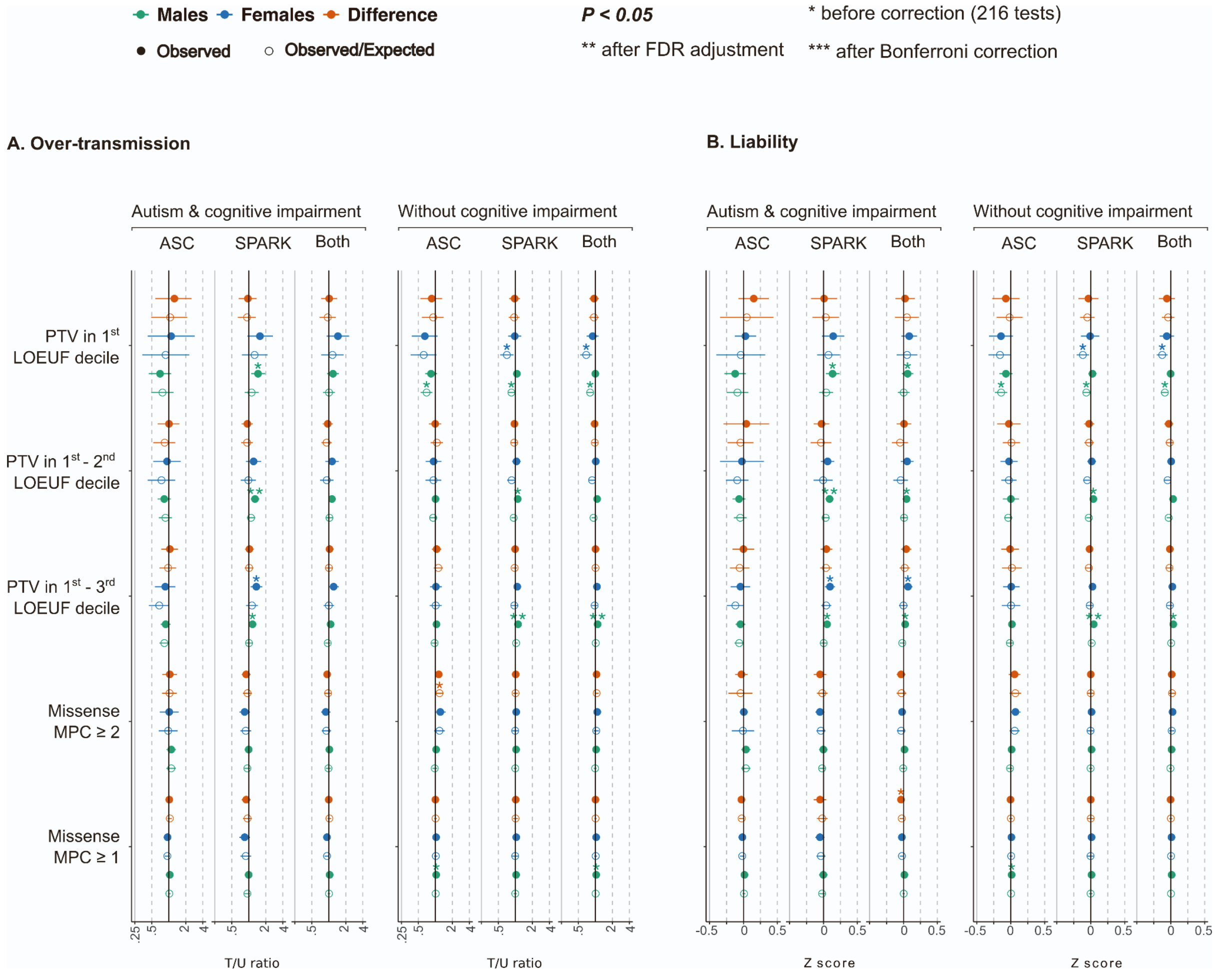

Figure S23: Over-transmission and liability of rare inherited variants in autosomal protein-coding genes showing female-biased expression in the human adult cortex.

A, Over-transmission of rare damaging variants in a set of 2,427 sex-differentially expressed protein-coding genes with higher expression in females compared to males in two RNA-seq datasets (381 genes in the 1st LOEUF decile, 344 in the 2nd decile, 319 in the 3rd decile; see section 7.1 of the Supplemental Methods). The enrichment was calculated separately in those with or without cognitive impairment (including those with unknown status). Additional comparisons were performed to compare the observed transmission rates and effect sizes to what is expected for a matched gene set (‘Observed/Expected’). **B**, The liability conveyed by inherited alleles in this gene set. Error bars show 95% confidence intervals. For sex differences, a transmission ratio > 1 indicates that females show a higher over-transmission; a Z score > 0 indicates that females show a higher effect size on the liability scale.

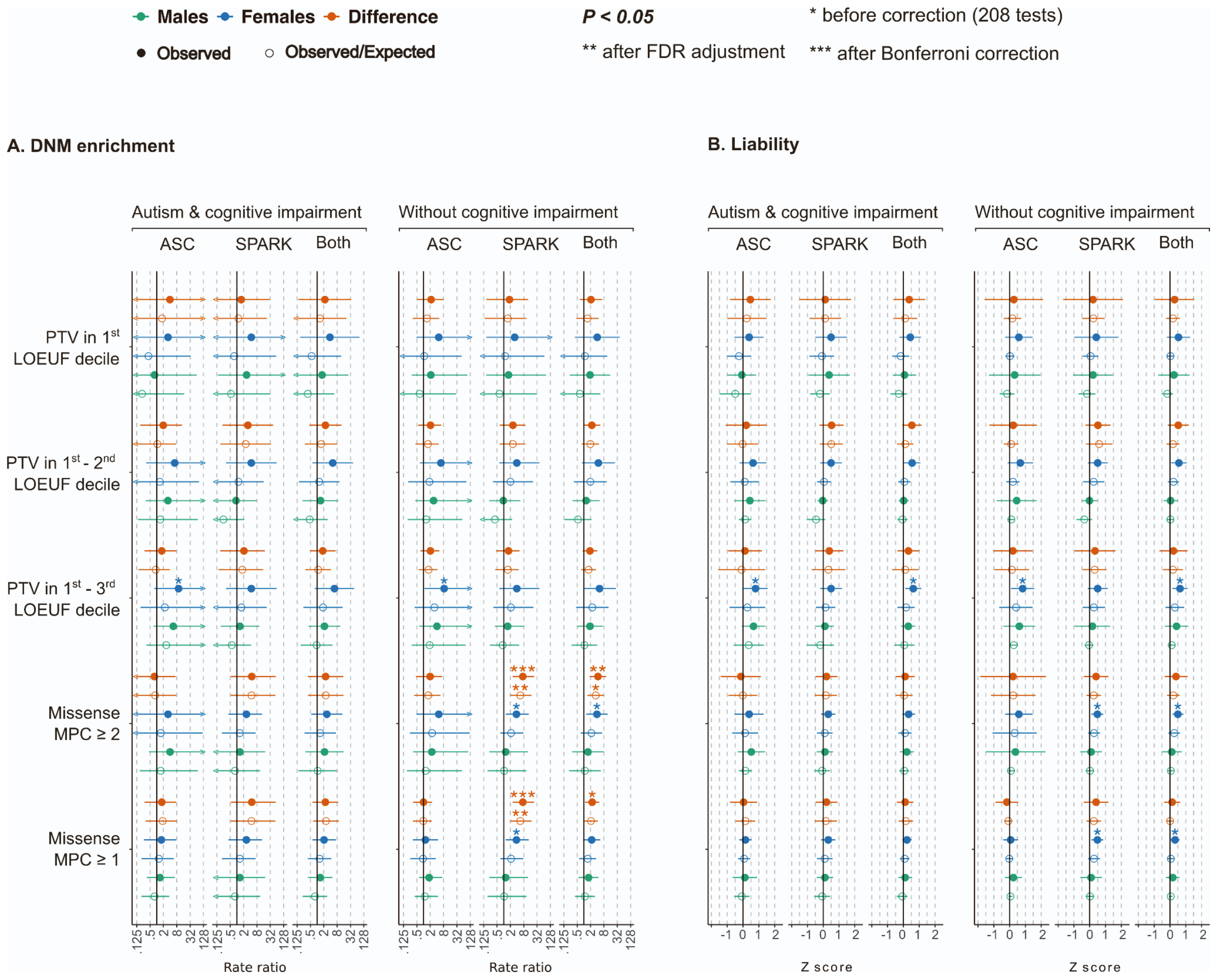

Figure S24: Burden and liability of *de novo* mutations in autosomal protein-coding genes showing male-biased expression in the human fetal cortex.

A, Enrichment of damaging *de novo* mutations in a set of 305 sex-differentially expressed protein-coding genes with higher expression in males compared to females in two RNA-seq datasets (39 genes in the 1st LOEUF decile, 49 in the 2nd decile, 42 in the 3rd decile; see section 7.1 of the Supplemental Methods). The enrichment was calculated separately in those with or without cognitive impairment (including those with unknown status). Additional comparisons were performed to compare the observed *de novo* rates and effect sizes to what is expected for a matched gene set ('Observed/Expected'). **B**, The liability conveyed by *de novo* mutations in this gene set. Error bars show 95% confidence intervals. For sex differences, a rate ratio > 1 indicates that females show a higher burden; a Z score > 0 indicates that females show a higher effect size on the liability scale.

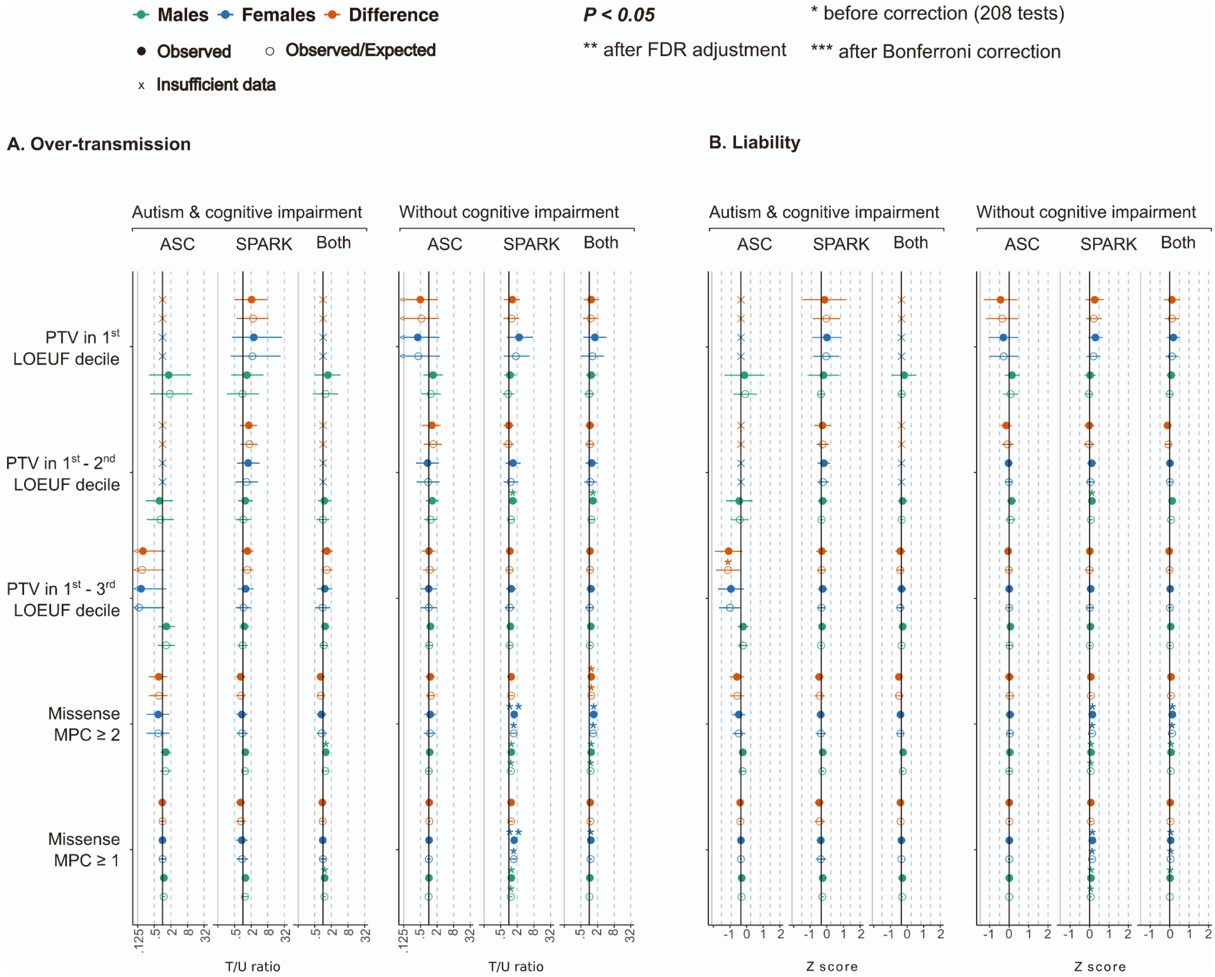

Figure S25: Over-transmission and liability of rare inherited variants in autosomal protein-coding genes showing male-biased expression in the human fetal cortex.

A, Over-transmission of rare damaging variants in a set of 305 sex-differentially expressed protein-coding genes with higher expression in males compared to females in two RNA-seq datasets (39 genes in the 1st LOEUF decile, 49 in the 2nd decile, 42 in the 3rd decile; see section 7.1 of the Supplemental Methods). The enrichment was calculated separately in those with or without cognitive impairment (including those with unknown status). Additional comparisons were performed to compare the observed transmission rates and effect sizes to what is expected for a matched gene set ('Observed/Expected'). **B**, The liability conveyed by inherited alleles in this gene set. Error bars show 95% confidence intervals. For sex differences, a transmission ratio > 1 indicates that females show a higher over-transmission; a Z score > 0 indicates that females show a higher effect size on the liability scale.

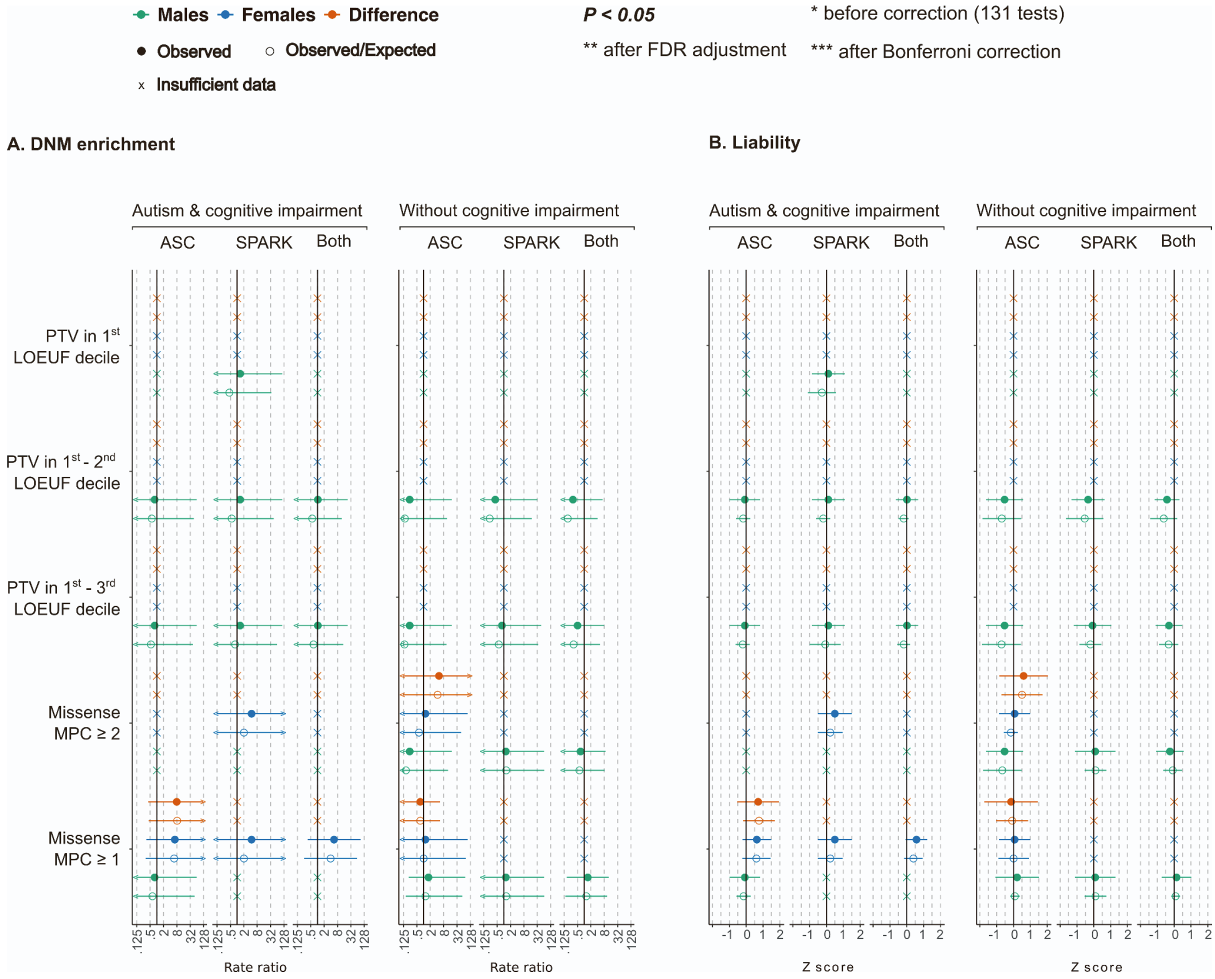

Figure S26: Burden and liability of *de novo* mutations in autosomal protein-coding genes showing female-biased expression in the human fetal cortex.

A, Enrichment of damaging *de novo* mutations in a set of 117 sex-differentially expressed protein-coding genes with higher expression in females compared to males in two RNA-seq datasets (8 genes in the 1st LOEUF decile, 15 in the 2nd decile, 11 in the 3rd decile; see section 7.1 of the Supplemental Methods). The enrichment was calculated separately in those with or without cognitive impairment (including those with unknown status). Additional comparisons were performed to compare the observed *de novo* rates and effect sizes to what is expected for a matched gene set ('Observed/Expected'). **B**, The liability conveyed by *de novo* mutations in this gene set. Error bars show 95% confidence intervals. For sex differences, a rate ratio > 1 indicates that females show a higher burden; a Z score > 0 indicates that females show a higher effect size on the liability scale. These analyses were underpowered and most did not have sufficient data (no variants in probands, siblings or both; marked with 'x').

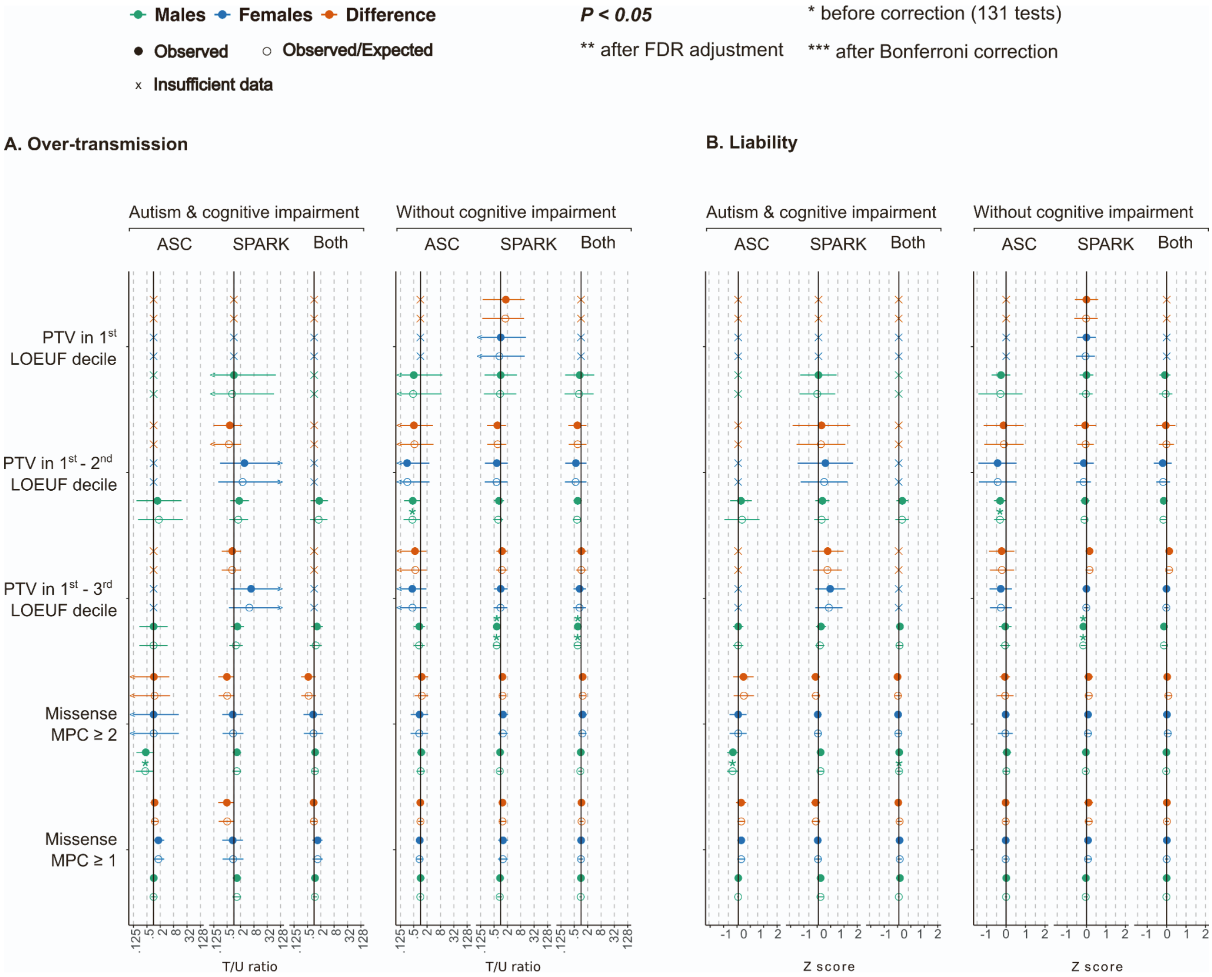

Figure S27: Over-transmission and liability of rare inherited variants in autosomal protein-coding genes showing female-biased expression in the human fetal cortex.

A, Over-transmission of rare damaging variants in a set of 117 sex-differentially expressed protein-coding genes with higher expression in females compared to males in two RNA-seq datasets (8 genes in the 1st LOEUF decile, 15 in the 2nd decile, 11 in the 3rd decile; see section 7.1 of the Supplemental Methods). The enrichment was calculated separately in those with or without cognitive impairment (including those with unknown status). Additional comparisons were performed to compare the observed transmission rates and effect sizes to what is expected for a matched gene set ('Observed/Expected'). **B**, The liability conveyed by inherited alleles in this gene set. Error bars show 95% confidence intervals. For sex differences, a transmission ratio > 1 indicates that females show a higher over-transmission; a Z score > 0 indicates that females show a higher effect size on the liability scale.

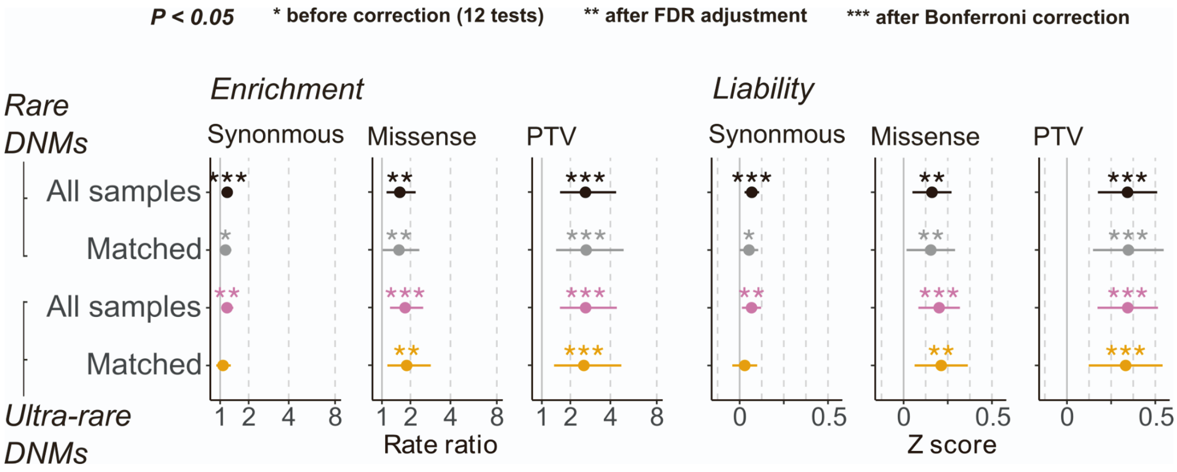

Figure S28: Enrichment of *de novo* mutations in ancestry-matched subset of autistic females without cognitive or motor impairment and siblings.

Enrichment and liability in 'All samples' was tested in 1,464 autistic females without motor or cognitive impairment and sex-matched siblings from different genetic ancestry groups (see Table S4). 'Matched' indicates comparisons between 868 female probands of European ancestry and 2,235 ancestry-matched siblings (see Supplemental Methods section 3.4).

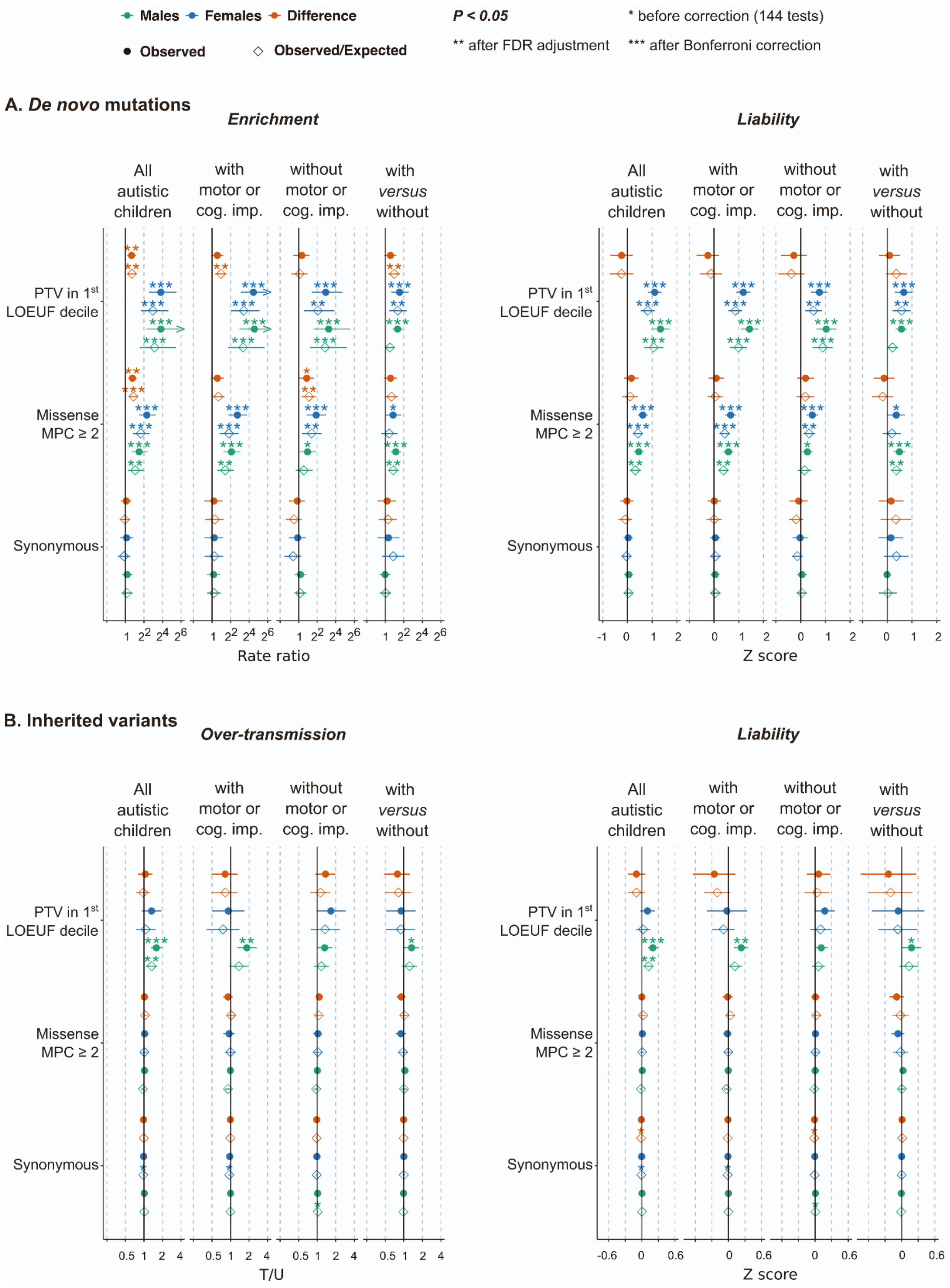

Figure S29: Enrichment of *de novo* mutations and rare inherited in 354 SFARI genes in autistic individuals with and without motor and cognitive impairment in SPARK trios.

Enrichment analysis of *de novo* mutations (DNM) (**A**) and rare variant over-transmission analysis (**B**) in SFARI high-confidence and syndromic genes in all SPARK trios, two SPARK sub-cohorts of autistic individuals ascertained to have autism with or without co-occurring developmental delay or cognitive impairment (*versus* siblings), and a direct comparison between these two groups. The rate ratio (DNMs) and transmitted/untransmitted alleles ratio (inherited variants) are shown on the left-hand side (i.e. observed scale); Average variant liability attributed to these variants is shown on the right-hand side. The enrichment and liability was also compared to a set of matched genes selected to have similar coding length, LoF-constraint, and brain expression.

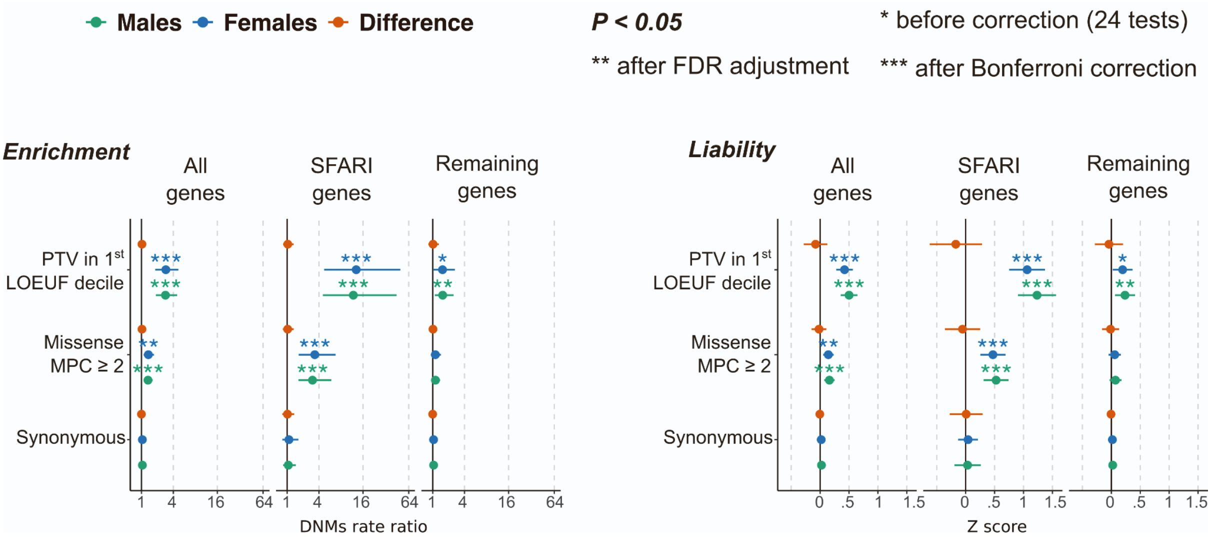

Figure S30: Permutation analysis in SPARK.

Each trio-sequenced sample was randomly assigned to the males or females cohort. This was performed within each phenotypic group in SPARK (autism with co-existing motor or cognitive difficulties, without co-occurring difficulties, unknown status, siblings) to keep the proportion of autistic individuals with or without motor or cognitive impairment constant. The enrichment and liability shown here were then calculated across all autistic individuals.

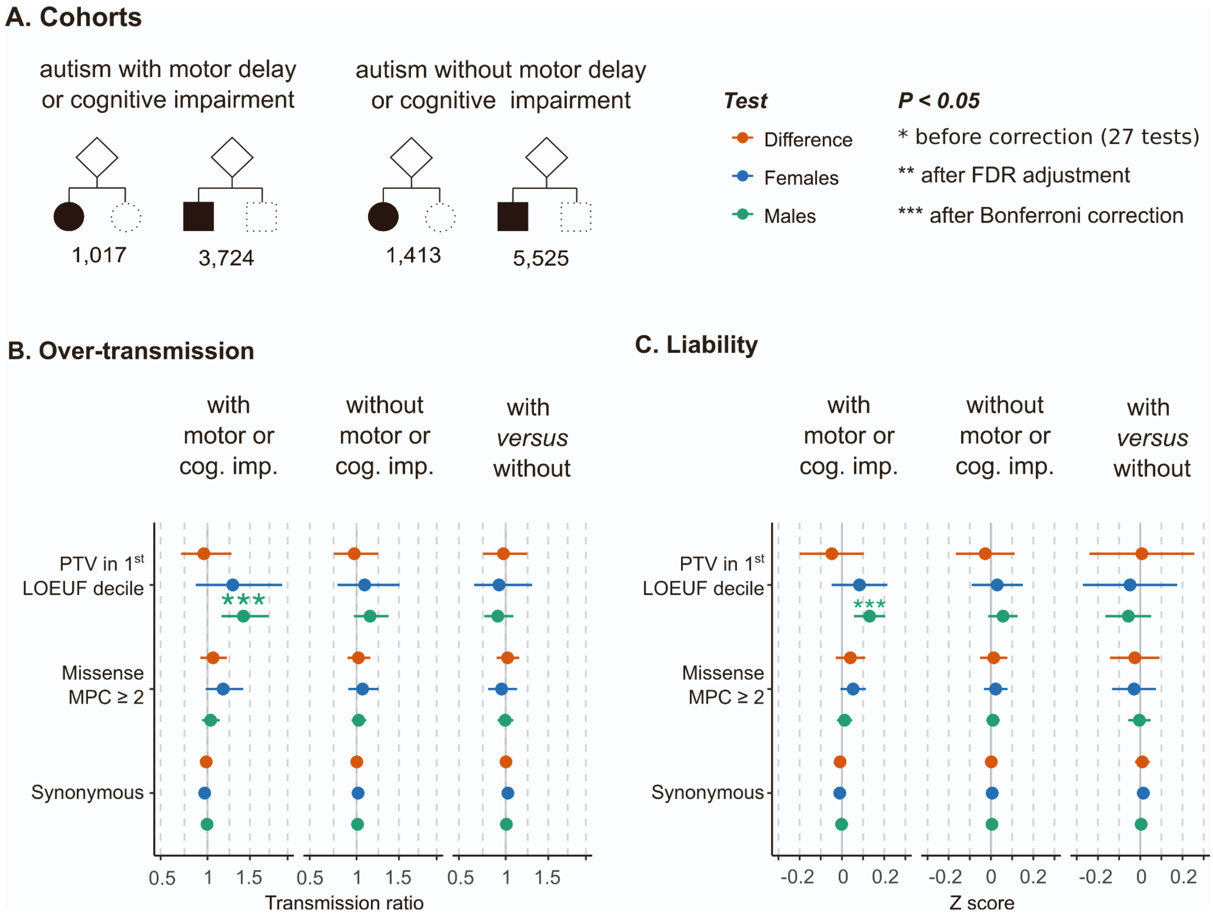

Figure S31: Over-transmission of ultra-rare inherited variants in SPARK individuals with one sequenced parent, stratified by motor and cognitive impairment.

A, The sample size of the different cohorts included in this analysis (note that the transmission analysis is done in probands only). **B**, Over-transmission analysis showing the ratio between rare parental alleles transmitted to autistic individuals with or without motor or cognitive impairment and untransmitted alleles. Transmission ratios were also compared between these two groups (see the Methods). **C**, The liability attributable to inherited rare variants. Related to the analysis presented in Figure S13A ('SPARK Duos').

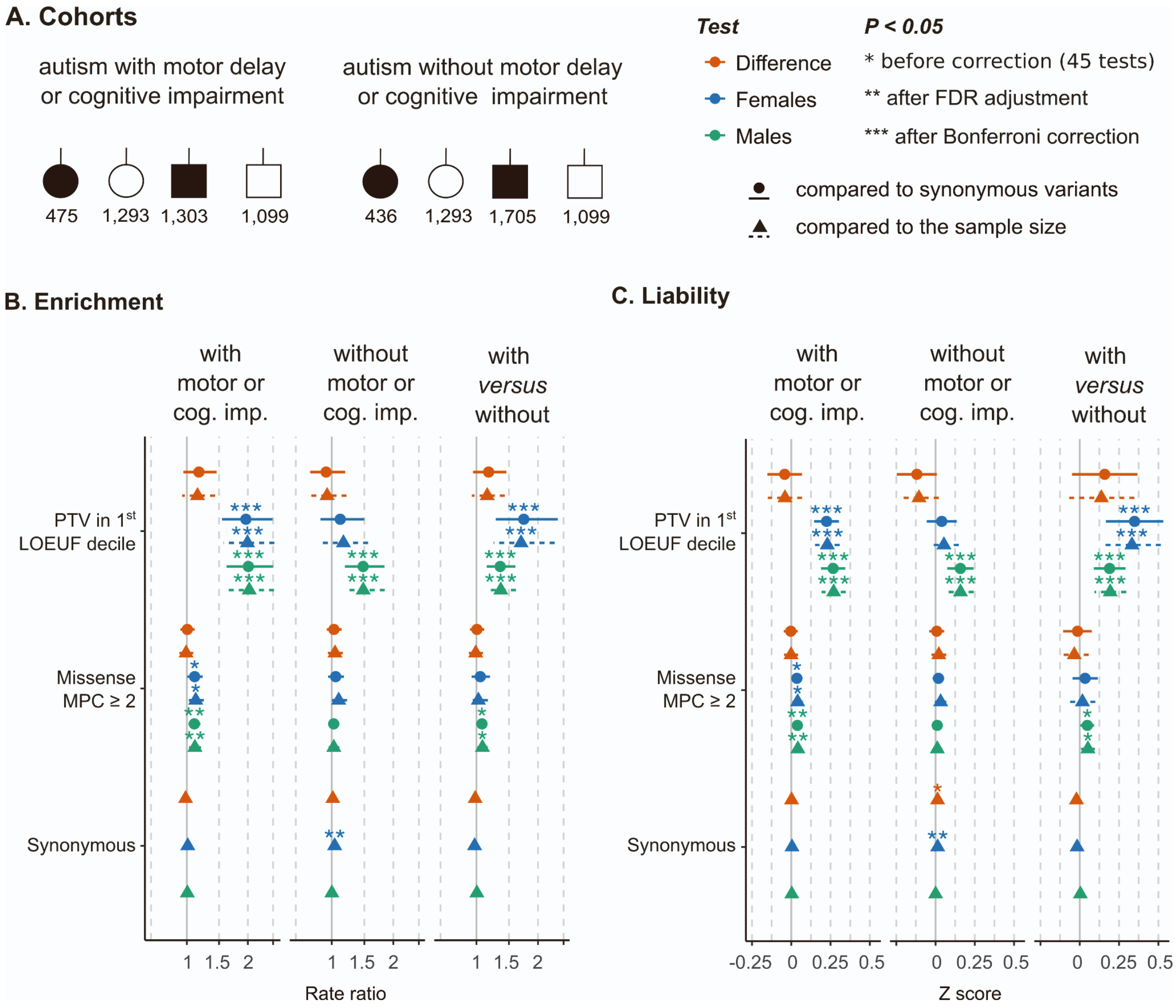

Figure S32: Enrichment of ultra-rare variants in SPARK individuals without sequenced parents, stratified by motor and cognitive impairment.

The enrichment of ultra-rare variants in a cohort of autistic probands without parental sequence data in SPARK (**A**) was examined on the observed scale (**B**) and liability scale (**C**). Variant rates were compared between autistic stratified by co-occurring cognitive or motor impairment and siblings not diagnosed with autism as well as between the two autism sub-cohorts ('with *versus* without') . The error bars show the 95% confidence intervals of the effect sizes. The enrichment and liability were assessed relative to the sample size of the case/control cohorts (i.e. assuming that the expected rare variant burden per sample is similar across cohorts). To account for the differences in ultra-rare variant counts arising from the differences in ancestry, damaging missense and protein truncating variants were also compared to the expected rate ratio from synonymous variants (i.e. normalizing the average variant rates using synonymous variant counts). This analysis is related to the analysis presented in Figure S14.

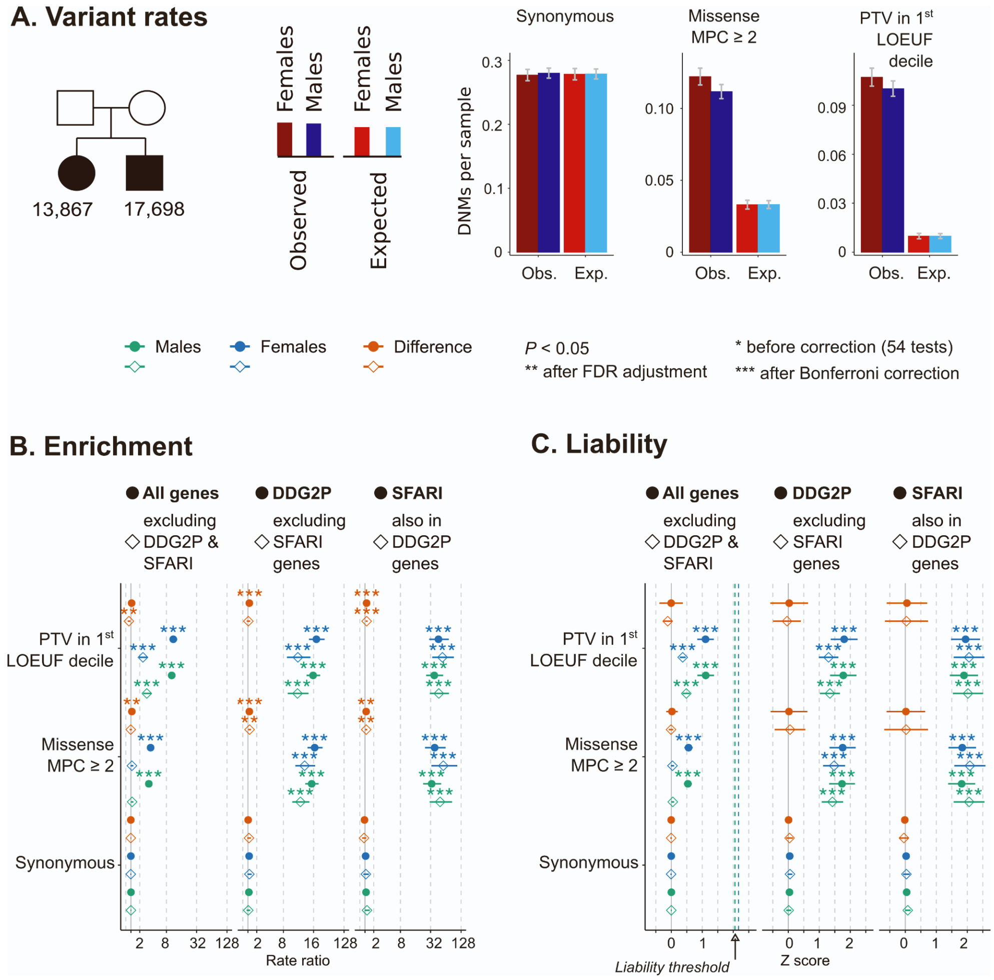

Figure S33: Enrichment and liability of damaging *de novo* mutations in 31,565 children diagnosed with neurodevelopmental disorders.

The observed *de novo* mutation rates in these trios were compared to the expected rates from a mutational model (**A**). The enrichment Sex-stratified *de novo* mutation rate ratios (**B**) and liability (**C**) were calculated for 17,296 autosomal protein-coding genes (1,742 in the 1st LOEUF decile), 845 mono-allelic DDG2P genes associated with neurodevelopmental conditions (384 in the 1st LOEUF decile), and 354 SFARI high-confidence and syndromic autism-predisposition genes (218 in the 1st LOEUF decile). The analysis was also performed in 15,032 autosomal protein-coding gene excluding DDG2P & SFARI genes (1,315 in the 1st LOEUF decile), 605 DDG2P genes excluding SFARI genes (209 in the 1st LOEUF decile), and 240 shared genes between SFARI & DDG2P genes (173 in the 1st LOEUF decile). For sex differences, a rate ratio > 1 indicates that females show a higher enrichment than males; a Z score > 0 indicates that females show a higher effect size on the liability scale. Error bars show 95% confidence intervals.

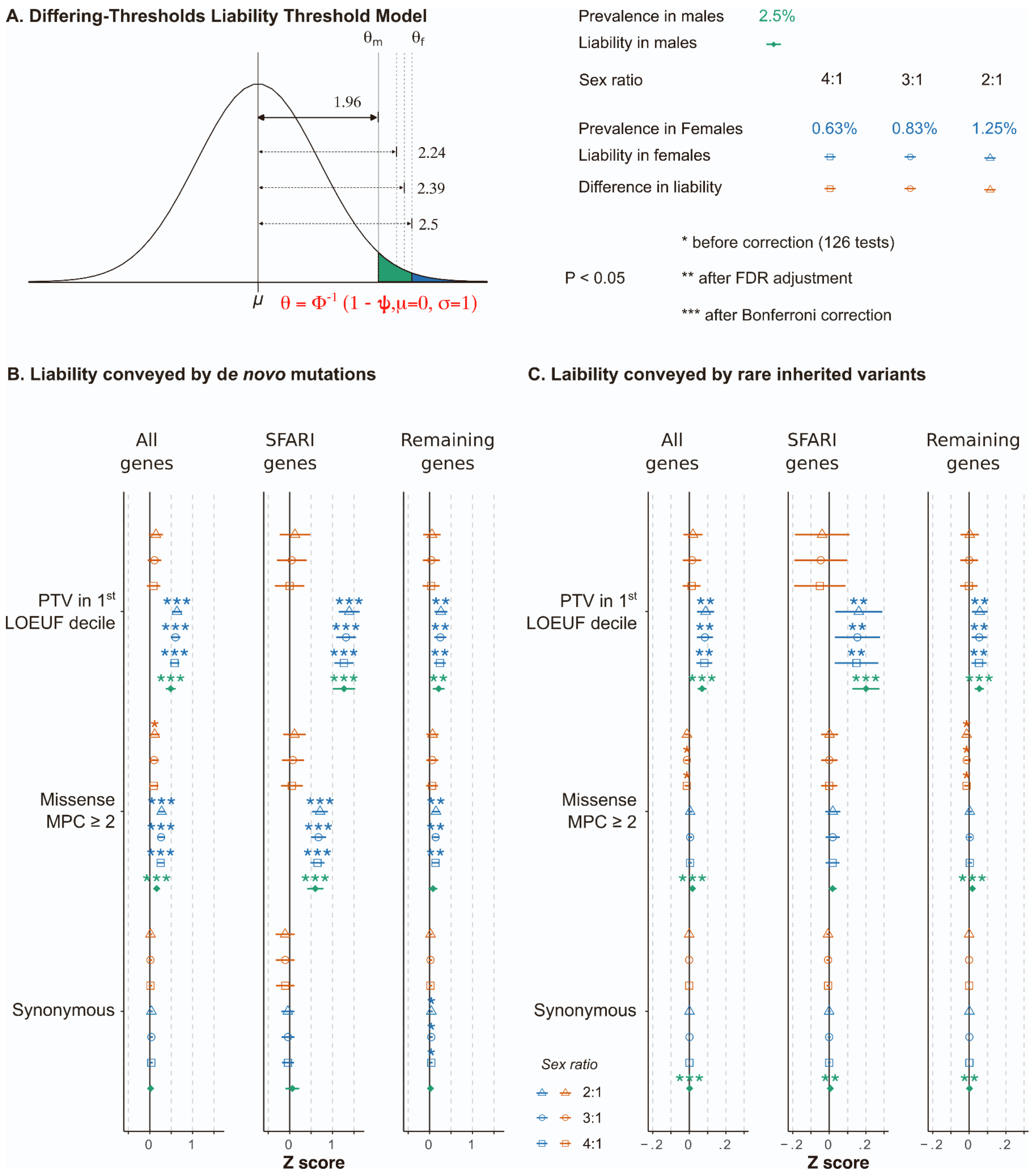

Figure S34: Testing different autism prevalences in females.

The 'standard' Liability Threshold Model (LTM) posits that the genetic liability to autism in the population is normally distributed with equal means (μ) in both sexes and a standard deviation (σ) of 1 (**A**). Since the threshold (θ) determines the prevalence, it can be (back-)calculated from the observed population prevalence using the inverse cumulative density function of the normal distribution (Φ^{-1}). In the Differing-Thresholds LTM, the sex difference in autism prevalence is attributed to females having a higher threshold (θ_f) than males (θ_m). Autism prevalence in males is estimated to be ~2.5%, whereas prevalence estimates in females are less certain (due to e.g., relative rarity or under-diagnosis in females). In the primary analysis (Figure 1), we assumed a prevalence of 0.625% in females (sex ratio of 4:1); Here, we tested higher prevalence estimates (ratios of 3:1 and 2:1). The liability attributed to damaging variants was comparable across a range of autism prevalences in females, both for *de novo* mutations (**B**) and rare inherited variants (**C**). This figure shows a meta-analysis between ASC and SPARK.

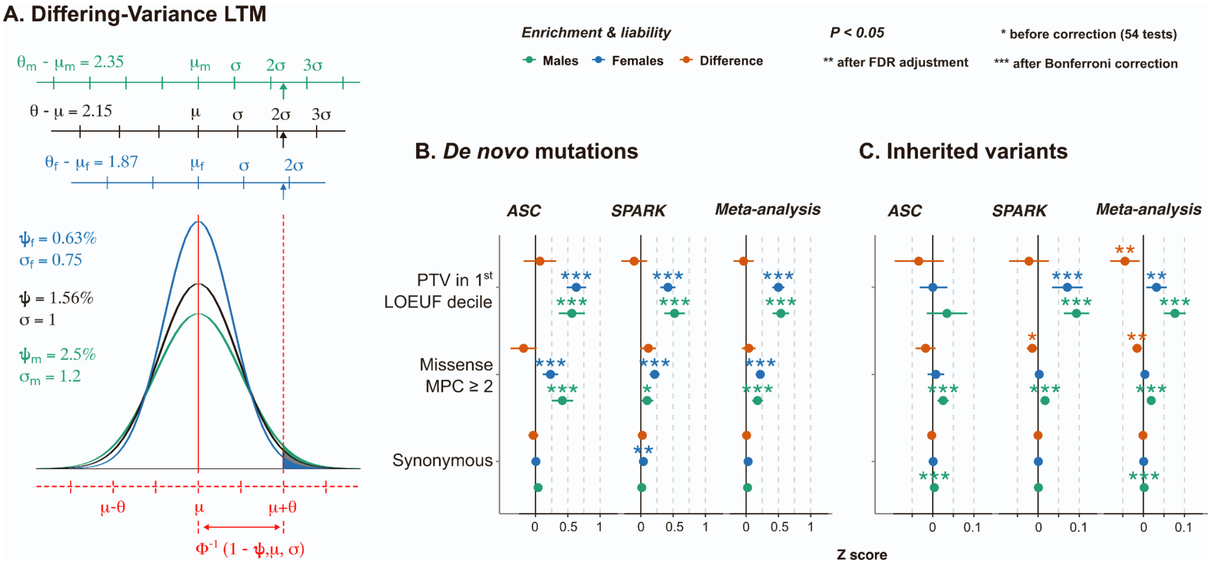

Figure S35: Sex differences in the effect size of de novo and rare inherited variants under different-variances Liability Threshold Model.

Sex differences in autism prevalence can be attributed to the two sexes having differing variances instead of differing thresholds as explained in Figure S4. **A**, An alternative Liability Threshold Model (LTM) which assumes that the liability in the population is normally distributed with a standard deviation (σ) of 1 and equal means (μ=0) in both sexes but higher variance in males (σ_m = 1.2) than females (σ_f = 0.75). Note that the parameters were chosen as a demonstration (the true variance of autism liability in the population is unknown). **B**, The liability conveyed by *de novo* and rare inherited variants under this differing variance model. The error bars indicate the 95% confidence intervals. The effect sizes are shown on the population scale (σ = 1, μ = 0). See section 6.2 of the Supplemental Methods for more details on calculating the effect sizes.

Supplemental Results

1. DNM and inherited variant rates

We processed exome sequencing data from autism probands, siblings and parents from the second integrated release of whole exome sequencing data (WES) from the Simons Powering Autism Research for Knowledge (SPARK iWES2) study and combined these data with data from the SSC and other smaller cohorts previously curated by the ASC. An overview of all analyses is presented in Figure S1. Here we describe the observed variant rates in the study population.

The curation of *de novo* and rare inherited variants is described in section 3 of the Supplemental Methods. The *de novo* mutation rates observed in the probands and siblings in the currently analyzed SPARK release (iWES2) were comparable to, albeit slightly lower than, the variant rates seen in the ASC cohort. We identified ~0.22 - 0.25 synonymous *de novo* mutations per child (across probands and siblings) in our analysis of SPARK iWES2 trios - slightly lower than the previous ASC analysis of SPARK Pilot/WES1 samples (first sequencing wave) which identified ~0.27 synonymous mutations per child (Figure S6). Small differences are expected given the different *de novo* calling pipelines (see section 3 of the Supplemental Methods) and these rates are within the range seen in other studies. The rate of synonymous DNMs from two different previous analyses of SPARK first sequencing wave was between ~0.20 - 0.23 per child in one study[1] and ~27 - 0.32 per child in another.[2] An integrated analysis across several studies of neurodevelopmental disorders identified between 0.2 - 0.3 synonymous mutations per trio.[3] Though damaging missense variants were defined similarly (MPC ≥ 2), we note that the ASC used a different (earlier) version of MPC scores (ExAC-based v1) than the version we used for SPARK iWES2 (gnomAD-based v2). However, the fraction of missense DNMs with MPC score ≥ 2 among siblings not diagnosed with autism was similar between ASC (4.8%) and SPARK (4.98%).

The rates of rare inherited variants (MAF < 0.1%) in SPARK iWES2 were higher than those seen in the ASC cohort and this difference was most prominent in damaging missense variants. In addition to technical differences, average rare inherited variant counts are also sensitive to population differences, partly because different ancestral groups differ in their demographic histories and hence allele frequency spectra, and also because of how the variants are filtered based on in-sample frequencies. While the ASC cohort is predominantly European (composed of the Simons Simplex Collection and other smaller cohorts; see Figure S4), SPARK has more diversity, being only 73% European ancestry in iWES2 (see

Figure S2 and Table S3). To have a more informative comparison of potential artifactual differences in processing less confounded by the differences in ancestry, we explored the variant rates in the previously analyzed SPARK cohort (Pilot and first sequencing wave WES1; 78% European ancestry), which was processed using the same pipeline as the ASC. The rates (per child) of rare inherited synonymous variants in SPARK Pilot/WES1 were also higher than in the other jointly-processed ASC cohorts but were comparable to the currently used SPARK iWES2 dataset processed differently (Figure S6). This suggests that these differences in rare variant rates are a reflection of the diverse genetic ancestry of SPARK samples more so than mere technical differences.

Ultra-rare variants (transmitted/untransmitted; case/(sib-)control) were defined as detailed in section 4 of the Supplemental Methods. To evaluate over-transmission in SPARK probands who have exome data from one parent (duos), we examined the rates of transmitted and untransmitted ultra-rare inherited variants - i.e. those seen in one parent in the dataset & absent from gnomAD. We also defined ultra-rare variants in SPARK and ASC trios in a similar manner to allow joint analysis of parent of origin effects in all child-parent pairs. Ultra-rare synonymous variant rates were comparable between those duo-sequenced children in SPARK and trio-sequenced children in SPARK and ASC with an average of ~5-6 transmitted ultra-rare synonymous alleles per child (Figure S7A). Ultra-rare 'case-control' synonymous variant rates (i.e., in those without parental sequence data; gnomAD MAF ~ 0.005%) were also comparable between the ASC case-control cohorts and SPARK probands/siblings without parental sequence data (Figure S7B).

2. Sex differences in autism likelihood conferred by exome-wide rare variants

Here we describe in more detail the cohort-level results shown in Figure 1 and related analyses in the remaining cohorts.

2.1. *De novo* mutations

2.1.1. Damaging protein-truncating DNMs

Damaging protein-truncating DNM rates in autistic males were three times higher than sex-matched siblings, and were comparable in SPARK and ASC; We found a 2.9 fold-enrichment in SPARK (95% CI = 2 - 4.3; $p = 2.1 \times 10^{-10}$; Bonferroni-corrected $p = 1.1 \times 10^{-8}$) and a 3.1 fold-enrichment in ASC (95% CI = 1.9 - 5.3 ; $p = 5.1 \times 10^{-8}$; Bonferroni-corrected p =

$2.8x10^{-6}$). The enrichment in females *versus* sex-matched siblings was slightly stronger than what is seen in males in SPARK (risk ratio = 3.6; 95% CI = 2.4 - 5.5; p = $4.8x10^{-12}$; Bonferroni-corrected p = $2.6x10^{-10}$) and much more pronounced in females in the ASC cohort (risk ratio = 6.4, 95% CI =3.8 - 11.6 ; p = $1.7x10^{-17}$; Bonferroni-corrected p = $9.4x10^{-16}$). The most notable sex difference was seen in *de novo* damaging PTV rates in the ASC cohort, where DNMs rates in autistic females were 1.8 times higher than autistic males (95% CI = 1.4 - 2.2; p = $5.1x10^{-7}$; Bonferroni-corrected p = $2.7x10^{-5}$). The difference in SPARK was lower (risk ratio = 1.3; 95% CI = 1 - 1.6) and significant only before correction for multiple testing (p = 0.04). SFARI genes showed significant sex differences on the observed scale. An enrichment of protein-truncating *de novo* mutations, in both sexes, was still observable when removing these known autism predisposition genes, however without significant sex differences (Figure S10A).

On the liability scale, *de novo* damaging PTVs increased the liability by 0.5 standard deviation units in SPARK - similarly for both sexes (95% CI: 0.3 - 0.6 ; Bonferroni-corrected p < $3x10^{-9}$). In the ASC cohort, they also increased the liability by 0.5 units in males (95% CI = 0.3 - 0.7; Bonferroni-corrected p = $3x10^{-7}$), and caused a larger shift in females (Z = 0.75; 95% CI = 0.6 - 0.9 ; Bonferroni-corrected p = $4.7x10^{-16}$), albeit not significantly different from males (p = 0.09). SFARI genes showed the same general patterns of protein-truncating DNM liability, with larger effect sizes. Similar patterns were observed when removing these known autism predisposition genes, with considerably lower effect sizes (Figure S10A). We note here that the discovery of SFARI genes was based, in part, on these datasets.

2.1.2. Damaging missense DNMs

Damaging missense mutations showed comparable enrichment in females *versus* siblings in SPARK (risk ratio = 2; 95% CI = 1.5 - 2.6 ; p = $7x10^{-8}$; Bonferroni-corrected p = $3.8x10^{-6}$) and ASC (risk ratio = 2.1; 95% CI = 1.4 - 3.1; p = 0.00013; Bonferroni-corrected p = 0.0075). The enrichment in males was slightly higher in the ASC (risk ratio = 2.4; 95% CI = 1.6 - 3.6 ; p = $8x10^{-7}$; Bonferroni-corrected p = $4.3x10^{-5}$) but considerably lower in SPARK (risk ratio = 1.2; 95% CI = 1 - 1.5 ; p = 0.04; not significant after FDR or Bonferroni correction). In terms of direct comparisons, damaging missense DNMs were slightly more frequent in autistic females *versus* autistic males in SPARK (risk ratio = 1.3 , 1.1 - 1.5; p = 0.0073; FDR-adjusted p = 0.017; FDR-adjusted p = ; Bonferroni-corrected p = 0.39) but did not show a significant sex difference in ASC (p = 0.45).

Females in both ASC and SPARK had comparable variant liability attributed to damaging missense DNMs (ASC = 0.27; 95% CI = 0.13 - 0.4; SPARK = 0.25; 95% CI = 0.16 - 0.24; Bonferroni-corrected p < 0.00044). In males, these missense DNMs showed different

estimates between ASC (Z = 0.38; 95% CI = 0.23 - 0.53) and SPARK (0.09 units; 95% CI = 0.003 - 0.18), and this difference between cohorts was significant ($Z_{Difference}$ = 0.29; 95% CI: 0.12 - 0.46; p = 0.0011). In terms of sex differences, damaging missense DNMs had similar average liability in ASC but significantly lower liability in males in SPARK ($Z_{Difference}$ = -0.16; 95% CI: -0.28 - 0.034; p = 0.013; FDR-adjusted p = 0.031; Bonferroni-corrected p = 0.70). However, these sex-stratified estimates were more congruent between males and females when examining ultra-rare DNMs (see 2.1.3 below), suggesting that the effect sizes are indeed similar. This is in line with the findings from meta-analysis, where the estimates were not significantly different between males and females (Figure 1B). As seen with protein-truncating PTVs, damaging missense DNMs in SFARI genes and the remaining autosomal genes showed similar patterns compared to that obtained with exome-wide analysis, with larger and smaller effect sizes, respectively (Figure S10).

2.1.3. Synonymous DNMs

We note that rare synonymous DNMs showed a small but significant liability in autistic females in SPARK (Z = 0.046, 95% CI = 0.01 - 0.082; p = 0.011; FDR-adjusted p = 0.029). This likely reflects the higher genetic diversity in autistic females (72% individuals of European genetic ancestry) compared to sex-matched siblings (75% individuals of European genetic ancestry). This imbalance, however, did not persist after meta-analyzing the two cohorts (Z = 0.036; 95% CI = 0.0055 - 0.067; p = 0.052). A stringently-defined set of ultra-rare DNMs in SPARK (allele frequency < 0.005%) showed well-balanced synonymous mutation counts between female probands and sex-matched siblings while having the same protein-truncating DNM liability (see 2.1.4 below). Therefore, it is unlikely that the meta-analyzed estimates of damaging DNM liability presented in Figure 1 are biased.

2.1.4. Ultra-rare *de novo* mutations in SPARK

Almost all protein-truncating DNMs and most damaging missense DNMs were ultra-rare, whereas only half of the synonymous variants were in this group (Figure S12). Consequently, the liability of ultra-rare and rare protein-truncating DNMs were similar; the liability of ultra-rare damaging missense DNMs in males (Z = 0.16; 95% CI = 0.06 - 0.25; p = 0.0015) was higher than that seen with rare missense DNMs (Z = 0.09; 95% CI = 0.003 - 0.18; p = 0.04). There was no significant sex difference in ultra-rare DNM liability (p = 0.065) in contrast to what is seen with rare DNMs (p = 0.012). Ultra-rare synonyms DNMs were not enriched in the probands *versus* siblings, nor did they show a significant sex difference.

All in all, this analysis showed a similar (PTVs) or stronger (missense) enrichment in ultra-rare damaging DNMs along with better-balanced ultra-rare synonymous DNM burden,

which suggests that the imbalance in synonymous DNMs seen in the rare variant analysis is not accompanied by an inflation of the estimates for damaging mutations particularly protein-truncating mutations, which showed equal (or rather slightly higher) risk ratios in ultra-rare DNM compared to rare DNMs.

2.2. Rare inherited variants

2.2.1. Damaging protein-truncating variants

Damaging PTVs showed evidence of over-transmission from non-autistic parents to autistic individuals in SPARK only - both to female (transmission ratio = 1.3 ; 95% CI = 1.1 - 1.4; p = 1.8×10^{-4}; Bonferroni-corrected p = 9.5×10^{-3}) and males (T/U = 1.22; 95% CI = 1.14 - 1.3; p = 3×10^{-9}; Bonferroni-corrected p = 1.6×10^{-7}). Similar to the observed over-transmission patterns, inherited damaging PTVs conveyed approximately the same liability in males (Z = 0.085 units; 95% CI = 0.057 - 0.11) and females (Z = 0.082; 95% CI = 0.04 - 0.13).

There was still a significant over-transmission and inherited liability in SPARK when excluding SFARI genes, suggesting that a substantial portion of the genes driving this association are not in the SFARI gene set. In particular, females showed significant over-transmission in the remaining genes rather than SFARI genes, whereas males showed significant over-transmission in both. In the ASC, only SFARI genes showed significant PTV over-transmisisonl, both in males and in females. These findings are shown in Figure S11.

2.2.2. Damaging missense variants

Damaging missense variants showed comparable over-transmission to autistic males but not to females in both cohorts; the transmission ratios were significantly higher than 1 in autistic males in ASC (T/U = 1.06; 95% CI = 1.02 - 1.09; p = 0.00033; Bonferroni-corrected p = 0.018) and in SPARK (T/U = 1.04; 95% CI = 1.02 - 1.06; p = 7.4×10^{-5}; Bonferroni-corrected p = 0.004) but not in females in both (T/U in ASC = 1.02; 95% CI = 0.96 - 1.09; p = 0.47; T/U in SPARK = 1.01; 95% CI = 0.98 - 1.04; p = 0.61). Despite this difference in sex-stratified estimates, there was no statistically significant over-transmission in autistic females when compared directly to autistic males (T/U in ASC = 0.99; 95% CI = 0.94 - 1.04; p = 0.58; T/U in SPARK = 0.99; 95% CI = 0.96 - 1.01; p = 0.32).

The cohort-level liability attributed to inherited damaging missense variants mirrored the over-transmission patterns, where it was significant in males in ASC (Z = 0.023; 95% CI = 0.010 - 0.035) and SPARK (Z = ; 95% CI = 0.015 - 0.0077) but not females, without a significant sex difference (p > 0.093). As noted in the main text, this sex difference was nominally significant in the meta-analyzed cohort (Figure 1). The liability attributed to

inherited damaging missense variants in SFARI genes did not differ significantly from the population mean. Consequently, the liability in the remaining genes mirrored that seen exome-wide (Figure S11B).

2.2.3. Synonymous variants

Inherited parental allele counts in SPARK were not significantly different from untransmitted alleles (Figure 1C). There was an imbalance in transmitted and untransmitted synonymous variants in autistic males in the ASC cohort (Transmitted alleles = 519,579; Untransmited = 515,406; Rate ratio = 1.0081) corresponding to a small effect size on the liability scale (Z = 0.0034; 95% CI = 0.0018 - 0.005; p = 4.12×10^{-5}; Bonferroni-corrected p = 0.0022). This difference is possibly due to the greater sensitivity to call rare variants that are transmitted (i.e. occur at least twice in the dataset) than those that are not transmitted. It was persistent after meta-analysis across cohorts but was extremely small (Z = 0.0016; 95% CI = 0.00070 - 0.0026; p = 0.0001; Bonferroni-corrected p = 0.0056) and thus unlikely to have major implications for the key conclusions.

2.3. Ultra-rare variants

2.3.1. Inherited variants in the remaining child-parent pairs in SPARK

Ultra-rare inherited PTVs ascertained in a cohort of autistic probands with sequence data from a single parent showed similar transmission ratios to females (T/U = 1.3; 95% CI = 1.05 - 0.65 ; p = 0.018; FDR-adjusted p = 0.14; Bonferroni-corrected p = 0.99) and males (T/U = 1.26; 95% CI = 1.12 - 1.43; p = 1.6×10^{-4}; FDR-adjusted p = 2.1×10^{-3}; Bonferroni-corrected p = 8.4×10^{-3}). Accordingly, these variants conveyed similar liabilities in females (Z = 0.068; 95% CI = 0.017 - 0.18) and males (Z = 0.10; 95% CI = 0.048 - 0.15). The effect size of ultra-rare PTVs were similar in trio-sequenced probands, albeit slightly less prominent in males (Figure S13A).

Ultra-rare damaging missense variants showed over-transmission to females (T/U = 1.13; 95% CI = 1.013 - 1.26; p = 0.028; FDR-adjusted p = 0.19; Bonferroni-corrected p = 1) but not males (T/U = 1.017; 95% CI = 0.096 - 1.077; p = 0.57). This was different from ultra-rare variants in trio-sequenced probands in SPARK, in whom these variants were over-transmitted to males (T/U = 1.042; 95% CI = 1.0010 - 1.085; p = 0.045; FDR-adjusted p = 0.20; Bonferroni-corrected p = 1) but not females (T/U = 1.030; 95% CI = 0.95 - 1.11; p = 0.46) (the over-transmission of ultra-rare variants in trios mirrored what was shown above for rare variants in the same individuals in Figure 1C).

Consequently, the liability was significantly higher than zero in females in the one cohort (Z = 0.043; 95% CI = 0.0046 - 0.081) and in males in the other (Z = 0.017; 95% CI = 0.00042 - 0.034), but not significantly higher than zero in a meta-analysis of the two (In males: Z = 0.014; 95% CI = 0.000096 - 0.028; p = 0.12; In females: Z = 0.021 ; 95% CI = -0.00095 - 0.044; p = 0.07). There was no significant sex difference in any of these ultra-rare variant comparisons. Siblings showed nominal under-transmission of PTVs that was not significantly different from zero on the liability scale (Figure S13B).

2.3.2. Ultra-rare variants in cases and controls

Case-control cohorts (including individuals recruited in family-based studies but currently without sequencing data from their parents) form a substantial portion of available autism cohorts. These constitute valuable independent datasets to study sex differences in the enrichment of ultra-rare variants. To leverage these data, we compared the enrichment of ultra-rare variants in 12,125 autistic individuals *versus* 10,962 controls or siblings not diagnosed with autism (Figure S7B). This analysis showed significant enrichment and liability attributed to damaging PTVs, and to a lesser extent damaging missense variants, without significant sex differences (Figure S14). Since the mode of inheritance of alleles in these cohorts is unknown, this analysis captures the combined effect size of *de novo* mutations and inherited variants. On the liability scale, the effect sizes attributed to damaging PTVs in females (Z=0.21; 95% CI = 0.16 - 0.25; p $=3.81\times10^{-21}$; Bonferroni-corrected p = 1.71×10^{-19}) and males (Z=0.28; 95% CI = 0.22 - 0.33; p = 8.03×10^{-36}; Bonferroni-corrected p = 3.61×10^{-34}) were not significantly different ($Z_{Difference}$ = -0.072; 95% CI = -0.14 - 0.0018 ; p =0.056).

2.3.3. Ultra-rare variants in mothers and fathers, parent-of-origin effects, and male-biased vulnerability

Previous work has shown that mothers of autistic children are more likely to carry rare damaging variants than fathers.[4] It was also hypothesized that the protective effect in females will render male children especially vulnerable to rare damaging alleles transmitted from mothers and conversely, females will be most protected against alleles transmitted from fathers.[5] Earlier work has shown relatively higher over-transmission from mothers (than fathers) to autistic male children,[4,6,7] but the findings from a more recent analysis were not consistent with the hypothesis of increased 'male vulnerability' to maternally inherited alleles.[1] These studies were performed in smaller cohorts largely overlapping with the current cohort. We therefore examined the burden of ultra-rare variants in mothers versus fathers of autistic children in a larger sample size, then went on to evaluate

over-transmission to male and female children (to test whether there are parent-of-origin or sex-biased protective effects).

First, we performed a 'case-control' analysis to compare the burden of ultra-rare parental alleles (seen in one parent and not in gnomAD) between the mothers and fathers of autistic individuals. Given the similar rates of ultra-rare parental alleles in ASC & SPARK (~6 synonymous ultra-rare parental alleles per child; Figure S7), we pooled all child-parent pairs in a mega-analysis (n = 55,521 child-parent pairs). Across these child-parent pairs, we found that the average rate of ultra-rare damaging protein-truncating variants in the mothers of autistic children (n = 22,496) were significantly higher than the rates in the fathers (n = 33,025) (Figure S15). This was most noticeable in PTVs in the 1st LOEUF decile (rate ratio = 1.15; 95% CI = 1.08 - 1.22; $p = 2.67 \times 10^{-6}$; Bonferroni-corrected $p = 1.34 \times 10^{-5}$) and less so in PTVs in the 2nd decile (rate ratio = 1.08; 95% CI = 1.03 - 1.14; $p = 2.9 \times 10^{-3}$; Bonferroni-corrected p = 0.015). Ultra-rare damaging missense variants with MPC scores ≥ 2 were also enriched in mothers compared to fathers (rate ratio = 1.06; 95% CI = 1.03 - 1.09; $p = 3.0 \times 10^{-5}$; Bonferroni-corrected $p = 1.5 \times 10^{-4}$), more so than missense variants with MPC scores between 1 & 2 (rate ratio = 1.015; 95% CI = 1.0031 - 1.027; p = 0.013; Bonferroni-corrected p = 0.065).

Next, we calculated the transmission ratios (T/U) of ultra-rare alleles transmitted from mothers to sons (n = 26,021 pairs), mothers to daughters (n= 7,004), fathers to sons (n = 17,852), and fathers to daughters (n = 4,644) as well as the liability conveyed by these variants (Z scores). (Instead of autistic males and females, we use the terms sons and daughters to refer to autistic children in this section to avoid confusion with the sex of the parent). We performed six pairwise comparisons (listed below; see section 5.3 of the Supplemental Methods for details) to test whether there are parent-of-origin effects and whether sons are more vulnerable to maternally inherited alleles than paternally inherited alleles (conversely, whether daughters are more protected from paternally inherited alleles than maternally inherited alleles).

Specifically, we measured the significance of the following comparisons (the tests numbered 'i' through 'vi' are labeled similarly in Figure S16):

i. Maternal alleles to daughters *versus* paternal alleles to daughters.

ii. Maternal alleles to sons *versus* paternal alleles to sons.

- *When looking at daughters and sons separately, do we see a difference between paternally and maternally inherited alleles*? We expect to see a significant difference if there are parent-of-origin effects e.g., maternal alleles having a larger effect size (than paternal alleles) in sons or paternal alleles having a significantly lower effect size (than maternal alleles) in daughters. In line with all other analyses, we did not test transmission to all children (sons and daughters) combined. Whether there was a

significant difference in such a test or not; we would have done post-hoc sex-stratified tests.

iii. Maternal alleles to daughters *versus* maternal alleles to sons.

iv. Paternal alleles to daughters *versus* paternal alleles to sons.

- *When looking at mothers and fathers separately, do we see a difference between daughters and sons*? If sons are especially vulnerable to maternal alleles, test ‘iii’ will be significant. If daughters are less vulnerable to paternal alleles, we expect to see a significant difference (daughters showing a lower effect size) in test ‘iv’.

v. Maternal alleles to daughters *versus* paternal alleles to sons.

vi. Maternal alleles to sons *versus* paternal alleles to daughters.

- *Is there a significant difference when comparing the most and least vulnerable children?* Test ‘vi’ will be significant if there is a sex difference in genetic predisposition in the child (i.e. sons are more vulnerable to maternally inherited alleles and daughters are more protected from paternally inherited alleles). If test ‘vi’ is significant, the result on test ‘v’ can help indicate whether this is indeed due to a sex difference in the children rather than a parent-of-origin effect; specifically, if both test ‘vi’ and test ‘v’ are significant, this suggests a parent-of-origin effect, whereas if test ‘vi’ is significant and test ‘v’ is not significant, this suggests that there is a sex difference in the child.

We did not find significant sex differences in parental transmission or variant liability that may suggest parent-of-origin effects or sex-biased liability. The transmission ratios and Z scores did not differ significantly across these comparisons ($p > 0.05$) when we tested different thresholds for damaging PTVs (1st, 1st - 2nd and 1st - 3rd LOEUF deciles) or missense variants (MPC ≥ 2, MPC ≥ 1). These results are presented in Figure S16. As the effect sizes of parental alleles were not significantly different, we went on and performed sex-stratified mega-analysis similar to the analysis presented in Figure 1C (n=21,043), leveraging the larger sample size (n=34,478 unique children). Again, we did not find significant sex differences between autistic females and males in these variant classes.

3. Examining the predisposition to autism with and without cognitive difficulties jointly in ASC and SPARK trios

We present here the findings from the analysis shown in Figure 2 and related analyses in gene sets.

3.1. Exome-wide enrichment and liability

The findings from the analysis of de novo mutations are presented in the main text. Here, we provide details on the effect size of inherited rare parental alleles.

3.1.1. *De novo* mutations

The sex differences on the observed scale in the meta-analysis were driven by DNMs from the ASC cohort, which showed female bias both amongst those with cognitive impairment (rate $\text{ratio}_{\text{Sex-Difference/ASC}}$ = 2.27; 95% CI = 1.53 - 3.34; p = $3.3\text{x}10^{-5}$; Bonferroni-corrected p = 0.0036) and without cognitive impairment (rate $\text{ratio}_{\text{Sex-Difference/ASC}}$ = 1.55; 95% CI = 1.18 - 2.03; p = 0.0015; FDR-adjusted p = 0.0047; Bonferroni-corrected p = 0.17). The sex difference in SPARK was not significant whether with cognitive impairment (rate $\text{ratio}_{\text{Sex-Difference/SPARK}}$ = 1.26; 95% CI = 0.86 - 1.82; p = 0.21) or without cognitive impairment (rate $\text{ratio}_{\text{Sex-Difference/SPARK}}$ = 1.23; 95% CI = 0.91 - 1.66; p = 0.16); However, the observed sex differences before stratifying by cognitive impairment was not substantial and only significant before correction for multiple testing (rate $\text{ratio}_{\text{Sex-Difference/SPARK}}$ = 1.27 ; 95% CI = 1.004 - 1.60; p = 0.043; Figure 1B). The sex difference in damaging missense DNMs was significant in SPARK (rate $\text{ratio}_{\text{Sex-Difference/SPARK}}$ = 1.34; 95% CI = 1.07 - 1.51; p = 0.0080; FDR-adjusted p = 0.021; Bonferroni-corrected p = 0.86) but not in the ASC cohort (rate $\text{ratio}_{\text{Sex-Difference/ASC}}$ = 1.16; 95% CI = 0.86 - 1.53; p = 0.30). There was no significant sex difference in damaging missense DNM rates between those with cognitive impairment in the individual cohorts (rate $\text{ratio}_{\text{Sex-Difference/ASC}}$ = 0.92; 95% CI = 0.58 - 1.42; p = 0.75; rate $\text{ratio}_{\text{Sex-Difference/SPARK}}$ = 1.14; 95% CI = 0.81 - 1.60; p = 0.44).

The average effect size of protein truncating DNMs in those with autism and cognitive impairment was 0.66 in females (95% CI = 0.54 - 0.78; p = $1.3\text{x}10^{-26}$; Bonferroni-corrected p = $1.5\text{x}10^{-24}$) and 0.53 in males (95% CI = 0.42 - 0.64; p = $5.2\text{x}10^{-21}$; Bonferroni-corrected p = $5.6\text{x}10^{-19}$), without a significant difference (p = 0.13). In those without cognitive difficulties, it was 0.49 in females (95% CI = 0.37 - 0.60; p = $7.8\text{x}10^{-17}$; Bonferroni-corrected p = $8.4\text{x}10^{-15}$)

and 0.42 in males (95% CI = 0.30 - 0.53; p = $7.4x10^{-12}$; Bonferroni-corrected = $8.0x10^{-10}$), and the difference was similarly not significant (p = 0.24). Damaging missense DNMs increased the average liability in autistic individuals without cognitive impairment by 0.23 units in females (95% CI = 0.15 - 0.31; p = $1.1x10^{-7}$; Bonferroni-corrected p = $1.2x10^{-7}$) and 0.11 in males (95% CI = 0.04 - 0.20; p = 9.8 $x10^{-5}$; Bonferroni-corrected p = 0.011). In those with cognitive impairment, damaging missense DNMs had an average liability of 0.26 in females (95% CI = 0.17 - 0.36; p = $3.8x10^{-7}$; Bonferroni-corrected p = $4.1x10^{-5}$) and 0.24 in males (95% CI = 0.17 - 0.32; p = $0.42x10^{-11}$; Bonferroni-corrected p = $4.5x10^{-9}$). These effect sizes were not significantly different between the two sexes whether with cognitive impairment (p = 0.71) or without it (p = 0.052).

We have shown that the rates and effect sizes of protein-truncating variants in the 2nd-3rd LOEUF decile and missense variants with 2 > MPC ≥ 1 (Figure S8) did not differ by sex, nor the effect sizes of highly deleterious variants (PTVs in 1st LOEUF decile, missense variants with MPC score ≥ 2) outside a limited group of SFARI high-confidence and syndromic genes (Figure S10). We performed a similar analysis across ASC & SPARK stratified by coexisting cognitive impairment, in which we removed SFARI genes and examined stringent and relaxed filtering thresholds (PTVs in 1st/1st-2nd/1st-3rd LOEUF deciles; missense variants with MPC ≥ 1 or MPC ≥ 2). In this meta-analysis, the rate ratios of damaging *de novo* mutations and their attributed liability did not differ significantly between males and females (Figure S17).

The cohort-level analyses revealed more nuanced patterns. First, the effect size of damaging protein-truncating DNMs was significantly higher in females *versus* males with cognitive impairment in ASC ($Z_{Sex-Difference/ASC}$ = 0.30, 95% CI = 0.05 - 0.55, p = 0.020, FDR-adjusted p = 0.054), but not in SPARK ($Z_{Sex-Difference/SPARK}$ = 0.0037; 95% CI = -0.21 - 0.21; p = 0.97). The liability estimated in ASC trios was 0.76 in females (95% CI = 0.57 - 0.94; p = $6.3x10^{-16}$; Bonferroni-corrected p = $6.9x10^{-14}$) and 0.45 in males (95% CI = 0.28 - 0.63; p = $3.7x10^{-7}$; Bonferroni-corrected p = $4.0x10^{-5}$), whereas in SPARK it was 0.58 both in females (95% CI = 0.43 - 0.74; p = $3.3x10^{-13}$; Bonferroni-corrected p = $3.6x10^{-11}$) and males (95% CI = 0.44 - 0.72; p = $2.8x10^{-16}$; Bonferroni-corrected p = $3.0x10^{-14}$). This could reflect an ascertainment bias from including relatively more females with severe phenotypes and profound difficulties (who are more likely to high-impact variants e.g., protein-truncating DNMs) in the ASC cohort resulting in over-estimated effect sizes in females. (To calculate the liability, we estimate the allele frequency of damaging variants in the autistic females in the population from the observed frequency in female probands).

Second, the effect size of damaging missense DNMs was significantly higher in females *versus* males without cognitive impairment in SPARK ($Z_{Sex-Difference/SPARK}$ = 0.17, 95% CI = 0.04 - 0.30, p = 0.010, FDR-adjusted p = 0.027, Bonferroni-corrected p = 1), but not in

ASC ($Z_{Sex\text{-}Difference/SPARK}$ = -0.078; 95% CI = -0.29 - 0.13; *p* = 0.47). In SPARK, the effect size was 0.22 in females (95% CI = 0.12 - 0.32; *p* = 7.4x10^{-6}; Bonferroni-corrected *p* = 8.0x10^{-4}) and 0.05 in males (95% CI = -0.04 - 0.14; *p* = 0.29). In ASC, it was 0.25 in females (95% CI = 0.10 - 0.39; *p* = 0.0007.5x10^{-4}; FDR-adjusted *p* = 0.0027; Bonferroni-corrected *p* = 0.081) and 0.32 in males (95% CI = 0.17 - 0.47; FDR-adjusted *p* =2.7x10^{-5}; p = 1.3x10^{-4}; Bonferroni-corrected *p* = 0.0029). This pattern is similar to what was seen when analyzing the full SPARK cohort before stratification on cognitive impairment (Figure 1B); there, we noticed that more stringent quality/frequency filtering of DNMs attenuates the sex difference in the estimated effect sizes of damaging missense DNMs (Figure S12).

3.1.2. Inherited variants

In the meta-analyzed cohort of autistic individuals with cognitive impairment (Figure 2C), over-transmission of damaging protein-truncating variants was not significantly different (*p* = 0.96) between males (rate ratio = 1.23; 95% CI = 1.09 - 1.38; *p* = 8.2x10^{-4}; FDR-adjusted *p* = 0.0029; Bonferroni-corrected *p* = 0.089) and females (rate ratio = 1.24; 95% CI = 1.0 - 1.53; *p* = 0.054). The sex difference was also not significant (*p* = 0.93) in the remaining individuals, where the transmission ratio was 1.17 in males (95% CI = 1.09 - 1.24; *p* = 3.3x10^{-6}; Bonferroni-corrected *p* = 3.5x10^{-4}) and 1.18 in females (95% CI = 1.04 - 1.34; *p* = 0.0091; FDR-adjusted *p* = 0.024; Bonferroni-corrected *p* = 0.99). In line with the analysis in the full cohort before stratification on cognitive or motor difficulties (Figure 1C), the increased transmission of damaging protein-truncating variants was driven by inherited variants seen in SPARK (Figure 2C). Damaging missense variants displayed a more homogeneous pattern where both ASC & SPARK showed significant, albeit small, over-transmission in males without cognitive impairment but not in females.

On the liability scale, damaging PTVs had a meta-analyzed effect size of 0.066 in females (95% CI = -0.002 - 0.13; *p* = 0.15) and 0.076 in males (95% CI = 0.03 - 0.12, *p* = 7.7x10^{-5}; Bonferroni-corrected *p* = 0.0083) when measured in those with cognitive impairment. The effect size was also comparable in those without cognitive impairment, both in females (Z = 0.06; 95% CI = 0.017 - 0.10; p = 0.0045; FDR-adjusted *p* = 0.013; Bonferroni-corrected *p* = 0.48) and males (Z = 0.062; 95% CI = 0.034 - 0.089; *p* = 1.3x10^{-5}; Bonferroni-corrected *p* = 0.0014). As noted above, this was driven by variants identified in SPARK. The effect size of damaging missense variants on the liability scale in males was 0.018 (95% CI = 0.011 - 0.025; *p* = 2.4x10^{-6}; Bonferroni-corrected *p* = 2.6x10^{-4}). In females, the effect size was lower (Z = 0.0086; 95% CI = -0.0034 - 0.021; *p* = 0.34) but the difference was not significant (*p* = 0.20). In those with cognitive impairment, the liability was not

significantly higher than zero in both sexes. As with transmission ratios, ASC & SPARK showed highly congruent patterns.

3.2. High-confidence and syndromic autism predisposition genes (SFARI genes)

We examined the enrichment and liability conveyed by SFARI high-confidence and syndromic genes across both ASC & SPARK, testing additional relaxed variant filters; We also performed similar comparisons against randomly selected genes matched for coding length, LoF constraint and adult expression.

The meta-analyzed rates of protein-truncating DNMs in SFARI genes (1st LOEUF decile) were significantly higher in autistic females compared to autistic males (Figure S18A), both when examining those with cognitive impairment (rate ratio = 2.0; 95% CI = 1.45 - 2.76, p =2.3x10^{-5}; Bonferroni-corrected p = 5.1x10^{-3}) (rate ratio compared to matched genes = 1.57; 95% CI = 1.14 - 2.17; p = 5.7x10^{-3}; FDR-adjusted p = 0.021) as well as the remaining individuals (rate ratio = 1.36; 95% CI = 1.03 - 1.79; p = 0.03; FDR-adjusted p = 0.083) (rate ratio compared to matched genes = 1.34; 95% CI = 1.03 - 1.79; p = 0.031; FDR-adjusted p = 0.083). Damaging missense variants (MPC ≥ 2) were significantly enriched in females *versus* males without cognitive impairment (rate ratio = 1.56; 95% CI = 1.14 - 2.15; p = 5.5x10^{-3}; FDR-adjusted p = 0.014; Bonferroni-corrected p = 1) (rate ratio compared to matched genes = 1.54; 95% CI = 1.13 - 2.12; p = 6.9x10-3; FDR-adjusted p = 0.024; Bonferroni-corrected p = 1) but not in those with cognitive impairment (rate ratio = 1.11; 95% CI = 0.74 - 1.69, p = 0.61) (rate ratio compared to matched genes = 1.05; 95% CI = 0.70 - 1.60; p = 0.80). These results remained consistent when less restrictive filters were applied (Figure S18A). The effect sizes of damaging protein-truncating and missense DNMs on the liability scale were not significantly different between the sexes (p > 0.05) and were higher than what is expected from matched genes (Figure S18B). Amongst these groups, protein-truncating DNMs in the 1st LOEUF decile increased the liability by 1-1.33 units (0.77-1.11 units relative to matched genes); damaging missense DNMs increased the liability by 0.47-0.73 units (0.3-0.46). When looking at individual cohorts, protein-truncating DNMs in the ASC cohort had a significantly higher effect size in females (see 3.1.1 for a discussion of possible explanations).

Rare inherited variants did not show significant differences in over-transmission of damaging protein-truncating missense variants to autistic males and females (Figure S19A). Notably, the effect size of rare inherited PTVs in the 1st LOEUF decile in those diagnosed autism with cognitive impairment was significantly lower in females (Z = 0.0037; 95% CI =

-0.097 - 1.10; p = 0.31) than males (Z = 0.18; 95% CI = 0.034 - 0.32; p = 0.033; FDR-adjusted p = 0.077) when examining highly LoF-intolerant SFARI genes in the 1st decile ($Z_{Difference}$ = 0.24; 95% CI = 0.072 - 0.40; p = 4.9×10^{-3}; FDR-adjusted p = 0.015; Bonferroni-corrected p = 1), and this was driven by inherited variants in SPARK (Figure S19B). However, the difference was not significant when relaxing the filters to include PTVs in the 1st and 2nd ($Z_{Difference}$ = 0.024; 95%CI = -0.094 - 0.14; p = 0.69); Here, the effect size was 0.16 in males (95% CI = 0.069 - 0.25) and 0.15 in females (95% CI = 0.00063 - 0.29). This favors that the liability of damaging PTVs is not significantly different between males and females.

3.3. Sex-differentially expressed genes in the cortex

3.3.1. Male-biased genes in the adult cortex

De novo mutations:

Genes with male-biased differential expression in the adult cortex (n = 2,852) showed stronger enrichment of damaging protein-truncating DNMs in autistic females compared to autistic males with cognitive impairment (rate ratio ~ 2.5) but there was no significant sex difference on the liability scale. The effect size of damaging protein-truncating DNMs (in 483 genes in the 1st LOEUF decile) was 0.78 in males (95% CI = 0.53 - 1.03; Bonferroni-corrected p = 9.3×10^{-7}) and 0.74 in females (95% CI = 0.55 - 0.93; Bonferroni-corrected p = 2.0×10^{-11}) (difference: p = 0.82). Comparable outcomes (with smaller effect sizes) were observed when including protein-truncating DNMs in the 2nd and the 3rd LOEUF decile in the analysis (443 and 416 genes respectively). Damaging missense DNMs in autistic individuals with cognitive impairment, as well as damaging protein-truncating and missense DNMs in autistic individuals without cognitive impairment or with unknown status, were enriched in both sexes but there was no significant sex difference in rate ratios (observed scale) or Z scores (liability scale) (Figure S20).

Notably, the enrichment of damaging protein-truncating DNMs on the observed scale in this gene set (~2.5 times higher in females than males) was more prominent than amongst SFARI autism-predisposition genes (2 times higher in females than males), although the enrichment in each sex in SFARI genes was stronger, with rate ratio of ~32 in females and ~20 in males (~9 in females and ~8 in males in this gene set). (The difference between the estimates of males and females in each gene set is lower than indicated the female bias because the sex-stratified estimates are from comparisons between autistic children and siblings not diagnosed with autism whereas the sex difference is from direct comparisons between autistic females and males).

Moreover, both the enrichment and liability in male-biased differentially expressed genes were not significantly higher (after multiple testing correction) than what is expected from matched genes with similar coding length, LoF-constraint and brain expression. The effect sizes in SFARI genes, on the other hand, were higher than matched genes on both scales (Figure S18A). This suggests that the enrichment of damaging DNMs in this gene set in autistic males or females (the sex-stratified rate ratios and Z-scores in Figure S20) is a reflection of the enrichment of male-biased, sex-differentially expressed, cortical genes in LoF-constrained and highly-brain-expressed genes more than them conveying a relatively higher risk for autism (like SFARI genes). This enrichment (of LoF-constrained genes amongst male-biased differentially expressed genes in the adult cortex) requires validation and the mechanisms responsible for it are not yet known.

Inherited variants:

Damaging rare inherited protein-truncating and missense variants were not significantly over-transmitted among autistic individuals with cognitive impairment when examined in each sex separately (after correction for multiple testing). The transmission ratio of damaging missense variants (but not damaging PTVs) was significantly lower in females than in males (rate ratio = 0.89; 95% CI = 0.81 - 0.97; FDR-adjusted p = 0.043); yet, there was no significant sex difference on the liability scale (after correction for multiple testing) (Figure S21).

Among autistic individuals without cognitive impairment or with unknown status, there was evidence of significant over-transmission of damaging protein-truncating variants to both females and males (T/U ratios ~ 1 - 1.5; Z-scores ~ 0 - 0.2) and damaging missense variants to males only (T/U ratios ~ 1 - 1.08; Z-scores = 0 - 0.03). There was no significant sex difference on both scales for either variant types after correcting for multiple testing, and the effect sizes were not significantly higher than what is expected for matched genes. These results are presented in Figure S21.

3.3.2. Female-biased genes in the adult cortex

Protein-truncating DNM rates amongst a set of genes with female-biased expression in the adult cortex (n = 2,427) were not significantly different between females and males (Figure S22). Amongst autistic individuals with cognitive impairment, the effect size (on the liability scale) of protein-truncating DNMs in the 1st LOEUF decile (381 genes) was 0.51 in males (95% CI = 0.29 - 0.72; Bonferroni-corrected = 0.0012) and females (95% CI = 0.24 - 0.78; FDR-adjusted p = 0.0044; Bonferroni-corrected p = 0.066); these Z scores were not significantly different between the two sexes (p = 0.96). Similar patterns were seen when

including PTVs in the 2nd (344 genes) and the 3rd LOEUF deciles (319 genes) in the analysis. The enrichment in those without cognitive impairment was less pronounced (smaller effect sizes on the liability scale) than what is seen in those with cognitive impairment, particularly amongst males. The enrichment was not significantly higher than what is expected for a similarly sized gene set matched for coding length, LoF-constraint and brain expression. Damaging missense variants (MPC ≥ 2; MPC ≥ 1) were not enriched in males or females in either of these two phenotypic groups after correction for multiple testing (Figure S22). Similarly, rare inherited protein-truncating and missense variants in this gene set did not convey significant increase in liability (Figure S23).

3.3.3. Sex-biased genes in the fetal cortex

We did not find a significant enrichment of damaging *de novo* and rare inherited protein-truncating variants - after correction for multiple testing - in 305 male-biased genes (39 genes in the 1st LOEUF decile, 49 in the 2nd decile, 42 in the 3rd decile) (see Figure S24 for DNMs and Figure S25 for rare inherited variants) nor 117 genes with female-biased expression in the fetal cortex (8 genes in the 1st LOEUF decile, 15 in the 2nd decile, 11 in the 3rd decile) (see Figures S26 for DNMs and Figure S27 for rare inherited variants), neither with the primary variant filters (PTVs in the 1st LOEUF decile, missense with MPC score ≥ 2) nor relaxed filtering (1st-3rd LOEUF deciles, MPC ≥ 1). Damaging missense DNMs in male-biased genes were significantly more enriched in females *versus* males amongst those without cognitive impairment or with unknown status (Figure S24), and this was driven by SPARK (however, the enrichment in each sex was not significant after correction for multiple testing). The effect sizes on the liability scale (for all the variant classes and phenotypic groups) did not differ significantly from zero (after correction for multiple testing). Some of these analyses were under-powered due to the small number of genes and several tests could not be performed (especially in female-biased genes; Figures S26-S27).

3.3.4. Limitations

It is important to approach the interpretation of all the presented results on sex-differentially-expressed genes in the adult and fetal cortex with caution given the heterogeneity of expression data[8,9] (which we aimed to mitigate by using results from meta-analyzed RNA-seq datasets to define gene sets), the exclusion of genes on chromosome X in our analysis, and the fact that we did not account the extent of sex-differential expression (fold-change) as well as other several sources of bias, e.g., the differences across brain regions and cell types and the time points at which expression is examined. In particular, limiting the analysis to autosomal genes showing sex differential

expression in the cortex has a substantial influence on the results of gene set analysis. For instance, Kissel *et al.*[10] observed that the enrichment of (fetal) female-biased differentially expressed genes in Developmental and Epileptic Encephalopathies (DEE) genes was driven by female-biased genes on chromosome X; Fass *et al.*[11] found a significant overlap between high-confidence autism predisposition genes and female-biased differentially expressed genes in several (adult) brain regions except for the cortex, which showed an enrichment of autism genes in male-bias differentially expressed genes. Interestingly, the enrichment in non-cortical regions was still attributed to the fact that many of the risk loci were found on the X chromosome, which renders them more likely to be upregulated in females. Replication in independent datasets or testing genes that show sex differences in protein expression[12] may add new insights.

4. Liability conveyed by *de novo* and rare variants in autistic individuals with *versus* without cognitive and motor difficulties in SPARK

4.1. Exome-wide effects of *de novo* and rare inherited variants

Here, we describe in more detail the findings presented in Figure 3 (SPARK trios).

4.1.1. Damaging DNMs

Contrary to the observed sex differences seen before stratifying by phenotype (rate $\text{ratio}_{\text{Sex-Difference/SPARK}}$ = 1.27 ; 95% CI = 1.004 - 1.60; p = 0.043; Figure 1B), rates of damaging *de novo* protein-truncating mutations were not significantly different between autistic females and males with motor and cognitive impairment (rate $\text{ratio}_{\text{Sex-Difference}}$ = 1.12; 95% CI = 0.79 - 1.57; p = 0.49) or autistic females and males without these difficulties (rate $\text{ratio}_{\text{Sex-Difference}}$ = 1.25; 95% CI = 0.84 - 1.83; p = 0.23). The effect size attributed to protein-truncating DNMs in autistic individuals with co-occurring motor or cognitive impairment was 0.60 in males (95% CI: 0.46 - 0.74; p = 2.3×10^{-17}; Bonferroni corrected p = 1.3×10^{-15}) and 0.55 in females (95% CI = 0.40 - 0.71; p = 3.2×10^{-12}; Bonferroni corrected p = 1.1×10^{-10}), without a significant difference ($Z_{\text{Sex-Difference}}$ = -0.045; 95% CI = -0.26 - 0.17; p = 0.68). *De novo* protein-truncating mutations increased the liability to autism without cognitive impairment or motor delay similarly in males (Z = 0.32; 95% CI: 0.16 - 0.47; p = 5.9×10^{-5}; Bonferroni corrected p = 0.0032) and females (Z = 0.34; 95% CI: 0.17 - 0.51; p = 6.2×10^{-5}; Bonferroni corrected p = 0.0034) ($Z_{\text{Sex-Difference}}$ = 0.026; 95% CI = -0.20 - 0.25; p =0.82). When comparing autistic individuals with motor delay or cognitive impairment to those without these co-occurring difficulties, these DNMs had similar liabilities (p = 0.66) in males (Z = 0.45; 95% CI = 0.30 - 0.61; p = 7.3×10^{-9}; Bonferroni-corrected p = 4×10^{-7}) and females (Z = 0.39; 95% CI = 0.11 - 0.66; p = 0.0053; FDR-adjusted p = 0.019).

Damaging *de novo* missense mutations increased the liability both in males (Z = 0.17; 95% CI = 0.077 - 0.25; p = 0.00024 ; Bonferroni-corrected p = 0.0016) and in females with motor or cognitive impairment (Z = 0.27; 95% CI = 0.16 - 0.34; p = 2.2×10^{-6}; FDR-adjusted p =0.00012) - without a significant difference (p = 0.16). In those without motor or cognitive impairment, these DNMs significantly increased autism liability in females (Z = 0.016; 95% CI: 0.05 - 0.27 ; p =0.0043; FDR-adjusted p = 0.018) but not in males (Z = 0.029; 95% CI: -0.07 - 0.13 ; p =0.57), though the difference was not significant (p = 0.081).

In direct comparisons between those with and without these difficulties, damaging missense DNMs had an increased liability to co-occurring difficulties in males (Z = 0.23; 95% CI = 0.10 - 0.35; p = 0.00032; Bonferroni-corrected p = 0.017) but the liability was not significantly increased in females (Z = 0.21; 95% CI = -0.012 - 0.43; p = 0.071). The difference was not statistically significant ($Z_{Difference}$ = -0.023; 95% CI = -0.28 - 0.23; p = 0.86).

4.1.2. Inherited damaging variants

In autistic individuals with motor or cognitive impairment (Figure 3C), inherited PTV liability was increased in males (Z = 0.12; 95% CI = 0.076 - 0.16; p = 1.1×10^{-7}; Bonferroni-corrected p = 6×10^{-6}) but not significantly so in females (Z = 0.063; 95% CI = -0.0060 - 0.13 ; p = 0.073), yet the difference was not significant ($Z_{Difference}$ = -0.057; 95% CI = -0.14 - 0.025; p = 0.17). In the other group (without co-occurring difficulties), inherited PTVs had comparable effect sizes (p = 0.54) in males (Z = 0.06; 95% CI: 0.25 - 0.10; p = 0.0049; Bonferroni-corrected p = 0.046) and in females (Z = 0.08; 95% CI: 0.25 - 0.14; p = 0.00085; FDR-adjusted p = 0.019). Notably, ultra-rare inherited variants ascertained in a separate cohort of autistic individuals with sequence data from one parent showed similar patterns in those with motor or cognitive difficulties but not in those without co-occurring difficulties (see 4.3.1 below).

Among those with motor or cognitive impairment (Figure 3C), inherited damaging missense variants conveyed significant liability in males (Z = 0.015; 95% CI = 0.0030 - 0.027; p = 0.015; FDR-adjusted p = 0.047) but not in females (Z = -0.011; 95% CI = -0.30 - 0.0083; p = 0.27). The difference was significant only before correction for multiple testing (Z = -0.026; 95% CI = -0.048 - 0.0032; p = 0.025). The liability attributed to inherited damaging missense variants in females without cognitive impairment was not significantly increased (Z = 0.00035; 95% CI = -0.13 - 0.02). These variants increased the liability in males (Z = 0.01; 95% CI = 0.0006 - 0.02; p = 0.037) but this did not pass FDR-adjustment (p = 0.11). There was no significant difference in the average liability of inherited missense variants between those with and without co-occurring difficulties (p > 0.43).

4.1.3. Imbalance of synonymous DNMs

As presented in the main text (Figure 3B), synonymous DNMs were more prevalent in autistic females without motor or cognitive impairment compared to those with these co-occurring difficulties as well as sex-matched siblings (rate ratio = 1.23; 95% CI = 1.082 - 1.38; p = 0.00099). We examined the burden and liability in ultra-rare DNMs in this sub-cohort (Figure S28) and found better-balanced DNM counts between the probands and siblings, yet not completely controlling the spurious association (rate ratio = 1.22; 95% CI =

1.038 - 1.43; p = 0.015), which is likely to be an artifact of the diverse ancestries included in the sample.

We then examined whether limiting the analysis to a stringently ancestry-matched set of 868 autistic females and 2,235 siblings could resolve this issue (see section 3.4 of the Supplemental Methods). This ancestry matching alone resulted in better-balanced DNM counts between the probands and siblings with some residual imbalance (rate ratio = 1.17; 95% CI = 1 - 1.37; p = 0.047). Next, we combined the two filtering strategies, i.e. by evaluating ultra-rare DNMs in samples well-matched on genetic ancestry. Here, synonymous DNMs were not associated with autism (rate Ratio = 1.09; 95% CI = 0.88 - 1.35; p = 0.42).

Lastly, we turned to protein-truncating DNMs to see whether the estimated enrichment in protein-truncating DNMs differed with more stringent frequency and ancestry matching. The rare DNM rate ratio was 2.65 for rare DNMs in the complete cohort (95% CI = 1.61 - 4.37; p = 6.2×10^{-5}) *versus* 2.68 in ancestry-matched samples (95% CI = 1.47 - 4.9; p = 0.00067). The ultra-rare DNM rate ratio was 2.66 in the complete cohort (95% CI = 1.6 - 4.42; p = 8.4×10^{-5}) *versus* 2.57 in ancestry-matched samples (95% CI = 1.4 - 4.7; p = 0.0018). Thus, the estimates of protein-truncating DNM enrichment obtained when examining the full cohort were robust to these differences in genetic ancestry and were slightly more precise.

4.2. *De novo* and rare variants in high-confidence and syndromic SFARI genes

4.2.1. Observed effect sizes

Among autistic individuals with motor and cognitive impairment (Figure S29A), protein-truncating DNMs were enriched in autistic males (rate ratio = 23.72; 95% CI = 7.78 - 117.82; p = 6.3×10^{-18}; Bonferroni-corrected p = 9.1×10^{-16}) and females (rate ratio = 22.04; 95% CI = 8.53 - 72.47; p = 2.4×10^{-16}; Bonferroni-corrected p = 3.4×10^{-14}) but not significantly different between the two sexes (risk ratio = 1.48, 95% CI = 0.94 - 2.28; p = 0.079). Amongst those without these co-occurring conditions, protein-truncating DNMs were also significantly enriched in autistic males (rate ratio = 9.43; 95% CI = 3.05 - 47.25; p = 4.4×10^{-7}; Bonferroni-corrected p = 6.3×10^{-5}) and females (rate ratio = 7.56; 95% CI = 2.65 - 26.38; p = 1.5×10^{-5}; Bonferroni-corrected p = 0.0021) without a significant difference between the sexes (risk ratio = 1.28, 95% CI = 0.68 - 2.27; p = 0.36). As expected for these known developmental genes, protein-truncating DNMs were substantially enriched in those with motor or cognitive impairment *versus* those without co-occurring difficulties, in males (rate

ratio = 2.51; 95% CI = 1.73 - 3.68; p = $6.6x10^{-7}$; Bonferroni-corrected p = $9.5x10^{-5}$) and females (rate ratio = 2.92; 95% CI = 1.55 - 5.69; p = $3.4x10^{-4}$; Bonferroni-corrected p = 0.048). The enrichment in missense DNM enrichment in SFARI genes was less prominent and there were no significant sex differences in rate ratios after accounting for multiple testing; these DNMs were also more enriched in those with motor or cognitive difficulties *versus* those without these co-occurring difficulties. The observed sex differences in DNM risk ratios did not translate to a significant sex-bias on the liability scale (Figure S29A). Inherited variants did not show significant differences in over-transmission rates and liability compared to matched genes (Figure S29B).

4.2.2. Effect sizes relative to matched genes

Both the rate ratio in autistic individuals *versus* siblings and the rate ratio in autistic females *versus* autistic males (sex difference) were significantly higher than what is expected from matched genes, i.e, significantly higher than the rate ratio calculated from randomly selected genes matched on LoF-constraint, sex-averaged brain expression and coding length profiles. In the full cohort analysis (not split by coexisting motor or cognitive impairment), this was evident in protein-truncating (driven by autistic probands who have motor/cognitive impairment) as well as missense DNMs (driven by those without motor/cognitive impairment). Notably, autistic females and males in the 'motor and cognitive impairment' group do not show a significant sex difference in their DNM rates *per se*, i.e. relative to what is expected from the sample size of the cohort, yet the sex difference in DNM rates is significantly higher than what is expected from matched genes. In the same vein, damaging missense DNMs in the 'autism without motor/cognitive impairment' had significantly higher female-bias when compared to matched genes but not when tested relative to the sample size. These findings are presented in Figure S29.

On the liability scale, protein-truncating DNMs in SFARI genes increased the liability to autism with cognitive and motor impairment by 0.99 units more than a matched gene set in males (95% CI = 0.64 - 1.34; p = $2.6x10^{-8}$; Bonferroni-corrected p = $3.7x10^{-6}$) and 0.86 units more that matched genes in females (95% CI = 0.59 - 1.14; p = $1.1x10^{-9}$; Bonferroni-corrected p = $1.6x10^{-7}$), without a significant sex difference (p= 0.58). In the 'autism without cognitive & motor impairment' group (relative to matched genes), the sex difference between males (Z = 0.89; 95% CI = 0.49 - 1.30; p = $1.8x10^{-5}$; Bonferroni-corrected p = 0.0025) and females (Z = 0.52; 95% CI = 1.7 - 0.86 ; p = 0.0032; FDR-adjusted p= 0.017; Bonferroni-corrected p = 0.46) was also not significant (p = 0.17). Despite this sex bias in the enrichment relative to matched genes, the 'excess' autism liability conferred by damaging variants in SFARI genes on top of what is expected from matched genes was not

significantly different between males and females (i.e. for these comparisons against matched genes, the sex difference on the observed scale did not translate into differences on the liability scale).

4.2.3. Permutation analysis

The lack of significant sex differences in DNM rates in SPARK is consistent before (Figure 2) and after (Figure 3) considering motor difficulties/removing those with missing information. As mentioned before (section 2.1), the remaining autosomal genes did not show significant sex-differences in DNM rate ratios (Figure S10). Since SFARI genes are known to cause multiple developmental difficulties with high penetrance including cognitive impairment and motor delays, this suggests that the sex difference on the observed scale may be driven by differences in the proportions of autistic females and males with these co-occurring difficulties (40% in females *versus* 35% in males; relative risk = 1.16). Indeed, there is no significant sex bias when the analysis is performed separately in those with/without motor or cognitive impairment as we show here. However, it is possible that the smaller sample size in these stratified analyses reduced the power. Moreover, we do not see similar results in ASC (Figure 2B). We ran a permutation analysis by shuffling the sex labels within the four phenotypic groups (i.e. sampling without replacement within these groups: autism with motor or cognitive impairment, without coexisting difficulties, unknown status, siblings) before examining the full cohort again, so that the proportion of individuals with coexisting conditions is kept constant, then calculated the sex-stratified variant rates. We repeated this procedure 1,000 times and took the average rate of synonymous & damaging DNMs, then examined the rate ratios between the probands and siblings. We did not see any significant sex difference when we performed this analysis for all protein-coding genes (exome-wide), SFARI high-confidence and syndromic autism genes or the remaining genes (Figure S30). This suggested that the difference in the proportion of co-occurring difficulties was not the main determinant of the difference in variant rates - congruent with a recent ASC analysis (using regression to evaluate the odds of harboring deleterious DNMs that are associated with sex and cognitive impairment status) which did not find evidence for a significant interaction between sex and cognitive impairment.[13]

4.3. Exome-wide burden of ultra-rare variants in the remaining individuals

4.3.1. Over-transmission in children with sequence data from one parent

As shown in Figure S31, inherited PTVs conferred significant liability in males with motor or cognitive co-occurring (Z = 0.13; 95% CI = 0.057 - 0.20; p = 0.00052; Bonferroni-corrected p = 0.14) but not females (Z = 0.083; 95% CI = -0.048 - 0.21; p = 0.21), yet without a significant sex difference (p = 0.54), whereas the liability in the other group was not significantly increased from the population mean in both sexes (p > 0.11). The liability attributed to inherited PTVs did not differ significantly between those with and without co-occurring difficulties (p > 0.31). Ultra-rare damaging missense variants in SPARK individuals with sequence data from one parent did not convey significant liability.

4.3.2. Enrichment of ultra-rare variants in the remaining individuals without parental sequence data

There were no significant sex differences in the enrichment and liability damaging of protein-truncating and missense ultra-rare variants among the remaining probands and siblings from SPARK (Figure S32). The effect size of ultra-rare protein-truncating variants (adjusted for synonymous variants) was similar in males (Z = 0.27; 95% CI = 0.19 - 0.34; p = $1.4 \times 10^{-11;}$ Bonferroni-corrected p = 6.4×10^{-10}) and females with motor and cognitive difficulties (Z = 0.22; 95% CI = 0.15 - 0.30; p = 2.2×10^{-8}; Bonferroni-corrected p = 1.0×10^{-7}). Damaging missense variants had an effect sizes of 0.039 in males (95% CI = 0.0096 - 0.068; p = 0.0094; FDR-adjusted p = 0.033; Bonferroni-corrected p = 0.42) and 0.036 in females with cognitive impairment or motor delay (95% CI = 0.0020 - 0.069; p = 0.038; FDR-adjusted p = 0.095).

Among those without motor and cognitive difficulties, PTVs had an effect size of 0.16 in males (95% CI = 0.074 - 0.24; p = 0.00021; Bonferroni-corrected p = 0.0096) compared to 0.040 in females (95% CI = -0.056 - 0.42; p = 0.42) although the difference was not statistically significant (p = 0.068). There was no significant enrichment in damaging missense variants in autistic individuals without these co-occurring conditions. When the two groups were compared (with *versus* without motor or cognitive impairment), the effect size of PTVs was 0.19 in males (95% CI = 0.089 - 0.29; p = 0.00023; Bonferroni-corrected p = 0.010) and 0.35 in females (95% CI = 0.17 - 0.53; p = 0.00017; Bonferroni-corrected p = 0.0079), and it was not significantly different (p = 0.14). Missense variants had a slightly higher liability to autism with (vs. without) motor or cognitive impairment in males (Z = 0.048;

95% CI = 0.0046 - 0.092; p = 0.03) but the liability did not differ between the two groups after correction for multiple testing (FDR-adjusted p = 0.08).

4.4. Effect sizes of *de novo* mutations in an NDD cohort

To test whether the genetic predisposition conveyed by damaging DNMs is sufficient to reach the threshold to have developmental impairments (i.e. 'highly' or 'fully penetrant') and not significantly different between the sexes, we estimated their liability in 31,565 trios diagnosed with neurodevelopmental disorders (NDDs) not specifically ascertained for autism (17,698 males and 13,867 females, sex ratio ~ 1.3). These DNMs were curated by Zhou and colleagues[2] from several published cohorts of trio-sequenced children with developmental disorders[14] (17,422 males and 13,636 females), developmental and epileptic encephalopathies[15] (210 males and 146 females) or severe intellectual disability[16,17] (66 male and 85 females).

The observed variant counts were compared to those expected from a mutational model (see section 5.1 of the Supplemental Methods). To estimate liability, we used a prevalence of 2% in males and 1.5% in females (sex ratio ~ 1.3). In addition to SFARI genes, we also tested another set of genes associated with developmental disorders with high-confidence[18] (DDG2P v4.6). Most of the autosomal SFARI genes are also DDG2P genes (281/354, 240 mono-allelic and 41 bi-allelic genes). Specifically, we tested the enrichment and liability in following sets:

- High-confidence autism genes: 354 SFARI genes (218 in the 1st LOEUF decile).
- High-confidence NDD genes: 845 monoallelic DDG2P genes (384 in the 1st decile).
- High-confidence NDD genes with high autism penetrance: 240 genes shared between SFARI & monoallelic DDG2P sets (173 in the 1st decile).
- High-confidence NDD genes with low autism penetrance: 605 DDG2P genes that are not amongst SFARI genes (209 in the 1st decile).
- Low confidence/penetrance NDD genes: 15,032 autosomal protein-coding genes after removing SFARI & mono-allelic DDG2P genes (1,315 in the 1st decile).

On the observed scale, the higher enrichment in damaging DNMs in females *versus* males was driven by known autism and NDD genes (Figure S33B). SFARI genes showed significant female bias in damaging protein-truncating in the 1st LOEUF decile (rate ratio = 1.17; 95% CI = 1.07 - 1.28; Bonferroni-corrected p = 0.017) and missense DNMs with MPC score ≥ 2 (rate ratio = 1.12; 95% CI = 1.02 - 1.24; FDR-adjusted p = 0.032). DDG2P genes showed similar female bias (protein-truncating DNMs: rate ratio = 1.15; 95% CI = 1.06 -

1.24; 0.028) (missense DNMs: rate ratio = 1.15; 95% CI = 1.06 - 1.24; Bonferroni-corrected $p = 0.033$).

In DDG2P genes, the enrichment in protein-truncating DNMs was driven by a subset overlapping SFARI genes (rate ratio = 1.18; 95% CI = 1.08 - 1.28; Bonferroni-corrected $p = 0.012$) rather than the remaining DDG2P genes (rate ratio = 1.04; 95% CI = 0.88 - 1.23; $p = 0.67$). The enrichment in damaging missense DNMs, however, was nominally higher in DDG2P genes not in SFARI genes (rate ratio = 1.17; 95% CI = 1.03 - 1.33; FDR-adjusted $p = 0.027$) than those overlapping SFARI genes (rate ratio = 1.13; 95% CI = 1.02 - 1.25; FDR-adjusted $p = 0.029$). This may indicate that there are additional genes with female bias driven by missense variants that are not (yet) confidently associated with autism (e.g., bias in gene discovery towards pLoF variants) or that have limited/low penetrance for autism. Notably, the burden of protein-truncating DNMs amongst the remaining autosomal genes was significant higher in males versus females when SFARI & DDG2P genes were removed (rate ratio = 1.26; 95% CI = 1.06 - 1.49; FDR-adjusted $p = 0.014$). It remains to be clarified how this relates to the phenotypic spectrum of NDDs.

In all these gene sets, the difference in liability between the two sexes was not significant (Figure S33C). Damaging protein-truncating DNMs in SFARI genes increased NDD risk by 1.89 units in males (95% CI = 1.43 - 2.35; Bonferroni-corrected $p < 1x10^{-20}$) and 1.94 in females (95% CI = 1.48 - 2.41; Bonferroni-corrected $p < 1x10^{-20}$). These effect sizes overlapped the threshold (~2 in males, ~2.2 in females) and were nominally higher than those estimated in DDG2P genes (Z_{Males} = 1.77; 95% CI = 1.35 - 2.20; Bonferroni-corrected $p < 1x10^{-20}$) ($Z_{Females}$ = 1.8; 95% CI = 1.37 - 2.24; Bonferroni-corrected $p < 1x10^{-20}$). This indicates that SFARI genes are nearly fully penetrant for NDDs. Amongst DDG2P genes, the liability was substantially higher amongst genes that are also SFARI genes (~2 units) compared to other DDG2P genes that are not (~1.3 units), again highlighting the high penetrance of SFARI genes for developmental phenotypes.

5. Altering the assumptions of the liability model

5.1. Lower threshold in females

In the different-thresholds model, the sex difference in autism prevalence is attributed to females having a higher threshold than males, with the assumption that the underlying liability distribution is the same in both sexes (Figure S5B). Estimating the effect sizes under this model requires assumptions about the liability threshold in males and females, which are calculated from estimated population prevalences in males and females using the inverse normal function. In the primary analysis, we used a prevalence estimate of ~2.5% in males and assuming the sex ratio is 4:1 we estimated the prevalence in females to be ~ 0.63%. Accordingly, we set the threshold in our primary analyses to ~1.96 in males and ~2.5 in females. However, the prevalence estimates in females are less certain (due to e.g., relative rarity or under-diagnosis in females) and the sex ratio could be overestimated.[19] Therefore, we tested two alternative models using ratios of 3:1 and 2:1. We did not see evidence of substantial differences in rare variant effect sizes on the liability scale after correction for multiple testing (Figure S34).

5.2. Higher variance in males

The true population parameters of the liability distribution are unknown. Sex differences in autism prevalence can alternatively be attributed to the two sexes having differing variances rather than differing thresholds (Figure S5B). We therefore tested an alternative model in which we assumed that the normally distributed liability in the general population (50% females) is the outcome of two underlying liability distributions in males and females with equal means in both sexes but with higher variance in males (standard deviation = 1.2) than females (standard deviation = 0.75) (variance ~2.5 times higher in males). We chose these two parameters so that the population variance is approximately equal to 1. (See section 6.2 of the Supplemental Methods for additional details).

We found that damaging protein-truncating and missense de novo mutations did not show significant differences in their effect sizes between males and females ($p > 0.05$). In contrast, inherited damaging protein-truncating variants had higher effect sizes in males ($Z = 0.077$; 95% CI = 0.051 - 0.10; $p = 1.2\times10^{-8}$; Bonferroni-corrected $p = 6.3\times10^{-7}$) than females ($Z = 0.032$; 95% CI = 0.0070 - 0.057; $p = 0.0017$; FDR-adjusted $p = 0.0046$; Bonferroni-corrected $p = 0.092$) and the difference was significant after multiple testing correction ($Z_{Difference} = 0.044$; 95% CI = 0.0088 - 0.081; $p = 0.015$; FDR-adjusted $p = 0.035$;

Bonferroni-corrected $p = 0.80$). Inherited damaging missense variants also had significantly higher effect sizes ($Z_{Difference} = 0.015$; 95% CI = 0.0037 - 0.027; $p = 0.0099$; FDR-adjusted $p = 0.025$; Bonferroni-corrected $p = 0.53$) in males ($Z = 0.019$; 95% CI = 0.012 - 0.026; $p = 4.5 \times 10^{-7}$; Bonferroni-corrected $p = 2.4 \times 10^{-5}$) than females ($Z = 0.0037$; 95% CI = -0.0055 - 0.013; $p = 0.65$). These comparisons are shown in Figure S35.

Supplemental Methods

1. SPARK dataset preparation

1.1. Exome Sequencing and phenotypic data

We downloaded the Simons Foundation Powering Autism Research for Knowledge (SPARK) integrated whole-exome sequencing data[2] release version 2 through Globus.[20] This release (jointly called VCF v2.2023_01, Deep Variant/GLNexus) encompassed 106,744 samples from five sequencing batches (WES1: 27,177 samples, WES2: 15,671, WES3: 16,476, WES4: 11,340, WES5: 36,080), sequenced using two capture systems (WES1-4: IDT, WES5: TWIST). The dataset included 44,304 autistic individuals (33,335 males and 10,969 females) and 62,440 individuals without an autism diagnosis (24,783 males and 37,657 females), including 27,775 father-child-pairs (19,977 autistic individuals) and 46,343 mother-child pairs (34,011 autistic individuals). These formed 25,386 trios (18,172 autistic individuals), 23,346 samples with one sequenced parent (17,644 autistic individuals), and 58,012 samples without parental WES (8,488 autistic individuals). The phenotypic data for the autistic individuals were downloaded from SFARI's Genotypes and Phenotypes in Families (GPF) database.[21] These phenotypic data were available for 40,346 autistic individuals with WES data.

1.2. Variant annotation

The provided dataset included 10,202,547 sites (11,236,834 after splitting multiallelic sites). These sites were annotated using Variant Effect Predictor[22] (VEP) Ensembl release 108. The following annotations were added: consequences on Ensembl genes and transcripts, exon/intron numbers, allele frequencies based on gnomAD exomes (r2.1.1) and gnomAD genomes (r3), MPC scores (v2, gnomAD), Loftee predictions, NMD plugin predictions.[23] The consequences were annotated on 'MANE Plus Clinical' transcripts (Matched Annotation between NCBI and Ensembl v1). For variants with consequences on more than one MANE transcript, one consequence was prioritized based on the severity of the predicted effects or gene constraint. We produced a working dataset in which we split multiallelic sites, set any variants with depth zero to missing, and filtered for variants that were in the exonic regions or the adjacent splice regions of MANE/Clinical transcripts.

1.3. Ancestry inference

SPARK provided ancestry labels based on the 1000 Genomes super-populations (Africans [AFR], Admixed Americans [AMR], East Asians [EAS], Europeans [EUR], and South Asians [SAS]), along with subpopulation labels, and probabilities for being assigned to these sub-populations. A group of 2,783 samples were labeled as having an unknown super-population (Table S3). To confirm these labels and to classify the samples in the 'Unknown' group to the nearest population when possible, we projected SPARK iWES2 samples onto a principal components (PCs) space based on 2,536 samples from the 1000 Genomes Project[24] calculated using PLINK.[25] PC analysis of the 1000 Genomes reference data (10 PCs) leveraged 8,718 pruned variants with allele frequency > 1% that are present in at least one sample in SPARK samples, limited to exonic regions of protein coding transcripts described above. SPARK samples were then projected into the 1,000 Genomes PC space. The clustering of these samples was contrasted with the pre-defined ancestry labels. We reclassified the samples where the superpopulation was labeled as 'unknown' to one of the 1,000 Genomes superpopulations if these had a probability exceeding 0.8 of belonging to that group (calculated as the sum of probabilities of belonging to the sub-populations under that group, provided by SPARK), or clustered with that group on each of the first four PCs. Last, we defined a group of admixed individuals that did not cluster closely with their corresponding groups on PC1-4 (Table S4 and Figure S2).

1.4. Sex inference

To infer the ploidy of sex chromosomes from sequencing data, we evaluated the depth and genotypes in the hemizygous regions of chrX and chrY, namely, (1) the read depth in chrX normalized by the median chrX depth in samples labeled as males, (2) the read depth in chrY normalized by the median chrY depth in samples labeled as males, (3) the fraction of missing calls on chrY, and (4) the F statistic (the difference between the expected and observed heterozygosity in the hemizygous region of chrX; PLINK). Male sex was inferred for samples with normalized chrY depth between 0.5 and 2, fraction of missing genotypes in chrY < 50%, normalized chrX depth < 2, and F statistic > 0.8. Female sex was inferred for samples with normalized chrX depth between 1 and 3, F statistic > -0.6 and < 0.6, normalized chrY depth < 0.15, and fraction of missing genotypes on chrY > 50%. 122 male and 88 female samples were classified as having ambiguous inferred sex or having a mismatch between reported and inferred sex, and were removed from the burden analyses.

1.5. Relatedness inference

The provided meta-data indicated the presence of 27,775 father-child-pairs and 46,343 mother-child pairs (25,386 complete trios). To verify the parental relationships, kinship was estimated using PLINK based on KING's robust algorithm.[26] We required the parent-child pairs to have a kinship estimate between 0.3 and 0.1 and a proportion of SNPs with Identity By State (IBS) sharing < $5x10^{-5}$. Ten parent-child pairs could not be verified based on kinship. A set of maximally unrelated probands (N=39,020) was selected by incrementally removing the samples with the highest number of related autistic individuals (kinship > 0.0884) while prioritizing those with sequenced parents and females in that order. Ties were resolved by retaining the sample with the older identifier. Similar sets of unrelated individuals were defined for the siblings (N=15,056) and parents (N=47,525). We estimated the allele frequencies, Hardy Weinberg Equilibrium *p*-values, and Excess Heterozygosity *p*-values separately in these three sets of unrelated samples (per population) and used the largest *p*-value (i.e. the least significant) as an input for variant quality control (QC).

1.6. Variant QC

A Random Forest was trained to identify low quality variant call sites. It was applied separately on SNVs and indels and the training sites were selected from variants heterozygous in one or more samples. Before running the variant QC, we applied basic genotype filtering by setting the genotypes that had any of the following to missing: (1) depth equal to zero, (2) genotype quality (GQ) equal to zero, (3) a conflict between the called genotype and the phred-scaled genotype likelihood, or (4) a *p*-value from a binomial test for allele balance less that $1x10^{-10}$ (for heterozygous calls). Negative training sites were then defined as those that met one of the following criteria, excluding multi-allelic sites: (1) more than 50% of the samples with non-reference (heterozygous or homozygous) genotypes that had GQ < 20, (2) more than 50% of samples with a heterozygous genotype had variant allele fraction (VAF) below 0.2, or (3) the Hardy-Weinberg Equilibrium test *p*-value (calculated for separately for unrelated parents, siblings and probands per population, taking the largest estimate across all populations) was lower than 10^{-9}. The positive training sites were the high confidence sites from the Broad bundle resource used in the Genome Analysis Toolkit (GATK) variant calling pipeline[27] (SNVs: Omni, Axiom, HapMap; indels: Axiom, Mills). Since these sites are enriched for common variants, we annotated an equal number of randomly selected high confidence transmitted singleton variants (seen in a single child-parent pair, with Allele Quality > 35) as positive training sites. To balance the number of positive (SNVs: 388,268; indels: 17,732) and negative (SNVs: 98,676; indels: 3,961) training

sites, the positive training set was down-sampled to match the size of the negative training set, resulting in a balanced set of 205,274 variants (SNV = 197,352, 2.8% of all SNVs, INDEL=7,922, 2.3% of all indels). The remaining sites were annotated as test sites (SNVs= 6,731,744, indels= 310,362).

We used the `ranger` package[28] in `R 4.1.0` to train random forest models on SNVs and indels separately, with 500 trees and probabilities as an output. The features used to train the random forest, and their relative importance, are depicted in Figure S3A. The training sites did not include any missing values in these features, and missing values among the test sites were set to the median of the respective feature across all sites of similar type (SNV or INDEL). The random forest model was then applied on all training and test sites to obtain probabilistic scores (range 0-1) indicating the probability of being a truly variable site. These scores were scaled to the range 0-100. The performance of the random forest was evaluated using precision and recall against the training sites using the `caret` package[29] in `R`. We then examined the fraction of retained sites from the total dataset and the transmission ratio of synonymous variants with incremental random forest cut-offs. Random Forest score cut-offs of 97 for SNVs (recall = 0.97, precision = 1) and 92 for indels (recall = 0.975, precision = 1.0) were selected, and sites with scores higher than these were retained. The transmitted/untransmitted ratio of autosomal synonymous variants was 0.5 (Figure S3B). Next, the variants were filtered to those with a minimum genotyping rate (per sequencing batch; across five sequencing batches) exceeding 95%. After this QC, 6,345,636 out of 7,273,162 sites were retained (12.8% filtered). These variants spanned 18,959 MANE-Plus transcripts (18,902 protein coding genes; including 57 genes with two transcripts).

1.7. Genotype filtering

We applied genotype filters based on genotype quality, allele depth, variant allele fraction, by setting the genotypes to missing if they were not consistent with the observed phred-scaled genotype likelihoods (PLs), had GQ < 10, had DP < 10, were reference genotypes with VAF > 0.25, were homozygous with VAF < 0.75, or were heterozygous with VAF < 0.2 or > 0.8.

1.8. Sample QC

We calculated the following sample-level metrics: total number of SNVs, total number of indels, total number of private variants (seen in one family), transition-transversion ratio, insertion-deletion ratio, and heterozygous-homozygous variants

ratio. We regressed these on the population label, four principal components and the number of sequenced samples per family, to account for the population structure and the differences in variant-calling sensitivity in related individuals. We filtered all samples that were more than six standard deviations from the mean of the residuals for any metric, removing 275 samples (AFR: 180, EUR: 55, AMR: 22, EAS: 3, SAS: 6, Unknown/Admixed: 9). Parents with autism and parents and siblings with motor delay or cognitive impairment were filtered (see 2.1). Subsequently, sets of maximally unrelated probands and siblings were selected. Note that this unrelated dataset of probands and siblings was used for the formal analysis of rare variant enrichment, and is prepared independently from the unrelated set presented in section 1.5, which was used to estimate the allele frequency for variant QC. Table S5 shows the sample size per population group after the sample QC.

2. Co-existing difficulties

2.1. Cognitive impairment in the ASC and SPARK

The ASC studies considered autistic individuals to have co-existing cognitive impairment/intellectual disability if the subject had a full-scale IQ test score < 70, if they were administered but could not complete an IQ test, or if the subject had a human phenotype ontology (HPO) term or International Classification of Diseases (ICD) code indicating intellectual disability or mental retardation.[13,30] The remaining individuals without cognitive impairment and those without information on cognitive impairment status were grouped together. To have a comparable set from SPARK for the purpose of a meta-analysis with ASC, we split autistic individuals in SPARK into those with *versus* another group combining those without cognitive impairment and those with an unknown cognitive impairment status. The phenotypic data for SPARK individuals included whether they had a reported professional diagnosis of cognitive impairment as well as binned IQ test scores. The ‘autism with cognitive impairment group included those with one of the following phenotypic entries: ‘Cognitive_impairment_latest’ = “True”, ‘dev_id’ = 1, or ‘reported_cog_test_score’ = ‘24_below’, ‘25_39’, ‘40_54’, or ‘55_69’.

2.2. Motor delay and cognitive impairment in SPARK

We used the following phenotypic data fields to stratify the samples based on the presence of a motor developmental disorder, cognitive impairment or an intellectual disability diagnosis:

- ‘cognitive_impairment_at_enrollment’ and ‘cognitive_impairment_latest’: provided information on cognitive impairment diagnosis (identical values). Probands with a diagnosis were coded as “True” and those without diagnosis were coded as “False”. Missing values were coded as “-”.
- 'dev_id' and 'dev_motor': provided information on cognitive impairment (Intellectual disability, cognitive impairment, global developmental delay, or borderline intellectual functioning, reported professional diagnosis) and motor development (Motor delay, e.g., delay in walking, or developmental coordination disorder; reported professional diagnosis). Diagnoses were coded as “1”. Typical development and missing values were coded as “-”.
- ‘cog_test_score’ and the ‘reported_cog_test_score’: provided binned full-scale IQ measurements (identical values). Missing values were coded as “-”.

We classified the autistic individuals in three phenotypic groups:

1. Autism with motor delay or cognitive impairment:
   ‘Cognitive_impairment_at_enrollment’ = “True, *or*
   ‘Cognitive_impairment_latest’ = “True”, *or*
   ‘dev_motor’ = 1, *or*
   ‘dev_id’ = 1, *or*
   ‘Cog_test_score’ = ‘24_below’,‘25_39’, ‘40_54’, ‘55_69’, or ‘70_79’, *or*
   ‘reported_cog_test_score’ = ‘24_below’,‘25_39’, ‘40_54’, ‘55_69’, or ‘70_79’.
2. Autism without motor or cognitive impairment:
   ‘Cognitive_impairment_at_enrollment’ = “False”, *and*
   ‘Cognitive_impairment_latest’ = “False”, *and*
   IQ bin equivalent to an IQ >= 80 or missing IQ data, *and*
   missing values in the ‘dev_motor’ field, *and*
   missing values in the ‘dev_id’ field.

The remaining probands with missing values in all fields were considered unclassified.

3. *De novo* and rare inherited variants in trios

As introduced in the main text, *de novo* mutations (DNMs) were studied in a total of 30,274 trios from the two cohorts by comparing DNM rates in 21,501 autistics (ASC: 8,028; SPARK: 13,473) to those seen in 8,773 siblings (ASC: 2,460; SPARK: 6,763). Over-transmission analysis of rare inherited variants was performed by evaluating the counts

of rare parental alleles transmitted to 21,043 autistic individuals (ASC: 7,570; SPARK: 13,473) *versus* untransmitted alleles. This section outlines the curation of DNMs and inherited alleles in each cohort.

3.1. ASC

DNMs in this dataset were obtained from a previous ASC study[13] (see 'Methods: ASC cohort'). These were curated in 9,929 trios (7,570 autistics, 2,359 siblings) as described in detail in the Supplementary Note of Ref.[13] Briefly, the `de_novo()` function from `Hail 0.2` python library[31] was used to identify candidate DNMs, with priors from population allele frequencies. These candidates were then filtered for rare alleles with internal (within ASC dataset) and external (gnomAD) allele frequencies < 0.1%, with additional filtering on genotype depth (allele balance and parent/child depth ratio), variant quality ('ExcessHet' filter and GATK variant quality score log-odds), as well as the number of candidate DNMs per sample. Additional published DNMs from 559 trios (458 probands and 101 siblings) were included, bringing the total sample size to 10,488 trios (8,028 autistics, 2,460 siblings). Transmitted and untransmitted rare parental alleles were counted in 7,570 trios (autistic probands) with available exome data.

3.2. SPARK

Following the variant, genotype and sample QC described in section 1 above (see 'SPARK dataset preparation'), we evaluated the genotypes of 20,236 trios (13,473 autistics and 6,763 siblings) for potential DNMs (see counts for probands and siblings in Table S1). We first filtered for candidate DNMs based on the parental genotypes and used the 'trio-dnm2' plugin from bcftools[32] 1.17 to estimate the error probability of *de novo* inheritance based on allele depth (adDNM) and genotype likelihoods (plDNM). Then for each candidate DNM, we transformed the probabilities of it being a *de novo* mutation based on these two methods to a phred-scaled error probability ($-10*\log_{10}$(minimum(plDNM,adDNM)). We then defined putative DNMs as those with phred-scaled error probability ≤ 60 having internal & gnomAD MAF < 0.1%, with an Allele Quality (AQ) score ≥ 30, and that had GQ ≥ 30 in all members of the trio. Finally, we selected one DNM per gene (worst consequence) per individual. This resulted in a dataset of 23,332 DNMs in 13,664 individuals (68% trios with DNMs, average rate of 1.15 DNMs per individual). Between 20% and 22% of the probands and siblings carried rare synonymous DNMs, compared to 23%-25% in the previous analysis of SPARK[13] (WES1). Another previous analysis of SPARK WES1 found ~ 0.20 - 0.23 synonymous DNMs per offspring,[1] suggesting that our DNM callset was of similar quality to

the published ones. To ensure that the sensitivity to identify DNMs was not biased by sample sex, we calculated the odds ratio of carrying a rare *de novo* synonymous (all autosomal genes) or protein-truncating DNM (most-constrained LOEUF decile) among male *versus* female siblings, and found that it did not differ significantly from one.

We also evaluated the transmission of rare variants (MAF < 0.1% in SPARK parents and gnomAD) in the same set of trio-sequenced autistic individuals. Similar to DNMs, we considered variants remaining after basic variant, genotype and sample QC. In instances where a single gene had multiple variants, the variant with the worst consequence was retained. We then filtered for heterozygous variants seen in one parent that had a GQ > 25 in all three samples in the trio to ensure the transmission ratio of synonymous variants was not significantly different from one.

3.3. Additional filtering of *de novo* mutations in SPARK

De novo mutations, especially damaging ones, are typically ultra-rare, usually seen in a few individuals or not seen at all in the general population. DNM calling from genotypes (see '3.2 SPARK' above) does not leverage allele frequencies as priors, and is best coupled with stringent allele frequency filtering by removing DNMs seen in more than a few individuals in the dataset or in the general population. Given that the ASC DNM dataset was prepared using a different pipeline and filtered for rare alleles (MAF < 0.1%), we adopted a similar cutoff in SPARK to have comparable call sets, thus facilitating fixed-effect meta-analysis of risk ratios. We then performed an additional analysis of ultra-rare DNMs in SPARK to ensure that the conclusions are the same in a call-set of high-confidence DNMs that favors precision over sensitivity. To define ultra-rare DNMs, we further dropped DNMs if the individual had more than one DNM in the same gene, those seen as *de novo* in more than 3 individuals in SPARK, DNMs with MAF ≥ 0.005% in SPARK parents or gnomAD, and DNMs seen in gnomAD and two of the three SPARK cohorts of unrelated individuals used for estimating allele frequency (probands, siblings, parents). This final dataset included 15,072 ultra-rare DNMs, with an average rate of 0.74 ultra-rare DNMs per sample.

3.4. Matching samples on principal components

As presented in the main text (Figure 2), *de novo* synonymous mutations showed a spurious association with autism when comparing autistic females without motor or cognitive impairment to sex-matched probands with motor or cognitive impairment or when compared to female siblings. This likely reflects the greater diversity among autistic females without motor or cognitive impairment (69% of samples with European genetic ancestry) relative to

the other two groups (75% of samples with European genetic ancestry in each; see Table S4). We performed an additional sensitivity analysis of rare and ultra-rare *de novo mutation* enrichment in a stringently matched set of samples to ensure that the results obtained for protein-truncating DNMs were not biased. Starting with 1,008 autistic females without cognitive impairment (`P`) and 2,596 female siblings (`S`) of European genetic ancestry (Table S5), we calculated the distance between all pairs of probands and siblings in the first four principal components (see section 1.3). Specifically, we subtracted the first four PCA eigenvectors for each proband `p` in `P` from the eigenvectors of each sibling `s` in `S`, then used the `norm(type="2")` function in `R` to calculate a Euclidean-type spectral norm representing the distance between the two samples in the PC space. We then took the minimum value for each sample in `P` and `S`, which represents the nearest neighboring sample from the opposite cohort. Samples with large values don't have any neighboring samples from the other group and are likely to be poorly-matched on ancestry. We excluded the outliers on this distance metric from the probands and siblings (> 2 median absolute deviations), leaving 868 autistic females and 2,235 siblings. See section 4.1.3 of the Supplementary Results for the outcomes of DNM enrichment analysis in this subset.

4. Ultra-rare variants

A substantial part of the ASC & SPARK datasets is formed of children with sequence data from one parent (N=18,816) or without any sequenced parents (N=23,114). Including these in analyses could potentially increase power. Specifically, they can be leveraged to study ultra-rare inherited alleles (when one parental exome is available) or the average effect of a mix of ultra-rare alleles of undetermined origin (case-control analysis), as detailed below. Similarly, ultra-rare variants ascertained in individuals without sequencing data from their parents ('case-control' cohorts) can be used to study the average effects of damaging *de novo* and inherited variants. Ultra-rare alleles in the parents are also well suited for determining whether the effect sizes of inherited variants differ between maternally and paternally inherited alleles (i.e. parent of origin effects) as they are already defined as 'those appearing in one parent' making it simpler to separate maternal from paternal alleles, and in addition offer the largest sample size possible by combining data across all trio- and duo-sequenced individuals.

4.1. Ultra-rare inherited variants in child-parent pairs

To be able to leverage the exome data from parents not sequenced as complete trios (Table S3), we studied the transmission of parental alleles in child-parent pairs, treating trios as two separate pairs rather than following the standard approach of comparing the transmission of rare variants in trios only. Studying transmission in parent-child pairs when sequence data is present from only one parent assumes the parents are not consanguineous (therefore unlikely to carry the same ultra-rare variant), and precludes the analysis of variants that are low-frequency but not extremely rare (as their transmission status cannot be determined reliably). Here, we examined ultra-rare parental alleles seen in one parent, in one family, and not seen in gnomAD. These were filtered for high-confidence calls (GQ > 32) to balance the transmission rates of synonymous variants.

To test for parent of origin effects, we ascertained ultra-rare inherited variants in ASC trios in addition to SPARK trios/duos, using a similar definition (seen in one parent in the ASC dataset and not in gnomAD). The rates of synonymous ultra-rare parental alleles were reasonably comparable between ASC & SPARK trio-sequenced probands, with an average of ~6 ultra-rare synonymous variants per parent, so we used all ultra-rare inherited variants to perform a mega-analysis (instead of a meta-analysis) of over-transmission in all 55,521 autistic-child-parent pairs.

4.2. Case-control variants in the ASC & SPARK

We evaluated the enrichment & liability of ultra-rare variants in the ASC case-control dataset as a supplementary analysis. This dataset contained samples from the Danish iPSYCH cohort[33] (cases = 4,863; controls = 5,002) and Swedish PAGES samples[30] (cases = 728; controls = 3,595). The processing and a previous sex-averaged analysis of these cohorts (5,591 autistic individuals and 8,597 controls) is described elsewhere.[13] Rare variants in iPSYCH were defined as those with an allele count ≤ 5 in the combined set of gnomAD non-Finnish Europeans (nonpsychiatric subset) and iPSYCH data (allele frequency ≤ 0.0043%). Rare variants in PAGES were defined as those with an allele count ≤ 5 in both ExAC r0.3 (nonpsychiatric subset; external allele frequency ≤ 0.0055%) and the autism cohort (internal allele frequency ≤ 0.014%).

For SPARK, we analyzed ultra-rare variant enrichment in the remaining individuals (6,646 probands and 2,436 siblings) who did not have sequence data from any parent (Table S5) - and as such were not included in all the previous analyses described above (*de novo* and inherited alleles). This design approximates the case-control analysis in ASC by using the siblings as controls (as done when examining the rate ratios of *de novo* mutations).

Whereas the ASC case-control dataset consisted of unrelated individuals, this SPARK sub-cohort included pairs of probands and siblings from the same family (like the trio-based analysis). We included variants seen with an allele frequency < 0.005% (in gnomAD and SPARK unrelated individuals' cohorts) that are seen in up to three individuals in the 'case-(sib)control' cohort of 9,082 individuals remaining after QC (allele frequency < 0.015%). We dropped 156 individuals with ultra-rare synonymous variants counts exceeding four median absolute deviations (> 33 variants), and compared the variant rates in the remaining 8,926 children (6,534 probands *versus* 2,392 siblings).

5. Measuring rare variant enrichment

5.1. Observed and expected variant rates

We calculated the *de novo* and inherited variant rates for synonymous, damaging missense and protein-truncating variants (most-constrained LOEUF decile) in each autism cohort (ASC, SPARK) by dividing the total number of variants by the sample size. The 95% Confidence Intervals (CI) for the variant rates (Figure S6, Figure S7, Figure S12, Figure S33) were estimated in `R` as follows:

$$95\%\ CI_{lower} = qchisq(0.025, \frac{\#\, Variants}{2 * \#\, Samples})$$

$$95\%\ CI_{upper} = qchisq(0.975, \frac{\#\, Variants + 1}{2 * \#\, Samples})$$

In the autism cohorts (ASC & SPARK), the expected variant rates were obtained from siblings or controls not diagnosed with autism (DNMs and case-control analysis) or rare untransmitted variants in the probands (inherited variants). In the NDD cohort (probands only; curated by Zhou and colleagues[2]), we compared the observed DNM counts to the expected DNM counts from a mutational model from gnomAD[23] (v2.1.1). To obtain exome-wide *de novo* mutation rates, we summed gene-level mutation rates across 17,296 autosomal protein-coding genes considered in our work. The expected rate of protein-truncating DNMs was ~0.05 (~0.0095 in the 1st LOEUF decile); for missense variants, we expect ~0.64 DNMs per child. To estimate the expected rate of damaging missense DNMs (MPC score ≥ 2), we explored the fraction of missense variants with MPC score ≥ 2 from missense variants identified in 9,223 trio-sequenced siblings not diagnosed with autism (included in the analysis presented in Figure 1). We find that ~4.8% of all missense DNMs in the ASC cohort and ~4.98% in SPARK have an MPC score ≥ 2. The corresponding fraction analyzed in another study[2] was 4.50% (largely overlapping samples).

We decided to use an estimate of 5% (rounding these fractions), corresponding to an expected rate of ~0.032 damaging missense DNMs per child.
The observed synonymous mutation rate in the total cohort (~0.28 per child) was significantly higher (p = 0.00076; binomial test) than the mutation rate from the gnomAD mutation model (~0.27). We therefore calibrated the damaging mutation rates to account for potential underlying heterogeneity between the sequencing cohorts (e.g., differences in capture targets & pipelines). For each sequencing cohort, we calculated the observed/expected synonymous DNM rate ratio (*versus* gnomAD model) (GeneDX= 1.01, DDD=1.09, RUMC = 0.97, DEE=0.9, ID=1.05); the mutation rates calculated from gnomAD model were then multiplied by these ratios to obtain calibrated mutation rates for each sequencing cohort. We then calculated the expected number of DNMs from a poisson distribution:

$$Expected\ DNM\ count\ =\ sum(rpois(n\ =\ N, lambda\ =\ mu\ *\ k)$$

Where `N` is the sample size (per sequencing cohort, per sex), `mu` is the mutation rate (from the gnomAD model), and `k` is the scaling factor (observed/expected synonymous rate). (`rpois` is an `R` function from `stats` package that generates random samples from a poisson distribution with a given rate parameter `lambda`). We repeated this procedure 5,000 times and took the median expected count. The counts were then summed across all sub-cohorts to get the expected DNM counts for 17,698 males and 13,867 females.

5.2. Sex-stratified comparisons

5.2.1. De novo mutations

We used the ratio between the rate of DNMs in the probands and the siblings (or female and male probands in direct comparisons) to evaluate the enrichment of a specific class of DNMs in a gene set or exome-wide:

$$DNM\ rate\ ratio\ =\ \frac{\#DNM\ in\ probands\ /\ \#\ probands}{\#\ DNM\ in\ siblings\ /\ \#\ siblings}$$

To test for significance, we compared the counts DNMs in the probands to the counts in their siblings in `R`:

```
binom.test(x= #DNMs_in_probands, n= (#DNMs_in_probands +
#DNMs_in_siblings), p= #probands/(#probands + #siblings),
alternative=”two.sided”)
```

We note that the measure used to test the relative enrichment in the binomial test is the ***fraction*** of DNMs in ***probands*** among ***all*** DNMs.

$$Fraction \ = \ \frac{\#DNM \ in \ probands}{\# \ DNM \ in \ siblings} = \frac{\#DNM \ in \ probands}{\# \ Total \ DNMs - \# \ DNMs \ in \ probands}$$

It is different from the DNM ***rate ratio*** (a risk ratio) between the ***probands*** and ***siblings***.

$$Rate \ Ratio \ = \ \frac{\#DNM \ in \ probands \ * \ \# \ siblings}{\# \ DNM \ in \ siblings \ * \ \# \ probands}$$

Here, we report the enrichment as rate ratios as these are easy to interpret and can be used for DNMs, inherited and case-control variants to express fold-enrichment. It is possible to convert between the rate ratio and fraction as follows:

$$Rate \ Ratio \ = \ \frac{\#DNM \ in \ probands \ * \ \# \ siblings}{(\# \ Total \ DNMs - \# \ DNM \ in \ probands) * \ \# \ probands}$$

$$Rate \ Ratio \ = \ \frac{\#DNM \ in \ probands \ * \ \# \ siblings}{(\# \ Total \ DNMs * \ \# \ probands) - \ (\# \ DNM \ in \ probands * \ \# \ probands)}$$

$$Rate \ Ratio \ = \ \frac{(\#DNM \ in \ probands \ / \ \# \ Total \ DNMs) \ * \ \# \ siblings}{\# \ probands - \ (\# \ DNM \ in \ probands / \ \# \ Total \ DNMs) * \ \# \ probands}$$

$$Rate \ Ratio \ = \ \frac{(\#DNM \ in \ probands \ / \ \# \ Total \ DNMs) \ * \ \# \ siblings}{(1 - \ (\# \ DNM \ in \ probands / \ \# \ Total \ DNMs) \ ) \ * \ \# \ probands}$$

$$Rate \ Ratio \ = \ \frac{Fraction * \ \# \ siblings}{(1 - \ Fraction \ ) \ * \ \# \ probands}$$

Although we calculated the rate ratios directly from the observed counts, the above equation is useful for estimating the confidence intervals with the same test used to calculate the *p*-values; the binomial test returns the upper and lower bounds of the 95% CI of the observed fraction. We converted these bounds to equivalent rate ratios by plugging them into this formula:

$$DNM \ ratio \ CI \ = \ \frac{Fraction \ CI_{binomial \ test} * \# \ siblings}{(1 - Fraction \ CI_{binomial \ test}) * \# \ probands}$$

5.2.2. Over-transmission

To assess over-transmission in the probands, we calculated the ratio between transmitted untransmitted parental alleles:

$$Transmitted / \ Untransmitted \ ratio \ = \ \frac{\# \ parental \ alleles \ transmitted \ to \ the \ probands}{\# \ untransmitted \ parental \ alleles}$$

We then compared the significance of the difference in the counts of transmitted and untransmitted alleles:

```
binom.test(
x=#variants_transmitted_to_probands,
n=#variants_transmitted_to_probands + #untransmitted_variants,
p=0.5,alternative="two.sided")
```

We scaled the 95% CIs from the binomial test as follows:

$$Tr. / \ Ut. \ ratio \ CI \ = \frac{Fraction \ CI_{binomial \ test}}{1 - Fraction \ CI_{binomial \ test}}$$

For ultra-rare inherited variants, this was used to test all parental alleles (maternal+paternal) as well as maternal and paternal alleles separately.

5.2.3. Case-control comparisons

Variants were compared between cases and controls in a similar manner to DNMs, i.e., using the sample size to derive the expected fraction of variants in the cases. These case-control comparisons are more sensitive to differences in population architecture than comparisons of *de novo* mutations and transmitted-untransmitted alleles. When testing the enrichment of ultra-rare variants in cases versus controls in a regression framework, synonymous variant counts can be used as a covariate along with principal components. When using a binomial test, the expected variant rates can be adjusted for population differences by using the synonymous variants. Therefore, the damaging variant comparisons were additionally adjusted by the synonymous variant counts as follows:

$$Case/Control\ rate\ ratio\ =\ \frac{\#\ damaging\ variants\ in\ cases\ /\ \#\ synonymous\ variants\ in\ cases}{\#\ damaging\ variants\ in\ controls\ /\ \#\ synonymous\ variants\ in\ controls}$$

```
binom.test(x= #damaging_in_cases, n= (#damaging_in_cases +
#damaging_in_ctrls), p= #syn_in_cases/(#syn_in_cases +
#syn_in_ctrls), alternative=”two.sided”)
```

$$Case/Ctrl\ CI\ =\ \frac{Fraction\ CI_{binomial\ test} * \#synonymous\ in\ controls}{(1 - Fraction\ CI_{binomial\ test}) * \#synonymous\ in\ cases}$$

Additionally, we calculated the rates of all ultra-rare parental alleles (transmitted & untransmitted) across all mothers and fathers in these child-parent pairs, then compared the variant rates in the mothers to the corresponding rates in the fathers using a binomial test. As these are similar to case-control analyses, we used synonymous variant counts as a reference point.

$$Mothers/Fathers\ rate\ ratio\ =\ \frac{\#\ damaging\ variants\ in\ mothers\ /\ \#\ synonymous\ variants\ in\ mothers}{\#\ damaging\ variants\ in\ fathers\ /\ \#\ synonymous\ variants\ in\ mothers}$$

```
binom.test(x= #damaging_in_mothers, n= (#damaging_in_mothers +
#damaging_in_fathers), p= #syn_in_mothers/(#syn_in_mothers +
#syn_in_fathers), alternative=”two.sided”)
```

$$Mothers/Fathers\ CI\ =\ \frac{Fraction\ CI_{binomial\ test} * \#synonymous\ in\ mothers}{(1 - Fraction\ CI_{binomial\ test}) * \#synonymous\ in\ fathers}$$

5.3. Sex differences in enrichment

Sex differences were assessed by comparing autistic females to autistic males in the same manner as we compared autistic individuals to their siblings. Rate ratios > 1 indicate higher variant rates in autistic females compared to autistic males. To test for significant differences in de novo mutation counts, we compared the DNM counts in females and males:

```
binom.test(x= #DNMs_in_females,
n= (#DNMs_in_males + #DNMs_in_females),
p= #females/(#males + #females), alternative="two.sided")
```

$$DNM\ ratio\ CI\ =\ \frac{Fraction\ CI_{binomial\ test}\ *\ \#\ Females}{(1 - Fraction\ CI_{binomial\ test})\ *\ \#\ Males}$$

Case-control variants were tested in the same manner as DNMs. For inherited variants, we compared the counts of parental alleles transmitted to females and males:

```
binom.test(x=#variants_transmitted_to_females,
n= #variants_transmitted_to_males_or_females,
p= #all_parental_alleles_in_females/#all_parental_alleles,
alternative="two.sided")
```

$$Tr./Ut.\ ratio\ CI\ =\ \frac{Fraction\ CI_{binomial\ test}\ *\ \#\ parental\ alleles\ in\ females}{(1 - Fraction\ CI_{binomial\ test})\ *\ \#\ parental\ alleles\ in\ males}$$

This was also used to test for sex differences in over-transmission of ultra-rare maternal or paternal alleles (i.e. transmission of maternal alleles to daughters *versus* to sons, paternal alleles to daughters *versus* to sons), thus testing indirectly for parent of origin effects, which would appear as significant sex differences in over-transmission from one group of parents but not the other. To examine parent of origin effects directly (maternal *versus* paternal alleles), we used the following scheme (this example shows how to test maternal *versus* paternal transmission to daughters):

```
binom.test(
x= #variants_transmitted_from_mothers_to_daughters,
n= #variants_transmitted_from_mothers_to_daughters+
   #variants_transmitted_from_fathers_to_daughters,
p= #transmitted_and_untransmitted_maternal_variants/
  (#transmitted_and_untransmitted_maternal_variants+
   #transmitted_and_ultransmitted_paternal_variants),
alternative="two.sided")
```

$$Tr./Ut.\ ratio\ CI\ =\ \frac{Fraction\ CI_{binomial\ test} * \#\ maternal\ \ alleles}{(1 - Fraction\ CI_{binomial\ test}) * \#\ paternal\ \ alleles}$$

5.4. Meta-analysis between ASC & SPARK cohorts

We performed a fixed-effect inverse-variance-weighted meta-analysis of the risk ratios (rate ratios or transmitted/untransmitted ratios) in ASC & SPARK using `metagen` function from `meta` package[34] in `R`:

```
metagen(sm = "RR",
      fixed = TRUE,
      studlab = Cohort,
      TE = Risk_Ratio,
      level.ci = 0.95,
      lower = Risk_Ratio_CIL,
      upper = Risk_Ratio_CIU,
      pval = Risk_Ratio_Pval,
      method.tau = "PM")
```

5.5. Correcting for multiple testing

We adjusted the p-values from each experiment (e.g. 54 tests when performing exome-wide enrichment testing) for the Family-wise (Experiment-wise) Error Rate using Bonferroni correction. We also performed more lenient adjustment for False Discovery Rate using Benjamini-Hochberg method. Both were performed in R:

```
p.adjust(method='bonferroni')
p.adjust(method='BH')
```

6. Measuring effect sizes on the liability scale

6.1. Standard (Differing Thresholds) Liability Threshold Model

We compared the change in trait liability for each variant class to reflect the deviation from population mean liability that would result from carrying a variant in that class. The relative differences in variant frequency between the probands and siblings was converted to Z scores on the liability scale, assuming the liability is normally distributed and centered around zero in the general population, with differing thresholds in males and females (Figure

S5A). The mathematical derivation is explained in Ref[30]. Here, we summarize the concept behind this procedure for those less familiar with the statistical concepts:

- In a Liability Threshold Model assuming additive genetic risk that is normally distributed in the population, the threshold is the point that forms the boundary of an area under the normal distribution curve that is equivalent to the trait prevalence (Figure S5A). For example, an autism prevalence of 0.025 indicates that the distance between the population mean risk and the threshold is 1.96 standardized units, putting 2.5% of the population in the right tail.
- We can work out the distance (in standardized units) between the population mean (zero, given how the model is defined) and the threshold using the inverse of the standard normal cumulative distribution function (Φ^{-1}), or '*norminv*' in short. The threshold in the population equals the average liability in the population (i.e., zero) + *norminv*(Prevalence) as shown in Figure S5A. The inverse normal function Φ^{-1} is implemented in `R` using the function `qnorm`, which returns the lower tail by default, whereas the prevalence reflects the upper tail (fraction of individuals *above* the threshold). We can get the upper tail by passing an argument to the function ('`qnorm(Prevalence,lower.tail=FALSE)`) or simply by using the complement of the prevalence, `qnorm(1 - Prevalence).`
  - Now we move to the individuals harboring a certain class of variants, for instance damaging PTVs. The liability in this sub-population can also be approximated by a normal distribution, and the mean of this distribution will be the *average PTV liability.* This average variant liability is a measure of the average effect size of PTVs on the liability scale. Individuals harboring PTVs who are autistic will form the upper tail above a certain threshold, and non-autistic individuals harboring PTVs will be in the lower tail (Figure S5A).
  - Whereas the threshold in the population liability distribution reflects the prevalence in the population, the threshold in the PTV liability distribution will reflect autism *prevalence among the individuals harboring PTVs* in the general population. In accordance with the original derivation in ref,[34] we refer to this fraction as the 'penetrance'.
  - Similar to the prevalence, the penetrance is a cumulative density and as such can be converted to equivalent standardized units in the PTV liability distribution using the inverse normal cumulative density distribution function (Φ^{-1}). The threshold here equals the (unknown) average PTV liability + *norminv*(Penetrance). We can get this is `R` using `qnorm(1 - Penetrance).`

- By leveraging the fact that the threshold in both distributions is the same, we can now estimate the distance between the population mean (zero) and the (unknown) mean protein-truncating variant liability. As shown in Figure S5A, we can write:
  threshold = `qnorm(1 - Prevalence)` = *average PTV liability* + `qnorm(1 - Penetrance)`
- The prevalence in the general population is a known parameter. Autism is diagnosed in 1 in 40 male individuals (a prevalence of 2.5% in males), with a 1:4 male-to-female ratio (prevalence in females = 0.25 x 2.5% = 0.625%). Previous estimates suggested that about one third of autistic individuals in the population have profound deficits with cognitive impairment [35,36]. Among 11,630 autistic individuals in SPARK who could be classified, ~ 36% fell in the autism with motor or cognitive impairment group (35% in males & 40% in females). For estimating liability, we scaled the sex-specific population prevalence using these percentages, i.e., using a prevalence estimate of 0.88% in males (35% x 2.5%) and 0.25% in females (40% x 0.625%) for liability calculations in the autism with motor or cognitive impairment group; for the autism without motor or cognitive impairment group, we used a prevalence of 1.62% in males (2.5% - 0.88%) and 0.38% in females (0.625% - 0.25%). For directly comparing those with motor or cognitive impairment to those without these co-occurring difficulties, we used a prevalence of 0.4 in females and 0.35 in males.
- The prevalence among the individuals harboring PTVs, on the other hand, is not known. We can, however, convert the variant rates in the study cohort to population estimates, and convert these population estimates to penetrance estimates. Specifically, the penetrance is the ratio between PTV rate among autistic individuals and the overall frequency of PTVs in the population. The overall population frequency of PTVs can, in turn, be estimated from the study cohort, namely by summing the frequency of PTVs in the probands weighed (multiplied) by their fraction in the population (trait prevalence) and the frequency of PTVs in the siblings weighed by their relative fraction as well (1 minus the trait prevalence).
- Now that we have the prevalence estimates both in the general population and among individuals harboring PTVs (penetrance), we estimate the average PTV liability as `qnorm(1 - Prevalence)- qnorm(1 - Penetrance)`.

- The liability stratified by sex was measured in `R` using these formulas:

$$Rate_{Population} = Prevalence * Rate_{Probands} + (1 - Prevalence) * Rate_{Siblings}$$

$$Penetrance = \frac{Rate_{Probands}}{Rate_{Population}} * Prevalence$$

$$Z = qnorm(1 - Prevalence) - qnorm(1 - Penetrance)$$

- The *p*-values were those obtained from the binomial tests detailed above. The standard error, and then the confidence intervals around Z, were estimated from these *p*-values:

$$Q = qnorm(1 - \frac{Binomial\ test\ P\ value}{2})$$

$$SE = \frac{abs(Z)}{Q}$$

$$95\%\ CI = Z \pm (1.96 * SE)$$

- The difference between males and females liability estimates was measured as follows:

$$Z_{Difference} = Z_{Females} - Z_{Males}$$

$$SE_{Difference} = sqrt(SE^2_{Females} + SE^2_{Males})$$

$$Q_{Difference} = \frac{abs(Z_{Difference})}{SE_{Difference}}$$

$$P_{Difference} = 2 * (1 - pnorm(Q_{Difference}))$$

- To test for parent of origin effects, we calculated the difference between the effect sizes of paternal or maternal alleles transmitted to female or male children (daughters/sons).

- The inverse-variance-weighted average from both sexes and its confidence interval was measured as follows:

$$Z_{Both} = (\frac{Z_{Females}}{SE^2_{Females}} + \frac{Z_{Males}}{SE^2_{Males}}) \div (\frac{1}{SE^2_{Females}} + \frac{1}{SE^2_{Males}})$$

$$SE_{Both} = 1 \div sqrt(\frac{1}{SE^2_{Females}} + \frac{1}{SE^2_{Males}})$$

$$95\%\ CI_{Both} = Z_{Both} \pm (1.96 * SE_{Both})$$

- The meta-analysis *p*-value was obtained using Fisher's method implemented in the `metap` package[34]:

$$P_{Both} = metap::sumlog(P_{Females}, P_{Males})$$

- The *p*-values for each experiment were corrected for Family-wise Error Rate (Bonferroni correction) and False Discovery Rate (Benjamini-Hochberg correction), as shown for the risk ratios.

6.2. Differing Variance Liability Model

The sex difference in a trait prevalence can be explained by the two sexes having differing variances instead of differing thresholds or means.[37] When the parameters of the liability distribution vary between the sexes, the inverse cumulative density function with a given mean and standard deviation ($\Phi_{\mu,\sigma}^{-1}$) is used to determine the threshold (θ) from the prevalence. Given that the variance of the distribution is different for the two sexes, the scale is also different, and the threshold will be in Z score units relative to the standard deviation of the underlying distribution (θ_m & θ_f). However, when we model the difference in prevalence as the outcome of differences in variance alone, the mean and the threshold are the same in both sexes. This entails that the distance between the mean, which can be considered the reference point (zero), and the threshold (θ_m & θ_f) is equal on both scales ($\theta = \theta_m = \theta_f$), and can in turn be used as a measuring unit across all scales. In other words, we can express the effect sizes as fractions of the threshold by dividing the measured variant liability in one sex by the threshold for in its scale. This in turn allows the projection of the effect sizes from one scale with a certain standard deviation to another scale with different standard deviation by multiplying the fraction by the threshold of the second scale.

To project the effect sizes from the two liability distributions with prevalence ψ_m & ψ_f and standard deviations σ_m & σ_f to a population scale with with a given prevalence (ψ) and standard deviation = 1, we did the following in `R`:

- We estimated the threshold on each scale (population distribution with variance = σ^2, males with variance = σ_m^2, females with variance = σ_f^2, mean = 0 on all scales):

$$\theta = qnorm(1 - \psi , sd = 1)$$

$$\theta_m = qnorm(1 - \psi_m, sd = \sigma_m)$$

$$\theta_f = qnorm(1 - \psi_f, sd = \sigma_f)$$

- As shown in section 6.1 above, we estimated the prevalence of autism among those harboring damaging variants in the population (‘penetrance’) in males (ρ_m) and females (ρ_f) and then the corresponding deviation from the threshold:

$$\vartheta_m = qnorm(1 - \rho_m, sd = \sigma_m)$$

$$\vartheta_f = qnorm(1 - \rho_f, sd = \sigma_f)$$

- The liability is the difference between these two Z scores (Figure S5A), however these are on different scales relative to their standard deviation (Figure S5B). To normalize the scores, we divide them by the corresponding threshold on each scale (θ_m or θ_f), which means that they will be expressed as a fraction of the threshold. The threshold is the same across all scales. To have both effect sizes on a unified population scale, we multiply these fractions by the threshold (θ):

$$Z_m = (\theta_m - \vartheta_m) * \theta \div \theta_m = \theta - \vartheta_m * \theta \div \theta_m$$

$$Z_f = (\theta_f - \vartheta_f) * \theta \div \theta_f = \theta - \vartheta_f * \theta \div \theta_f$$

7. Gene set enrichment

7.1. Gene sets

The Simons Foundation Autism Research Initiative (SFARI) database provides curated scores for 1,172 genes that reflect the strength of evidence linking each gene to autism risk.[38] There are 3,58 autosomal and 47 X-linked SFARI Category 1 (high-confidence) & S (syndromic) genes with strong evidence of association with autism (354 autosomal genes included in the current analysis cohort after QC). We tested the enrichment of DNMs and rare inherited variants after removing these genes as well as in this gene set only (216 in the 1st LOEUF decile, 62 in the 2nd decile, 24 in the 3rd decile).

Genes with sex-biased expression in the fetal cortex were obtained from a recent study by Kissel and and colleagues,[10] who meta-analyzed two large fetal brain bulk RNA-seq datasets: BrainVar project (dorsolateral prefrontal cortex) and UCLA dataset (cortex). The study identified 101 significant genes with FDR < 0.1 from a meta-analysis of two datasets but, according to the study, these genes did not overlap known autism predisposition genes. To maximize sensitivity in our analysis, we used the data presented by the authors in Table S4 (see the Supplements of Ref[10]) to create a list of genes with similar direction of effects in the two datasets (t_UCLA * t_BV > 0) that achieved nominal significance in the meta-analysis (invNorm_rawPval < 0.05), then split these into female-biased genes (t_UCLA < 0 & t_BV < 0) and male-biased genes (t_UCLA > 0 & t_BV > 0). These genes were filtered further for protein-coding autosomal genes that were included in our analysis, resulting in

117 female-biased (8 in the 1st LOEUF decile, 15 in the 2nd decile, 11 in the 3rd decile) and 305 male-biased genes (39 in 1st LOEUF decile, 49 in the 2nd decile, 42 in the 3rd decile).

Genes with significant sex-biased expression in the adult human cortex were obtained from a recent study by Fass and colleagues.[11] They examined two large collections of postmortem brain RNA sequencing data, the Genotype-Tissue Expression project (GTEx, cortex) and the CommonMind Consortium (CMC, frontocortical samples), producing a meta-analyzed set of sex-differential expression. Similar to the strategy outlined above, we used the results of the meta-analysis presented by the authors in Supplemental Table 4 (see the Supplements of Ref[11]) to create a list of genes that had the same direction of effect in GTEx and CMC and a nominally significant combined P-value (pvalue < 0.05). The gene sets were composed of 2,427 female-biased (381 in the 1st LOEUF decile, 344 in the 2nd decile, 319 in the 3rd decile) and 2,852 male-biased genes (483 in 1st LOEUF decile, 443 in the 2nd decile, 416 in the 3rd decile).

7.2. Gene set enrichment versus matched genes

For each tested gene set, we selected a matched set (see 7.3 below) and counted DNMs, transmitted and untransmitted variants; we repeated this procedure 10,000 times with replacement and took the average ratio (rate ratio between DNM counts in probands and siblings or transmitted to untransmitted ratio in the probands); we then used this ratio as the expected ratio in a binomial test as described above. Specifically, we tested the difference between the rate of DNMs between probands and siblings against the permutation-averaged expected ratio for this gene set (instead of the sample size ratio used in the exome-wide analyses), and similarly tested rare variant over-transmission against the permutation-averaged expected transmitted-to-untransmitted ratio for the given gene set (instead of 0.5 as used in the exome-wide analysis). We also used the average variant rates across these 10,000 permutations instead of the rate in siblings to estimate the variant liability attributed to a gene set in excess of what is expected for matched genes.

7.3. Selecting random sets of matching genes

We followed a procedure similar to that previously used by Ouwenga and Dougherty[39] to select sets of control genes for gene set burden analysis. We used a multi-dimensional kernel density estimator (KDE) from the `ks` package[40] in `R` to select these control genes. First, we created a table of features (coding length, brain expression level, and LOEUF bins) and used it to build a 3D KDE. Next, we used the test gene set to build a density distribution and evaluated the remaining genes (not in the gene set) on this

distribution. We then evaluated these remaining genes on a density distribution built using all genes rather than the gene set genes. The ratio of the two estimates was then used as a sampling weight. Here we show the `R` code used to implement this:

```
kde_set_genes = ks::kde(
                x = features_set_genes,
                eval.points = features_remaining_genes
                )
                #  feature_x_genes:  table  gene  x  length,  expr.,
          loeuf
kde_all_genes = ks::kde(
                x = features_all_genes,
                eval.points = features_remaining_genes
                )
kde_weights =  kde_set_genes$estimate ÷ kde_all_genes$estimate
genes_random_idx = sample(
                1:length(remaining_genes),
                length(set_genes),
                prob =kde_weights
                )
genes_randoms = remaining_genes[genes_random_idx]
```

8. Supplemental References

1. Antaki, D., Guevara, J., Maihofer, A.X., Klein, M., Gujral, M., Grove, J., Carey, C.E., Hong, O., Arranz, M.J., Hervas, A., et al. (2022). A phenotypic spectrum of autism is attributable to the combined effects of rare variants, polygenic risk and sex. Nat. Genet. *54*, 1284–1292. https://doi.org/10.1038/s41588-022-01064-5.
2. Zhou, X., Feliciano, P., Shu, C., Wang, T., Astrovskaya, I., Hall, J.B., Obiajulu, J.U., Wright, J.R., Murali, S.C., Xu, S.X., et al. (2022). Integrating de novo and inherited variants in 42,607 autism cases identifies mutations in new moderate-risk genes. Nat. Genet. *54*, 1305–1319. https://doi.org/10.1038/s41588-022-01148-2.
3. Wang, T., Kim, C.N., Bakken, T.E., Gillentine, M.A., Henning, B., Mao, Y., Gilissen, C., The SPARK Consortium, Nowakowski, T.J., Eichler, E.E., et al. (2022). Integrated gene analyses of de novo variants from 46,612 trios with autism and developmental disorders. Proc. Natl. Acad. Sci. *119*, e2203491119. https://doi.org/10.1073/pnas.2203491119.
4. Jacquemont, S., Coe, B.P., Hersch, M., Duyzend, M.H., Krumm, N., Bergmann, S., Beckmann, J.S., Rosenfeld, J.A., and Eichler, E.E. (2014). A Higher Mutational Burden in Females Supports a “Female Protective Model” in Neurodevelopmental Disorders. Am. J. Hum. Genet. *94*, 415–425. https://doi.org/10.1016/j.ajhg.2014.02.001.
5. Zhao, X., Leotta, A., Kustanovich, V., Lajonchere, C., Geschwind, D.H., Law, K., Law, P., Qiu, S., Lord, C., Sebat, J., et al. (2007). A unified genetic theory for sporadic and inherited autism. Proc. Natl. Acad. Sci. U. S. A. *104*, 12831–12836. https://doi.org/10.1073/pnas.0705803104.
6. Krumm, N., Turner, T.N., Baker, C., Vives, L., Mohajeri, K., Witherspoon, K., Raja, A., Coe, B.P., Stessman, H.A., He, Z.-X., et al. (2015). Excess of rare, inherited truncating mutations in autism. Nat. Genet. *47*, 582–588. https://doi.org/10.1038/ng.3303.
7. Iossifov, I., Levy, D., Allen, J., Ye, K., Ronemus, M., Lee, Y.-H., Yamrom, B., and Wigler, M. (2015). Low load for disruptive mutations in autism genes and their biased transmission. Proc. Natl. Acad. Sci. U. S. A. *112*, E5600-5607. https://doi.org/10.1073/pnas.1516376112.
8. Cui, W., Xue, H., Wei, L., Jin, J., Tian, X., and Wang, Q. (2021). High heterogeneity undermines generalization of differential expression results in RNA-Seq analysis. Hum. Genomics *15*, 7. https://doi.org/10.1186/s40246-021-00308-5.
9. Sturm, G., List, M., and Zhang, J.D. (2021). Tissue heterogeneity is prevalent in gene expression studies. NAR Genomics Bioinforma. *3*, lqab077. https://doi.org/10.1093/nargab/lqab077.
10. Kissel, L.T., Pochareddy, S., An, J.-Y., Sestan, N., Sanders, S.J., Wang, X., and Werling, D.M. (2024). Sex-Differential Gene Expression in Developing Human Cortex and Its Intersection With Autism Risk Pathways. Biol. Psychiatry Glob. Open Sci. *4*, 100321. https://doi.org/10.1016/j.bpsgos.2024.100321.
11. Fass, S.B., Mulvey, B., Chase, R., Yang, W., Selmanovic, D., Chaturvedi, S.M., Tycksen, E., Weiss, L.A., and Dougherty, J.D. (2024). Relationship between sex biases in gene expression and sex biases in autism and Alzheimer’s disease. Biol. Sex Differ. *15*, 47. https://doi.org/10.1186/s13293-024-00622-2.
12. Wingo, A.P., Liu, Y., Gerasimov, E.S., Vattathil, S.M., Liu, J., Cutler, D.J., Epstein, M.P., Blokland, G.A.M., Thambisetty, M., Troncoso, J.C., et al. (2023). Sex differences in brain protein expression and disease. Nat. Med. *29*, 2224–2232. https://doi.org/10.1038/s41591-023-02509-y.
13. Fu, J.M., Satterstrom, F.K., Peng, M., Brand, H., Collins, R.L., Dong, S., Wamsley, B., Klei, L., Wang, L., Hao, S.P., et al. (2022). Rare coding variation provides insight into the genetic architecture and phenotypic context of autism. Nat. Genet. *54*, 1320–1331. https://doi.org/10.1038/s41588-022-01104-0.

14. Deciphering Developmental Disorders Study, Kaplanis, J., Samocha, K.E., Wiel, L., Zhang, Z., Arvai, K.J., Eberhardt, R.Y., Gallone, G., Lelieveld, S.H., Martin, H.C., et al. (2020). Evidence for 28 genetic disorders discovered by combining healthcare and research data. Nature *586*, 757–762. https://doi.org/10.1038/s41586-020-2832-5.
15. Appenzeller, S., Balling, R., Barisic, N., Baulac, S., Caglayan, H., Craiu, D., De Jonghe, P., Depienne, C., Dimova, P., Djémié, T., et al. (2014). De Novo Mutations in Synaptic Transmission Genes Including DNM1 Cause Epileptic Encephalopathies. Am. J. Hum. Genet. *95*, 360–370. https://doi.org/10.1016/j.ajhg.2014.08.013.
16. Rauch, A., Wieczorek, D., Graf, E., Wieland, T., Endele, S., Schwarzmayr, T., Albrecht, B., Bartholdi, D., Beygo, J., Di Donato, N., et al. (2012). Range of genetic mutations associated with severe non-syndromic sporadic intellectual disability: an exome sequencing study. Lancet Lond. Engl. *380*, 1674–1682. https://doi.org/10.1016/S0140-6736(12)61480-9.
17. De Ligt, J., Willemsen, M.H., Van Bon, B.W.M., Kleefstra, T., Yntema, H.G., Kroes, T., Vulto-van Silfhout, A.T., Koolen, D.A., De Vries, P., Gilissen, C., et al. (2012). Diagnostic Exome Sequencing in Persons with Severe Intellectual Disability. N. Engl. J. Med. *367*, 1921–1929. https://doi.org/10.1056/NEJMoa1206524.
18. Wright, C.F., Fitzgerald, T.W., Jones, W.D., Clayton, S., McRae, J.F., van Kogelenberg, M., King, D.A., Ambridge, K., Barrett, D.M., Bayzetinova, T., et al. (2015). Genetic diagnosis of developmental disorders in the DDD study: a scalable analysis of genome-wide research data. The Lancet *385*, 1305–1314. https://doi.org/10.1016/S0140-6736(14)61705-0.
19. Loomes, R., Hull, L., and Mandy, W.P.L. (2017). What Is the Male-to-Female Ratio in Autism Spectrum Disorder? A Systematic Review and Meta-Analysis. J. Am. Acad. Child Adolesc. Psychiatry *56*, 466–474. https://doi.org/10.1016/j.jaac.2017.03.013.
20. Foster, I. (2011). Globus Online: Accelerating and Democratizing Science through Cloud-Based Services. IEEE Internet Comput. *15*, 70–73. https://doi.org/10.1109/MIC.2011.64.
21. Chorbadjiev, L., Cokol, M., Weinstein, Z., Shi, K., Fleisch, C., Dimitrov, N., Mladenov, S., Xu, S., Hall, J., Ford, S., et al. (2024). The Genotype and Phenotypes in Families (GPF) platform manages the large and complex data at SFARI (Bioinformatics) https://doi.org/10.1101/2024.02.08.579330.
22. McLaren, W., Gil, L., Hunt, S.E., Riat, H.S., Ritchie, G.R.S., Thormann, A., Flicek, P., and Cunningham, F. (2016). The Ensembl Variant Effect Predictor. Genome Biol. *17*, 122. https://doi.org/10.1186/s13059-016-0974-4.
23. Genome Aggregation Database Consortium, Karczewski, K.J., Francioli, L.C., Tiao, G., Cummings, B.B., Alföldi, J., Wang, Q., Collins, R.L., Laricchia, K.M., Ganna, A., et al. (2020). The mutational constraint spectrum quantified from variation in 141,456 humans. Nature *581*, 434–443. https://doi.org/10.1038/s41586-020-2308-7.
24. The 1000 Genomes Project Consortium (2015). A global reference for human genetic variation. Nature *526*, 68–74. https://doi.org/10.1038/nature15393.
25. Chang, C.C., Chow, C.C., Tellier, L.C., Vattikuti, S., Purcell, S.M., and Lee, J.J. (2015). Second-generation PLINK: rising to the challenge of larger and richer datasets. GigaScience *4*, 7. https://doi.org/10.1186/s13742-015-0047-8.
26. Manichaikul, A., Mychaleckyj, J.C., Rich, S.S., Daly, K., Sale, M., and Chen, W.-M. (2010). Robust relationship inference in genome-wide association studies. Bioinformatics *26*, 2867–2873. https://doi.org/10.1093/bioinformatics/btq559.
27. Van der Auwera, G.A., Carneiro, M.O., Hartl, C., Poplin, R., del Angel, G., Levy-Moonshine, A., Jordan, T., Shakir, K., Roazen, D., Thibault, J., et al. (2013). From FastQ Data to High-Confidence Variant Calls: The Genome Analysis Toolkit Best Practices Pipeline: The Genome Analysis Toolkit Best Practices Pipeline. In Current Protocols in Bioinformatics, A. Bateman, W. R. Pearson, L. D. Stein, G. D. Stormo, and J. R. Yates, eds. (John Wiley & Sons, Inc.), p. 11.10.1-11.10.33.
28. Wright, M.N., and Ziegler, A. (2017). **ranger** : A Fast Implementation of Random Forests for High Dimensional Data in *C++* and *R*. J. Stat. Softw. *77*.

https://doi.org/10.18637/jss.v077.i01.
29. Kuhn, M. (2008). Building Predictive Models in *R* Using the **caret** Package. J. Stat. Softw. *28*. https://doi.org/10.18637/jss.v028.i05.
30. Satterstrom, F.K., Kosmicki, J.A., Wang, J., Breen, M.S., De Rubeis, S., An, J.-Y., Peng, M., Collins, R., Grove, J., Klei, L., et al. (2020). Large-Scale Exome Sequencing Study Implicates Both Developmental and Functional Changes in the Neurobiology of Autism. Cell *180*, 568-584.e23. https://doi.org/10.1016/j.cell.2019.12.036.
31. Hail Team (2022). Hail. Version 0.2.
32. Danecek, P., Bonfield, J.K., Liddle, J., Marshall, J., Ohan, V., Pollard, M.O., Whitwham, A., Keane, T., McCarthy, S.A., Davies, R.M., et al. (2021). Twelve years of SAMtools and BCFtools. GigaScience *10*, giab008. https://doi.org/10.1093/gigascience/giab008.
33. Satterstrom, F.K., Walters, R.K., Singh, T., Wigdor, E.M., Lescai, F., Demontis, D., Kosmicki, J.A., Grove, J., Stevens, C., Bybjerg-Grauholm, J., et al. (2019). Autism spectrum disorder and attention deficit hyperactivity disorder have a similar burden of rare protein-truncating variants. Nat. Neurosci. *22*, 1961–1965. https://doi.org/10.1038/s41593-019-0527-8.
34. Balduzzi, S., Rücker, G., and Schwarzer, G. (2019). How to perform a meta-analysis with R: a practical tutorial. Evid. Based Ment. Health *22*, 153–160. https://doi.org/10.1136/ebmental-2019-300117.
35. Dougherty, J.D., Marrus, N., Maloney, S.E., Yip, B., Sandin, S., Turner, T.N., Selmanovic, D., Kroll, K.L., Gutmann, D.H., Constantino, J.N., et al. (2022). Can the “female protective effect” liability threshold model explain sex differences in autism spectrum disorder? Neuron *110*, 3243–3262. https://doi.org/10.1016/j.neuron.2022.06.020.
36. Zeidan, J., Fombonne, E., Scorah, J., Ibrahim, A., Durkin, M.S., Saxena, S., Yusuf, A., Shih, A., and Elsabbagh, M. (2022). Global prevalence of autism: A systematic review update. Autism Res. *15*, 778–790. https://doi.org/10.1002/aur.2696.
37. Falconer, D.S. (1967). The inheritance of liability to diseases with variable age of onset, with particular reference to diabetes mellitus. Ann. Hum. Genet. *31*, 1–20. https://doi.org/10.1111/j.1469-1809.1967.tb02015.x.
38. Abrahams, B.S., Arking, D.E., Campbell, D.B., Mefford, H.C., Morrow, E.M., Weiss, L.A., Menashe, I., Wadkins, T., Banerjee-Basu, S., and Packer, A. (2013). SFARI Gene 2.0: a community-driven knowledgebase for the autism spectrum disorders (ASDs). Mol. Autism *4*, 36. https://doi.org/10.1186/2040-2392-4-36.
39. Ouwenga, R.L., and Dougherty, J. (2015). Fmrp targets or not: long, highly brain-expressed genes tend to be implicated in autism and brain disorders. Mol. Autism *6*, 16. https://doi.org/10.1186/s13229-015-0008-1.
40. Duong, T. (2022). ks: Kernel Smoothing. R package version 1.14.0.
